# Reactivity Studies on the Terminal Thorium Imido Metallocene (η^5^‑C_5_Me_5_)_2_Th(=Ndipp)(dmap)

**DOI:** 10.1021/acs.inorgchem.6c01355

**Published:** 2026-06-28

**Authors:** Yi Heng, Xiaoying Cao, Wanjian Ding, Guohua Hou, Guofu Zi, Marc D. Walter

**Affiliations:** † Department of Chemistry, 47836 Beijing Normal University, Beijing 100875, China; ‡ Institut für Anorganische und Analytische Chemie, Technische Universität Braunschweig, Hagenring 30, 38106 Braunschweig, Germany

## Abstract

Treatment of the thorium dimethyl metallocene (η^5^-C_5_Me_5_)_2_ThMe_2_ (**1**) with dippNH_2_ (dipp = 2,6-^*i*^Pr_2_C_6_H_3_) in toluene in the presence of 4-dimethylaminopyridine (dmap) affords the terminal thorium imido metallocene (η^5^-C_5_Me_5_)_2_Th­(=Ndipp)­(dmap) (**4**), accompanied by methane releases. Complex **4** shows broad reactivity in small molecule activation. For example, **4** may activate S_8_, alkynes, carbodiimides, aldehydes, CS_2_, organic nitriles and isonitriles, and organic azides, yielding metallaheterocycle, pyridyl amido, oxido, and bis-amido complexes, respectively. Moreover, while **4** forms with Me_3_PO the adduct (η^5^-C_5_Me_5_)_2_Th­(=Ndipp)­(OPMe_3_) (**5**), the imido moiety may promote deprotonation reactions as established by its reactions with thiazole, pyridine-*N*-oxide derivative 2-MepyNO, amine mesitylNH_2_, hydrazine derivative PhNHNHPh, terminal alkyne PhC≡CH, cyclohexanone, amidate PhCONHPh, imine Ph_2_C=NH, and nitriles PhCH_2_CN and C_6_H_11_CN. In addition, the imido moiety may also behave as a nucleophile toward PhSiH_2_Cl, whereas **4** undergoes a Friedel–Crafts type reaction with Me_3_SiX (X = Cl, I, N_3_) and Ph_3_CN_3_. Lastly, **4** furnishes the bromo amido complex (η^5^-C_5_Me_5_)_2_Th­(Br)­[NH­(2,6-^*i*^Pr_2_-4-(Me_5_C_5_)­C_6_H_2_)] (**13**) in the presence of CuBr, while the C–C coupling product [(η^5^-C_5_Me_5_)_2_Th­(I)]_2_[μ-4,4′–(NH–2,6-^*i*^Pr_2_C_6_H_2_)_2_] (**14**) is isolated in the presence of AgI. Furthermore, a comparison with related terminal imido thorium metallocene derivatives demonstrates nicely how different substituents on the cyclopentadienyl and imido ligands influence the reactivity of these compounds.

## Graphical abstract

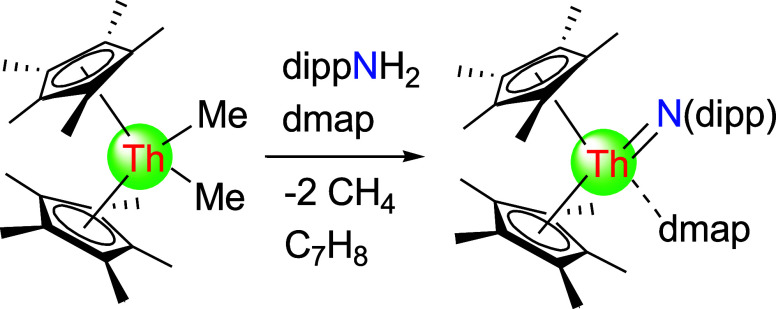

## Introduction

Terminal actinide imido complexes (An=NR) have garnered sustained interest over the past four decades because of their potential in small-molecule activation and catalysis.^1–4^ While numerous examples exist for thorium and uranium, the structure–reactivity relationships governing their reactivity still remain underexplored.^2–4^ This gap is particularly relevant given the broader context of actinide chemistry, where the degree of covalency, 5f/6d orbital participation,^5^ and steric effects critically influence reactivity.^6^ As part of our long-standing interest in organoactinide complexes featuring An-element multiple bonds,^7,8^ we prepared, for example, the Lewis base-stabilized thorium imido complex, [η^5^-1,3-(Me_3_C)_2_C_5_H_3_]_2_Th=N­(dipp)­(dmap),^8b^ which was previously shown to activate S_8_, conjugated alkynes, ketones, thio-ketones, CS_2_, isothiocyanates, seleno-ketones, esters, diazabutadienes, carbodiimides, and organic azides.^8b^ Furthermore, it deprotonates thiazole, terminal alkynes, and nitriles, and exhibits Cannizzaro-type reactivity with aldehydes and Friedel–Crafts alkylation with Ph_3_CN_3_.^8b^ These results underscore the synthetic versatility of terminal thorium imidos. To elucidate the role of ligand sterics, we now report the synthesis and reactivity of the pentamethylcyclopentadienyl (C_5_Me_5_) analog. Previous studies have already indicated that the reactivity patterns of organoactinide complexes are significantly altered by the steric effects.^7,8^ To further explore this aspect, we detail, herein, some observations regarding the synthesis, structure, and structure–reactivity relationship of the terminal thorium imido metallocene (η^5^-C_5_Me_5_)_2_Th­(=Ndipp)­(dmap) (**4**) and compare its reactivity to related, but less sterically congested derivatives.^9^

## Results and Discussion

### Synthesis of (η^5^-C_5_Me_5_)_2_Th­(=Ndipp)­(dmap) (**4**)

Reaction of the thorium dimethyl complex (η^5^-C_5_Me_5_)_2_ThMe_2_ (**1**) with 2 equiv of dippNH_2_ (dipp = 2,6-^*i*^Pr_2_C_6_H_3_) yields nearly quantitatively the bis-amido complex (η^5^-C_5_Me_5_)_2_Th­(NHdipp)_2_ (**2**) and methane (Scheme 1). X-ray diffraction analysis reveals a distorted tetrahedral environment at the Th­(IV) atom, with Th–N bond lengths of 2.343(4) and 2.340(4) Å and an N(1)–Th–N(2) angle of 99.8(1)° (Figure 1 and Table 1). In contrast, treatment of **1** with 1 equiv of dippNH_2_ in the presence of pyridine affords the amido pyridyl complex (η^5^-C_5_Me_5_)_2_Th­[NH­(dipp)]­(κ^2^-*C*,*N*–C_5_H_4_N) (**3**) also in excellent yield (Scheme 1). The molecular structure (Figure 2 and Table 1) features a chelating pyridyl ligand with Th–N(1) = 2.367(7) Å and Th–N(2) = 2.483(8) Å, along with a Th–C(33) distance of 2.444(8) Å. We propose that this product arises from deprotonation of a transient pyridine adduct (η^5^-C_5_Me_5_)_2_Th­(=Ndipp)­(py) (Scheme 1). To favor terminal imido formation, we employed the more basic Lewis base 4-dimethylaminopyridine (dmap). Indeed, heating **1** with 1 equiv of dippNH_2_ and dmap in toluene at 80 °C yields the terminal imido complex (η^5^-C_5_Me_5_)_2_Th­(=Ndipp)­(dmap) (**4**) in 82% isolated yield (Scheme 1). Notably, **4** can also be accessed via **2** with 1 equiv of (η^5^-C_5_Me_5_)_2_ThMe_2_ (**1**) and in the presence of dmap (Scheme 1). X-ray crystallography confirms the Th–N(1) bond length of 2.072(12) Å (Figure 3 and Table 1), consistent with other thorium imidos (e.g., 2.038(3)–2.091(7) Å).^7b,7f,8a,8b^ The Th–N(2) distance of 2.608(5) Å suggests dative coordination, contrasting the bis­(dmap) adduct (η^5^-C_5_Me_5_)_2_Th=N­(*p*-tolyl)­(dmap)_2_, which crystallizes with two dmap ligands.^8c^ This difference is attributed to the steric bulk of the 2,6-^*i*^Pr_2_C_6_H_3_ group, which favors a 1:1 adduct. In C_6_D_6_ solution, however, ^1^H NMR spectroscopy reveals an equilibrium between **4** and the amido pyridyl complex (η^5^-C_5_Me_5_)_2_Th­(NHdipp)­[κ^2^-*C*,*N*-4-(Me_2_N)­C_5_H_3_N] (**4′**) with *K*_eq_ = [**4**]/[**4′**] ≈ 1.5 at 293 K, which translates to Δ*G*_exp_(293 K) = 0.24 kcal/mol for the equilibrium **4** ⇌ **4′**. This behavior parallels (η^5^-C_5_Me_5_)_2_Th=N­(mesityl)­(dmap),^7f^ but contrasts with less sterically encumbered complexes like [η^5^-1,3-(Me_3_C)_2_C_5_H_3_]_2_Th=N­(dipp)­(dmap) and (η^5^-C_5_Me_5_)_2_Th=N­(*p*-tolyl)­(dmap)_2_,^8b,8c^ which show no isomerization. The equilibrium is driven by steric and electronic factors, as supported by DFT calculations, suggesting that an α-H atom of dmap is transferred to the Th=N­(dipp) moiety via the transition state **TS4** (Figure 4). The formation of **4′** from **4** is energetically slightly unfavorable (Δ*G*_DFT_(298 K) = 0.5 kcal/mol) with a reaction barrier of Δ*G* ^‡^(298 K) = 23.9 kcal/mol, which indicates that an equilibrium between **4** and **4′** may exist in the solution, favoring **4**, consistent with the observations detected by NMR spectroscopy. Furthermore, the computed Δ*G*_DFT_ is in good agreement with the experimental data. The solution-phase equilibrium suggests that **4** may exhibit distinct reactivity from its analogues. While [η^5^-1,3-(Me_3_C)_2_C_5_H_3_]_2_Th=N­(dipp)­(dmap) is stable as the imido complex and (η^5^-C_5_Me_5_)_2_Th=N­(*p*-tolyl)­(dmap)_2_ forms an amido pyridyl complex (η^5^-C_5_Me_5_)_2_Th­[NH­(*p*-tolyl)]­[κ^2^-*C*,*N*-4-(Me_2_N)­C_5_H_3_N] and a dmap in C_6_D_6_ solution,^8b,8c,9^ the dynamic isomerization of **4** implies that its reactivity could be tuned by ligand choice or reaction conditions.

**Scheme 1:**
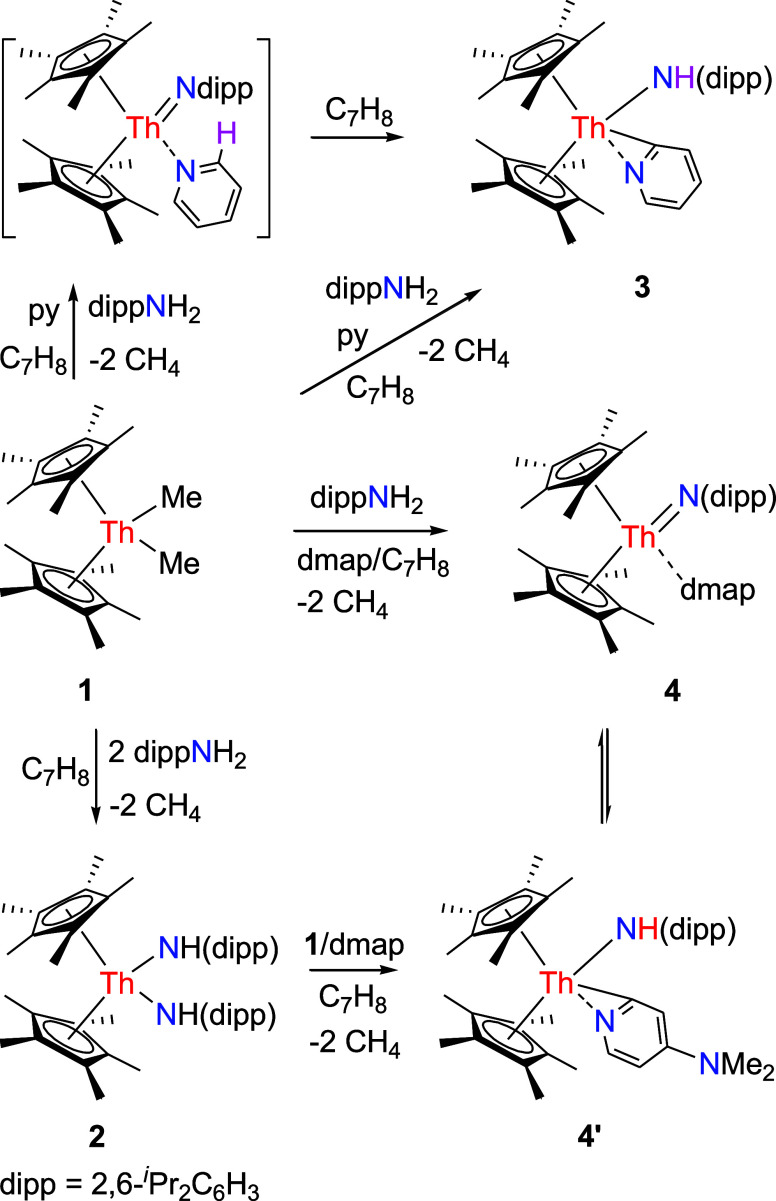
Synthesis of Compounds **2–4**

**Figure 1:**
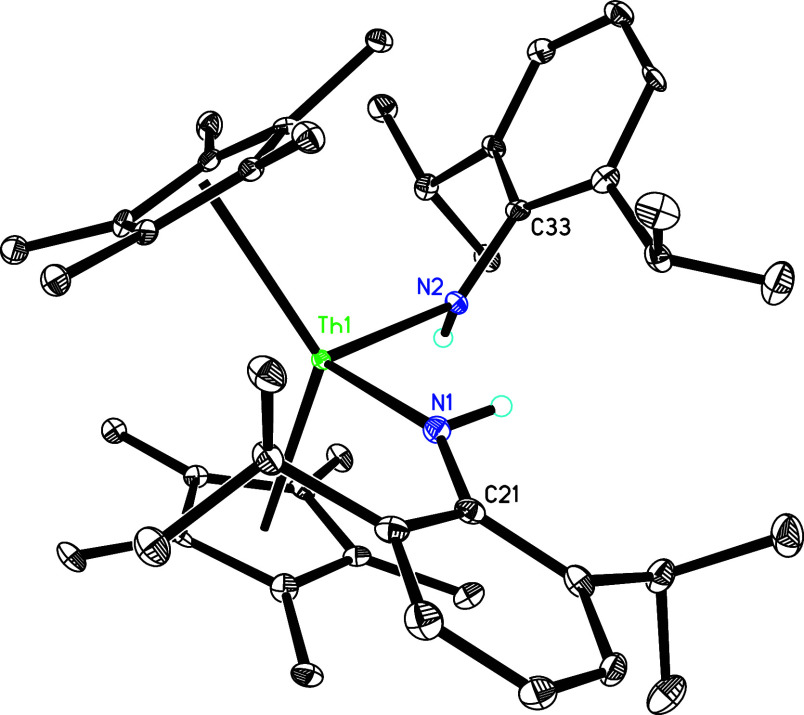
Molecular structure of **2** (thermal ellipsoids drawn at the 35% probability level).

**Table 1:** Selected Distances (Å) and Angles (deg) for Compounds **2–7**, **9–19**, **21–32** ^a^

compound	C(Cp)-Th^b^	C(Cp)-Th^c^	Cp(cent)-Th	Th-X	Cp(cent)-Th-Cp(cent)	X-Th-X/Y
**2**	2.850(8)	2.817(5) to 2.884(4)	2.566(4), 2.593(4)	N(1) 2.343(4), N(2) 2.340(3)	129.0(1)	99.8(1)
**3**	2.837(6)	2.817(9) to 2.867(9)	2.555(9), 2.575(9)	N(1) 2.367(7), N(2) 2.483(8)	136.2(3)	31.6(3)^d^
				C(33) 2.444(8)		
**4**	2.893(17)	2.813(13) to 2.958(12)	2.629(12), 2.632(12)	N(1) 2.072(12), N(2) 2.608(5)	126.8(3)	96.3(3)
**5**	2.859(11)	2.817(6) to 2.919(5)	2.597(5), 2.585(5)	N(1) 2.086(4), O(1) 2.390(4)	131.4(2)	100.2(2)
**6**	2.823(6)	2.800(5) to 2.856(6)	2.560(6), 2.544(6)	N(1) 2.344(4), N(2) 2.558(4)	134.9(3)	31.2(3)^d^
				C(33) 2.484(6)		
**7**	2.847(15)	2.779(17) to 2.913(10)	2.589(10), 2.564(10)	N(1) 2.378(10), O(1) 2.434(8)	131.2(3)	53.8(3)^e^
				C(33) 2.607(12)		
**9**	2.855(11)	2.810(7) to 2.910(4)	2.603(4), 2.571(4)	N(1) 2.327(3), N(2) 2.298(4)	127.8(3)	37.0(1)^f^
				N(3) 2.592(4)		
**10**	2.819(7)	2.787(4) to 2.851(4)	2.547(4), 2.549(4)	N(1) 2.616(3), Cl(1) 2.705(1)	134.1(1)	147.9(1)^g^
				Cl(2) 2.746(1)		
**11**	2.800(8)	2.767(11) to 2.831(11)	2.536(8), 2.520(8)	N(1) 2.308(7), Cl(1) 2.679(2)	133.5(3)	122.3(2)
**12**	2.802(7)	2.763(9) to 2.834(8)	2.529(8), 2.526(8)	N(1) 2.286(6), I(1) 3.084(1)	133.7(3)	118.8(2)
**13**	2.813(12)	2.739(11) to 2.851(13)	2.553(11), 2.539(11)	N(1) 2.284(6), Br(1) 2.854(1)	135.1(3)	115.2(2)
**14**	Th(1)	Th(1)	Th(1)	Th(1)	Th(1)	Th(1)
	2.815(3)	2.745(4) to 2.840(5)	2.526(4), 2.544(4)	N(1) 2.315(4), I(1) 3.099(1)	132.4(3)	107.3(1)
	Th(2)	Th(2)	Th(2)	Th(2)	Th(2)	Th(2)
	2.810(12)	2.756(5) to 2.873(4)	2.524(5), 2.549(5)	N(2) 2.284(4), I(2) 3.114(1)	133.8(3)	116.2(1)
**15**	2.820(3)	2.805(4) to 2.832(5)	2.547(4), 2.549(4)	S(1) 2.802(1), S(3) 2.806(1)	129.0(3)	112.7(1)
**16**	2.871(5)	2.838(5) to 2.930(5)	2.613(5), 2.604(5)	N(1) 2.473(4), N(2) 2.441(4)	125.3(2)	32.3(2)^h^
				C(42) 2.457(5)		
**17**	2.866(13)	2.805(5) to 2.920(4)	2.597(4), 2.604(4)	N(1) 2.448(4), N(2) 2.428(4)	124.3(2)	32.3(1)^i^
				C(49) 2.447(5)		
**18**	2.811(11)	2.756(6) to 2.861(6)	2.544(6), 2.532(6)	N(1) 2.303(5), C(33) 2.469(7)	135.2(2)	112.9(2)
**19**	2.906(14)	2.859(14) to 2.983(13)	2.666(13), 2.620(13)	N(1) 2.425(16), N(3) 2.363(17)	124.8(4)	57.1(5)^j^
				N(4) 2.655(8)		
**21**	2.829(12)	2.779(8) to 2.880(8)	2.565(8), 2.550(8)	N(1) 2.335(6), O(1) 2.174(5)	132.3(3)	111.9(2)
**22**	2.823(13)	2.780(13) to 2.886(13)	2.565(13), 2.547(13)	S(1) 2.822(4), S(2) 2.806(4)	134.4(3)	64.1(1)^k^
				N(2) 2.631(7)		
**23**	2.835(10)	2.805(15) to 2.888(15)	2.550(15), 2.584(15)	N(1) 2.582(12), N(2) 2.580(13)	139.2(4)	51.2(3)^l^
				O(1) 2.498(9), O(2) 2.493(10)		51.0(4)^m^
**24**	2.817(5)	2.796(3) to 2.838(3)	2.537(3), 2.554(3)	N(1) 2.267(3), N(2) 2.280(3)	140.4(1)	110.6(1)
**25**	2.843(10)	2.806(5) to 2.887(5)	2.565(4), 2.583(4)	N(1) 2.117(4), N(3) 2.560(4)	131.9(2)	93.9(1)
**26**	2.816(10)	2.775(6) to 2.863(6)	2.537(6), 2.548(6)	N(1) 2.608(5), N(2) 2.421(5)	130.0(2)	53.1(2)^f^
				N(3) 2.426(5)		
**27**	2.845(10)	2.797(10) to 2.882(12)	2.553(10), 2.581(10)	N(1) 2.586(13), N(2) 2.418(10)	127.9(2)	52.5(3)^f^
				N(3) 2.335(12)		
**28**	2.841(8)	2.809(5) to 2.875(4)	2.574(4), 2.567(4)	N(2) 2.591(4), N(3) 2.437(4)	139.9(1)	52.2(1)^n^
				N(4) 2.394(4)		
**29**	2.874(10)	2.841(4) to 2.929(3)	2.596(3), 2.621(3)	N(1) 2.395(3), N(5) 2.257(3)	125.2(1)	85.6(1)
**30**	2.826(8)	2.797(6) to 2.868(5)	2.556(5), 2.554(5)	N(1) 2.310(5), N(2) 2.527(5)	135.9(2)	51.7(1)^o^
				N(5) 2.617(5)		
**31**	2.802(11)	2.754(7) to 2.852(5)	2.531(5), 2.538(5)	N(1) 2.284(5), N(2) 2.370(6)	135.5(2)	114.2(2)
**32**	2.795(4)	2.777(7) to 2.814(7)	2.533(7), 2.516(7)	N(1) 2.309(6), N(2) 2.338(6)	137.2(2)	118.4(2)

a Cp is the cyclopentadienyl ring.

b Average value, the value in parentheses is the standard deviation of the mean.

c Range.

d Angle of N(2)–Th(1)–C(33).

e Angle of C(33)–Th(1)–O(1).

f Angle of N(1)–Th(1)–N(2).

g Angle of Cl(1)–Th(1)–Cl(2).

h Angle of C(42)–Th(1)–N(2).

i Angle of N(2)–Th(1)–C(49).

j Angle of N(1)–Th(1)–N(3).

k Angle of S(1)–Th(1)–S(2).

l Angle of O(1)–Th(1)–N(1).

m Angle of O(2)–Th(1)–N(2).

n Angle of N(2)–Th(1)–N(3).

o Angle of N(2)–Th(1)–N(5).

**Figure 2:**
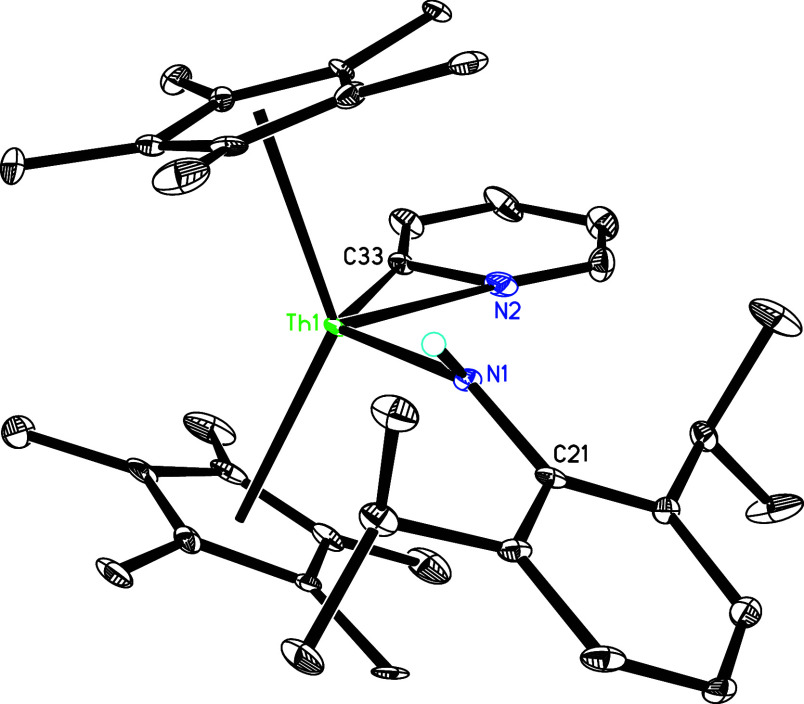
Molecular structure of **3** (thermal ellipsoids drawn at the 35% probability level).

**Figure 3:**
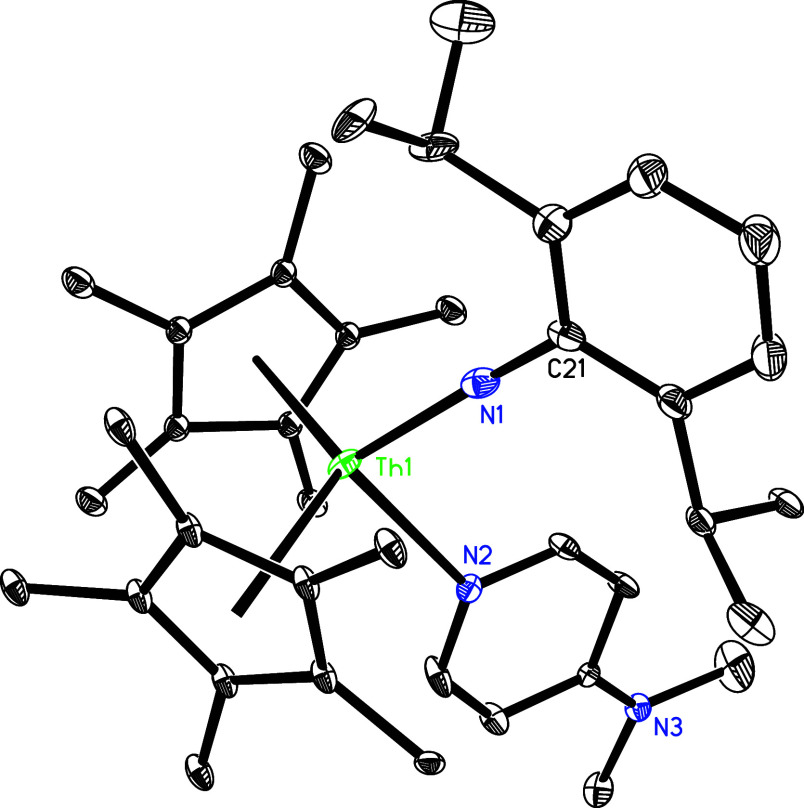
Molecular structure of **4** (thermal ellipsoids drawn at the 35% probability level).

**Figure 4:**
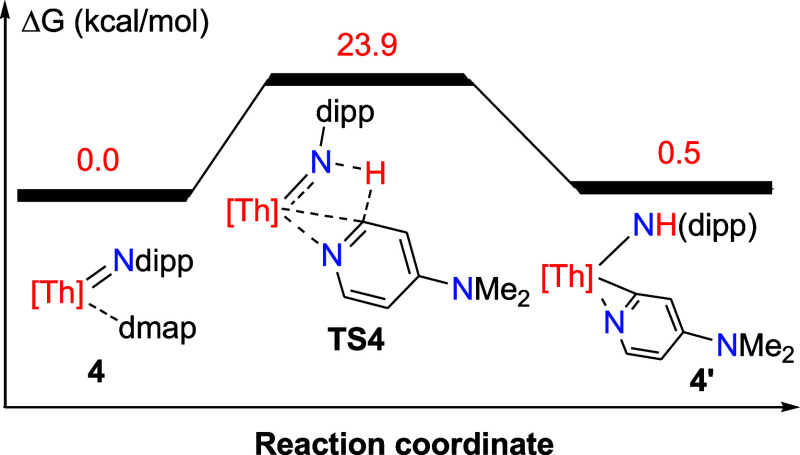
Free energy profile (kcal/mol) for the reaction of **4** ⇌ **4′**. [Th]=(η^5^-C_5_Me_5_)_2_Th.

### Reactivity Studies

Having established the solid-state structure of the Lewis-base-supported terminal imido, (η^5^-C_5_Me_5_)_2_Th­(=Ndipp)­(dmap) (**4**), we examined its reactivity toward a series of small-molecule substrates. For clarity, the product distribution from **4** is compared with that obtained from the previously reported thorium imido systems (η^5^-C_5_Me_5_)_2_Th=N­(*p*-tolyl)­(dmap)_2_ (Figure S1),^8c,9^ (η^5^-C_5_Me_5_)_2_Th=N­(mesityl)­(dmap) (Figure S2),^7f,7g,9^ [η^5^-1,3-(Me_3_C)_2_C_5_H_3_]_2_Th=N­(dipp)­(dmap) (Figure S3),^8b,9^ [η^5^-1,2,4-(Me_3_C)_3_C_5_H_2_]_2_Th=N­(*p*-tolyl) (Figure S4),^7a,7b,7c,7d,7e^−,^9,9^ and [η^5^-1,2,4-(Me_3_Si)_3_C_5_H_2_]_2_Th=N­(*p*-tolyl)­(bipy) (Figure S5).^8a,9^

### Reaction with Lewis Bases, Amines, and Hydrazine Derivatives

In C_6_D_6_ solution **4** exists in equilibrium with the amido-pyridyl isomer **4′** (*K*_eq_ = [**4**]/[**4′**] ≈ 1.5 at 293 K), a behavior that mirrors that of (η^5^-C_5_Me_5_)_2_Th=N­(mesityl)­(dmap).^7f^ The coordinated dmap ligand in **4** is, however, labile and can be displaced by a range of external Lewis bases. For example, treatment of **4** with trimethylphosphine oxide (Me_3_PO) furnishes the phosphine-oxide adduct (η^5^-C_5_Me_5_)_2_Th­(=Ndipp)­(OPMe_3_) (**5**) in 95% isolated yield (Scheme 2). Its molecular structure (Figure 5 and Table 1) shows a Th–O distance of 2.390(4) Å, whereas the short Th–N bond of 2.086(4) Å is consistent with a genuine Th=N double bond.^7b^ The formation of **5** is reminiscent of the Me_3_PO adduct of [η^5^-1,3-(Me_3_C)_2_C_5_H_3_]_2_Th=N­(dipp)­(OPMe_3_) (Figure S3),^8b,9^ whereas (η^5^-C_5_Me_5_)_2_Th=N­(*p*-tolyl)­(dmap)_2_ forms an amido alkyl (η^5^-C_5_Me_5_)_2_Th­[NH­(*p*-tolyl)]­[κ^2^-*C*,*N*–N­(*p*-tolyl)­P­(Me_2_)­CH_2_] with Me_3_PO (Figure S1).^8c,9^ The difference between **4** and (η^5^-C_5_Me_5_)_2_Th=N­(*p*-tolyl)­(dmap)_2_ can be ascribed to the more steric bulk of the 2,6-^*i*^Pr_2_C_6_H_3_ substituent. Nevertheless, when **4** is reacted with thiazole, the expected thiazole-bound imido adduct is not isolated. Instead, quantitative formation of κ^2^-*C*,*N*-thiazolyl amido complex (η^5^-C_5_Me_5_)_2_Th­[NH­(dipp)]­(κ^2^-*C*,*N*–C_3_H_2_NS) (**6**) occurs with concomitant loss of dmap (Scheme 2). We propose that thiazole initially displaces dmap to give a transient thiazole-imido species, which then undergoes intramolecular deprotonation of the coordinated thiazole ring to generate the κ^2^-*C*,*N*-thiazolyl amido product **6**. The ORTEP diagram (Figure 6 and Table 1) shows a Th–N(2) distance of 2.558(4) Å (dative interaction) and a short Th–C(33) distance of 2.484(6) Å, while the Th–N(1) distance is significantly shorter with 2.344(4) Å, consistent with a Th-amido bond. Analogous thiazole-mediated amido formation has been observed for (η^5^-C_5_Me_5_)_2_Th=N­(mesityl)­(dmap) (Figure S2),^7g,9^ [η^5^-1,3-(Me_3_C)_2_C_5_H_3_]_2_Th=N­(dipp)­(dmap) and [η^5^-1,2,4-(Me_3_Si)_3_C_5_H_2_]_2_Th=N­(*p*-tolyl)­(bipy) (Figures S3 and S5).^8a,8b,9^

**Scheme 2:**
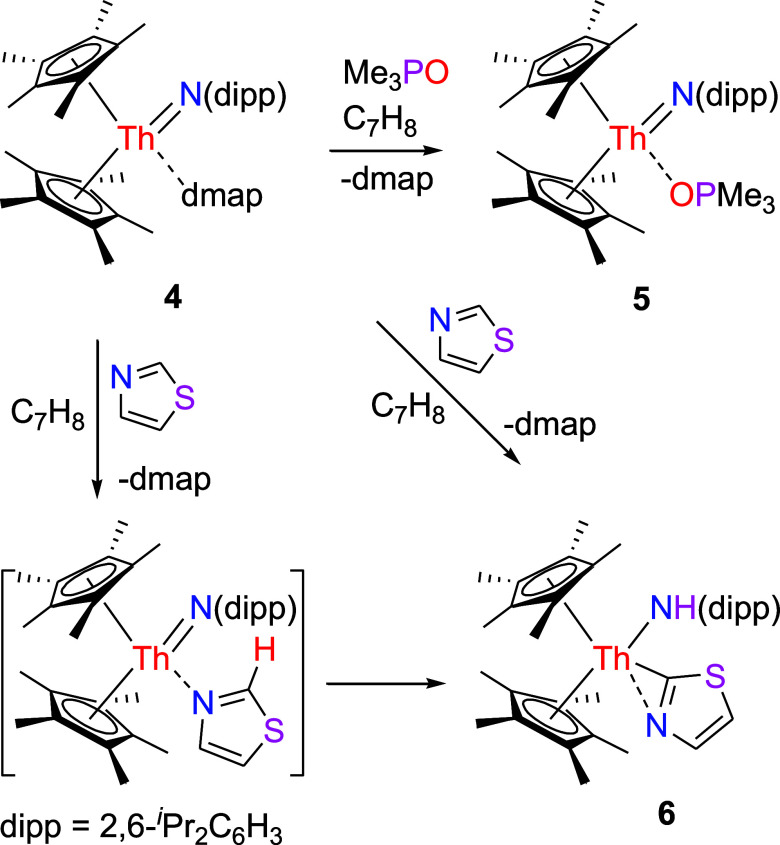
Synthesis of Compounds **5** and **6**

**Figure 5:**
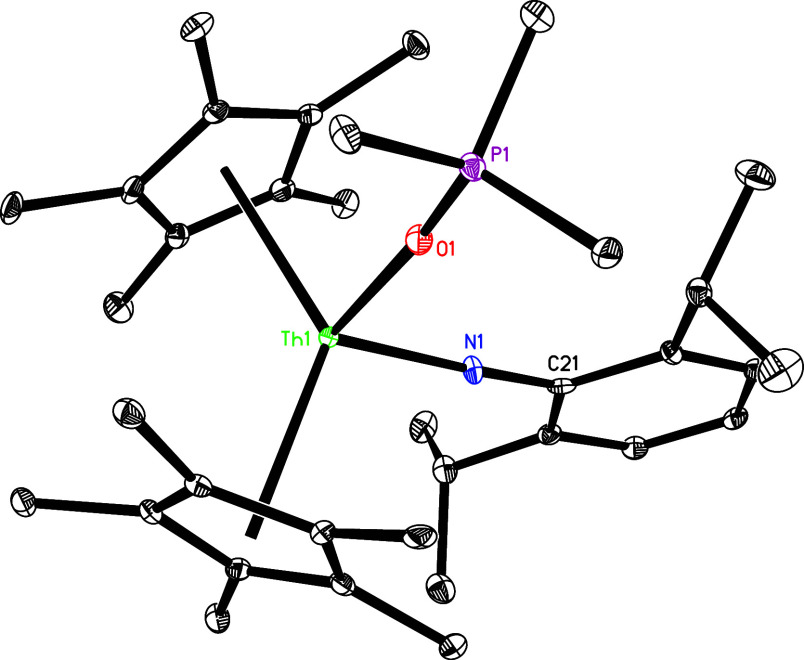
Molecular structure of **5** (thermal ellipsoids drawn at the 35% probability level).

**Figure 6:**
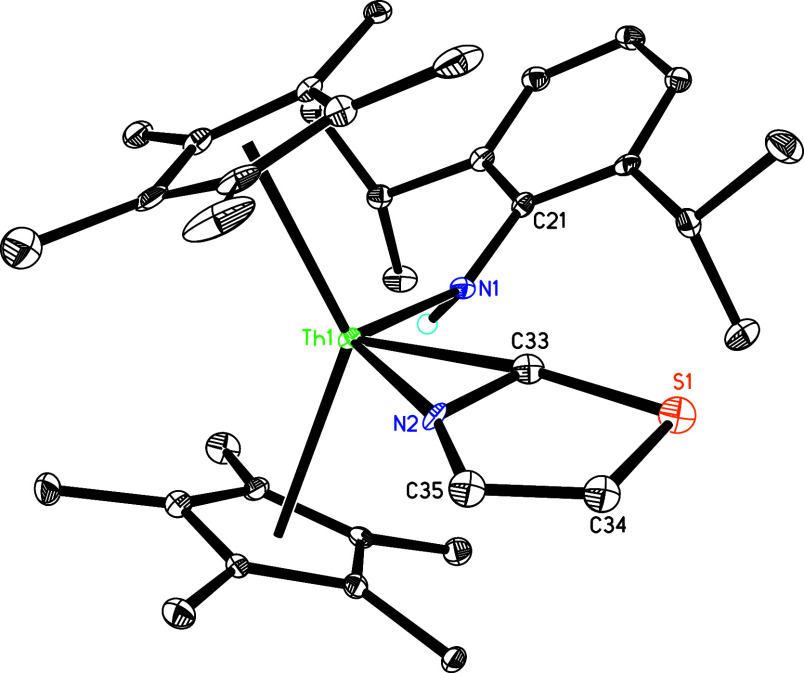
Molecular structure of **6** (thermal ellipsoids drawn at the 35% probability level).

Moreover, when **4** is treated with 2-methylpyridine *N*-oxide in toluene, quantitative conversion to the amido-pyridinium-oxide complex (η^5^-C_5_Me_5_)_2_Th­(NHdipp)­(κ^2^-*C*,*O*-2-MeC_5_H_3_NO) (**7**) is observed, accompanied by loss of dmap (Scheme 3). The transformation proceeds by an α-H atom from the *N*-oxide to the imido Th=N­(dipp) moiety. Notably, the methyl sp^3^–C–H bond remains untouched, indicating that the sp^2^ C–H bond is preferentially deprotonated. This reactivity resembles that observed for [η^5^-1,2,4-(Me_3_C)_3_C_5_H_2_]_2_Th=N­(*p*-tolyl) (Figure S4).^7e,9^ The solid-state structure of **7** (Figure 7 and Table 1) displays a Th–O distance of 2.434(8) Å, whereas the Th–C(33) and Th–N(1) distances are 2.607(12) and 2.378(10) Å, respectively. In addition, the imido moiety in **4** can deprotonate sterically demanding primary amines. For example, treatment of **4** with 2 equiv of mesitylNH_2_ furnishes the known bis-amido complex (η^5^-C_5_Me_5_)_2_Th­(NHmesityl)_2_ (**8**),^7f^ in quantitative yield concomitant with dippNH_2_ and dmap release (Scheme 3). Furthermore, addition of 1,2-diphenylhydrazine PhNHNHPh to **4** results in deprotonation and quantitative formation of the three-membered *N*-bisaryl–hydrazido complex (η^5^-C_5_Me_5_)_2_Th­[N­(Ph)­N­(Ph)]­(dmap) (**9**) together with the liberation of dippNH_2_ (Scheme 3). The molecular structure of **9** is shown in Figure 8, and selected bond distances and angles are listed in Table 1. The relatively long Th–N(3) distance of 2.592(4) Å is consistent with a datively coordinated nitrogen atom, whereas the Th–N(1) and Th–N(2) distances are 2.327(3) and 2.298(4) Å, respectively.

**Scheme 3:**
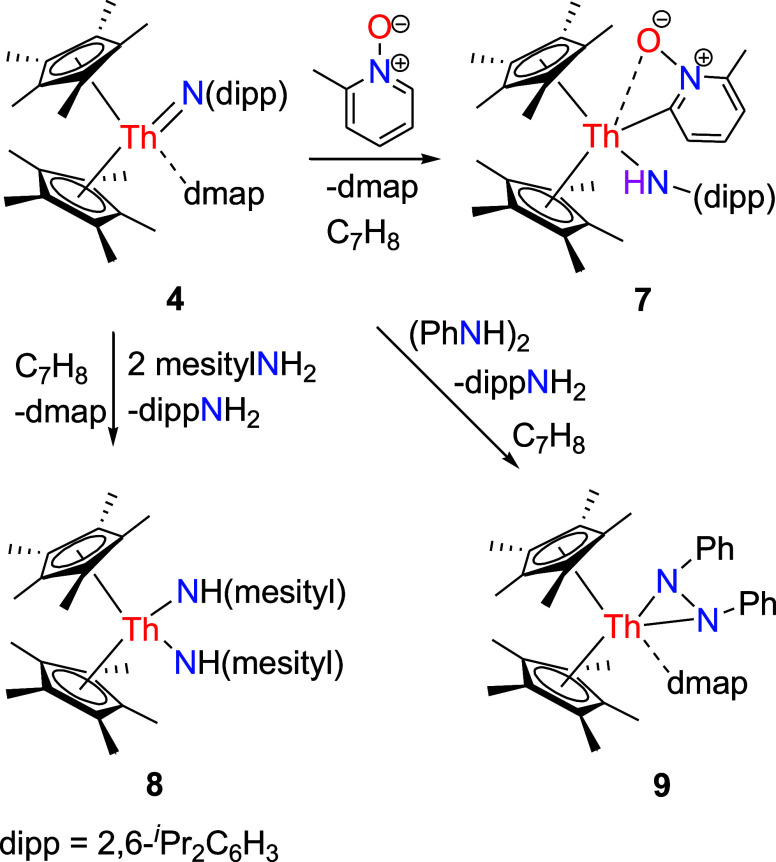
Synthesis of Compounds **7–9**

**Figure 7:**
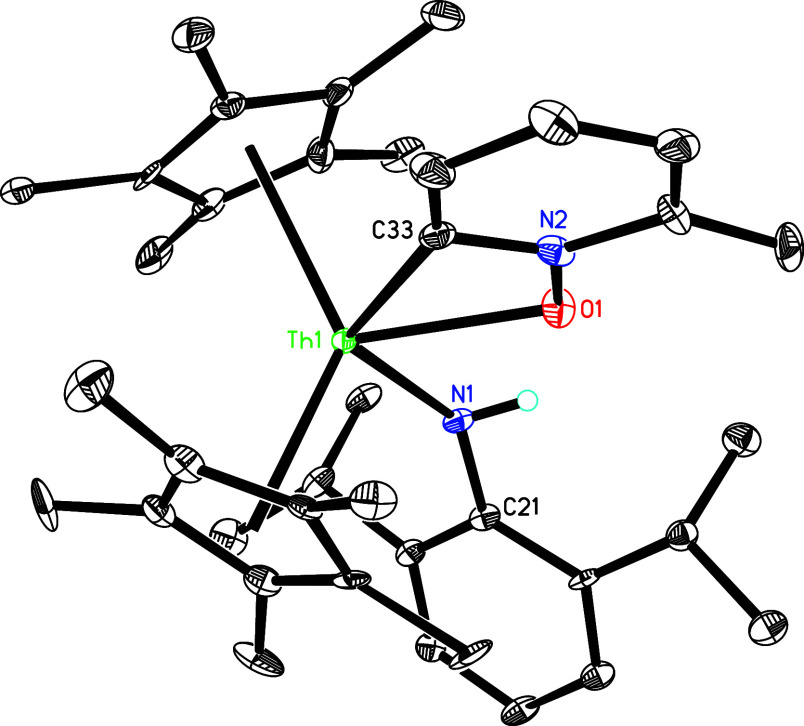
Molecular structure of **7** (thermal ellipsoids drawn at the 35% probability level).

**Figure 8:**
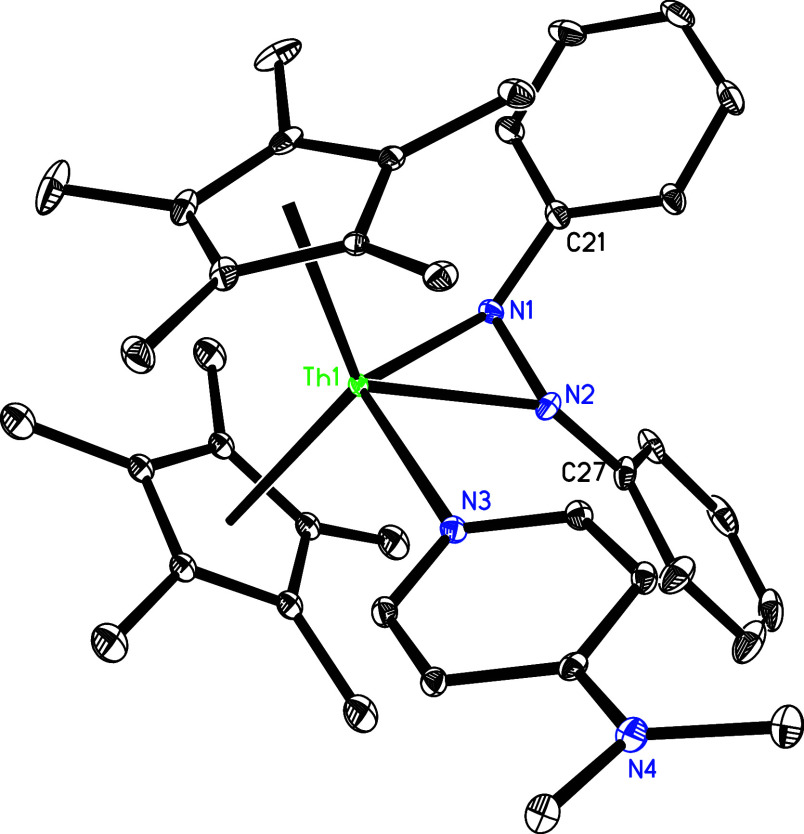
Molecular structure of **9** (thermal ellipsoids drawn at the 35% probability level).

### Reaction with Halidosilanes, Metal Halides, and Elemental Sulfur

The terminal imido **4** reacts with PhSiH_2_Cl to give the dichloride complex (η^5^-C_5_Me_5_)_2_ThCl_2_(dmap) (**10**) together with the imido-derived amine dippN­(SiH_2_Ph)_2_ in quantitative conversion (Scheme 4). In this transformation the Th=N­(dipp) fragment functions as a nucleophile, attacking the silicon atom. The molecular structure of **10** (Figure 9 and Table 1) shows two Th–Cl bonds (2.705(1) (Th–Cl(1)) and 2.746(1) Å (Th–Cl(2))) and a dative Th–N­(dmap) distance of 2.616(3) Å (Th–N(1)). The Cl–Th–Cl angle is 147.9(1)°, consistent with a distorted trigonal-bipyramidal coordination sphere at the Th atom. For the imido complexes bearing more sterically encumbered Cp ligands such as [η^5^-1,2,4-(Me_3_C)_3_C_5_H_2_]_2_Th=N­(*p*-tolyl) and [η^5^-1,2,4-(Me_3_Si)_3_C_5_H_2_]_2_Th=N­(*p*-tolyl)­(bipy) the reaction can be stopped at the amido chloride intermediate [η^5^-1,2,4-(Me_3_E)_3_C_5_H_2_]_2_Th­(Cl)­[N­(*p*-tolyl)­SiH_2_Ph] (E = C,^7e^ Si^8a^) (Figures S4 and S5),^9^ while [η^5^-1,3-(Me_3_C)_2_C_5_H_3_]_2_Th=N­(dipp)­(dmap) (Figure S3) shows analogous reactivity as **4** toward PhSiH_2_Cl.^8b,9^ Nevertheless, the same outcome does not occur for the reaction of (η^5^-C_5_Me_5_)_2_Th=N­(*p*-tolyl)­(dmap)_2_ with PhSiH_2_Cl, which instead furnishes the chloro pyridyl complex (η^5^-C_5_Me_5_)_2_Th­(Cl)­[κ^2^-*C*,*N*-4-(Me_2_N)­C_5_H_3_N] (Figure S1),^8c,9^ as a consequence of the reduced steric hindrance of the *p*-tolyl group.

**Scheme 4:**
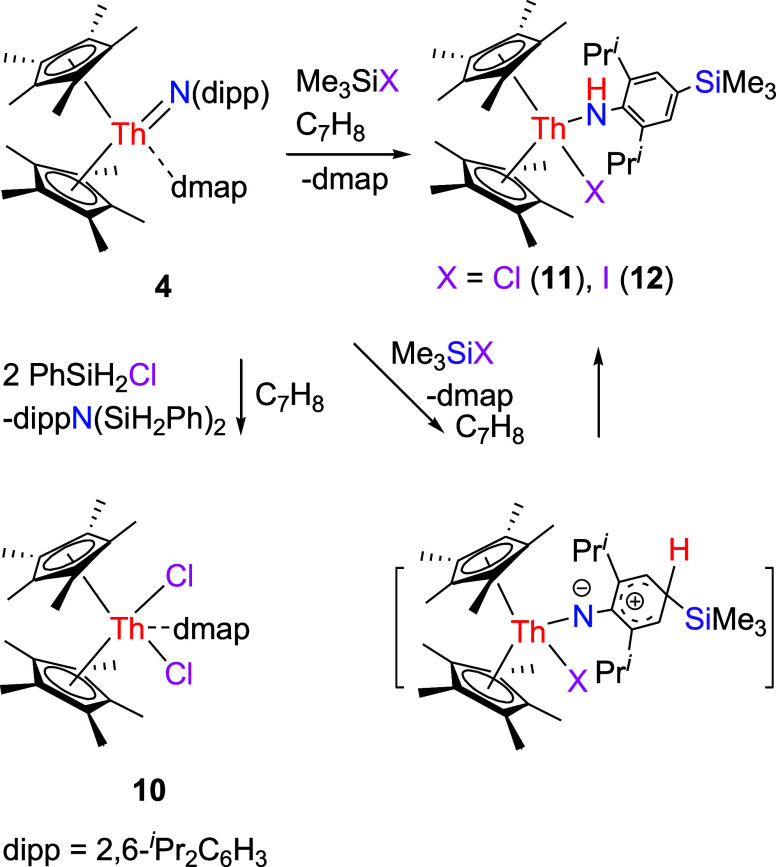
Synthesis of Compounds **10–12**

**Figure 9:**
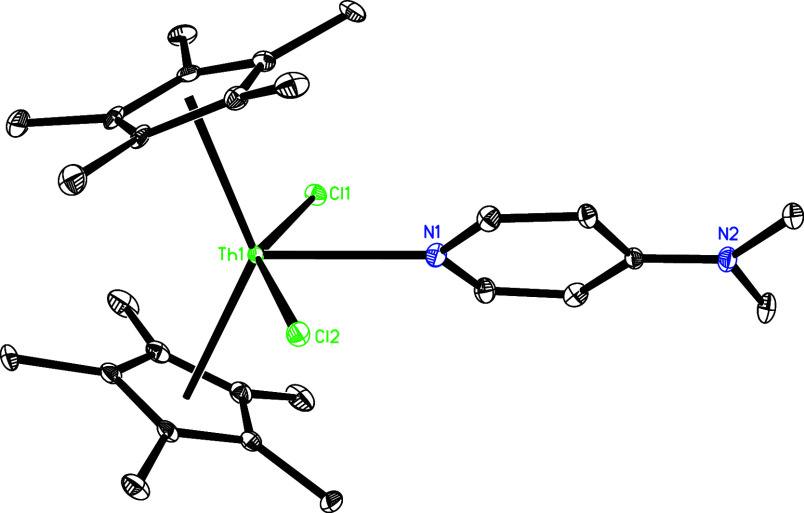
Molecular structure of **10** (thermal ellipsoids drawn at the 35% probability level).

However, when **4** is treated with the sterically demanding halidosilanes Me_3_SiCl and Me_3_SiI, no dichloro/diiodo products are observed. Instead, a Friedel–Crafts type reaction C–Si bond formation occurs, delivering the chloro amido complex (η^5^-C_5_Me_5_)_2_Th­(Cl)­[NH­(2,6-^*i*^Pr_2_-4-(Me_3_Si)­C_6_H_2_)] (**11**) and iodo amido complex (η^5^-C_5_Me_5_)_2_Th­(I)­[NH­(2,6-^*i*^Pr_2_-4-(Me_3_Si)­C_6_H_2_)] (**12**), respectively, in good isolated yields (Scheme 4). The molecular structure of **11** (Figure 10 and Table 1) reveals a Th–N(1) bond of 2.308­(**7**) and Th–Cl(1) bond of 2.679­(**2**) Å. The ORTEP diagram of **12** is provided in the Supporting Information (Figure S7 and Table 1). While the Th–N(1) distance of 2.286(6) is close to that in **11**, the Th–I(1) distance of 3.084(1) Å is significantly larger, consistent with the larger ionic radius of iodide.

**Figure 10:**
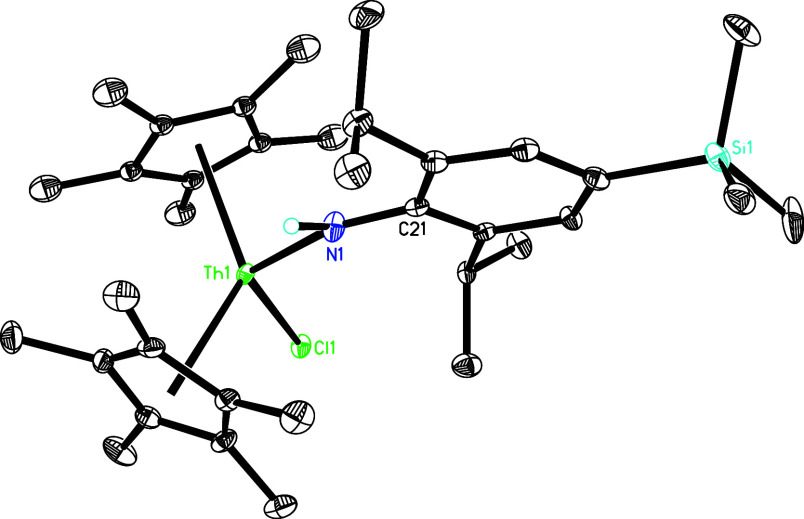
Molecular structure of **11** (thermal ellipsoids drawn at the 35% probability level).

Moreover, **4** readily reacts with CuBr, affording the bromo amido complex (η^5^-C_5_Me_5_)_2_Th­(Br)­[NH­(2,6-^*i*^Pr_2_-4-(Me_5_C_5_)­C_6_H_2_)] (**13**) in 34% yield (Scheme 5). This differentiates its reactivity from those of related thorium imido complexes (η^5^-C_5_Me_5_)_2_Th=N­(*p*-tolyl)­(dmap)_2_, (η^5^-C_5_Me_5_)_2_Th=N­(mesityl)­(dmap), and [η^5^-1,3-(Me_3_C)_2_C_5_H_3_]_2_Th=N­(dipp)­(dmap) (Figures S1–S3).^7f,8b,8c,9^ For example, (η^5^-C_5_Me_5_)_2_Th=N­(mesityl)­(dmap) reacts with CuBr to yield the heterobimetallic compound (η^5^-C_5_Me_5_)_2_Th­(Br)­[N­(mesityl)­Cu­(dmap)] (Figure S2),^7f,9^ while (η^5^-C_5_Me_5_)_2_Th=N­(*p*-tolyl)­(dmap)_2_ affords the dibromide complex (η^5^-C_5_Me_5_)_2_ThBr_2_(dmap) (Figure S1),^8c,9^ and [η^5^-1,3-(Me_3_C)_2_C_5_H_3_]_2_Th=N­(dipp)­(dmap) forms with CuCl a heterobimetallic complex [η^5^-1,3-(Me_3_C)_2_C_5_H_3_]_2_Th­(Cl)­[N­(dipp)­Cu­(dmap)] (Figure S3).^8b,9^ The reactivity differences between **4** and related imido derivatives are most likely attributed to the increased sterics around the metal Th. Although no intermediates were observed by NMR spectroscopy, we assume that **4** is initially oxidized by CuBr to yield the 5-bromo-1,2,3,4,5-pentamethyl-1,3-cyclopentadiene (Me_5_C_5_Br),^10^ elemental copper, and other unidentified thorium complexes (Scheme 5). In the next step, Me_5_C_5_Br proceeds via a Friedel–Crafts alkylation with a second molecule of **4** to yield the product **13** (Scheme 5). The molecular structure of **13** is shown in Figure 11, and selected bond distances and angles are listed in Table 1. The Th–N(1) distance is 2.284(6) Å, whereas the Th–Br(1) distance is 2.854(1) Å. Moreover, unlike (η^5^-C_5_Me_5_)_2_Th=N­(*p*-tolyl)­(dmap)_2_, which reacts with AgF to yield the difluoro complex (η^5^-C_5_Me_5_)_2_ThF_2_(dmap) (Figure S1),^8c,9^ the bulk-protected imido **4** undergoes a Friedel–Crafts type reaction with AgI to give an iodo imido intermediate **B**, which collapses by the elimination of Ag metal to yield the C–C coupled binuclear complex [(η^5^-C_5_Me_5_)_2_Th­(I)]_2_[μ-4,4′–(NH–2,6-^*i*^Pr_2_C_6_H_2_)_2_] (**14**) in good yield (Scheme 5), again, presumably due to the sterically encumbered 2,6-^*i*^Pr_2_C_6_H_3_ group. The ORTEP diagram of **14** (Figure 12 and Table 1) displays the Th(1)–N(1) and Th(1)–I(1) distances of 2.315(4) and 3.099(1) Å, respectively, which are comparable to those of Th(2)–N(2) (2.284(4) Å) and Th(2)–I(2) (3.114(1) Å).

**Scheme 5:**
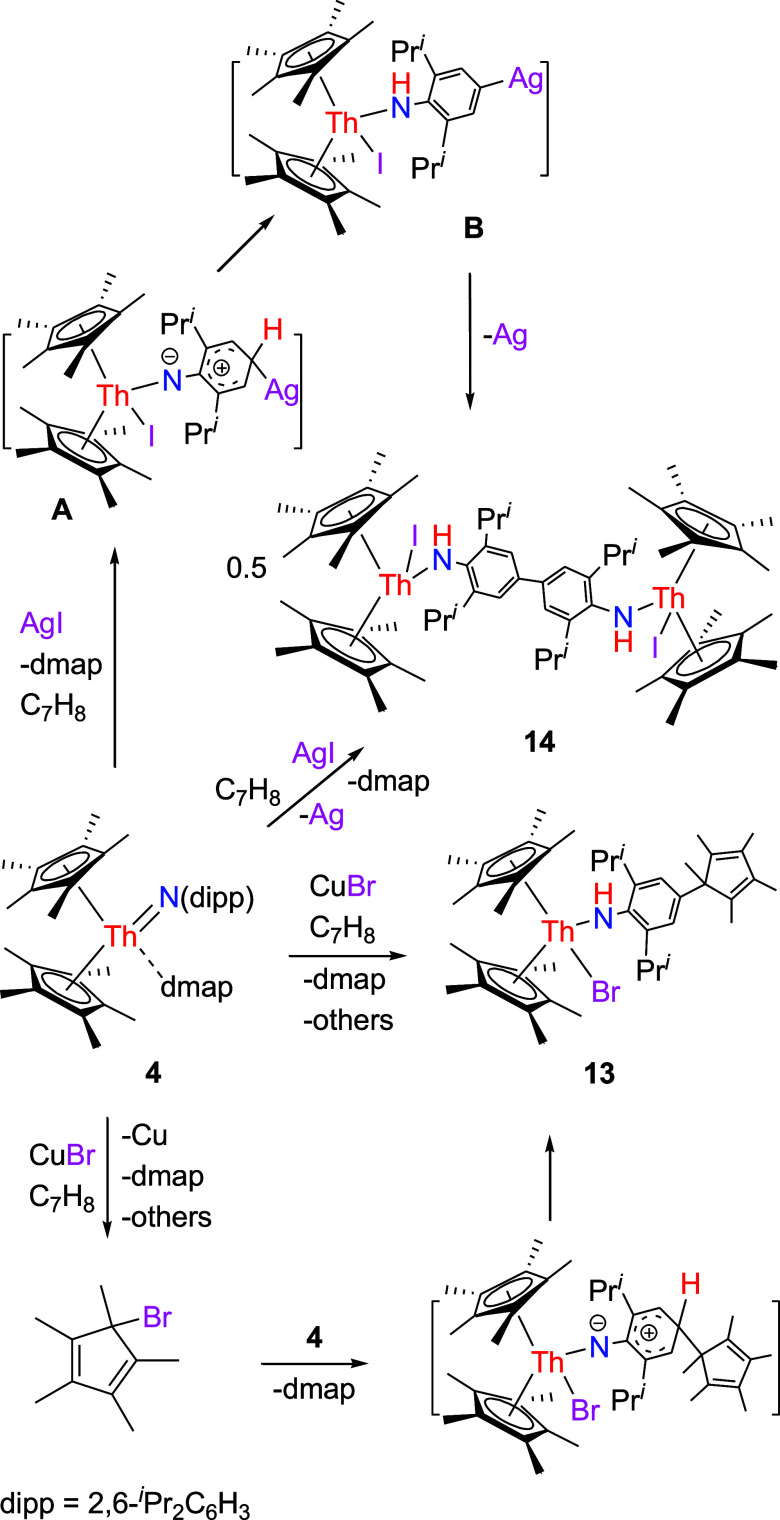
Synthesis of Compounds **13** and **14**

**Figure 11:**
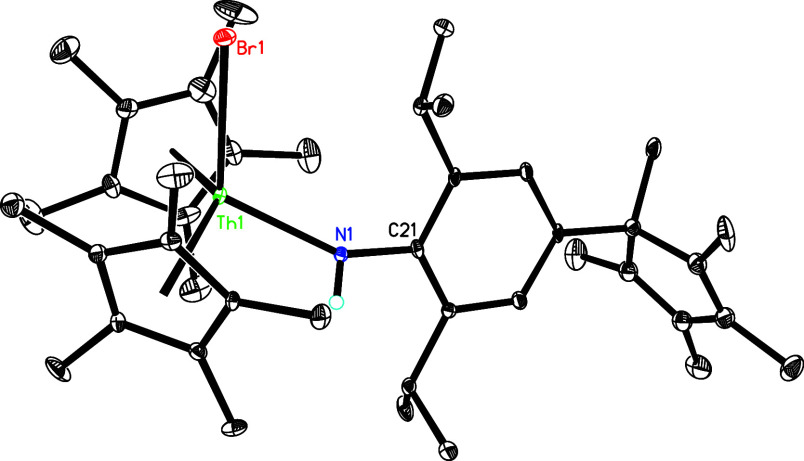
Molecular structure of **13** (thermal ellipsoids drawn at the 35% probability level).

**Figure 12:**
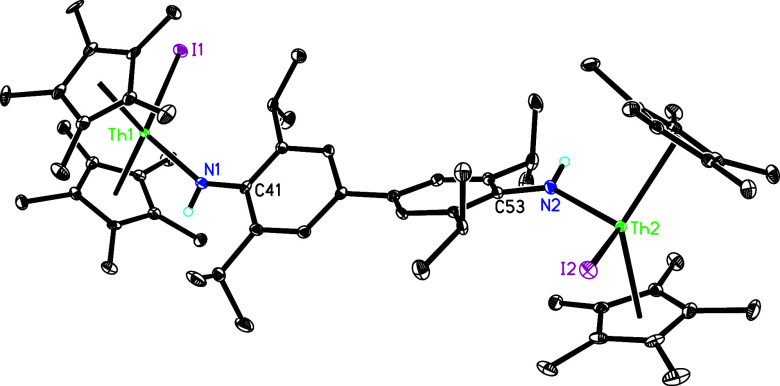
Molecular structure of **14** (thermal ellipsoids drawn at the 35% probability level).

Furthermore, treatment of **4** with elemental sulfur (S_8_) leads to clean formation of the six-membered metallaheterocycle (η^5^-C_5_Me_5_)_2_Th­[SSN­(dipp)­SS] (**15**) along with free dmap (Scheme 6). The product results from ring-opening of S_8_ by the terminal imido nitrogen atom, generating a Th–S–S–N linkage. The molecular structure of **15** is shown in Figure 13, and selected bond distances and angles are listed in Table 1. The Th–S(1) and Th–S(3) distances are 2.802(1) and 2.806(1) Å, respectively, and the S(1)–Th–S(3) angle is 112.7(1)°. Nevertheless, imido [η^5^-1,2,4-(Me_3_C)_3_C_5_H_2_]_2_Th=N­(*p*-tolyl) forms with S_8_ the four-membered complex [η^5^-1,2,4-(Me_3_C)_3_C_5_H_2_]_2_Th­[N­(*p*-tolyl)­SS] (Figure S4),^7b,9^ and (η^5^-C_5_Me_5_)_2_Th=N­(*p*-tolyl)­(dmap)_2_ results in the six-membered amino–sulfido complex (η^5^-C_5_Me_5_)_2_Th­[N­(*p*-tolyl)­S_4_] (Figure S1),^8c,9^ whereas [η^5^-1,3-(Me_3_C)_2_C_5_H_3_]_2_Th=N­(dipp)­(dmap) (Figure S3) shows similar reactivity to **4**.^8b,9^ While the reaction outcome is dictated by the steric bulk around the Th atom, all thorium imido complexes are capable of ring-opening reactions with S_8_ and form well-defined metallaheterocycles. However, unlike [η^5^-1,3-(Me_3_C)_2_C_5_H_3_]_2_Th=N­(dipp)­(dmap) (Figure S3),^8b,9^ no reaction is observed for complex **4** and Ph_2_E_2_ (E = S, Se) even when the reaction mixture is heated at 100 °C for 1 week, presumably due to the increased steric hindrance imposed by the C_5_Me_5_ ligand.

**Scheme 6:**
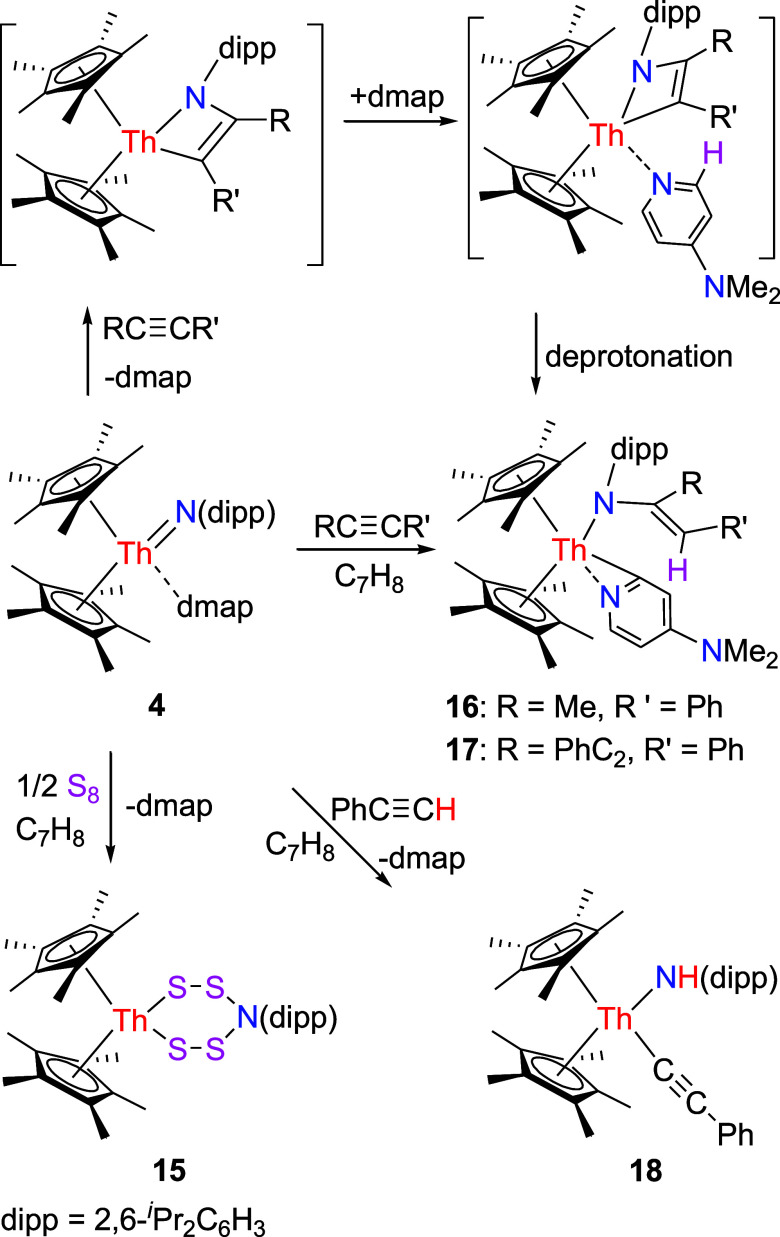
Synthesis of Compounds **15–18**

**Figure 13:**
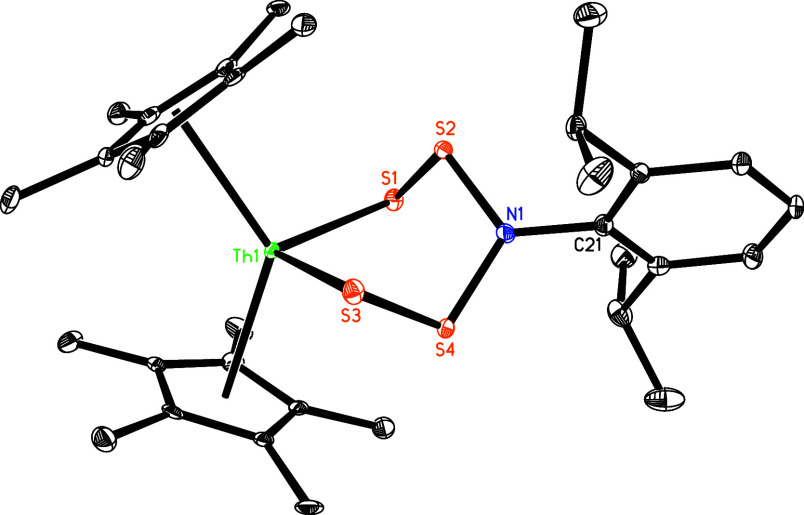
Molecular structure of **15** (thermal ellipsoids drawn at the 35% probability level).

### Reaction with Alkynes

The reactivity of the complex **4** toward alkynes RC≡CR′ was examined in comparison to those of the previously reported (η^5^-C_5_Me_5_)_2_Th=N­(*p*-tolyl)­(dmap)_2_ (Figure S1),^8c,9^ (η^5^-C_5_Me_5_)_2_Th=N­(mesityl)­(dmap) (Figure S2),^7g,9^ [η^5^-1,3-(Me_3_C)_2_C_5_H_3_]_2_Th=N­(dipp)­(dmap) (Figure S3),^8b,9^ [η^5^-1,2,4-(Me_3_C)_3_C_5_H_2_]_2_Th=N­(*p*-tolyl) and [η^5^-1,2,4-(Me_3_Si)_3_C_5_H_2_]_2_Th=N­(*p*-tolyl)­(bipy) (Figures S4 and S5).^7b,8a,9^ For example, like (η^5^-C_5_Me_5_)_2_Th=N­(*p*-tolyl)­(dmap)_2_ and (η^5^-C_5_Me_5_)_2_Th=N­(mesityl)­(dmap) (Figures S1 and S2),^7g,8c,9^ but unlike the reaction of the thorium imido complex [η^5^-1,2,4-(Me_3_C)_3_C_5_H_2_]_2_Th=N­(*p*-tolyl) with internal alkynes,^7b,9^ the [2 + 2] cycloaddition products are not isolated from the reaction of imido complex **4** with 1 equiv of PhC≡CMe or PhC≡C–C≡CPh; instead, the amido pyridyl compounds (η^5^-C_5_Me_5_)_2_Th­[N­(dipp)­C­(Me)=CHPh]­(κ^2^-*C*,*N*-4-Me_2_NC_5_H_3_N) (**16**) and (η^5^-C_5_Me_5_)_2_Th­[N­(dipp)­C­(C_2_Ph)=CHPh]­(κ^2^-*C*,*N*-4-Me_2_NC_5_H_3_N) (**17**) are formed, respectively (Scheme 6). A plausible pathway involves initial [2 + 2] cycloaddition of the Th=NR fragment of **4** with the C≡C bond in PhC≡CMe or PhC≡C–C≡CPh, followed by α-H abstraction from the coordinated dmap ligand to give the observed enamido products **16** and **17**, respectively (Scheme 6). In principle, two isomers can be formed in this transformation, but in both cases only one isomer, **16** or **17**, is detected by ^1^H NMR spectroscopy. This selectivity is rationalized by steric repulsion between the bulky 2,6-^*i*^Pr_2_C_6_H_3_ substituent on the N atom and the incoming alkyne, which forces the alkyne to approach the imido nitrogen from the less encumbered side and locks the geometry into a single product. The molecular structures of **16** and **17** are shown in Figures 14 and 15, and selected bond distances and angles are listed in Table 1. The Th–N distances for **16** (2.473(4) and 2.441(4) Å) and **17** (2.448(4) and 2.428(4) Å) are in a similar range, whereas the Th–C(42) distance of 2.457(5) Å for **16** is comparable to that found in **17** (Th–C(49): 2.447(5) Å). However, for the less-hindered imido [η^5^-1,3-(Me_3_C)_2_C_5_H_3_]_2_Th=N­(dipp)­(dmap) a pyridyl alkenyl complex [η^5^-1,3-(Me_3_C)_2_C_5_H_3_]_2_Th­[(1-(2,6-^*i*^Pr_2_C_6_H_3_)-2,5-Ph_2_C_4_HN]­(κ^2^-*C*,*N*-4-Me_2_NC_5_H_3_N) is formed in the reaction with PhC≡C–C≡CPh (Figure S3).^8b,9^

**Figure 14:**
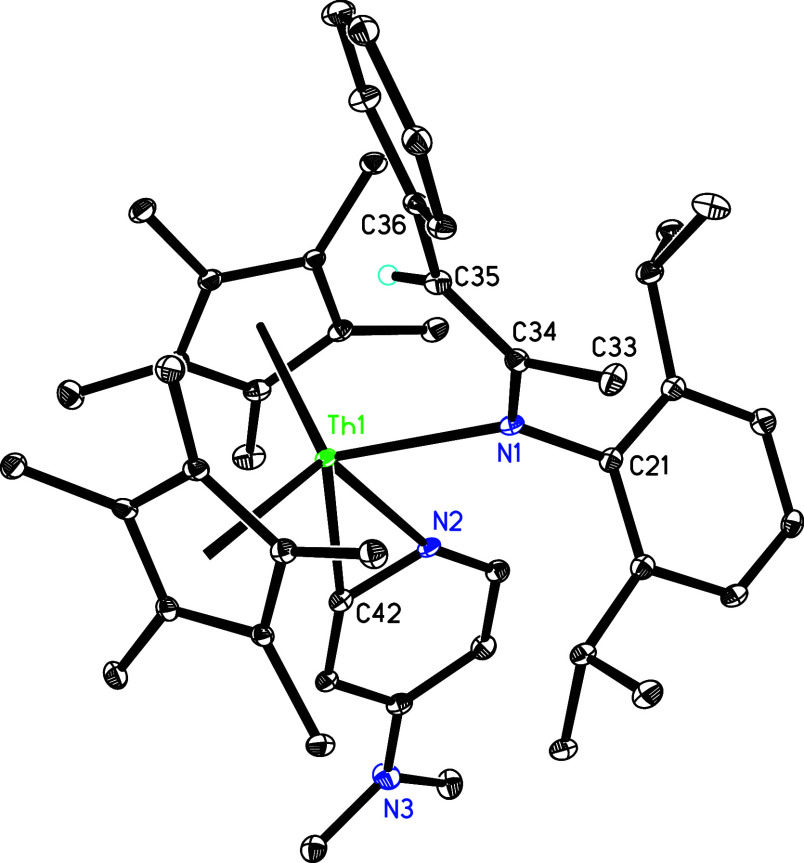
Molecular structure of **16** (thermal ellipsoids drawn at the 35% probability level).

**Figure 15:**
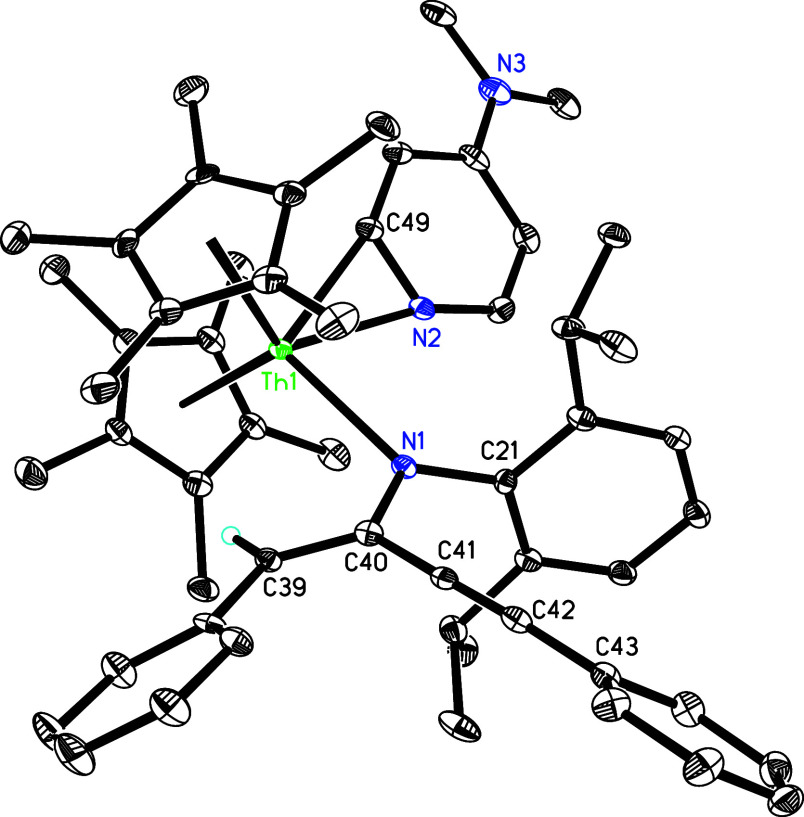
Molecular structure of **17** (thermal ellipsoids drawn at the 35% probability level).

Nevertheless, when **4** is exposed to a terminal alkyne PhC≡CH the alkynyl amido complex (η^5^-C_5_Me_5_)_2_Th­(NHdipp)­(C≡CPh) (**18**) is formed in quantitative conversion with dmap release (Scheme 6). The molecular structure of **18** is shown in Figure 16 (for selected bond distances and angles, see Table 1). The Th–C(33) distance is 2.469(7) Å, whereas the Th–N(1) distance is 2.303(5) Å. The same alkyne reacts with the bulkier Th imido compound [η^5^-1,2,4-(Me_3_Si)_3_C_5_H_2_]_2_Th=N­(*p*-tolyl)­(bipy) to give the μ-imido-bridged dimer {[η^5^-1,2,4-(Me_3_Si)_3_C_5_H_2_]­Th­(C_2_Ph)­(bipy)}_2_[μ-N­(*p*-tolyl)]_2_ (Figure S5).^8a,8b,9^ In contrast the bulk-reduced imido [η^5^-1,3-(Me_3_C)_2_C_5_H_3_]_2_Th=N­(dipp)­(dmap), however, leads to a bis–alkynyl complex [η^5^-1,3-(Me_3_C)_2_C_5_H_3_]_2_Th­(C≡CPh)_2_(dmap) (Figure S3).^8c,9^ Again, steric congestion dictates whether a single alkynyl fragment or two can be accommodated.

**Figure 16:**
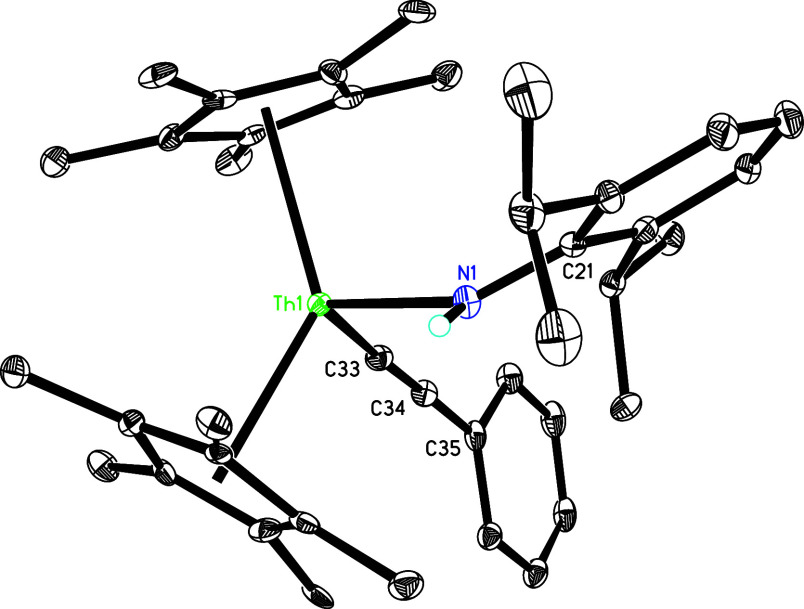
Molecular structure of **18** (thermal ellipsoids drawn at the 35% probability level).

Despite the facile reactions described above, **4** shows no observable reaction with the sterically demanding internal alkyne PhC≡CPh, even after heating at 100 °C for 1 week. This lack in reactivity mirrors that of (η^5^-C_5_Me_5_)_2_Th=N­(mesityl)­(dmap) and [η^5^-1,3-(Me_3_C)_2_C_5_H_3_]_2_Th=N­(dipp)­(dmap),^7g,8b,9^ and is attributed to the increased steric hindrance of the 2,6-^*i*^Pr_2_C_6_H_3_ substituent. However, it should be noted that the thorium imidos (η^5^-C_5_Me_5_)_2_Th=N­(*p*-tolyl)­(dmap)_2_ (Figure S1),^8c,9^ [η^5^-1,2,4-(Me_3_C)_3_C_5_H_2_]_2_Th=N­(*p*-tolyl) and [η^5^-1,2,4-(Me_3_Si)_3_C_5_H_2_]_2_Th=N­(*p*-tolyl)­(bipy) (Figures S4 and S5)^7b,8a,9^ do react with PhC≡CPh.

### Reaction with Carbodiimides, Aldehydes, Ketones, CS_2_, Amidates, and Imines

Thorium-imido complexes are well-known to react with polar C-E bonds of heterounsaturated organic substrates. The behavior of the thorium imido complex **4** is directly comparable to that of the previously reported derivatives (η^5^-C_5_Me_5_)_2_Th=N­(*p*-tolyl)­(dmap)_2_ (Figure S1),^8c,9^ (η^5^-C_5_Me_5_)_2_Th=N­(mesityl)­(dmap) (Figure S2),^7g,9^ [η^5^-1,3-(Me_3_C)_2_C_5_H_3_]_2_Th=N­(dipp)­(dmap) (Figure S3),^8b,9^ [η^5^-1,2,4-(Me_3_C)_3_C_5_H_2_]_2_Th=N­(*p*-tolyl) and [η^5^-1,2,4-(Me_3_Si)_3_C_5_H_2_]_2_Th=N­(*p*-tolyl)­(bipy) (Figures S4 and S5).^7b,8a,9^ In each case the steric environment of the imido-aryl group together with the steric and electronic nature of the substrates dictates whether a [2 + 2] cycloaddition, a nucleophilic addition, or a deprotonation pathway is accessed.

When **4** is treated with the symmetric carbodiimide (^*i*^PrN=)_2_C, a clean [2 + 2] cycloaddition occurs to give the complex (η^5^-C_5_Me_5_)_2_Th­[N­(dipp)­C­(=N^*i*^Pr)­N­(^*i*^Pr)]­(dmap) (**19**) in good yield (Scheme 7). The molecular structure of **19** is shown in Figure 17, and selected bond distances and angles are listed in Table 1. The Th–N(1) and Th–N(3) distances are 2.425(16) and 2.363(17) Å, respectively, whereas the Th–N(4) distance of 2.655(8) Å is best described as a datively coordinated nitrogen atom. This [2 + 2] cycloaddition reactivity is closely related to that observed for (η^5^-C_5_Me_5_)_2_Th=N­(*p*-tolyl)­(dmap)_2_ and (η^5^-C_5_Me_5_)_2_Th=N­(mesityl)­(dmap) (Figures S1 and S2).^7g,8c,9^

**Scheme 7:**
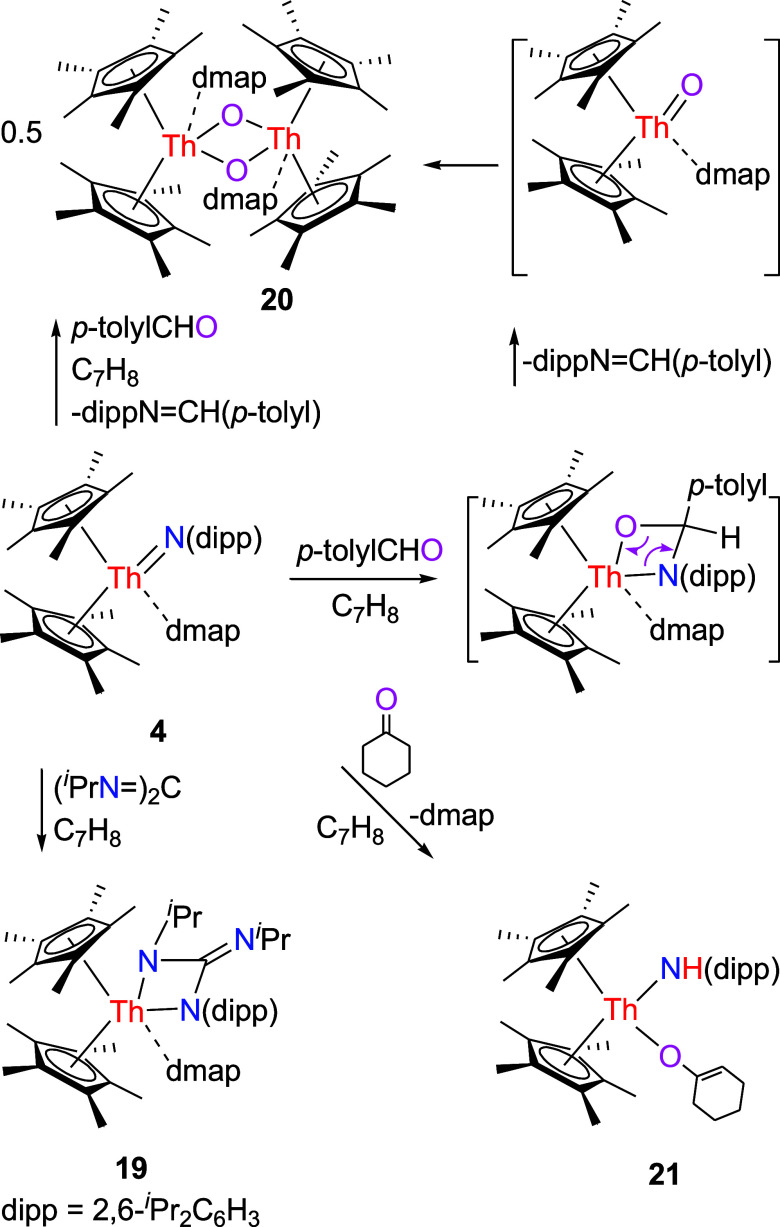
Synthesis of Compounds **19–21**

**Figure 17:**
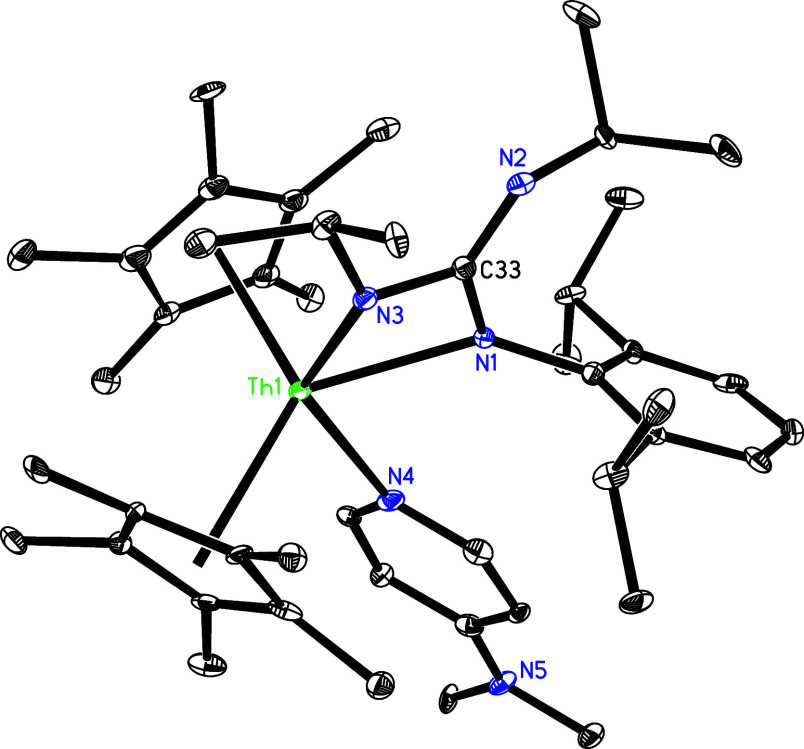
Molecular structure of **19** (thermal ellipsoids drawn at the 35% probability level).

Nevertheless, **4** reacts with an aromatic aldehyde *p*-tolylCHO to give the μ-oxido dimer [(η^5^-C_5_Me_5_)_2_Th­(dmap)]_2_(μ-O)_2_ (**20**) in good yield (Scheme 7). This reactivity, however, is distinctively different from that observed for [η^5^-1,3-(Me_3_C)_2_C_5_H_3_]_2_Th=N­(dipp)­(dmap) and *p*-tolylCHO, which yields the alkoxido-amidate [η^5^-1,3-(Me_3_C)_2_C_5_H_3_]_2_Th­[OCH_2_(*p*-MePh)]­[OC­(*p*-MePh)­N­(dipp)] (Figure S3),^8b,9^ because of the reduced steric hindrance of the 1,3-(Me_3_C)_2_C_5_H_3_ ligand. Based on the reaction of the thorium imido (η^5^-C_5_Me_5_)_2_Th=N­(*p*-tolyl)­(dmap)_2_ with Ph_2_CO (Figure S1),^8c,9^ it may be suggested that complex **4** undergoes an initial [2 + 2] cycloaddition with *p*-tolylCHO to form an unstable four-membered metallaheterocyclic intermediate, which then converts to the thorium oxido (η^5^-C_5_Me_5_)_2_ThO­(dmap) by elimination of (dipp)­N=CH­(*p*-tolyl). However, unlike the more sterically encumbered actinide oxido derivatives [η^5^-1,2,4-(Me_3_C)_3_C_5_H_2_]_2_AnO­(dmap) (An = Th, U),^3m,7a^ the Cp* ligand lacks sufficient steric demand to stabilize the monomeric oxido intermediate (η^5^-C_5_Me_5_)_2_ThO­(dmap), which immediately dimerizes to the final product **20** (Scheme 7).

Yet, treatment of **4** with cyclohexanone affords the amido enolyl complex (η^5^-C_5_Me_5_)_2_Th­[NH­(dipp)]­(κ-*O*-1-OC_6_H_9_) (**21**) with dmap release, in which an α-H of cyclohexanone is transferred to the imido Th=Ndipp moiety (Scheme 7). The molecular structure of **21** is shown in Figure 18, and selected bond distances and angles are listed in Table 1. The Th–N(1) distance is 2.335(6) Å, whereas the Th–O(1) distance is 2.174(5) Å. This reactivity closely resembles that of [η^5^-1,3-(Me_3_C)_2_C_5_H_3_]_2_Th=N­(dipp)­(dmap) with cyclohexanone (Figure S3).^8b,9^ However, **4** shows no reaction toward Ph_2_CO or Ph_2_CS even when the reaction mixture is heated at 100 °C for 1 week, which contrasts the reactivity of other thorium imidos (η^5^-C_5_Me_5_)_2_Th=N­(*p*-tolyl)­(dmap)_2_ (Figure S1),^8c,9^ (η^5^-C_5_Me_5_)_2_Th=N­(mesityl)­(dmap) (Figure S2),^7f,7g,9^ [η^5^-1,3-(Me_3_C)_2_C_5_H_3_]_2_Th=N­(dipp)­(dmap) (Figure S3),^8b,9^ [η^5^-1,2,4-(Me_3_C)_3_C_5_H_2_]_2_Th=N­(*p*-tolyl) (Figure S4),^7a,9^ and [η^5^-1,2,4-(Me_3_Si)_3_C_5_H_2_]_2_Th=N­(*p*-tolyl)­(bipy) (Figure S5).^8a,9^ We ascribe this difference again to the steric shielding of the Th atom in **4**.

**Figure 18:**
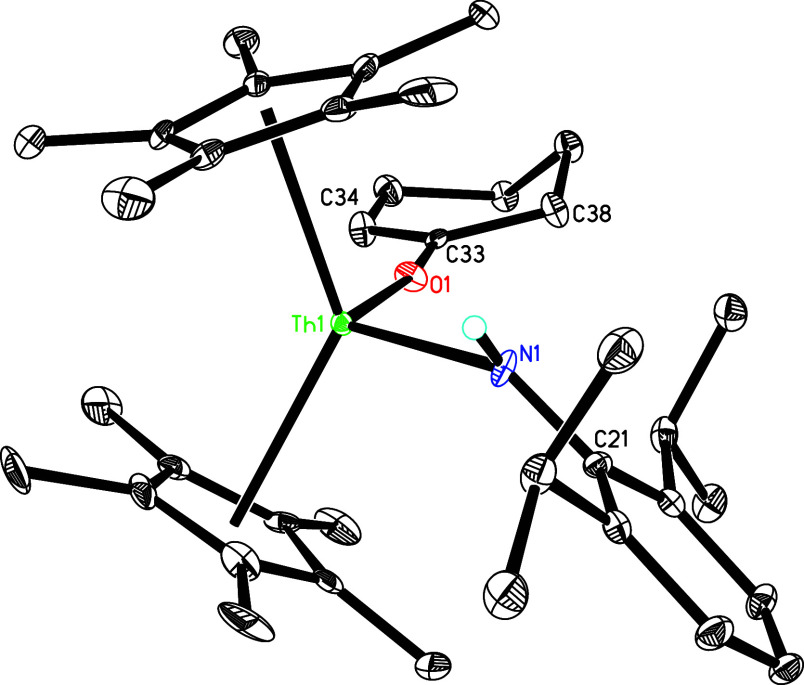
Molecular structure of **21** (thermal ellipsoids drawn at the 35% probability level).

Nevertheless, exposure of **4** to CS_2_ furnishes the four-membered metallaheterocycle (η^5^-C_5_Me_5_)_2_Th­[SC=N­(dipp)-S]­(dmap) (**22**) (Scheme 8), which resembles the reactivity observed for (η^5^-C_5_Me_5_)_2_Th=N­(mesityl)­(dmap) (Figure S2).^7g,9^ The formation of **22** may be explained by an initial [2 + 2] cycloaddition of **4** with CS_2_, followed by a [1,3]-Th migration (Scheme 8). The molecular structure of **22** is shown in Figure 19, and selected bond distances and angles are listed in Table 1. The relatively long Th–N(2) distance of 2.631(7) Å is indicative of a datively coordinated nitrogen atom, whereas the Th–S(1) and Th–S(2) distances are 2.822(4) and 2.806(4) Å, respectively. Nevertheless, this reactivity pattern is distinctively different to those observed for (η^5^-C_5_Me_5_)_2_Th=N­(*p*-tolyl)­(dmap)_2_ (Figure S1),^8c,9^ [η^5^-1,3-(Me_3_C)_2_C_5_H_3_]_2_Th=N­(dipp)­(dmap), and [η^5^-1,2,4-(Me_3_C)_3_C_5_H_2_]_2_Th=N­(*p*-tolyl) (Figures S3 and S4).^7a,8b,9^ The first two imido complexes form dimeric products with [(η^5^-C_5_Me_5_)_2_Th]_2_{μ-[N­(*p*-tolyl)­C­(S)­S]}_2_ (Figure S1)^8c,9^ and {[η^5^-1,3-(Me_3_C)_2_C_5_H_3_]_2_Th}_2_(μ-S)_2_ (Figure S3),^8b,9^ respectively, whereas the more bulky derivative [η^5^-1,2,4-(Me_3_C)_3_C_5_H_2_]_2_Th=N­(*p*-tolyl) yields the four-membered metallaheterocycles [η^5^-1,2,4-(Me_3_C)_3_C_5_H_2_]_2_Th­[N­(*p*-tolyl)­C­(S)–S] (Figure S4).^7a,9^ Again, this difference is ascribed to the steric environment of the Th atom.

**Scheme 8:**
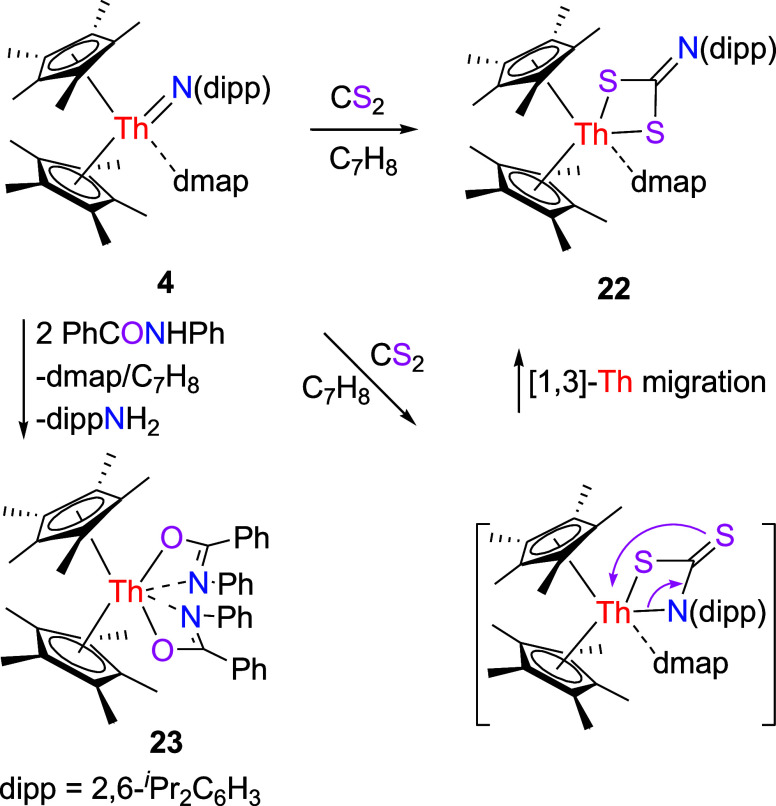
Synthesis of Compounds **22** and **23**

**Figure 19:**
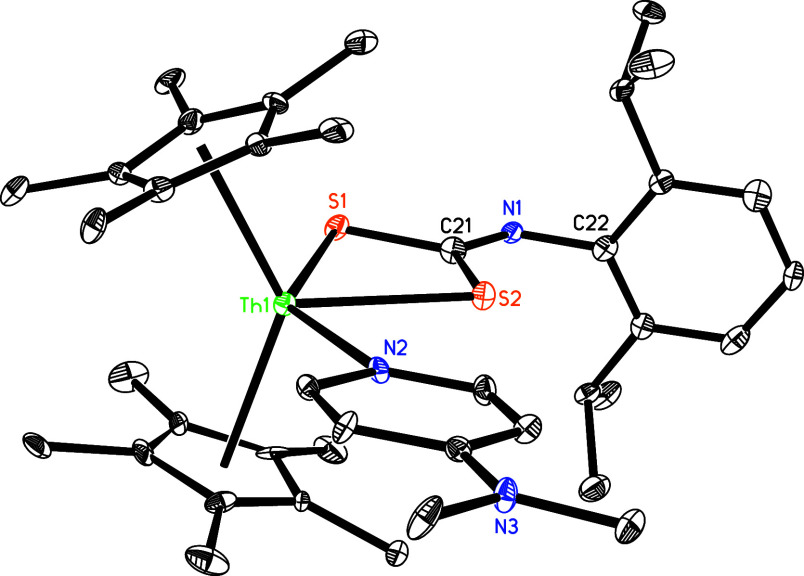
Molecular structure of **22** (thermal ellipsoids drawn at the 35% probability level).

However, when **4** is combined with 2 equiv of the amidate PhCONHPh the bis-amidate (η^5^-C_5_Me_5_)_2_Th­[OC­(Ph)­N­(Ph)]_2_ (**23**) is formed in quantitative conversion besides free dmap and dippNH_2_ (Scheme 8). In this reaction the Th=N­(dipp) moiety serves as a strong base, and this agrees with the reactivity of (η^5^-C_5_Me_5_)_2_Th=N­(*p*-tolyl)­(dmap)_2_ and [η^5^-1,3-(Me_3_C)_2_C_5_H_3_]_2_Th=N­(dipp)­(dmap) (Figures S1 and S3).^8b,8c,9^ The molecular structure of **23** (Figure 20 and Table 1) features two essentially identical Th–O(1) and Th–O(2) distances of 2.498(9) Å and 2.493(10) Å, respectively, the same holds true for the Th–N(1) and Th–N(2) distances of 2.582(12) Å and 2.580(13) Å, respectively. Moreover, the pronounced difference in the Th–O vs. Th–N bond distances suggests that the negative charge is mainly located on the O atoms of the amidate fragment.

**Figure 20:**
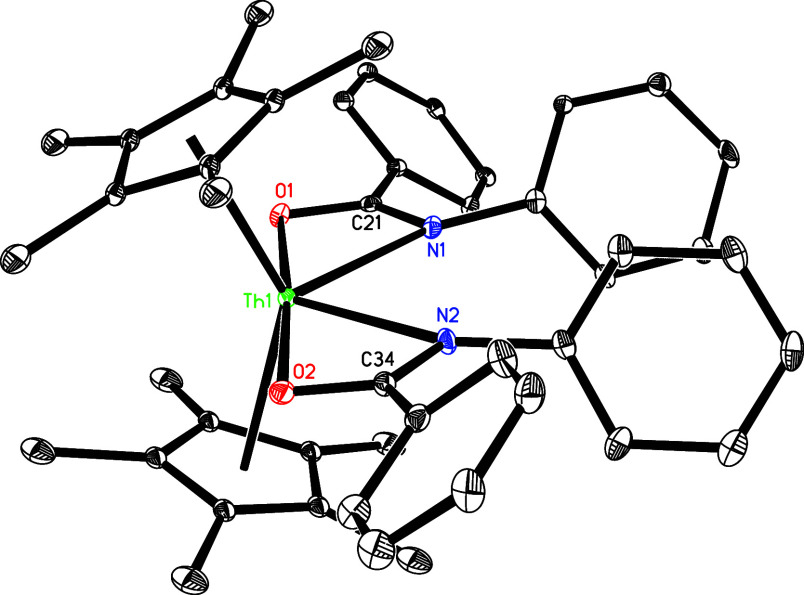
Molecular structure of **23** (thermal ellipsoids drawn at the 35% probability level).

Moreover, **4** may also react with imines R_2_C=NH similar to [η^5^-1,3-(Me_3_C)_2_C_5_H_3_]_2_Th=N­(dipp)­(dmap) and [η^5^-1,2,4-(Me_3_Si)_3_C_5_H_2_]_2_Th=N­(*p*-tolyl)­(bipy) (Figures S3 and S5).^8a,8c,9^ When **4** is exposed to Ph_2_C=NH the bis–iminato complex (η^5^-C_5_Me_5_)_2_Th­(N=CPh_2_)_2_ (**24**) is detected in quantitative conversion besides free dmap and dippNH_2_ (Scheme 9). The ORTEP diagram of **24** is shown in Figure 21, while Table 1 lists selected bond distances and angles. The Th–N(1) and Th–N(2) distances are 2.267(3) and 2.280(3) Å, respectively, and the N(1)–Th–N(2) angle is 110.6(1)°. This reactivity is, however, contrary to that of [η^5^-1,3-(Me_3_C)_2_C_5_H_3_]_2_Th=N­(dipp)­(dmap) forming with (*p*-tolyl)_2_C=NH the amido–iminato complex [η^5^-1,3-(Me_3_C)_2_C_5_H_3_]_2_Th­[NH­(dipp)]­[N=C­(*p*-tolyl)_2_] (Figure S3),^8c,9^ again, presumably due to the reduced steric hindrance of the 1,3-(Me_3_C)_2_C_5_H_3_ ligand.

**Scheme 9:**
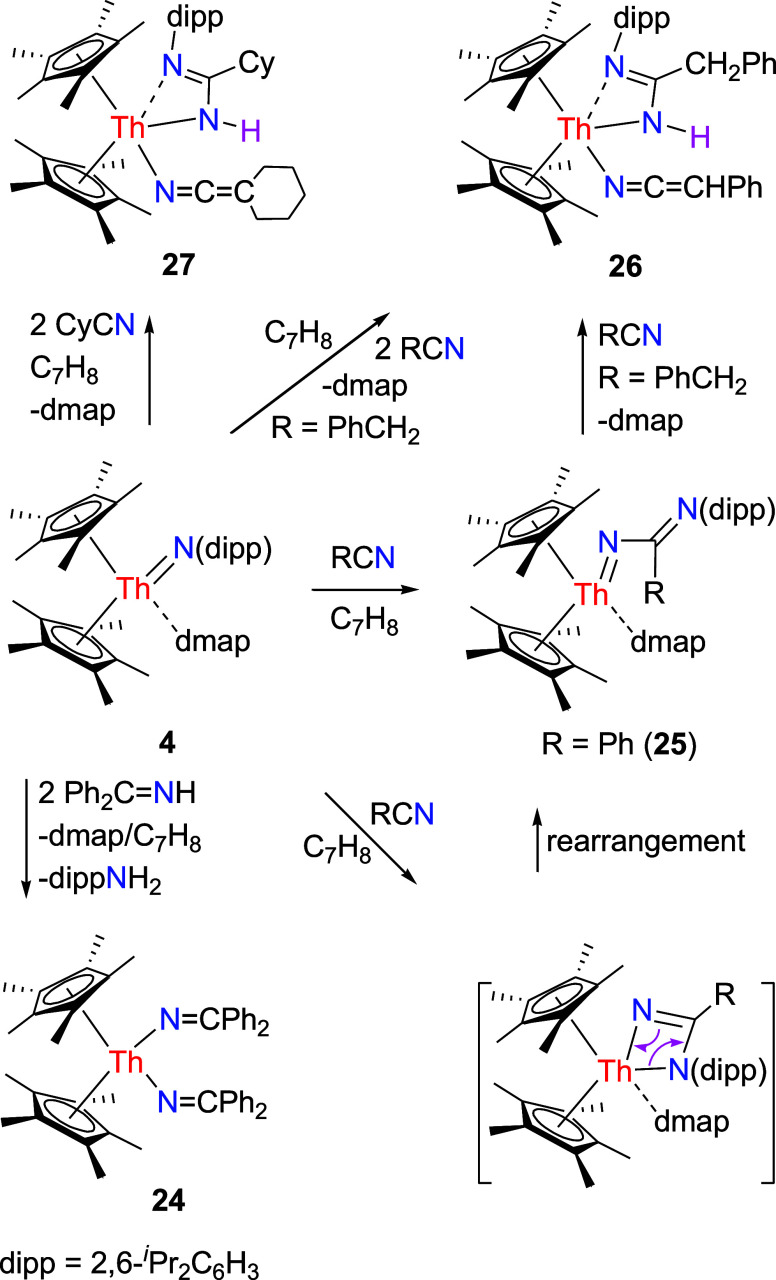
Synthesis of Compounds **24–27**

**Figure 21:**
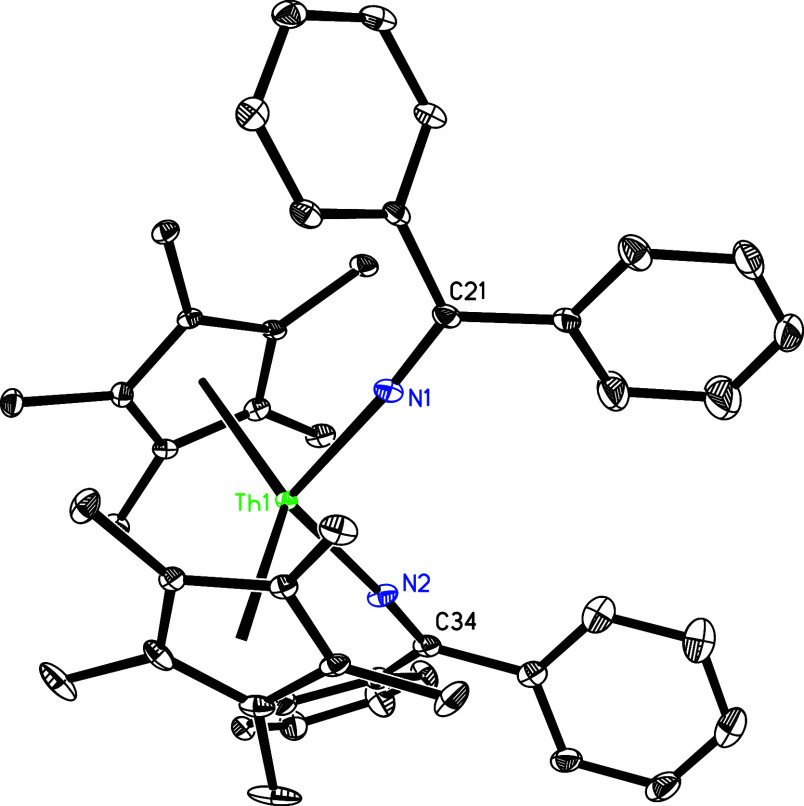
Molecular structure of **24** (thermal ellipsoids drawn at the 35% probability level).

### Reaction with Organic Nitriles and Isonitriles

Complex **4** exhibits reactivity toward organic nitriles analogous to the previously reported imido compounds (η^5^-C_5_Me_5_)_2_Th=N­(*p*-tolyl)­(dmap)_2_ (Figure S1),^8c,9^ (η^5^-C_5_Me_5_)_2_Th=N­(mesityl)­(dmap) (Figure S2),^7g,9^ [η^5^-1,3-(Me_3_C)_2_C_5_H_3_]_2_Th=N­(dipp)­(dmap) (Figure S3),^8b,9^ [η^5^-1,2,4-(Me_3_C)_3_C_5_H_2_]_2_Th=N­(*p*-tolyl) (Figure S4),^7b,9^ and [η^5^-1,2,4-(Me_3_Si)_3_C_5_H_2_]_2_Th=N­(*p*-tolyl)­(bipy) (Figure S5).^8a,9^ For example, upon treatment with PhCN, **4** yields the dmap imido adduct (η^5^-C_5_Me_5_)_2_Th­[=NC­(Ph)=N­(dipp)]­(dmap) (**25**) in good yield (Scheme 9). This behavior diverges from other imido systems which form the amidinyl pyridyl complex (η^5^-C_5_Me_5_)_2_Th­[η^3^-NHC­(Ph)­N­(*p*-tolyl)]­[κ^2^-*C*,*N*-4-(Me_2_N)­C_5_H_3_N] (Figure S1),^8c,9^ the amido pyridyl complex [η^5^-1,3-(Me_3_C)_2_C_5_H_3_]_2_Th­[NHC­(Ph)=Ndipp]­[κ^2^-*C*,*N*-4-(Me_2_N)­C_5_H_3_N] (Figure S3),^8b,9^ and the [2 + 2] cycloaddition product [η^5^-1,2,4-(Me_3_C)_3_C_5_H_2_]_2_Th­[N­(*p*-tolyl)­C­(Ph)=N] (Figure S4),^7b,9^ respectively. However, **4** behaves similarly to (η^5^-C_5_Me_5_)_2_Th=N­(mesityl)­(dmap) (Figure S2),^7g,9^ suggesting that sterics at the thorium atom dictate the reaction pathway. A proposed mechanism for the formation of **25** involves initial formation of a four-membered intermediate. For steric relief this intermediate rapidly rearranges to the imido product **25** (Scheme 9). The molecular structure of **25** is shown in Figure 22, and selected bond distances and angles are listed in Table 1. The relatively long Th–N(3) distance of 2.560(4) Å indicates a datively coordinated nitrogen atom, whereas the short Th–N(1) distance of 2.117(4) Å aligns with a Th=N double bond character.^7b^

**Figure 22:**
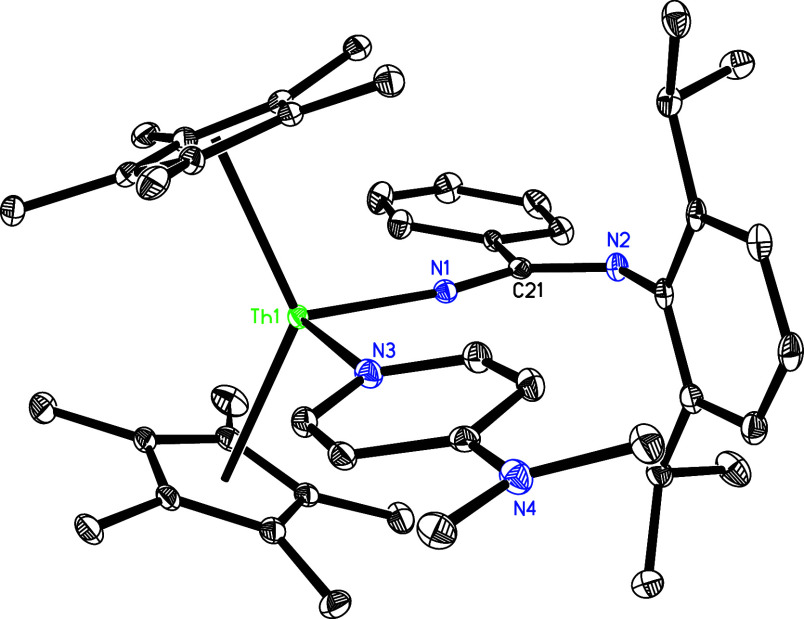
Molecular structure of **25** (thermal ellipsoids drawn at the 35% probability level).

Nevertheless, the thorium imido complexes (η^5^-C_5_Me_5_)_2_Th=N­(*p*-tolyl)­(dmap)_2_ (Figure S1),^8c,9^ [η^5^-1,3-(Me_3_C)_2_C_5_H_3_]_2_Th=N­(dipp)­(dmap) (Figure S3),^8b,9^ [η^5^-1,2,4-(Me_3_Si)_3_C_5_H_2_]_2_Th=N­(*p*-tolyl)­(bipy) (Figure S5),^8a,9^ and **4** exhibit identical reactivity toward benzyl nitrile PhCH_2_CN. For **4**, the reaction with PhCH_2_CN yields the iminato amido complex (η^5^-C_5_Me_5_)_2_Th­[NHC­(CH_2_Ph)=N­(dipp)]­(N=C=CHPh) (**26**) in quantitative conversion, accompanied by dmap release (Scheme 9). A proposed mechanism involves initial formation of a dmap imido adduct, which subsequently reacts with a second PhCH_2_CN molecule. Deprotonation of the weak, benzylic C–H bond facilitated by dmap release forms the final product **26** (Scheme 9). A similar reaction with cyclohexyl nitrile C_6_H_11_CN affords the iminato amido complex (η^5^-C_5_Me_5_)_2_Th­[NHC­(C_6_H_11_)=N­(dipp)]­[N=C=C­(CH_2_)_5_] (**27**) in quantitative conversion with dmap release (Scheme 9). The molecular structure of **26** is shown in Figure 23, whereas the molecular structure of **27** is provided in the Supporting Information. In complex **26**, the Th–N(1), Th–N(2), and Th–N(3) distances are 2.608(5), 2.421(5), and 2.426(5) Å, respectively, which are comparable to those found in **27** (2.586(13), 2.418(10) and 2.335(12) Å).

**Figure 23:**
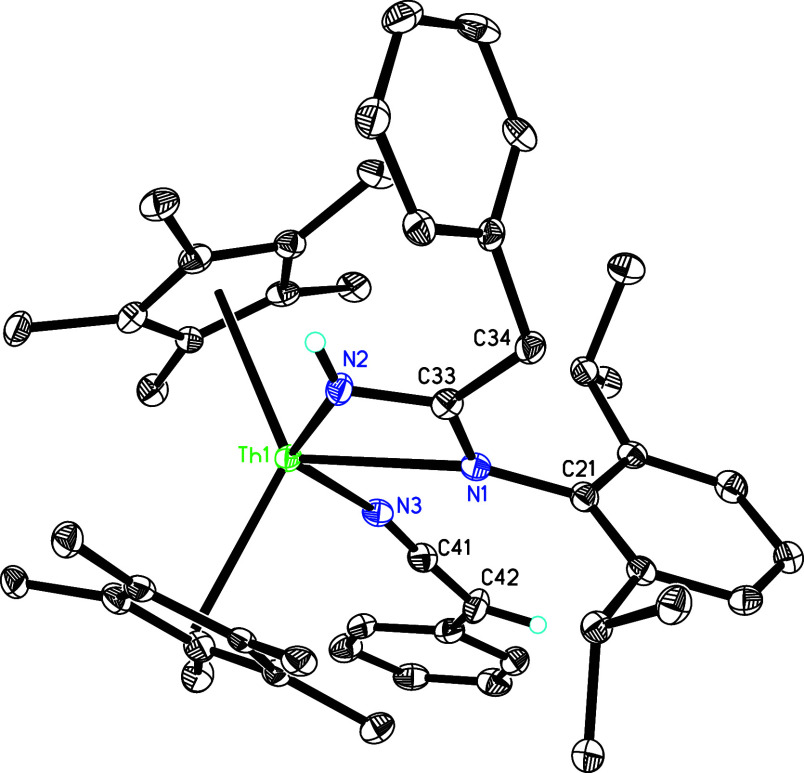
Molecular structure of **26** (thermal ellipsoids drawn at the 35% probability level).

However, the reaction of **4** with the dinitrile 1,4-(CH_2_)_4_(CN)_2_ furnishes the bis-amido complex (η^5^-C_5_Me_5_)_2_Th­{NH­[C=C­(CN)­(CH_2_)_3_]}]­{NH­[C=NC­(=Ndipp)­(CH_2_)_4_]} (**28**) in good yield concomitant with dmap release (Scheme 10). This complex represents a remarkable example of dinitrile activation, most likely proceeding via a cascade of transformations. The formation of **28** may be rationalized through a sequence of intermediates (Scheme 10): Initial deprotonation of an α–C–H-bond in 1,4-(CH_2_)_4_(CN)_2_ by **4**, releasing dmap and forming amido alkyl complex **A**. Nitrile insertion into the Th–CHCN moiety of **A** generates amido–iminato complex **B**. A [1,3]-H migration in **B** yields bis-amido complex **C**. Second dinitrile coordination via insertion into the Th–NH­(dipp) moiety forms amido–iminato complex **D**. Subsequent nitrile insertion into the Th–N=C moiety of **D**, followed by a [1,5]-H shift, affords the final product **28**. The reactivity of **4** with 1,4-(CH_2_)_4_(CN)_2_ differs significantly from that of [η^5^-1,3-(Me_3_C)_2_C_5_H_3_]_2_Th=N­(dipp)­(dmap), which gives rise to the eight-membered metallacycle [η^5^-1,3-(Me_3_C)_2_C_5_H_3_]_2_Th­[NHC=C­{(CH_2_)_3_}­C­(=NH)­NC=N­{C­(=Ndipp)­(CH_2_)_4_}] (Figure S3).^8b,9^ This divergence is attributed to the reduced steric hindrance of the 1,3-(Me_3_C)_2_C_5_H_3_ ligand. The molecular structure of **28** is shown in Figure 24, whereas selected bond distances and angles are listed in Table 1. The Th–N(3) and Th–N(4) distances are 2.437(4) and 2.394(4) Å, respectively, whereas the Th–N(2) distance is 2.591(4) Å, consistent with a datively coordinated nitrogen atom.

**Scheme 10:**
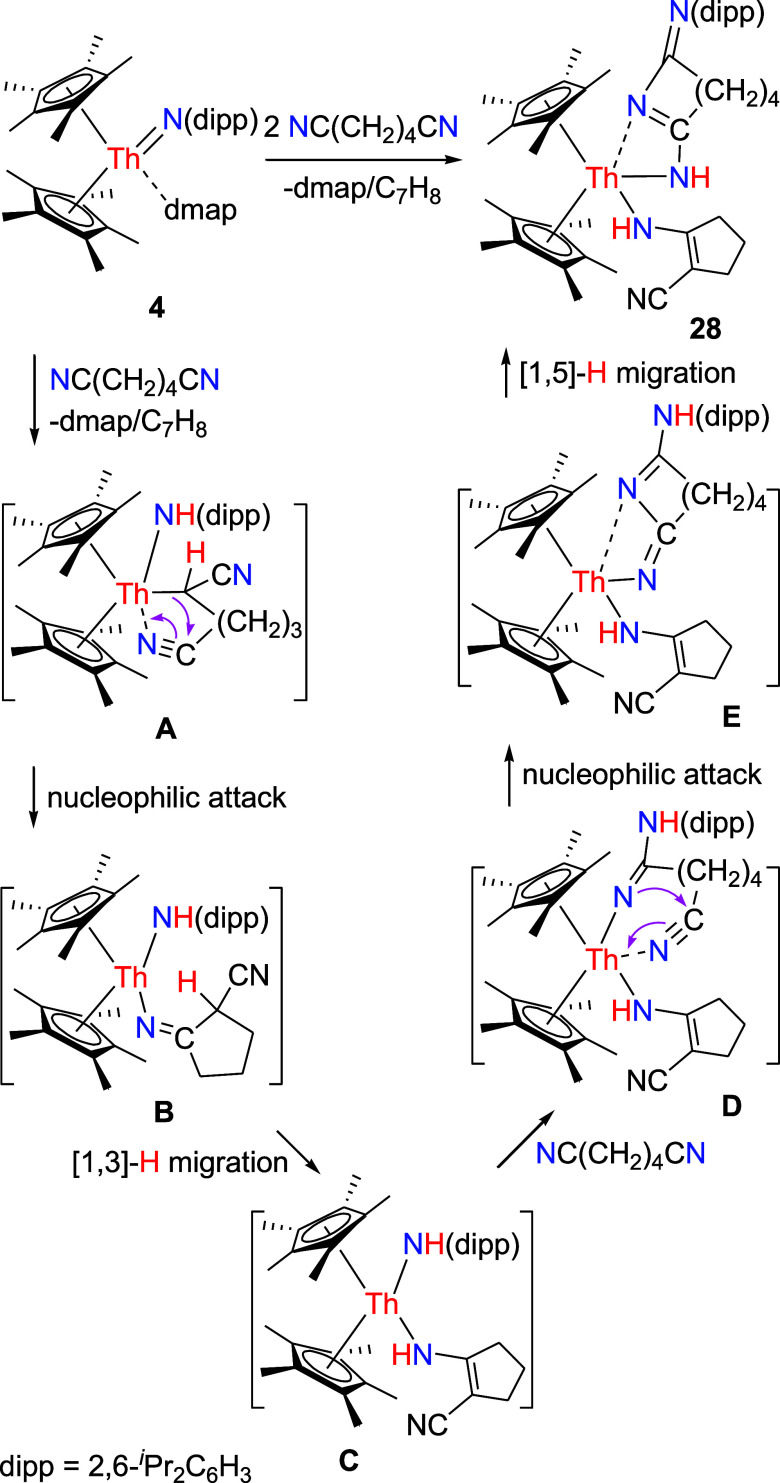
Synthesis of Compound **28**

**Figure 24:**
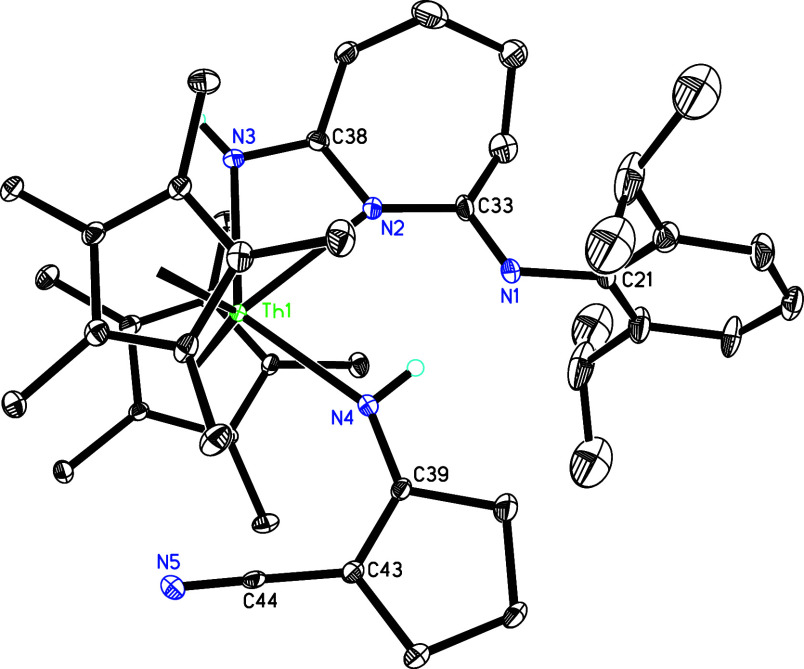
Molecular structure of **28** (thermal ellipsoids drawn at the 35% probability level).

Analogous to the thorium imido complexes (η^5^-C_5_Me_5_)_2_Th=N­(*p*-tolyl)­(dmap)_2_ (Figure S1),^8c,9^ [η^5^-1,3-(Me_3_C)_2_C_5_H_3_]_2_Th=N­(dipp)­(dmap) (Figure S3),^8b,9^ [η^5^-1,2,4-(Me_3_C)_3_C_5_H_2_]_2_Th=N­(*p*-tolyl) (Figure S4),^7h,9^ and [η^5^-1,2,4-(Me_3_Si)_3_C_5_H_2_]_2_Th=N­(*p*-tolyl)­(bipy) (Figure S5),^8a,9^ complex **4** also reacts with organic isonitriles. However, the reaction outcomes differ significantly due to ligand steric and electronic environments. Upon treatment with C_6_H_11_NC, complex **4** forms the six-membered heterocyclic complex (η^5^-C_5_Me_5_)_2_Th­[2-C­(=Ndipp)­N­(C_6_H_11_)-3-N­(C_6_H_11_)-5-(C_6_H_11_)-6,6-(CH_2_)_5_-(1,3-C_4_HN_2_)] (**29**) with dmap loss (Scheme 11). This contrasts with the other imido compounds mentioned above, which yield an amido alkenyl (η^5^-C_5_Me_5_)_2_Th­[NH­(*p*-tolyl)]­[κ^2^-*C*,*N*-2-(C_6_H_11_NC)-4-(Me_2_N)­C_5_H_3_N] (Figure S1),^8c,9^ the five-membered metallaheterocycle [η^5^-1,3-(Me_3_C)_2_C_5_H_3_]_2_Th­[N­(C_6_H_11_)­CH=C­{C­(=Ndipp)­N=C­(CH_2_)_5_}­N­(C_6_H_11_)] (Figure S3),^8b,9^ and the bis-amido [η^5^-1,2,4-(Me_3_Si)_3_C_5_H_2_]_2_Th­[1-C_6_H_11_-2,2-(CH_2_)_5_-4-(*p*-tolyl)­N-5-C_6_H_11_N-(1,3-C_4_HN_2_)] (Figure S5),^8a,9^ respectively, with C_6_H_11_NC. The distinct reactivity of **4** is attributed to the increased sterics around the metal, favoring the oligomerization of isonitriles. No intermediates were observed by NMR spectroscopy, but based on the reactivity of the thorium phosphinidene complex [η^5^-1,2,4-(Me_3_C)_3_C_5_H_2_]_2_Th = P­(2,4,6-^*t*^Bu_3_Ph) with isonitriles RNC,^7h^ the reaction is proposed to proceed via initial [2 + 1] cycloaddition with C_6_H_11_NC to give a metallaaziridine **A**, followed by a [1,3]-Th migration to yield another metallaaziridine **B**. Subsequent reactions with two additional C_6_H_11_NC molecules form a four-membered heterometallacycle and then a six-membered heterometallacycle. The reactive carbene (RR′C:) moiety in the latter then couples with a fourth molecule of isonitrile C_6_H_11_NC to afford the intermediate **E**, followed by a [1,5]-H migration to yield intermediate **F**. Finally, the intermediate **F** undergoes a [4 + 2] cycloaddition reaction to form **29** (Scheme 11). The molecular structure of **29** (Figure 25) features the Th–N(1) and Th–N(5) distances of 2.395(3) and 2.257(3) Å, respectively, and the N(1)–Th–N(5) angle of 85.6(1)° (Table 1).

**Scheme 11:**
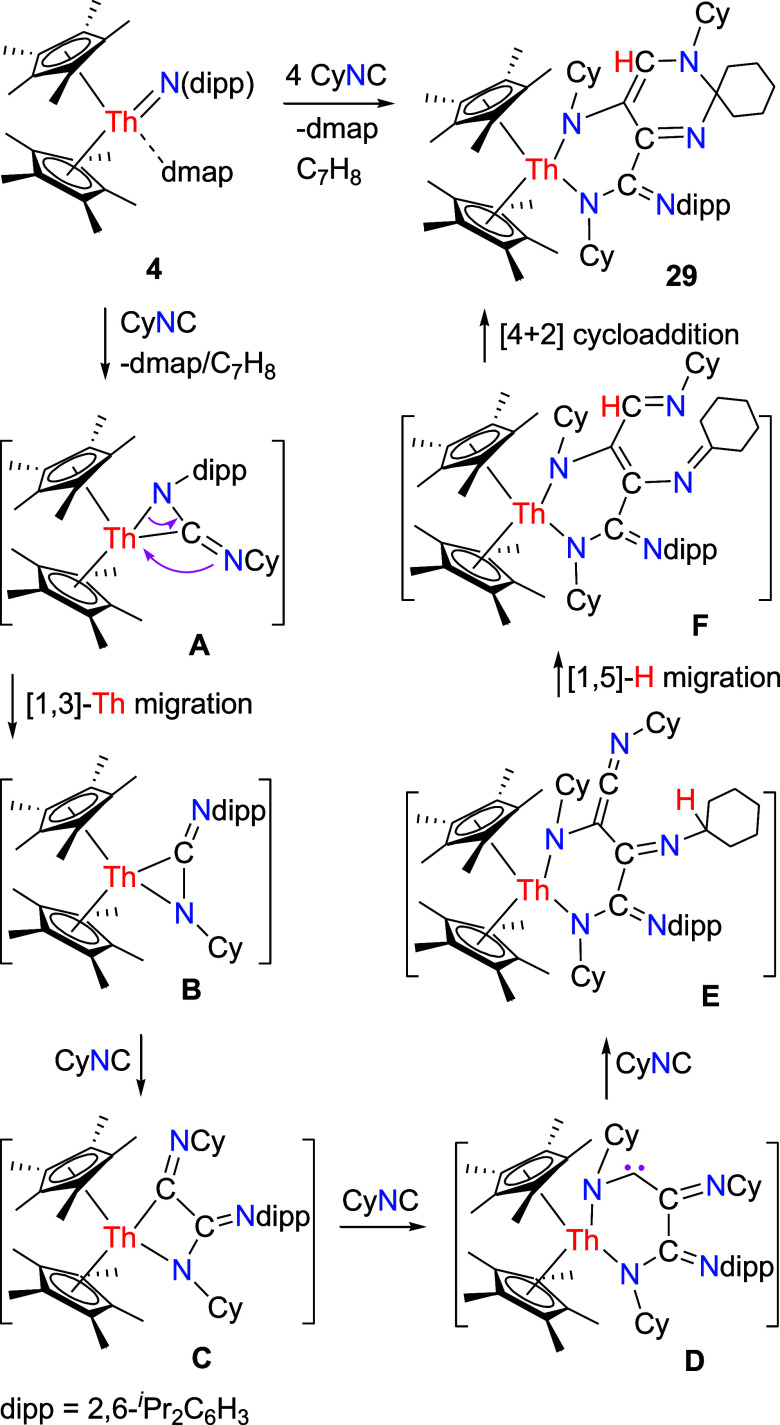
Synthesis of Compound **29**

**Figure 25:**
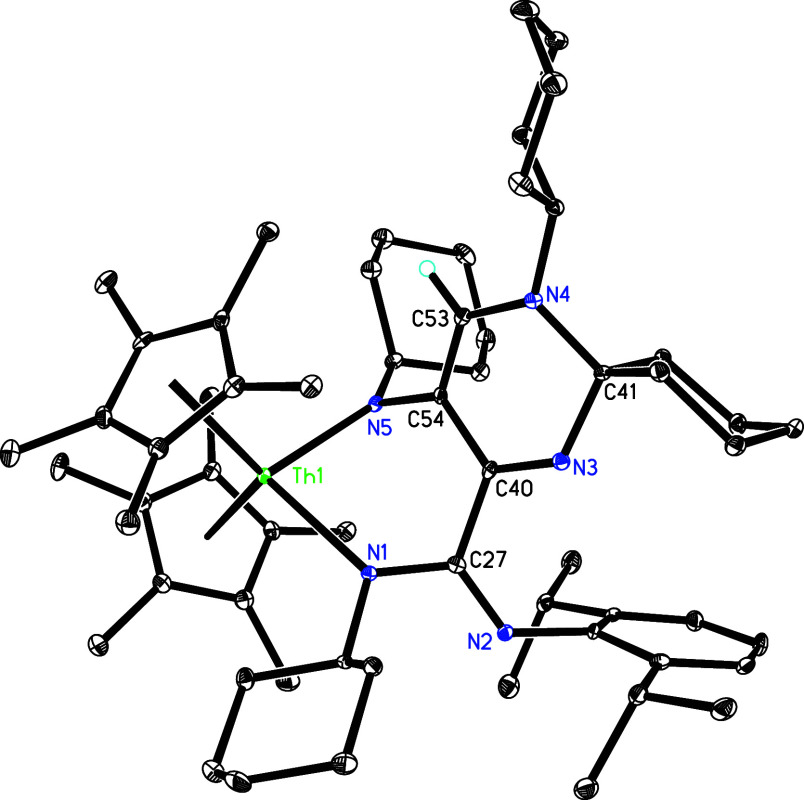
Molecular structure of **29** (thermal ellipsoids drawn at the 35% probability level).

### Reaction with Organic Azides

Analogous to the thorium imido compounds (η^5^-C_5_Me_5_)_2_Th=N­(*p*-tolyl)­(dmap)_2_ (Figure S1),^8c,9^ (η^5^-C_5_Me_5_)_2_Th=N­(mesityl)­(dmap) (Figure S2),^7g,9^ [η^5^-1,3-(Me_3_C)_2_C_5_H_3_]_2_Th=N­(dipp)­(dmap) (Figure S3),^8b,9^ and [η^5^-1,2,4-(Me_3_C)_3_C_5_H_2_]_2_Th=N­(*p*-tolyl) (Figure S4),^7d,9^ complex **4** also reacts with organic azides. However, the reaction outcomes vary significantly depending on ligand architecture. For example, unlike (η^5^-C_5_Me_5_)_2_Th=N­(*p*-tolyl)­(dmap)_2_ and (η^5^-C_5_Me_5_)_2_Th=N­(mesityl)­(dmap), which form the tetraazametallacyclopentene (η^5^-C_5_Me_5_)_2_Th­[N­(*p*-tolyl)­N=NN­(*p*-tolyl)] (Figures S1 and S2),^7g,8c,9^ and [η^5^-1,2,4-(Me_3_C)_3_C_5_H_2_]_2_Th=N­(*p*-tolyl) yielding the tetraazametallacyclopentene [η^5^-1,2,4-(Me_3_C)_3_C_5_H_2_]_2_Th­[N­(*p*-tolyl)­N=NN­(*p*-tolyl)] (Figure S4)^7d,9^ with (*p*-tolyl)­N_3_, respectively, **4** behaves similarly to [η^5^-1,3-(Me_3_C)_2_C_5_H_3_]_2_Th=N­(dipp)­(dmap) (Figure S3).^8b,9^ Treatment of **4** with (*p*-tolyl)­N_3_ affords the bis-amido complex (η^5^-C_5_Me_5_)_2_Th­[NH­(*p*-tolyl)]­[2-(dippN_3_)-4-(Me_2_N)­C_5_H_3_N] (**30**) (Scheme 12), a product consistent with the steric bulk of the 2,6- ^*i*^Pr_2_C_6_H_3_ substituent. The proposed reaction pathway includes the initial reaction of **4** with (*p*-tolyl)­N_3_, releasing dmap and forming a five-membered tetraazametallacyclopentene. However, to reduce steric crowding, dippN_3_ is eliminated yielding the thorium imido (η^5^-C_5_Me_5_)_2_Th=N­(*p*-tolyl). The instability of this imido intermediate, due to insufficient steric protection by the (η^5^-C_5_Me_5_)_2_Th fragment, leads to coordination of the released dmap ligand, forming the amido pyridyl complex (η^5^-C_5_Me_5_)_2_Th­[NH­(*p*-tolyl)]­[κ^2^-*C*,*N*-4-(Me_2_N)­C_5_H_3_N],^8c^ followed by subsequent reaction with the eliminated dippN_3_ ligand, affording the final product **30** (Scheme 12). The molecular structure of **30** is presented in Figure 26, for selected bond distances and angles consult Table 1. The Th–N(2) distance of 2.527(5) Å is longer than the Th–N(1) distance of 2.310(5) Å, but it is comparable to the Th–N(5) distance of 2.617(5) Å, presumably attributed to steric repulsion between the [2-(dippN_3_)-4-(Me_2_N)­C_5_H_3_N] fragment and the Cp ligand.

**Scheme 12:**
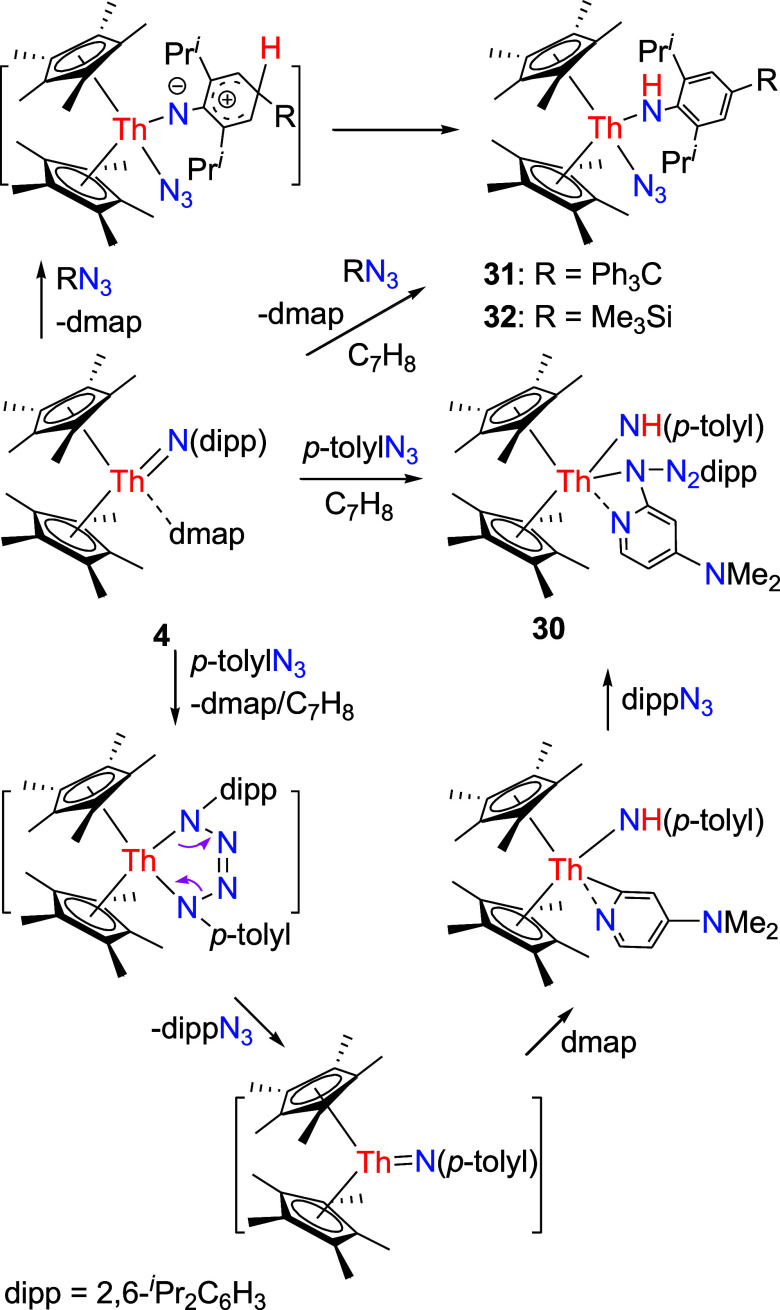
Synthesis of Compounds **30–32**

**Figure 26:**
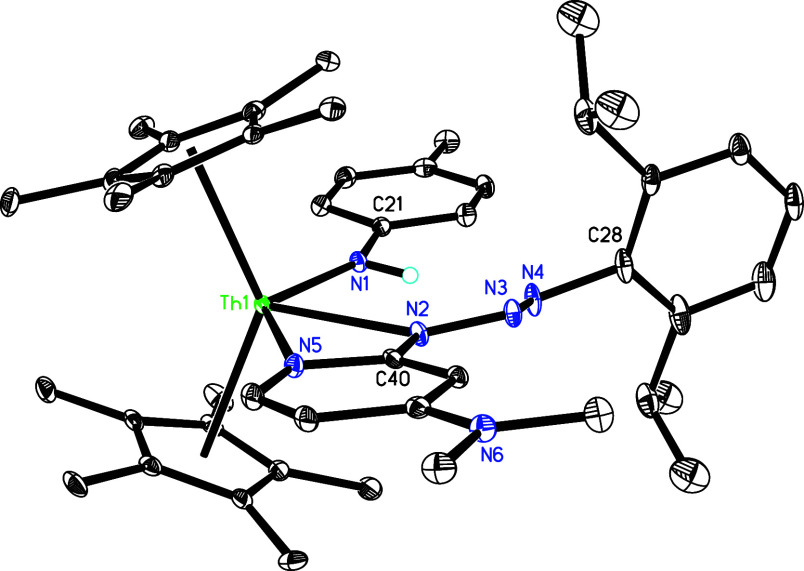
Molecular structure of **30** (thermal ellipsoids drawn at the 35% probability level).

Nevertheless, the reaction of **4** with bulky azides Ph_3_CN_3_ or Me_3_SiN_3_ proceeds via a Friedel–Crafts type alkylation, contrasting the behavior of [η^5^-1,3-(Me_3_C)_2_C_5_H_3_]_2_Th=N­(dipp)­(dmap), which forms an imido dmap adduct [η^5^-1,3-(Me_3_C)_2_C_5_H_3_]_2_Th=N­(2,6-^*i*^Pr_2_-4-(Ph_3_C)­C_6_H_2_)­(dmap) with Ph_3_CN_3_ and a bis–azido complex [η^5^-1,3-(Me_3_C)_2_C_5_H_3_]_2_Th­(N_3_)_2_(dmap)_2_ with Me_3_SiN_3_ (Figure S3).^8b,9^ Instead **4** yields with Ph_3_CN_3_ or Me_3_SiN_3_ the azido amido complexes (η^5^-C_5_Me_5_)_2_Th­(N_3_)­[NH­(2,6-^*i*^Pr_2_-4-(Ph_3_C)­C_6_H_2_] (**31**) and (η^5^-C_5_Me_5_)_2_Th­(N_3_)­[NH­(2,6-^*i*^Pr_2_-4-(Me_3_Si)­Ph)] (**32**) (Scheme 12), respectively. This distinct reactivity is attributed to the enhanced steric bulk of the (η^5^-C_5_Me_5_)_2_Th fragment. By contrast, (η^5^-C_5_Me_5_)_2_Th=N­(*p*-tolyl)­(dmap)_2_ forms different products with these azides, yielding the bis-amido complex (η^5^-C_5_Me_5_)_2_Th­[NH­(*p*-tolyl)]­[κ^2^-*N*,*N*-2-N­(NNCPh_3_)-4-(Me_2_N)­C_5_H_3_N] with Ph_3_CN_3_ and the azido amido complex (η^5^-C_5_Me_5_)_2_Th­(N_3_)­[N­(*p*-tolyl)­SiMe_3_] with Me_3_SiN_3_ (Figure S1),^8c,9^ respectively. Here we assume that the reactivity difference originates from the reduced steric hindrance of the *p*-tolyl group. The molecular structure of **31** is shown in Figure 27, whereas the molecular structure of **32** is provided in the Supporting Information. In complex **31**, the Th–N(1) and Th–N(2) distances are 2.284(5) and 2.370(6) Å, respectively, which are comparable to those found in **32** (2.309(6) and 2.338(6) Å).

**Figure 27:**
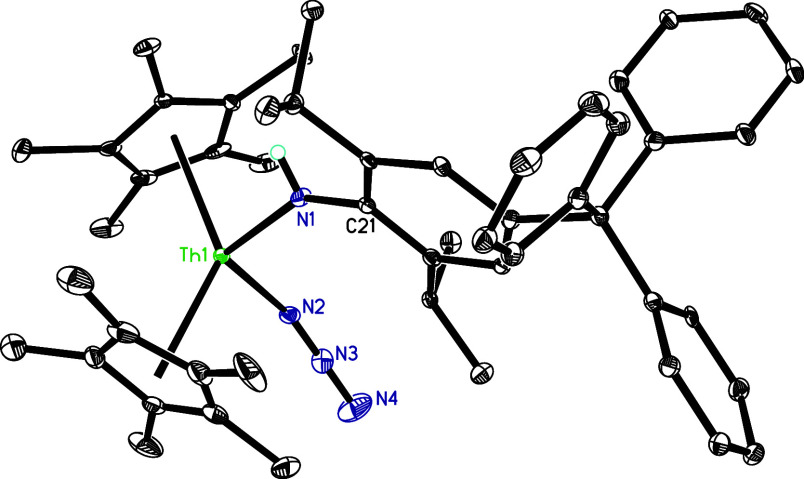
Molecular structure of **31** (thermal ellipsoids drawn at the 35% probability level).

## Conclusions

This study has successfully synthesized and structurally characterized the Lewis base-supported thorium imido metallocene (η^5^-C_5_Me_5_)_2_Th­(=Ndipp)­(dmap) (**4**). This complex exhibits diverse and tunable reactivity toward a broad range of small molecules, including Lewis bases, halides, alkynes, nitriles, isonitriles, azides, and elemental sulfur (S_8_). The complex **4** exhibits distinct reaction pathways that are strongly influenced by both the steric and electronic properties of its supporting ligands and substrates. For instance, reactions with bulky halidosilanes and azides proceed via Friedel–Crafts-type C–H activation, yielding stable amido complexes (**11**–**12**, and **31**–**32**). Moreover, it may react with Me_3_PO, thiazole, pyridine *N*-oxide derivatives, primary amines, hydrazine derivatives, clorosilanes, elemental sulfur (S_8_), alkynes, carbodiimides, aldehydes, ketones, CS_2_, amidates, imines R_2_C=NH, organic nitriles and isonitriles, and organic azides, leading to the formation of the Me_3_PO imido adduct, amido thiazolyl, amido-pyridinium-oxide, bis-amido, dichloro, heterometallacycles, alkynyl amido complex, oxido, amido enolyl, bis-amidate, bis-iminato, imido and iminato amido complexes, respectively. Furthermore, while **4** forms with CuBr the bromo amido complex (η^5^-C_5_Me_5_)_2_Th­(Br)­[NH­(2,6-^*i*^Pr_2_-4-(Me_5_C_5_)­C_6_H_2_)] (**13**), its exposure to AgI induces C–C coupling to yield the bimetallic iodo amido complex [(η^5^-C_5_Me_5_)_2_Th­(I)]_2_[μ-4,4′–(NH–2,6-^*i*^Pr_2_C_6_H_2_)_2_] (**14**).

Overall, while the predominant reactivity patterns within this series of thorium imido metallocenes are similar, subtle changes in the steric environment at the Th atom such as the substituents on the Cp or imido ligands modulate the individual reactivity patterns. For example, in contrast to [η^5^-1,3-(Me_3_C)_2_C_5_H_3_]_2_Th=N­(dipp)­(dmap),^8b^ an equilibrium between the imido complex **4** and the amido pyridyl complex (η^5^-C_5_Me_5_)_2_Th­(NHdipp)­[κ^2^-*C*,*N*-4-(Me_2_N)­C_5_H_3_N] (**4′**) exists in C_6_D_6_ solution. While complex **4** forms a bromo amido complex (η^5^-C_5_Me_5_)_2_Th­(Br)­[NH­(2,6-^*i*^Pr_2_-4-(Me_5_C_5_)­C_6_H_2_)] (**13**) in the presence of CuBr, complex [η^5^-1,3-(Me_3_C)_2_C_5_H_3_]_2_Th=N­(dipp)­(dmap) reacts with CuCl to yield a heterobimetallic compound [η^5^-1,3-(Me_3_C)_2_C_5_H_3_]_2_Th­(Cl)­[N­(dipp)­Cu­(dmap)] (Figure S3).^8b,9^ Reaction of complex **4** with PhC≡C–C≡CPh affords an amido pyridyl complex (η^5^-C_5_Me_5_)_2_Th­[N­(dipp)­C­(C_2_Ph)=CHPh]­(κ^2^-*C*,*N*-4-Me_2_NC_5_H_3_N) (**17**), whereas complex [η^5^-1,3-(Me_3_C)_2_C_5_H_3_]_2_Th=N­(dipp)­(dmap) forms a pyridyl alkenyl species [η^5^-1,3-(Me_3_C)_2_C_5_H_3_]_2_Th­[(1-(2,6-^*i*^Pr_2_C_6_H_3_)-2,5-Ph_2_C_4_HN]­(κ^2^-*C*,*N*-4-Me_2_NC_5_H_3_N) (Figure S3).^8b,9^ Moreover, while complex **4** reacts with PhC≡CH to form an alkynyl amido complex (η^5^-C_5_Me_5_)_2_Th­(NHdipp)­(C≡CPh) (**18**), the related derivative [η^5^-1,3-(Me_3_C)_2_C_5_H_3_]_2_Th=N­(dipp)­(dmap) gives a bis–alkynyl complex [η^5^-1,3-(Me_3_C)_2_C_5_H_3_]_2_Th­(C≡CPh)_2_(dmap) (Figure S3).^8c,9^ Exposure of **4** to *p*-tolylCHO results in the oxido dimer [(η^5^-C_5_Me_5_)_2_Th­(dmap)]_2_(μ-O)_2_ (**20**), whereas [η^5^-1,3-(Me_3_C)_2_C_5_H_3_]_2_Th=N­(dipp)­(dmap) affords an alkoxyl amidate complex [η^5^-1,3-(Me_3_C)_2_C_5_H_3_]_2_Th­[OCH_2_(*p*-MePh)]­[OC­(*p*-MePh)­N­(dipp)] (Figure S3).^8b,9^ However, no reaction of **4** is observed with sterically very encumbered substrates such as Ph_2_E_2_ (E = S, Se), Ph_2_CO, or Ph_2_CS, while the less sterically encumbered derivative [η^5^-1,3-(Me_3_C)_2_C_5_H_3_]_2_Th=N­(dipp)­(dmap) does react (Figure S3).^8b,9^ In the presence of CS_2_ complex **4** yields a four-membered metallaheterocycle (η^5^-C_5_Me_5_)_2_Th­[SC=N­(dipp)-S]­(dmap) (**22**), while [η^5^-1,3-(Me_3_C)_2_C_5_H_3_]_2_Th=N­(dipp)­(dmap) forms the μ-sulfido dimer {[η^5^-1,3-(Me_3_C)_2_C_5_H_3_]_2_Th}_2_(μ-S)_2_ (Figure S3).^8b,9^ Furthermore, a bis–iminato complex (η^5^-C_5_Me_5_)_2_Th­(N=CPh_2_)_2_ (**24**) is formed from reaction of complex **4** with Ph_2_C=NH, whereas [η^5^-1,3-(Me_3_C)_2_C_5_H_3_]_2_Th=N­(dipp)­(dmap) affords with (*p*-tolyl)_2_C=NH an amido–iminato complex [η^5^-1,3-(Me_3_C)_2_C_5_H_3_]_2_Th­[NH­(dipp)]­[N=C­(*p*-tolyl)_2_] (Figure S3).^8c,9^ Contrary to [η^5^-1,3-(Me_3_C)_2_C_5_H_3_]_2_Th=N­(dipp)­(dmap) yielding with PhCN the amido pyridyl complex [η^5^-1,3-(Me_3_C)_2_C_5_H_3_]_2_Th­[NHC­(Ph)=Ndipp]­[κ^2^-*C*,*N*-4-(Me_2_N)­C_5_H_3_N] (Figure S3),^8b,9^ the more sterically encumbered **4** forms the dmap imido adduct (η^5^-C_5_Me_5_)_2_Th­[=NC­(Ph)=N­(dipp)]­(dmap) (**25**). Diverging reactions are also observed for the reaction with 1,4-(CH_2_)_4_(CN)_2_, while **4** yields the bis-amido complex (η^5^-C_5_Me_5_)_2_Th­{NH­[C=C­(CN)­(CH_2_)_3_]}]­{NH­[C=NC­(=Ndipp)­(CH_2_)_4_]} (**28**), the eight-membered metallacycle [η^5^-1,3-(Me_3_C)_2_C_5_H_3_]_2_Th­[NHC=C­{(CH_2_)_3_}­C­(=NH)­NC=N­{C­(=Ndipp)­(CH_2_)_4_}] is formed from complex [η^5^-1,3-(Me_3_C)_2_C_5_H_3_]_2_Th=N­(dipp)­(dmap) (Figure S3).^8b,9^ Treatment of **4** with C_6_H_11_NC affords the six-membered metallaheterocycle (η^5^-C_5_Me_5_)_2_Th­[2-C­(=Ndipp)­N­(C_6_H_11_)-3-N­(C_6_H_11_)-5-(C_6_H_11_)-6,6-(CH_2_)_5_-(1,3-C_4_HN_2_)] (**29**), whereas complex [η^5^-1,3-(Me_3_C)_2_C_5_H_3_]_2_Th=N­(dipp)­(dmap) gives the five-membered metallaheterocycle [η^5^-1,3-(Me_3_C)_2_C_5_H_3_]_2_Th­[N­(C_6_H_11_)­CH=C­{C­(=Ndipp)­N=C­(CH_2_)_5_}­N­(C_6_H_11_)] (Figure S3).^8b,9^ Furthermore, while treatment of **4** with Ph_3_CN_3_ or Me_3_SiN_3_ affords the azido amido compounds (η^5^-C_5_Me_5_)_2_Th­(N_3_)­[NH­(2,6-^*i*^Pr_2_-4-(Ph_3_C)­C_6_H_2_)] (**31**) and (η^5^-C_5_Me_5_)_2_Th­(N_3_)­[NH­(2,6-^*i*^Pr_2_-4-(Me_3_Si)­C_6_H_2_)] (**32**), respectively, complex [η^5^-1,3-(Me_3_C)_2_C_5_H_3_]_2_Th=N­(dipp)­(dmap) gives the imido dmap adduct [η^5^-1,3-(Me_3_C)_2_C_5_H_3_]_2_Th=N­(2,6-^*i*^Pr_2_-4-(Ph_3_C)­C_6_H_2_)­(dmap) with Ph_3_CN_3_ (Figure S3),^8b,9^ and a bis–azido complex [η^5^-1,3-(Me_3_C)_2_C_5_H_3_]_2_Th­(N_3_)_2_(dmap)_2_ with Me_3_SiN_3_ (Figure S3),^8b,9^ respectively.

However, not only the substituents on the Cp ring affect the reactivity of the imido complexes but also the substituents on the imido moiety. For example, complexes **4** and (η^5^-C_5_Me_5_)_2_Th=N­(mesityl)­(dmap) form monodmap adducts, whereas (η^5^-C_5_Me_5_)_2_Th=N­(*p*-tolyl)­(dmap)_2_ crystallizes with two coordinated dmap ligands. While an equilibrium between the imido **4** and the amido pyridyl (η^5^-C_5_Me_5_)_2_Th­(NHdipp)­[κ^2^-*C*,*N*-4-(Me_2_N)­C_5_H_3_N] (**4′**), or between imido (η^5^-C_5_Me_5_)_2_Th=N­(mesityl)­(dmap) and amido pyridyl (η^5^-C_5_Me_5_)_2_Th­(NHmesityl)­[κ^2^-*C*,*N*-4-(Me_2_N)­C_5_H_3_N], is detected by ^1^H NMR spectroscopy in C_6_D_6_ solution,^7f^ only resonances attributed to the amido pyridyl (η^5^-C_5_Me_5_)_2_Th­(NHdipp)­[κ^2^-*C*,*N*-4-(Me_2_N)­C_5_H_3_N] and free dmap are observed in the ^1^H NMR spectrum of (η^5^-C_5_Me_5_)_2_Th=N­(*p*-tolyl)­(dmap)_2_ recorded in the C_6_D_6_ solution.^8c^ Complex **4** forms the Me_3_PO adduct (η^5^-C_5_Me_5_)_2_Th­(=Ndipp)­(OPMe_3_) (**5**), whereas (η^5^-C_5_Me_5_)_2_Th=N­(*p*-tolyl)­(dmap)_2_ yields the amido alkyl compound (η^5^-C_5_Me_5_)_2_Th­[NH­(*p*-tolyl)]­[κ^2^-*C*,*N*–N­(*p*-tolyl)­P­(Me_2_)­CH_2_] with Me_3_PO (Figure S1).^8c,9^ While treatment of **4** with PhSiH_2_Cl gives the dichloride complex (η^5^-C_5_Me_5_)_2_ThCl_2_(dmap) (**10**), (η^5^-C_5_Me_5_)_2_Th=N­(*p*-tolyl)­(dmap)_2_ affords the chloro pyridyl complex (η^5^-C_5_Me_5_)_2_Th­(Cl)­[κ^2^-*C*,*N*-4-(Me_2_N)­C_5_H_3_N] (Figure S1).^8c,9^ Moreover, the reaction of **4** with CuBr gives a bromo amido species (η^5^-C_5_Me_5_)_2_Th­(Br)­[NH­(2,6-^*i*^Pr_2_-4-(Me_5_C_5_)­C_6_H_2_)] (**13**), whereas (η^5^-C_5_Me_5_)_2_Th=N­(*p*-tolyl)­(dmap)_2_ and (η^5^-C_5_Me_5_)_2_Th=N­(mesityl)­(dmap) afford the dibromide complex (η^5^-C_5_Me_5_)_2_ThBr_2_(dmap) and the heterobimetallic complex (η^5^-C_5_Me_5_)_2_Th­(Br)­[N­(mesityl)­Cu­(dmap)] (Figures S1 and S2),^7f,8c,9^ respectively. Treatment of complex **4** with elemental sulfur (S_8_) forms a six-membered disulfido complex (η^5^-C_5_Me_5_)_2_Th­[SSN­(dipp)­SS] (**15**), while (η^5^-C_5_Me_5_)_2_Th=N­(*p*-tolyl)­(dmap)_2_ yields the six-membered amido–sulfido complex (η^5^-C_5_Me_5_)_2_Th­[N­(*p*-tolyl)­S_4_] (Figure S1).^8c,9^ Complexes **4** and (η^5^-C_5_Me_5_)_2_Th=N­(mesityl)­(dmap) are inert in the presence of PhC≡CPh, whereas (η^5^-C_5_Me_5_)_2_Th=N­(*p*-tolyl)­(dmap)_2_ does react (Figure S1).^8c,9^ In addition, while **4** exhibits no reactivity toward Ph_2_CO or Ph_2_CS, complexes (η^5^-C_5_Me_5_)_2_Th=N­(*p*-tolyl)­(dmap)_2_ and (η^5^-C_5_Me_5_)_2_Th=N­(mesityl)­(dmap) do react (Figures S1 and S2).^7g,8c,9^ Reaction of complexes **4** or (η^5^-C_5_Me_5_)_2_Th=N­(mesityl)­(dmap) with CS_2_ gives a four-membered metallaheterocycle (η^5^-C_5_Me_5_)_2_Th­[SC=N­(dipp)-S]­(dmap) (**22**) or (η^5^-C_5_Me_5_)_2_Th­[SC=N­(mesityl)-S]­(dmap) (Figure S2),^7g,9^ respectively, whereas (η^5^-C_5_Me_5_)_2_Th=N­(*p*-tolyl)­(dmap)_2_ affords the dimer [(η^5^-C_5_Me_5_)_2_Th]_2_{μ-[N­(*p*-tolyl)­C­(S)­S]}_2_ (Figure S1).^8c,9^ Exposure of **4** or (η^5^-C_5_Me_5_)_2_Th=N­(mesityl)­(dmap) with PhCN yields the imido species (η^5^-C_5_Me_5_)_2_Th­[=NC­(Ph)=N­(dipp)]­(dmap) (**25**) and (η^5^-C_5_Me_5_)_2_Th­[=NC­(Ph)=N­(mesityl)]­(dmap) (Figure S2),^7g,9^ respectively, whereas (η^5^-C_5_Me_5_)_2_Th=N­(*p*-tolyl)­(dmap)_2_ gives the amidinyl pyridyl complex (η^5^-C_5_Me_5_)_2_Th­[η^3^-NHC­(Ph)­N­(*p*-tolyl)]­[κ^2^-*C*,*N*-4-(Me_2_N)­C_5_H_3_N] (Figure S1).^8c,9^ Reaction of **4** with C_6_H_11_NC yields a six-membered metallaheterocycle (η^5^-C_5_Me_5_)_2_Th­[2-C­(=Ndipp)­N­(C_6_H_11_)-3-N­(C_6_H_11_)-5-(C_6_H_11_)-6,6-(CH_2_)_5_-(1,3-C_4_HN_2_)] (**29**), whereas (η^5^-C_5_Me_5_)_2_Th=N­(*p*-tolyl)­(dmap)_2_ forms the amido alkenyl complex (η^5^-C_5_Me_5_)_2_Th­[NH­(*p*-tolyl)]­[κ^2^-*C*,*N*-2-(C_6_H_11_NC)-4-(Me_2_N)­C_5_H_3_N] (Figure S1).^8c,9^ Furthermore, while the tetraazametallacyclopentene (η^5^-C_5_Me_5_)_2_Th­[N­(*p*-tolyl)­N=NN­(*p*-tolyl)] can be isolated from (η^5^-C_5_Me_5_)_2_Th=N­(*p*-tolyl)­(dmap)_2_ (Figure S1)^8c,9^ with (*p*-tolyl)­N_3_, NN cleavage and (mesityl)­N_3_ or (dipp)­N_3_ elimination occurs for those tetraazametallacyclopentene complexes derived from (η^5^-C_5_Me_5_)_2_Th=N­(mesityl)­(dmap) (Figure S2)^7g,9^ or **4**, respectively, with (*p*-tolyl)­N_3_. Treatment of **4** with Ph_3_CN_3_ or Me_3_SiN_3_ yields the azido amido species (η^5^-C_5_Me_5_)_2_Th­(N_3_)­[NH­(2,6-^*i*^Pr_2_-4-(Ph_3_C)­C_6_H_2_)] (**31**) and (η^5^-C_5_Me_5_)_2_Th­(N_3_)­[NH­(2,6-^*i*^Pr_2_-4-(Me_3_Si)­C_6_H_2_)] (**32**), respectively, whereas (η^5^-C_5_Me_5_)_2_Th=N­(*p*-tolyl)­(dmap)_2_ furnishes the bis-amido complex (η^5^-C_5_Me_5_)_2_Th­[NH­(*p*-tolyl)]­[κ^2^-*N*,*N*-2-N­(NNCPh_3_)-4-(Me_2_N)­C_5_H_3_N] with Ph_3_CN_3_ and the azido amido complex (η^5^-C_5_Me_5_)_2_Th­(N_3_)­[N­(*p*-tolyl)­SiMe_3_] with Me_3_SiN_3_ (Figure S1),^8c,9^ respectively. Further syntheses and explorations of actinide imido complexes are in progress and will be reported in the future.

## Experimental Section

### General Procedures

All reactions and product manipulations were performed under a N_2_ atmosphere with rigid exclusion of air and moisture using standard Schlenk or cannula techniques, or in a glovebox. All organic solvents were freshly distilled from sodium benzophenone ketyl immediately prior to use. (η^5^-C_5_Me_5_)_2_ThMe_2_ (**1**)^7f,11^ was prepared according to literature method. All other chemicals were purchased from Aldrich Chemical Co. and Beijing Chemical Co. and used as received unless stated otherwise. Infrared spectra were recorded in KBr pellets on an Avatar 360 Fourier transform spectrometer. ^1^H and ^13^C­{^1^H} spectra were recorded on a JEOL 400 at 400 and 100 MHz, respectively, or recorded on a JEOL 600 spectrometer at 600 and 151 MHz, respectively. ^29^Si­{^1^H} NMR spectra were recorded on a JEOL 600 spectrometer at 119.2 MHz. All chemical shifts are reported in δ units with reference to the residual protons of the deuterated solvents, which served as internal standards, for proton and carbon chemical shifts, and to external Me_4_Si (0.00 ppm) for silicon chemical shifts. Melting points were obtained on an X-6 melting point apparatus and were uncorrected. Elemental analyses were performed on a Vario EL elemental analyzer.

*Caution*: Natural thorium (primary isotope ^232^Th) is a weak α-emitter (4.012 MeV) with a half-life of 1.41 × 10^10^ years. Therefore, manipulations and reactions should be carried out in monitored fume hoods or in an inert-atmosphere drybox in a laboratory equipped with α- and β-counting equipment. Moreover, all organic reactants used in this work are standard or commercially available reagents, there are no uncommon hazards involved.

### Preparation of (η^5^-C_5_Me_5_)_2_Th­(NHdipp)_2_ (**2**). Method **A**

A toluene (10 mL) solution of dippNH_2_ (0.71 g, 4.0 mmol) was added to a stirred toluene (20 mL) solution of (η^5^-C_5_Me_5_)_2_ThMe_2_ (**1**; 1.06 g, 2.0 mmol) at room temperature. After the solution was stirred at room temperature overnight, the solvent was removed. The residue was extracted with benzene (10 mL × 3) and filtered. The volume of the filtrate was reduced to 10 mL, colorless crystals of **2** formed when this solution was cooled at 10 °C for 1 day. Crystals of **2** were isolated by filtration, quickly washed with cold *n*-hexane (5 mL), and dried under vacuum at room temperature overnight. Yield: 1.57 g (92%). M.p.: 176–178 °C (dec.). ^1^H NMR (C_6_D_6_): δ 7.20 (d, *J* = 7.5 Hz, 4H, phenyl), 7.00 (t, *J* = 7.5 Hz, 2H, phenyl), 4.58 (s, 2H, N*H*), 3.42 (m, 4H, C*H*(CH_3_)_2_), 1.91 (s, 30H, CpC*H*_3_), 1.34 (d, *J* = 6.6 Hz, 24H, C*H*(CH_3_)_2_) ppm. ^13^C­{^1^H} NMR (C_6_D_6_): δ 150.9 (phenyl *C*), 139.5 (phenyl *C*), 126.0 (ring *C*), 122.7 (phenyl *C*), 120.7 (phenyl *C*), 28.8 (*C*H­(CH_3_)_2_), 25.1 (CH­(*C*H_3_)_2_), 11.6 (Cp*C*H_3_) ppm. IR (KBr, cm^–1^): ν 2958 (s), 2920 (s), 2866 (s), 1588 (s), 1459 (s), 1429 (s), 1311 (s), 1235 (s), 1189 (s), 1108 (s), 1036 (s), 837 (s). Anal. Calcd for C_44_H_66_N_2_Th: C, 61.81; H, 7.78; N, 3.28. Found: C, 61.83; H, 7.76; N, 3.30.

### Method **B**. NMR Scale

A C_6_D_6_ (0.3 mL) solution of dippNH_2_ (7.1 mg, 0.04 mmol) was slowly added to a J. Young NMR tube charged with (η^5^-C_5_Me_5_)_2_ThMe_2_ (**1**; 10.6 mg, 0.02 mmol) and C_6_D_6_ (0.2 mL). Resonances of **2** and that of methane were observed by ^1^H NMR spectroscopy (100% conversion) when this solution was kept at room temperature overnight.

### Preparation of (η^5^-C_5_Me_5_)_2_Th­[NH­(dipp)]­(κ^2^-*C*,*N*–C_5_H_4_N) (**3**). Method **A**

A toluene (10 mL) solution of dippNH_2_ (0.35 g, 2.0 mmol) and pyridine (0.16 g, 2.0 mmol) was added to a stirred toluene (20 mL) solution of (η^5^-C_5_Me_5_)_2_ThMe_2_ (**1**; 1.06 g, 2.0 mmol) at room temperature. After the solution was stirred at room temperature overnight, the solvent was removed. The residue was extracted with benzene (10 mL × 3) and filtered. The volume of the filtrate was reduced to 10 mL, yellow crystals of **3** formed when this solution was cooled to 10 °C for 1 day. Crystals of **3** were isolated by filtration, quickly washed with cold *n*-hexane (5 mL), and dried under vacuum at room temperature overnight. Yield: 1.36 g (90%). M.p.: 224–226 °C (dec.). ^1^H NMR (C_6_D_6_): δ 8.31 (d, *J* = 5.2 Hz, 1H, py), 7.91 (d, *J* = 7.3 Hz, 1H, py), 7.28 (d, *J* = 7.3 Hz, 2H, phenyl), 7.18 (t, *J* = 7.5 Hz, 1H, py), 6.97 (t, *J* = 7.6 Hz, 1H, phenyl), 6.65 (m, 1H, py), 4.26 (s, 1H, C*H*(CH_3_)_2_), 4.21 (s, 1H, N*H*), 3.42 (s, 1H, C*H*(CH_3_)_2_), 1.84 (s, 30H, CpC*H*_3_), 1.49 (s, 6H, CH­(C*H*_3_)_2_), 1.47 (s, 6H, CH­(C*H*_3_)_2_) ppm. ^13^C­{^1^H} NMR (C_6_D_6_): δ 232.3 (Th*C*), 152.8 (aryl *C*), 143.8 (aryl *C*), 136.8 (aryl *C*), 132.4 (aryl *C*), 123.6 (aryl *C*), 123.24 (aryl *C*), 123.15 (ring *C*), 122.6 (aryl *C*), 118.1 (aryl *C*), 29.0 (*C*H­(CH_3_)_2_), 27.7 (*C*H­(CH_3_)_2_), 25.1 (CH­(*C*H_3_)_2_), 24.0 (CH­(*C*H_3_)_2_), 11.2 (Cp*C*H_3_) ppm. IR (KBr, cm^–1^): ν 2956 (s), 2901 (s), 2860 (s), 1588 (s), 1574 (s), 1458 (s), 1426 (s), 1378 (s), 1321 (s), 1242 (s), 1214 (s), 1020 (s), 883 (s), 842 (s). Anal. Calcd for C_37_H_52_N_2_Th: C, 58.72; H, 6.93; N, 3.70. Found: C, 58.75; H, 6.92; N, 3.71.

### Method **B**. NMR Scale

A C_6_D_6_ (0.3 mL) solution of dippNH_2_ (3.5 mg, 0.02 mmol) and pyridine (1.6 mg, 0.02 mmol) was slowly added to a J. Young NMR tube charged with (η^5^-C_5_Me_5_)_2_ThMe_2_ (**1**; 10.6 mg, 0.02 mmol) and C_6_D_6_ (0.2 mL). Resonances of **3** and that of methane were observed by ^1^H NMR spectroscopy (100% conversion) when this solution was kept at room temperature overnight.

### Preparation of (η^5^-C_5_Me_5_)_2_Th­(=Ndipp)­(dmap) (4) and (η^5^-C_5_Me_5_)_2_Th­(NHdipp)­[κ^2^-*C*,*N*-4-(Me_2_N)­C_5_H_3_N] (**4′**). Method **A**

A toluene (10 mL) solution of dippNH_2_ (0.35 g, 2.0 mmol) and dmap (0.25 g, 2.05 mmol) was added to a stirred toluene (20 mL) solution of (η^5^-C_5_Me_5_)_2_ThMe_2_ (**1**; 1.06 g, 2.0 mmol) at room temperature. After the solution was stirred 80 °C overnight, the solvent was removed. The residue was extracted with benzene (10 mL × 3) and filtered. The volume of the filtrate was reduced to 10 mL, yellow crystals formed when this solution was cooled to 10 °C for 1 day. Crystals were isolated by filtration, quickly washed with cold *n*-hexane (5 mL) and dried under vacuum at room temperature overnight. The product was identified as **4** by X-ray diffraction analysis. Yield: 1.31 g (82%). M.p.: 280–282 °C (dec.). Nevertheless, two isomers **4** and **4′** were observed in a C_6_D_6_ solution, and the ratio of **4** and **4′** is ca. 1.5:1. Complex **4**: ^1^H NMR (C_6_D_6_): δ 8.47 (d, *J* = 6.2 Hz, 2H, py), 7.30 (m, 2H, phenyl), 6.83 (t, *J* = 7.5 Hz, 1H, phenyl), 6.06 (d, *J* = 6.1 Hz, 2H, py), 4.10 (s, 2H, C*H*(CH_3_)_2_), 2.10 (s, 30H, CpC*H*_3_), 1.99 (s, 6H, N­(C*H*_3_)_2_), 1.60 (s, 6H, CH­(C*H*_3_)_2_), 1.49 (s, 6H, CH­(C*H*_3_)_2_) ppm. ^13^C­{^1^H} NMR (C_6_D_6_): δ 153.7 (py *C*), 150.6 (py *C*), 142.9 (phenyl *C*), 128.4 (phenyl *C*), 127.9 (phenyl *C*), 122.5 (ring *C*), 122.3 (phenyl *C*), 106.6 (py *C*), 38.6 (N­(*C*H_3_)_2_), 28.1 (*C*H­(CH_3_)_2_), 26.2 (CH­(*C*H_3_)_2_), 11.8 (Cp*C*H_3_) ppm. Complex **4′**: ^1^H NMR (C_6_D_6_): δ 8.17 (d, *J* = 6.1 Hz, 1H, py), 7.30 (m, 2H, phenyl), 7.20 (s, 1H, py), 6.99 (t, *J* = 7.4 Hz, 1H, phenyl), 6.11 (d, *J* = 6.1 Hz, 1H, py), 4.49 (s, 1H, C*H*(CH_3_)_2_), 4.18 (s, 1H, N*H*), 3.50 (s, 1H, C*H*(CH_3_)_2_), 2.39 (s, 6H, N­(C*H*_3_)_2_), 1.96 (s, 30H, CpC*H*_3_), 1.60 (s, 6H, CH­(C*H*_3_)_2_), 1.49 (s, 6H, CH­(C*H*_3_)_2_) ppm. ^13^C­{^1^H} NMR (C_6_D_6_): δ 157.8 (py *C*), 155.0 (py *C*), 153.2 (py *C*), 128.5 (phenyl *C*), 128.1 (phenyl *C*), 122.8 (ring *C*), 117.6 (phenyl *C*), 114.6 (phenyl *C*), 111.9 (py *C*), 107.8 (py *C*), 38.1 (N­(*C*H_3_)_2_), 28.1 (*C*H­(CH_3_)_2_), 22.5 (CH­(*C*H_3_)_2_), 11.4 (Cp*C*H_3_) ppm. IR (KBr, cm^–1^): ν 2961 (s), 2922 (s), 2866 (s), 1612 (s), 1528 (s), 1439 (s), 1382 (s), 1227 (s), 998 (s), 801 (s). Anal. Calcd for C_39_H_57_N_3_Th: C, 58.56; H, 7.18; N, 5.25. Found: C, 58.58; H, 7.16; N, 5.24. The unambiguous assignment of the NMR resonances corresponding to **4** and **4′** is not straightforward. However, isomers **4** and **4′** were observed in different ratios (ca. 1.5:1) at 20 °C and therefore their NMR resonances feature different intensities. This information was used for the assignment of the respective NMR resonances. Nevertheless, while two isomers **4** and **4′** in a C_6_D_6_ solution were detected by ^1^H NMR spectroscopy, the mixture of **4** and **4′** could be isomerized to the imido isomer **4** during the reactions in benzene or toluene solution, indicating that an equilibrium between **4** and **4′** exists in the solution.

### Method **B**. NMR Scale

A C_6_D_6_ (0.3 mL) solution of (η^5^-C_5_Me_5_)_2_Th­(NHdipp)_2_ (**2**; 8.6 mg, 0.01 mmol) and dmap (2.5 mg, 0.02 mmol) was slowly added to a J. Young NMR tube charged with (η^5^-C_5_Me_5_)_2_ThMe_2_ (**1**; 5.3 mg, 0.01 mmol) and C_6_D_6_ (0.2 mL). Resonances of **4** and **4′** and that of methane were observed by ^1^H NMR spectroscopy (100% conversion) when this solution was heated at 80 °C overnight.

### Preparation of (η^5^-C_5_Me_5_)_2_Th = N­(dipp)­(OPMe_3_)·0.5C_6_H_6_ (5·0.5C_6_H_6_)

A benzene (5 mL) solution of Me_3_PO (23 mg, 0.25 mmol) was added to a benzene (10 mL) solution of (η^5^-C_5_Me_5_)_2_Th=N­(dipp)­(dmap) (**4**; 200 mg, 0.25 mmol) without stirring at room temperature. After the solution was stored at room temperature overnight without stirring, colorless crystals were isolated from the solution by filtration. The product was washed with *n*-hexane and dried at room temperature under vacuum overnight, and the product was identified as **5**·0.5C_6_H_6_ by X-ray diffraction analysis. Yield: 192 mg (95%). M.p.: > 300 °C (dec.). IR (KBr, cm^–1^): ν 2962 (s), 2925 (s), 1610 (s), 1383 (s), 1357 (s), 1260 (s), 1108 (s), 1016 (s), 945 (s), 801 (s). Anal. Calcd for C_38_H_59_NOPTh: C, 56.43; H, 7.35; N, 1.73. Found: C, 56.45; H, 7.37; N, 1.72. This compound was insoluble in common (deuterated) solvents such as pyridine, THF, toluene, and CD_2_Cl_2_, precluding its characterization by NMR spectroscopy.

### Preparation of (η^5^-C_5_Me_5_)_2_Th­[NH­(dipp)]­(κ^2^-*C*,*N*–C_3_H_2_NS) (**6**). Method **A**

A toluene (5 mL) solution of thiazole (22 mg, 0.25 mmol) was added to a toluene (10 mL) solution of (η^5^-C_5_Me_5_)_2_Th=N­(dipp)­(dmap) (**4**; 200 mg, 0.25 mmol) with stirring at room temperature. After this solution was stirred at room temperature overnight, the solvent was removed. The residue was extracted with benzene (10 mL × 3) and filtered. The volume of the filtrate was reduced to 3 mL, colorless crystals of **6** formed when this solution was cooled to 10 °C for 2 days. Crystals of **6** were isolated by filtration, quickly washed with cooled *n*-hexane (2 mL), and dried at room temperature under vacuum overnight. Yield: 172 mg (90%). M.p.: 110–112 °C (dec.). ^1^H NMR (C_6_D_6_): δ 7.71 (d, *J* = 2.7 Hz, 1H, C_3_ *H*_2_NS), 7.58 (d, *J* = 2.7 Hz, 1H, C_3_ *H*_2_NS), 7.25 (m, 2H, phenyl), 6.97 (t, *J* = 7.5 Hz, 1H, phenyl), 4.41 (s, 1H, N*H*), 4.12 (m, 1H, C*H*(CH_3_)_2_), 3.40 (m, 1H, C*H*(CH_3_)_2_), 1.85 (s, 30H, CpC*H*_3_), 1.54 (d, *J* = 6.6 Hz, 6H, CH­(C*H*_3_)_2_), 1.42 (d, *J* = 6.7 Hz, 6H, CH­(C*H*_3_)_2_) ppm. ^13^C­{^1^H} NMR (C_6_D_6_): δ 237.3 (Th*C*), 152.3 (phenyl *C*), 138.0 (*C*_3_H_2_NS), 137.1 (*C*_3_H_2_NS), 135.7 (phenyl *C*), 130.7 (phenyl *C*), 124.3 (ring *C*), 122.8 (phenyl *C*), 122.7 (phenyl *C*), 118.7 (phenyl *C*), 29.9 (*C*H­(CH_3_)_2_), 27.6 (*C*H­(CH_3_)_2_), 25.0 (CH­(*C*H_3_)_2_), 23.8 (CH­(*C*H_3_)_2_), 11.2 (Cp*C*H_3_) ppm. IR (KBr, cm^–1^): ν 2954 (s), 2904 (s), 2862 (s), 1610(s), 1456 (s), 1429 (s), 1246 (s), 1222 (s), 1105 (s), 1056 (s), 1020 (s), 844 (s), 804 (s). Anal. Calcd for C_35_H_50_N_2_STh: C, 55.10; H, 6.61; N, 3.67. Found: C, 55.12; H, 6.59; N, 3.69.

### Method **B**. NMR Scale

A C_6_D_6_ (0.3 mL) solution of thiazole (1.7 mg, 0.02 mmol) was slowly added to a J. Young NMR tube charged with (η^5^-C_5_Me_5_)_2_Th=N­(dipp)­(dmap) (**4**; 16.0 mg, 0.02 mmol) and C_6_D_6_ (0.2 mL). Resonances of **6** and those of dmap were observed by ^1^H NMR spectroscopy (100% conversion) when this solution was kept at room temperature overnight.

### Preparation of (η^5^-C_5_Me_5_)_2_Th­(NHdipp)­(κ^2^-*C*,*O*-2-MeC_5_H_3_NO) (**7**). Method **A**

This compound was prepared as colorless crystals from the reaction of (η^5^-C_5_Me_5_)_2_Th=N­(dipp)­(dmap) (**4**; 200 mg, 0.25 mmol) and 2-MepyNO (28 mg, 0.25 mmol) in toluene (15 mL) at room temperature and recrystallization from a benzene solution by a similar procedure as that in the synthesis of **6**. The product was isolated by filtration, quickly washed with cooled *n*-hexane (2 mL), and dried at room temperature under vacuum overnight. Yield: 169 mg (86%). M.p.: 178–180 °C (dec.). ^1^H NMR (C_6_D_6_): δ 7.55 (d, *J* = 7.2 Hz, 1H, py), 7.33 (m, 2H, phenyl), 7.00 (t, *J* = 7.5 Hz, 1H, phenyl), 6.82 (t, *J* = 7.4 Hz, 1H, py), 6.29 (d, *J* = 7.5 Hz, 1H, py), 4.18 (s, 1H, N*H*), 3.93 (m, 1H, C*H*(CH_3_)_2_), 3.59 (m, 1H, C*H*(CH_3_)_2_), 2.14 (s, 3H, C*H*_3_), 1.92 (s, 30H, CpC*H*_3_), 1.48 (d, *J* = 6.7 Hz, 12H, CH­(C*H*_3_)_2_) ppm. ^13^C­{^1^H} NMR (C_6_D_6_): δ 213.0 (Th*C*), 154.1 (aryl *C*), 144.6 (aryl *C*), 138.6 (aryl *C*), 135.8 (aryl *C*), 132.5 (aryl *C*), 128.8 (aryl *C*), 123.2 (ring *C*), 122.7 (aryl *C*), 122.6 (aryl *C*), 121.9 (aryl *C*), 118.1 (aryl *C*), 29.4 (*C*H­(CH_3_)_2_), 27.7 (*C*H­(CH_3_)_2_), 25.4 (CH­(*C*H_3_)_2_), 24.4 (CH­(*C*H_3_)_2_), 16.9 (*C*H_3_), 11.5 (Cp*C*H_3_) ppm. IR (KBr, cm^–1^): ν 2958 (s), 2901 (s), 2860 (s), 1590 (s), 1457 (s), 1423 (s), 1321 (s), 1241 (s), 1200 (s), 1167 (s), 1112 (s), 841 (s). Anal. Calcd for C_38_H_54_N_2_OTh: C, 58.00; H, 6.92; N, 3.56. Found: C, 57.98; H, 6.94; N, 3.58.

### Method **B**. NMR Scale

A C_6_D_6_ (0.3 mL) solution of 2-MepyNO (2.2 mg, 0.02 mmol) was slowly added to a J. Young NMR tube charged with (η^5^-C_5_Me_5_)_2_Th=N­(dipp)­(dmap) (**4**; 16.0 mg, 0.02 mmol) and C_6_D_6_ (0.2 mL). Resonances of **7** and those of dmap were observed by ^1^H NMR spectroscopy (100% conversion) when this solution was kept at room temperature overnight.

### Preparation of (η^5^-C_5_Me_5_)_2_Th­(NHmesityl)_2_ (**8**). Method **A**

This compound was prepared as colorless crystals from the reaction of (η^5^-C_5_Me_5_)_2_Th=N­(dipp)­(dmap) (**4**; 200 mg, 0.25 mmol) and mesitylNH_2_ (68 mg, 0.50 mmol) in toluene (15 mL) at room temperature and recrystallization from a benzene solution by a similar procedure as that in the synthesis of **6**. The product was isolated by filtration, quickly washed with cooled *n*-hexane (2 mL), and dried at room temperature under vacuum overnight. Yield: 183 mg (95%). ^1^H NMR (C_6_D_6_): δ 6.85 (s, 4H, phenyl), 4.68 (s, 2H, N*H*), 2.37 (s, 12H, C*H*_3_), 2.29 (s, 6H, C*H*_3_), 1.96 (s, 30H, CpC*H*_3_) ppm. These spectroscopic data agreed with those reported in the literature.^7f^ Furthermore, this complex was also characterized by X-ray diffraction analysis and its molecular structure is shown in the Supporting Information (Figure S6).

### Method **B**. NMR Scale

A C_6_D_6_ (0.3 mL) solution of mesitylNH_2_ (5.4 mg, 0.04 mmol) was slowly added to a J. Young NMR tube charged with (η^5^-C_5_Me_5_)_2_Th=N­(dipp)­(dmap) (**4**; 16.0 mg, 0.02 mmol) and C_6_D_6_ (0.2 mL). Resonances of **8** and those of dmap and of dippNH_2_ were observed by ^1^H NMR spectroscopy (100% conversion) when this solution was kept at room temperature overnight.

### Preparation of (η^5^-C_5_Me_5_)_2_Th­[N­(Ph)­N­(Ph)]­(dmap) (**9**). Method **A**

This compound was prepared as yellow crystals from the reaction of (η^5^-C_5_Me_5_)_2_Th = N­(dipp)­(dmap) (**4**; 200 mg, 0.25 mmol) and PhNHNHPh (16 mg, 0.25 mmol) in toluene (15 mL) at 80 °C and recrystallization from a benzene solution by a similar procedure as that in the synthesis of **6**. The product was isolated by filtration, quickly washed with cooled *n*-hexane (2 mL) and dried at room temperature under vacuum overnight. Yield: 182 mg (90%). M.p.: 190–192 °C (dec.). ^1^H NMR (C_6_D_6_): δ 8.17 (d, *J* = 6.7 Hz, 2H, py), 7.30 (m, 5H, phenyl), 6.82 (d, *J* = 7.6 Hz, 2H, phenyl), 6.72 (m, 3H, phenyl). 5.97 (d, *J* = 6.7 Hz, 2H, py), 2.03 (s, 6H, NH­(C*H*_3_)_2_), 1.98 (s, 30H, CpC*H*_3_) ppm. ^13^C­{^1^H} NMR (C_6_D_6_): δ 155.4 (py *C*), 154.9 (phenyl *C*), 154.3 (phenyl *C*), 149.6 (py *C*), 124.0 (ring *C*), 114.2 (phenyl *C*), 113.9 (phenyl *C*), 106.5 (py *C*), 38.2 (NH­(*C*H_3_)_2_), 11.9 (Cp*C*H_3_) ppm. IR (KBr, cm^–1^): ν 2959 (s), 2926 (s), 2858 (s), 1615 (s), 1585 (s), 1468 (s), 1329 (s), 1292 (s), 1243 (s), 1226 (s), 1159 (s), 1003 (s), 984 (s), 873 (s), 812 (s). Anal. Calcd for C_39_H_50_N_4_Th: C, 58.05; H, 6.25; N, 6.94. Found: C, 58.03; H, 6.24; N, 6.96.

### Method **B**. NMR Scale

A C_6_D_6_ (0.3 mL) solution of PhNHNHPh (3.7 mg, 0.02 mmol) was slowly added to a J. Young NMR tube charged with (η^5^-C_5_Me_5_)_2_Th=N­(dipp)­(dmap) (**4**; 16.0 mg, 0.02 mmol) and C_6_D_6_ (0.2 mL). Resonances of **9** and those of dippNH_2_ were observed by ^1^H NMR spectroscopy (100% conversion) when this solution was heated at 80 °C overnight.

### Preparation of (η^5^-C_5_Me_5_)_2_ThCl_2_(dmap) (**10**). Method **A**

This compound was prepared as colorless crystals from the reaction of (η^5^-C_5_Me_5_)_2_Th=N­(dipp)­(dmap) (**4**; 200 mg, 0.25 mmol) and PhSiH_2_Cl (72 mg, 0.50 mmol) in toluene (15 mL) at room temperature and recrystallization from a benzene solution by a similar procedure as that in the synthesis of **6**. The product was isolated by filtration, quickly washed with cooled *n*-hexane (2 mL), and dried at room temperature under vacuum overnight. Yield: 178 mg (92%). M.p.: 170–172 °C (dec.). ^1^H NMR (C_6_D_6_): δ 8.77 (s, 2H, py), 5.78 (d, *J* = 5.6 Hz, 2H, py), 2.29 (s, 30H, CpC*H*_3_), 2.03 (s, 6H, N­(C*H*_3_)_2_) ppm. ^13^C­{^1^H} NMR (C_6_D_6_): δ 154.3 (py *C*), 151.3 (py *C*), 126.2 (ring *C*), 105.7 (py *C*), 38.0 (s, 6H, N­(*C*H_3_)_2_), 12.6 (Cp*C*H_3_) ppm. IR (KBr, cm^–1^): ν 2963 (s), 2933 (s), 2863 (s), 1614 (s), 1532 (s), 1441 (s), 1429 (s), 1384 (s), 1234 (s), 1001 (s), 806 (s). Anal. Calcd for C_33_H_46_N_2_Cl_2_Th: C, 51.23; H, 5.99; N, 3.62. Found: C, 51.21; H, 6.01; N, 3.64.

### Method **B**. NMR Scale

A C_6_D_6_ (0.3 mL) solution of PhSiH_2_Cl (5.7 mg, 0.04 mmol) was slowly added to a J. Young NMR tube charged with (η^5^-C_5_Me_5_)_2_Th=N­(dipp)­(dmap) (**4**; 16.0 mg, 0.02 mmol) and C_6_D_6_ (0.2 mL). Resonances of **10** and those of dippN­(SiH_2_Ph)_2_ ^8b^ were observed by ^1^H NMR spectroscopy (100% conversion) when this solution was kept at room temperature overnight.

### Preparation of (η^5^-C_5_Me_5_)_2_Th­(Cl)­[NH­(2,6-^*i*^Pr_2_-4-(Me_3_Si)­C_6_H_2_)] (**11**). Method **A**

This compound was prepared as colorless crystals from the reaction of (η^5^-C_5_Me_5_)_2_Th=N­(dipp)­(dmap) (**4**; 200 mg, 0.25 mmol) and Me_3_SiCl (28 mg, 0.25 mmol) in toluene (15 mL) at room temperature and recrystallization from a benzene solution by a similar procedure as that in the synthesis of **6**. The product was isolated by filtration, quickly washed with cooled *n*-hexane (2 mL), and dried at room temperature under vacuum overnight. Yield: 161 mg (82%). M.p.: 210–212 °C (dec.). ^1^H NMR (C_6_D_6_): δ 7.45 (br s, 2H, phenyl), 4.71 (s, 1H, N*H*), 3.46 (m, 1H, C*H*(CH_3_)_2_), 2.66 (m, 1H, C*H*(CH_3_)_2_), 1.99 (s, 30H, CpC*H*_3_), 1.43 (s, 12H, CH­(C*H*_3_)_2_), 0.37 (s, 9H, Si­(C*H*_3_)_3_) ppm. ^13^C­{^1^H} NMR (C_6_D_6_): δ 152.9 (phenyl *C*), 133.9 (phenyl *C*), 131.5 (phenyl *C*), 128.3 (phenyl *C*), 126.6 (ring *C*), 35.3 (*C*H­(CH_3_)_2_), 27.9 (*C*H­(CH_3_)_2_), 23.9 (CH­(*C*H_3_)_2_), 22.6 (CH­(*C*H_3_)_2_), 11.6 (Cp*C*H_3_), −0.36 (Si­(*C*H_3_)_3_) ppm. ^29^Si­{^1^H} NMR (C_6_D_6_): δ −5.26 ppm. IR (KBr, cm^–1^): ν 2962 (s), 2929 (s), 2872 (s), 1618 (s), 1584 (s), 1459 (s), 1440 (s), 1382 (s), 1259 (s), 1243 (s), 1158 (s), 1021 (s), 1005 (s), 908 (s), 832 (s). Anal. Calcd for C_35_H_56_NClSiTh: C, 53.46; H, 7.18; N, 1.78. Found: C, 53.43; H, 7.21; N, 1.80.

### Method **B**. NMR Scale

A C_6_D_6_ (0.3 mL) solution of Me_3_SiCl (2.2 mg, 0.02 mmol) was slowly added to a J. Young NMR tube charged with (η^5^-C_5_Me_5_)_2_Th=N­(dipp)­(dmap) (**4**; 16.0 mg, 0.02 mmol) and C_6_D_6_ (0.2 mL). Resonances of **11** and those of dmap were observed by ^1^H NMR spectroscopy (100% conversion) when this solution was kept at room temperature overnight.

### Preparation of (η^5^-C_5_Me_5_)_2_Th­(I)­[NH­(2,6-^*i*^Pr_2_-4-(Me_3_Si)­C_6_H_2_)] (**12**). Method **A**

This compound was prepared as colorless crystals from the reaction of (η^5^-C_5_Me_5_)_2_Th=N­(dipp)­(dmap) (**4**; 200 mg, 0.25 mmol) and Me_3_SiI (43 mg, 0.25 mmol) in toluene (15 mL) at room temperature and recrystallization from a benzene solution by a similar procedure as that in the synthesis of **6**. The product was isolated by filtration, quickly washed with cooled *n*-hexane (2 mL), and dried at room temperature under vacuum overnight. Yield: 189 mg (86%). M.p.: 224–226 °C (dec.). ^1^H NMR (C_6_D_6_): δ 7.49 (s, 1H, phenyl), 7.45 (s, 1H, phenyl), 4.96 (s, 1H, N*H*), 3.13 (m, 1H, C*H*(CH_3_)_2_), 2.92 (m, 1H, C*H*(CH_3_)_2_), 2.04 (s, 30H, CpC*H*_3_), 1.43 (d, *J* = 6.5 Hz, 6H, CH­(C*H*_3_)_2_), 1.38 (d, *J* = 6.8 Hz, 6H, CH­(C*H*_3_)_2_), 0.36 (s, 9H, Si­(C*H*_3_)_3_) ppm. ^13^C­{^1^H} NMR (C_6_D_6_): δ 152.5 (phenyl *C*), 136.1 (phenyl *C*), 134.9 (phenyl *C*), 129.1 (phenyl *C*), 127.4 (ring *C*), 35.2 (*C*H­(CH_3_)_2_), 27.8 (*C*H­(CH_3_)_2_), 24.5 (CH­(*C*H_3_)_2_), 22.9 (CH­(*C*H_3_)_2_), 12.5 (Cp*C*H_3_), −0.39 (Si­(*C*H_3_)_3_) ppm. ^29^Si­{^1^H} NMR (C_6_D_6_): δ −5.16 ppm. IR (KBr, cm^–1^): ν 2959 (s), 2868 (s), 1616 (s), 1584 (s), 1534 (s), 1459 (s), 1323 (s), 1251 (s), 1228 (s), 1159 (s), 1003 (s), 923 (s), 907 (s). Anal. Calcd for C_35_H_56_NISiTh: C, 47.89; H, 6.43; N, 1.60. Found: C, 47.91; H, 6.41; N, 1.62.

### Method **B**. NMR Scale

A C_6_D_6_ (0.3 mL) solution of Me_3_SiI (3.5 mg, 0.02 mmol) was slowly added to a J. Young NMR tube charged with (η^5^-C_5_Me_5_)_2_Th=N­(dipp)­(dmap) (**4**; 16.0 mg, 0.02 mmol) and C_6_D_6_ (0.2 mL). Resonances of **12** and those of dmap were observed by ^1^H NMR spectroscopy (100% conversion) when this solution was kept at room temperature overnight.

### Preparation of (η^5^-C_5_Me_5_)_2_Th­(Br)­[NH­(2,6-^*i*^Pr_2_-4-(Me_5_C_5_)­C_6_H_2_)] (**13**). Method **A**

Solid CuBr (36 mg, 0.25 mmol) was added to a toluene (15 mL) solution of (η^5^-C_5_Me_5_)_2_Th=N­(dipp)­(dmap) (**4**; 200 mg, 0.25 mmol) with stirring at room temperature. After this solution was stirred at room temperature for 2 days, the solvent was removed. The residue was extracted with benzene (10 mL × 3) and filtered. The volume of the filtrate was reduced to 3 mL, colorless crystals of **13** formed when this solution was cooled to 10 °C for 2 days. The product was isolated by filtration, quickly washed with cooled *n*-hexane (2 mL), and dried at room temperature under vacuum overnight. Yield: 76 mg (34%; based on Th). M.p.: 188–190 °C (dec.). ^1^H NMR (C_6_D_6_): δ 6.98 (s, 1H, phenyl), 6.95 (s, 1H, phenyl), 5.00 (s, 1H, N*H*), 3.11 (m, 2H, C*H*(CH_3_)_2_), 2.04 (s, 3H, C*H*_3_), 2.02 (s, 15H, CpC*H*_3_), 2.01 (s, 15H, CpC*H*_3_), 1.83 (s, 6H, C*H*_3_), 1.76 (s, 6H, C*H*_3_), 1.41 (d, *J* = 6.4 Hz, 6H, CH­(C*H*_3_)_2_), 1.32 (d, *J* = 6.4 Hz, 6H, CH­(C*H*_3_)_2_) ppm. ^13^C­{^1^H} NMR (C_6_D_6_): δ 151.2 (phenyl *C*), 148.8 (phenyl *C*), 144.7 (phenyl *C*), 133.4 (phenyl *C*), 126.8 (ring *C*), 126.7 (ring *C*), 120.6 (C=*C*), 120.0 (*C*=C), 60.9 (Ph*C*(Me)), 28.0 (*C*H­(CH_3_)_2_), 27.8 (*C*H­(CH_3_)_2_), 24.4 (CH­(*C*H_3_)_2_), 24.3 (CH­(*C*H_3_)_2_), 19.7 (*C*H_3_), 11.82 (Cp*C*H_3_), 11.78 (Cp*C*H_3_), 11.3 (*C*H_3_), 10.4 (*C*H_3_) ppm. IR (KBr, cm^–1^): ν 2959 (s), 2919 (s), 2863 (s), 1616 (s), 1436 (s), 1379 (s), 1247 (s), 1218 (s), 845 (s). Anal. Calcd for C_42_H_62_NBrTh: C, 56.50; H, 7.00; N, 1.57. Found: C, 56.48; H, 7.02; N, 1.55. After isolation of the colorless crystals of **13**, the solvent of the mother liquid was removed. ^1^H NMR spectroscopy showed the presence of the resonances of **13**, dmap, and other unidentified compounds in the residue.

### Method **B**. NMR Scale

A C_6_D_6_ (0.3 mL) solution of Me_5_C_5_Br^10^ (4.3 mg, 0.02 mmol) was slowly added to a J. Young NMR tube charged with (η^5^-C_5_Me_5_)_2_Th = N­(dipp)­(dmap) (**4**; 16.0 mg, 0.02 mmol) and C_6_D_6_ (0.2 mL). Resonances of **13** and those of dmap were observed by ^1^H NMR spectroscopy (100% conversion) when this solution was kept at room temperature overnight.

### Preparation of [(η^5^-C_5_Me_5_)_2_Th­(I)]_2_[μ-4,4′–(NH–2,6-^*i*^Pr_2_C_6_H_2_)_2_] (**14**)

This compound was prepared as colorless microcrystals from the reaction of (η^5^-C_5_Me_5_)_2_Th=N­(dipp)­(dmap) (**4**; 200 mg, 0.25 mmol) and AgI (59 mg, 0.25 mmol) in toluene (15 mL) at room temperature and recrystallization from a toluene solution by a similar procedure as that in the synthesis of **13**. The product was isolated by filtration, quickly washed with cooled *n*-hexane (2 mL), and dried at room temperature under vacuum overnight. Yield: 129 mg (64%). M.p.: 220–222 °C (dec.). ^1^H NMR (C_6_D_6_): δ 7.68 (d, *J* = 3.4 Hz, 4H, phenyl), 5.09 (s, 2H, N*H*), 3.15 (m, 4H, C*H*(CH_3_)_2_), 2.07 (s, 60H, CpC*H*_3_), 1.47 (d, *J* = 6.4 Hz, 12H, CH­(C*H*_3_)_2_), 1.39 (d, *J* = 6.7 Hz, 12H, CH­(C*H*_3_)_2_) ppm. ^13^C­{^1^H} NMR (C_6_D_6_): δ 149.9 (phenyl), 137.8 (phenyl), 136.3 (phenyl), 134.7 (phenyl), 127.3 (ring *C*), 34.9 (*C*H­(CH_3_)_2_), 28.4 (*C*H­(CH_3_)_2_), 24.5 (CH­(*C*H_3_)_2_), 23.0 (CH­(*C*H_3_)_2_), 12.5 (Cp*C*H_3_) ppm. IR (KBr, cm^–1^): ν 2962 (s), 2924 (s), 2870 (s), 1620(s), 1535 (s), 1442 (s), 1257 (s), 1234 (s), 1002 (s), 802 (s). Anal. Calcd for C_64_H_94_N_2_I_2_Th_2_: C, 47.76; H, 5.89; N, 1.74. Found: C, 47.74; H, 5.91; N, 1.72. After isolation of the colorless microcrystals of **14**, the solvent of the mother liquid was removed. ^1^H NMR spectroscopy showed the presence of the resonances of **14**, dmap, and other unidentified compounds in the residue. Moreover, colorless crystals of **14**·2.5C_6_H_6_ suitable for X-ray structural analysis were isolated from a mixture of toluene and benzene (4:1) solution.

### Preparation of (η^5^-C_5_Me_5_)_2_Th­[SSN­(dipp)­SS] (**15**). Method **A**

This compound was prepared as colorless crystals from the reaction of (η^5^-C_5_Me_5_)_2_Th=N­(dipp)­(dmap) (**4**; 200 mg, 0.25 mmol) and S_8_ (32 mg, 0.125 mmol) in toluene (15 mL) at room temperature and recrystallization from a benzene solution by a similar procedure as that in the synthesis of **13**. The product was isolated by filtration, quickly washed with cooled *n*-hexane (2 mL), and dried at room temperature under vacuum overnight. Yield: 165 mg (82%). M.p.: 224–226 °C (dec.). ^1^H NMR (C_6_D_6_): δ 7.08 (t, *J* = 7.5 Hz, 1H, phenyl), 7.02 (d, *J* = 7.5 Hz, 2H, phenyl), 3.49 (m, 2H, C*H*(CH_3_)_2_), 2.12 (s, 30H, CpC*H*_3_), 1.41 (d, *J* = 6.7 Hz, 6H, CH­(C*H*_3_)_2_), 1.27 (d, *J* = 6.9 Hz, 6H, CH­(C*H*_3_)_2_) ppm. ^13^C­{^1^H} NMR (C_6_D_6_): δ 147.9 (phenyl *C*), 147.6 (phenyl *C*), 129.0 (phenyl *C*), 125.6 (ring *C*), 124.3 (phenyl *C*), 30.1 (*C*H­(CH_3_)_2_), 25.4 (CH­(*C*H_3_)_2_), 24.8 (CH­(*C*H_3_)_2_), 12.1 (Cp*C*H_3_) ppm. IR (KBr, cm^–1^): ν 2962 (s), 2908 (s), 2865 (s), 1606 (s), 1440 (s), 1378 (s), 1183 (s), 1100 (s), 1019 (s), 791 (s). Anal. Calcd for C_32_H_47_NS_4_Th: C, 47.68; H, 5.88; N, 1.74. Found: C, 47.66; H, 5.89; N, 1.77.

### Method **B**. NMR Scale

Solid S_8_ (2.6 mg, 0.01 mmol) was added to a J. Young NMR tube charged with (η^5^-C_5_Me_5_)_2_Th=N­(dipp)­(dmap) (**4**; 16.0 mg, 0.02 mmol) and C_6_D_6_ (0.5 mL). Resonances of **10** and those of dmap were observed by ^1^H NMR spectroscopy (100% conversion) when this solution was kept at room temperature for 2 days.

### Preparation of (η^5^-C_5_Me_5_)_2_Th­[N­(dipp)­C­(Me)=CHPh]­(κ^2^-*C*,*N*-4-Me_2_NC_5_H_3_N) (**16**). Method **A**

This compound was prepared as yellow crystals from the reaction of (η^5^-C_5_Me_5_)_2_Th=N­(dipp)­(dmap) (**4**; 200 mg, 0.25 mmol) and PhC≡CMe (29 mg, 0.25 mmol) in toluene (15 mL) at 120 °C and recrystallization from a benzene solution by a similar procedure as that in the synthesis of **6**. The product was isolated by filtration, quickly washed with cooled *n*-hexane (2 mL), and dried at room temperature under vacuum overnight. Yield: 193 mg (84%). M.p.: 260–262 °C (dec.). ^1^H NMR (C_6_D_6_): δ 7.65 (d, *J* = 7.6 Hz, 2H, phenyl), 7.39 (t, *J* = 7.5 Hz, 2H, phenyl), 7.18 (m, 2H, phenyl and py), 7.15 (s, 2H, phenyl), 7.06 (t, *J* = 7.3 Hz, 1H, phenyl), 6.55 (d, *J* = 6.1 Hz, 1H, py), 6.40 (s, 1H, C=C*H*), 5.81 (d, *J* = 4.8 Hz, 1H, py), 3.28 (m, 2H, C*H*(CH_3_)_2_), 2.31 (s, 6H, NH­(C*H*_3_)_2_), 2.12 (s, 30H, CpC*H*_3_), 2.10 (s, 3H, C*H*_3_), 1.16 (d, *J* = 6.5 Hz, 6H, CH­(C*H*_3_)_2_), 0.95 (d, *J* = 6.4 Hz, 6H, CH­(C*H*_3_)_2_) ppm. ^13^C­{^1^H} NMR (C_6_D_6_): δ 230.4 (Th*C*), 159.2 (aryl *C*), 154.6 (aryl *C*), 151.2 (aryl *C*), 145.2 (aryl *C*), 144.1 (aryl *C*), 142.2 (aryl *C*), 136.3 (aryl *C*), 131.8 (aryl *C*), 128.3 (aryl *C*), 124.2 (ring *C*), 123.8 (aryl *C*), 122.5 (aryl *C*), 109.2 (aryl *C*), 107.6 (*C*=CH), 97.5 (C=*C*H), 38.7 (N*C*H_3_), 28.2 (*C*H­(CH_3_)_2_), 26.3 (*C*H­(CH_3_)_2_), 24.8 (CH­(*C*H_3_)_2_), 23.4 (CH­(*C*H_3_)_2_), 21.7 (C=C*C*H3), 12.7 (Cp*C*H_3_) ppm. IR (KBr, cm^–1^): ν 2958 (s), 2928 (s), 2861 (s), 1619 (s), 1527 (s), 1433 (s), 1259 (s), 1193 (s), 1108 (s), 1001 (s), 808 (s). Anal. Calcd for C_48_H_65_N_3_Th: C, 62.93; H, 7.15; N, 4.59. Found: C, 62.91; H, 7.17; N, 4.62.

### Method **B**. NMR Scale

A C_6_D_6_ (0.3 mL) solution of PhC≡CMe (2.4 mg, 0.02 mmol) was slowly added to a J. Young NMR tube charged with (η^5^-C_5_Me_5_)_2_Th = N­(dipp)­(dmap) (**4**; 16.0 mg, 0.02 mmol) and C_6_D_6_ (0.2 mL). Resonances of **16** were observed by ^1^H NMR spectroscopy (100% conversion) when this solution was heated to 120 °C overnight.

### Preparation of (η^5^-C_5_Me_5_)_2_Th­[N­(dipp)­C­(C_2_Ph)=CHPh]­(κ^2^-*C*,*N*-4-Me_2_NC_5_H_3_N) (**17**). Method **A**

This compound was prepared as yellow crystals from the reaction of (η^5^-C_5_Me_5_)_2_Th=N­(dipp)­(dmap) (**4**; 200 mg, 0.25 mmol) and PhC≡C–C≡CPh (51 mg, 0.25 mmol) in toluene (15 mL) at 80 °C and recrystallization from a benzene solution by a similar procedure as that in the synthesis of **6**. The product was isolated by filtration, quickly washed with cooled *n*-hexane (2 mL), and dried at room temperature under vacuum overnight. Yield: 205 mg (82%). M.p.: 178–180 °C (dec.). ^1^H NMR (C_6_D_6_): δ 8.34 (d, *J* = 7.8 Hz, 2H, phenyl), 7.45 (t, *J* = 7.5 Hz, 2H, phenyl), 7.27 (m, 3H, phenyl), 7.19 (t, *J* = 8.0 Hz, 1H, phenyl), 7.13 (m, 3H, phenyl), 6.89 (t, *J* = 7.5 Hz, 2H, phenyl), 6.84 (d, *J* = 7.2 Hz, 1H, py), 6.75 (d, *J* = 6.2 Hz, 1H, py), 6.70 (s, 1H, C*H*=C), 5.80 (m, 1H, py), 3.36 (m, 2H, C*H*(CH_3_)_2_), 2.29 (s, 6H, N­(C*H*_3_)_2_), 2.17 (s, 30H, CpC*H*_3_), 1.46 (d, *J* = 6.5 Hz, 6H, CH­(C*H*_3_)_2_), 0.99 (d, *J* = 6.4 Hz, 6H, CH­(C*H*_3_)_2_) ppm. ^13^C­{^1^H} NMR (C_6_D_6_): δ 229.8 (Th*C*), 154.8 (aryl *C*), 152.0 (aryl *C*), 146.0 (aryl *C*), 144.1 (aryl *C*), 142.2 (aryl *C*), 140.5 (aryl *C*), 131.9 (aryl *C*), 129.9 (aryl *C*), 128.6 (aryl *C*), 128.5 (aryl *C*), 128.3 (aryl *C*), 124.6 (ring *C*), 124.4 (aryl *C*), 124.2 (aryl *C*), 123.8 (aryl *C*), 123.7 (aryl *C*), 109.1 (aryl *C*), 107.8 (*C*≡CPh), 104.9 (C≡*C*Ph), 96.2 (*C*=CH), 91.3 (C=*C*H), 38.7 (N­(*C*H_3_)_2_), 28.8 (*C*H­(CH_3_)_2_), 27.6 (*C*H­(CH_3_)_2_), 24.8 (CH­(*C*H_3_)_2_), 12.7 (Cp*C*H_3_) ppm. IR (KBr, cm^–1^): ν 2960 (s), 2926 (s), 2864 (s), 1611(s), 1579 (s), 1537 (s), 1431 (s), 1256 (s), 994 (s), 804 (s). Anal. Calcd for C_55_H_67_N_3_Th: C, 65.92; H, 6.74; N, 4.19. Found: C, 65.91; H, 6.73; N, 4.21.

### Method **B**. NMR Scale

A C_6_D_6_ (0.3 mL) solution of PhC≡C–C≡CPh (4.1 mg, 0.02 mmol) was slowly added to a J. Young NMR tube charged with (η^5^-C_5_Me_5_)_2_Th = N­(dipp)­(dmap) (**4**; 16.0 mg, 0.02 mmol) and C_6_D_6_ (0.2 mL). Resonances of **17** were observed by ^1^H NMR spectroscopy (100% conversion) when this solution was heated to 80 °C overnight.

### Preparation of (η^5^-C_5_Me_5_)_2_Th­(NHdipp)­(C≡CPh) (**18**). Method **A**

This compound was prepared as colorless crystals from the reaction of (η^5^-C_5_Me_5_)_2_Th=N­(dipp)­(dmap) (**4**; 200 mg, 0.25 mmol) and PhC≡CH (26 mg, 0.25 mmol) in toluene (15 mL) at room temperature and recrystallization from a benzene solution by a similar procedure as that in the synthesis of **6**. The product was isolated by filtration, quickly washed with cooled *n*-hexane (2 mL), and dried at room temperature under vacuum overnight. Yield: 172 mg (88%). M.p.: 120–122 °C (dec.). ^1^H NMR (C_6_D_6_): δ 7.66 (m, 2H, phenyl), 7.12 (m, 4H, phenyl), 7.03 (m, 1H, phenyl), 6.83 (t, *J* = 7.5 Hz, 1H, phenyl), 4.39 (s, 1H, N*H*), 2.42 (s, 2H, C*H*(CH_3_)_2_), 2.09 (s, 30H, CpC*H*_3_), 1.41 (d, *J* = 4.7 Hz, 12H, CH­(C*H*_3_)_2_) ppm. ^13^C­{^1^H} NMR (C_6_D_6_): δ 181.4 (Th*C*≡C), 152.5 (phenyl *C*), 135.0 (phenyl *C*), 131.9 (phenyl *C*), 128.5 (phenyl *C*), 127.1 (phenyl *C*), 125.4 (ring *C*), 123.0 (phenyl *C*), 122.9 (phenyl *C*), 118.7 (phenyl *C*), 109.3 (ThC≡*C*), 28.1 (*C*H­(CH_3_)_2_), 23.5 (CH­(*C*H_3_)_2_), 22.6 (CH­(*C*H_3_)_2_), 11.6 (Cp*C*H_3_) ppm. IR (KBr, cm^–1^): ν 2963 (s), 1602 (s), 1423 (s), 1261 (s), 1095 (s), 1021 (s), 804 (s). Anal. Calcd for C_40_H_53_NTh: C, 61.60; H, 6.85; N, 1.80. Found: C, 61.61; H, 6.83; N, 1.82.

### Method **B**. NMR Scale

A C_6_D_6_ (0.3 mL) solution of PhC≡CH (2.1 mg, 0.02 mmol) was slowly added to a J. Young NMR tube charged with (η^5^-C_5_Me_5_)_2_Th = N­(dipp)­(dmap) (**4**; 16.0 mg, 0.02 mmol) and C_6_D_6_ (0.2 mL). Resonances of **18** and those of dmap were observed by ^1^H NMR spectroscopy (100% conversion) when this solution was kept at room temperature overnight.

### Preparation of (η^5^-C_5_Me_5_)_2_Th­[N­(dipp)­C­(=N^*i*^Pr)­N­(^*i*^Pr)]­(dmap) (**19**). Method **A**

This compound was prepared as colorless crystals from the reaction of (η^5^-C_5_Me_5_)_2_Th=N­(dipp)­(dmap) (**4**; 200 mg, 0.25 mmol) and (^*i*^PrN=)_2_C (32 mg, 0.25 mmol) in toluene (15 mL) at 80 °C and recrystallization from a benzene solution by a similar procedure as that in the synthesis of **6**. The product was isolated by filtration, quickly washed with cooled *n*-hexane (2 mL), and dried at room temperature under vacuum overnight. Yield: 199 mg (86%). M.p.: 268–270 °C (dec.). ^1^H NMR (C_6_D_6_): δ 8.45 (d, *J* = 5.0 Hz, 2H, py), 7.26 (d, *J* = 7.6 Hz, 2H, phenyl), 7.09 (t, *J* = 7.5 Hz, 1H, phenyl), 6.10 (d, *J* = 5.5 Hz, 2H, py), 4.30 (m, 2H, NC*H*(CH_3_)_2_)), 3.53 (m, 2H, C*H*(CH_3_)_2_), 2.23 (s, 6H, N­(C*H*_3_)_2_), 1.99 (s, 30H, CpC*H*_3_), 1.46 (d, *J* = 6.8 Hz, 12H, NCH­(C*H*_3_)_2_), 1.27 (d, *J* = 6.2 Hz, 12H, CH­(C*H*_3_)_2_) ppm. ^13^C­{^1^H} NMR (C_6_D_6_): δ 154.1 (py *C*), 150.5 (py *C*), 147.6 (phenyl *C*), 144.3 (phenyl *C*), 138.9 (phenyl *C*), 126.8 (ring *C*), 121.8 (phenyl *C*), 119.6 (*C*=N), 106.8 (py *C*), 46.3 (N*C*H­(CH_3_)_2_), 38.3 (N­(*C*H_3_)_2_), 29.1 (NCH­(*C*H_3_)_2_), 25.7 (*C*H­(CH_3_)_2_), 23.5 (CH­(*C*H_3_)_2_), 11.8 (Cp*C*H_3_) ppm. IR (KBr, cm^–1^): ν 2955 (s), 2921 (s), 2864 (s), 1616 (s), 1539 (s), 1445 (s), 1428 (s), 1378 (s), 1229 (s), 1002 (s), 987 (s), 958 (s), 802 (s). Anal. Calcd for C_46_H_71_N_5_Th: C, 59.66; H, 7.73; N, 7.56. Found: C, 59.64; H, 7.71; N, 7.58.

### Method **B**. NMR Scale

A C_6_D_6_ (0.3 mL) solution of (^*i*^PrN=)_2_C (2.5 mg, 0.02 mmol) was slowly added to a J. Young NMR tube charged with (η^5^-C_5_Me_5_)_2_Th=N­(dipp)­(dmap) (**4**; 16.0 mg, 0.02 mmol) and C_6_D_6_ (0.2 mL). Resonances of **19** were observed by ^1^H NMR spectroscopy (100% conversion) when this solution was kept at 80 °C overnight.

### Preparation of [(η^5^-C_5_Me_5_)_2_Th­(dmap)]_2_(μ-O)_2_ (**20**). Method **A**

This compound was prepared as colorless crystals from the reaction of (η^5^-C_5_Me_5_)_2_Th=N­(dipp)­(dmap) (**4**; 200 mg, 0.25 mmol) and *p*-tolylCHO (30 mg, 0.25 mmol) in toluene (15 mL) at room temperature and recrystallization from a benzene solution by a similar procedure as that in the synthesis of **6**. The product was isolated by filtration, quickly washed with cooled *n*-hexane (2 mL), and dried at room temperature under vacuum overnight. Yield: 141 mg (88%). M.p.: 204–206 °C (dec.). ^1^H NMR (C_6_D_6_): δ 9.30 (s, 4H, py), 6.33 (d, *J* = 6.3 Hz, 4H, py), 2.32 (s, 60H, CpC*H*_3_), 2.09 (s, 12H, NH­(C*H*_3_)_2_) ppm. ^13^C­{^1^H} NMR (C_6_D_6_): δ 154.5 (py *C*), 152.8 (py *C*), 120.6 (ring *C*), 105.5 (py *C*), 38.1 (NH­(*C*H_3_)_2_), 13.6 (Cp*C*H_3_) ppm. IR (KBr, cm^–1^): ν 2960 (s), 2924 (s), 1613 (s), 1534 (s), 1384 (s), 1260 (s), 1230 (s), 1001 (s), 810 (s). Anal. Calcd for C_54_H_80_N_4_O_2_Th_2_: C, 50.62; H, 6.29; N, 4.37. Found: C, 50.64; H, 6.31; N, 4.38.

### Method **B**. NMR Scale

A C_6_D_6_ (0.3 mL) solution of *p*-tolylCHO (2.5 mg, 0.02 mmol) was slowly added to a J. Young NMR tube charged with (η^5^-C_5_Me_5_)_2_Th=N­(dipp)­(dmap) (**4**; 16.0 mg, 0.02 mmol) and C_6_D_6_ (0.2 mL). Resonances of **20** and those of *p*-tolylCH=Ndipp were observed by ^1^H NMR spectroscopy (100% conversion) when this solution was kept at room temperature overnight.

### Preparation of (η^5^-C_5_Me_5_)_2_Th­[NH­(dipp)]­(κ-*O*-1-OC_6_H_9_) (**21**). Method **A**

This compound was prepared as colorless crystals from the reaction of (η^5^-C_5_Me_5_)_2_Th=N­(dipp)­(dmap) (**4**; 200 mg, 0.25 mmol) and cyclohexanone (25 mg, 0.25 mmol) in toluene (15 mL) at room temperature and recrystallization from a benzene solution by a similar procedure as that in the synthesis of **6**. The product was isolated by filtration, quickly washed with cooled *n*-hexane (2 mL), and dried at room temperature under vacuum overnight. Yield: 159 mg (82%). M.p.: 116–118 °C (dec.). ^1^H NMR (C_6_D_6_): δ 7.20 (s, 2H, phenyl), 6.93 (t, *J* = 7.6 Hz, 1H, phenyl), 4.98 (s, 1H, N*H*), 4.37 (s, 1H, C*H*), 3.21 (s, 2H, C*H*(CH_3_)_2_), 2.24 (d, *J* = 3.5 Hz, 2H, C*H*_2_), 2.14 (m, 2H, C*H*_2_), 2.01 (s, 30H, CpC*H*_3_), 1.71 (m, 2H, C*H*_2_), 1.56 (m, 2H, C*H*_2_), 1.42 (s, 6H, CH­(C*H*_3_)_2_), 1.38 (s, 6H, CH­(C*H*_3_)_2_) ppm. ^13^C­{^1^H} NMR (C_6_D_6_): δ 157.8 (O*C*), 151.8 (phenyl *C*), 128.3 (phenyl *C*), 124.8 (ring *C*), 123.1 (phenyl *C*), 118.6 (phenyl *C*), 102.9 (OC=*C*), 32.7 (*C*H­(CH_3_)_2_), 27.8 (CH­(*C*H_3_)_2_), 24.6 (Cy *C*), 24.4 (Cy *C*), 24.0 (Cy *C*), 22.5 (Cy *C*), 11.2 (Cp*C*H_3_) ppm. IR (KBr, cm^–1^): ν 2958 (s), 2923 (s), 2862 (s), 1651(s), 1600 (s), 1428 (s), 1252 (s), 1184 (s), 797 (s). Anal. Calcd for C_38_H_57_NOTh: C, 58.82; H, 7.40; N, 1.81. Found: C, 58.84; H, 7.41; N, 1.79.

### Method **B**. NMR Scale

A C_6_D_6_ (0.3 mL) solution of cyclohexanone (2.0 mg, 0.02 mmol) was slowly added to a J. Young NMR tube charged with (η^5^-C_5_Me_5_)_2_Th=N­(dipp)­(dmap) (**4**; 16.0 mg, 0.02 mmol) and C_6_D_6_ (0.2 mL). Resonances of **21** and those of dmap were observed by ^1^H NMR spectroscopy (100% conversion) when this solution was kept at room temperature overnight.

### Preparation of (η^5^-C_5_Me_5_)_2_Th­[SC=N­(dipp)-S]­(dmap) (**22**)

A benzene (5 mL) solution of CS_2_ (19 mg, 0.25 mmol) was added to a benzene (10 mL) solution of (η^5^-C_5_Me_5_)_2_Th=N­(dipp)­(dmap) (**4**; 200 mg, 0.25 mmol) without stirring at room temperature. After the solution was stored at room temperature overnight without stirring, colorless crystals were isolated from the solution by filtration. The product was washed with *n*-hexane and dried at room temperature under vacuum overnight, and the product was identified as **22** by X-ray diffraction analysis. Yield: 206 mg (94%). M.p.: 246–248 °C (dec.). IR (KBr, cm^–1^): ν 2963 (s), 2927 (s), 1604 (s), 1478 (s), 1383 (s), 1260 (s), 1099 (s), 1012 (s), 801 (s). Anal. Calcd for C_40_H_57_N_3_S_2_Th: C, 54.84; H, 6.56; N, 4.80. Found: C, 54.82; H, 6.57; N, 4.82. This compound was insoluble in common (deuterated) solvents such as pyridine, THF, toluene, and CD_2_Cl_2_, precluding its characterization by NMR spectroscopy.

### Preparation of (η^5^-C_5_Me_5_)_2_Th­[OC­(Ph)­N­(Ph)]_2_ (**23**). Method **A**

This compound was prepared as colorless crystals from the reaction of (η^5^-C_5_Me_5_)_2_Th=N­(dipp)­(dmap) (**4**; 200 mg, 0.25 mmol) and PhCONHPh (99 mg, 0.50 mmol) in toluene (15 mL) at room temperature and recrystallization from a benzene solution by a similar procedure as that in the synthesis of **6**. The product was isolated by filtration, quickly washed with cooled *n*-hexane (2 mL), and dried at room temperature under vacuum overnight. Yield: 188 mg (84%). M.p.: 146–148 °C (dec.). ^1^H NMR (C_6_D_6_): δ 7.57 (d, *J* = 7.7 Hz, 4H, phenyl), 6.94 (m, 6H, phenyl), 6.67 (m, 6H, phenyl), 6.57 (m, 4H, phenyl), 2.22 (s, 30H, CpC*H*_3_) ppm. ^13^C­{^1^H} NMR (C_6_D_6_): δ 174.3 (O*C*), 147.8 (phenyl *C*), 136.5 (phenyl *C*), 130.3 (phenyl *C*), 130.0 (phenyl *C*), 128.5 (phenyl *C*), 128.3 (phenyl *C*), 125.2 (phenyl *C*), 123.8 (ring *C*), 123.2 (phenyl *C*), 12.1 (Cp*C*H_3_) ppm. IR (KBr, cm^–1^): ν 2905 (s), 2859 (s), 1660 (s), 1585 (s), 1509 (s), 1487 (s), 1413 (s), 1252 (s), 1122 (s), 1026 (s), 922 (s). Anal. Calcd for C_46_H_50_N_2_O_2_Th: C, 61.74; H, 5.63; N, 3.13. Found: C, 61.72; H, 5.61; N, 3.15.

### Method **B**. NMR Scale

A C_6_D_6_ (0.3 mL) solution of PhCONHPh (7.9 mg, 0.04 mmol) was slowly added to a J. Young NMR tube charged with (η^5^-C_5_Me_5_)_2_Th=N­(dipp)­(dmap) (**4**; 16.0 mg, 0.02 mmol) and C_6_D_6_ (0.2 mL). Resonances of **23** and those of dmap and dippNH_2_ were observed by ^1^H NMR spectroscopy (100% conversion) when this solution was kept at room temperature overnight.

### Preparation of (η^5^-C_5_Me_5_)_2_Th­(N=CPh_2_)_2_ (**24**). Method **A**

This compound was prepared as orange crystals from the reaction of (η^5^-C_5_Me_5_)_2_Th=N­(dipp)­(dmap) (**4**; 200 mg, 0.25 mmol) and Ph_2_C=NH (91 mg, 0.50 mmol) in toluene (15 mL) at room temperature and recrystallization from a benzene solution by a similar procedure as that in the synthesis of **6**. The product was isolated by filtration, quickly washed with cooled *n*-hexane (2 mL), and dried at room temperature under vacuum overnight. Yield: 186 mg (86%). M.p.: 178–180 °C (dec.). ^1^H NMR (C_6_D_6_): δ 7.64 (d, *J* = 6.8 Hz, 8H, phenyl), 7.16 (m, 12H, phenyl), 1.96 (s, 30H, CpC*H*_3_) ppm. ^13^C­{^1^H} NMR (C_6_D_6_): δ 173.3 (N=*C*), 143.8 (phenyl *C*), 128.8 (phenyl *C*), 128.7 (phenyl *C*), 128.1 (phenyl *C*), 123.3 (ring *C*), 11.3 (Cp*C*H_3_) ppm. IR (KBr, cm^–1^): ν 2892 (s), 2855 (s), 1618 (s), 1573 (s), 1443 (s), 1366 (s), 1245 (s), 927 (s), 898 (s), 776 (s). Anal. Calcd for C_46_H_50_N_2_Th: C, 64.02; H, 5.84; N, 3.25. Found: C, 64.05; H, 5.81; N, 3.23.

### Method **B**. NMR Scale

A C_6_D_6_ (0.3 mL) solution of Ph_2_C=NH (7.3 mg, 0.04 mmol) was slowly added to a J. Young NMR tube charged with (η^5^-C_5_Me_5_)_2_Th=N­(dipp)­(dmap) (**4**; 16.0 mg, 0.02 mmol) and C_6_D_6_ (0.2 mL). Resonances of **24** and those of dmap and dippNH_2_ were observed by ^1^H NMR spectroscopy (100% conversion) when this solution was kept at room temperature overnight.

### Preparation of (η^5^-C_5_Me_5_)_2_Th­[=NC­(Ph)=N­(dipp)]­(dmap) (**25**). Method **A**

This compound was prepared as yellow crystals from the reaction of (η^5^-C_5_Me_5_)_2_Th=N­(dipp)­(dmap) (**4**; 200 mg, 0.25 mmol) and PhCN (26 mg, 0.25 mmol) in toluene (15 mL) at room temperature and recrystallization from a benzene solution by a similar procedure as that in the synthesis of **6**. The product was isolated by filtration, quickly washed with cooled *n*-hexane (2 mL), and dried at room temperature under vacuum overnight. Yield: 190 mg (84%). M.p.: 148–150 °C (dec.). ^1^H NMR (C_6_D_6_): δ 8.86 (d, *J* = 6.4 Hz, 1H, phenyl), 8.50 (d, *J* = 6.9 Hz, 2H, py), 7.50 (t, *J* = 7.4 Hz, 2H, phenyl), 7.37 (m, 4H, phenyl), 7.10 (d, *J* = 7.9 Hz, 1H, phenyl), 6.05 (s, 2H, py), 3.79 (m, 2H, C*H*(CH_3_)_2_), 2.13 (s, 6H, N­(C*H*_3_)_2_), 2.01 (s, 30H, CpC*H*_3_), 1.57 (d, *J* = 6.5 Hz, 6H, CH­(C*H*_3_)_2_), 1.49 (d, *J* = 6.8 Hz, 6H, CH­(C*H*_3_)_2_) ppm. ^13^C­{^1^H} NMR (C_6_D_6_): δ 153.9 (py *C*), 145.1 (py *C*), 142.0 (phenyl *C*), 139.4 (phenyl *C*), 132.6 (phenyl *C*), 129.4 (phenyl *C*), 129.3 (phenyl *C*), 128.8 (phenyl *C*), 127.3 (ring *C*), 123.4 (phenyl *C*), 122.4 (phenyl *C*), 121.9 (N=*C*), 107.4 (py *C*), 38.6 (N­(*C*H_3_)_2_), 29.1 (*C*H­(CH_3_)_2_), 26.0 (*C*H­(CH_3_)_2_), 24.0 (CH­(*C*H_3_)_2_), 22.6 (CH­(*C*H_3_)_2_), 11.7 (Cp*C*H_3_) ppm. IR (KBr, cm^–1^): ν 2959 (s), 2916 (s), 2863 (s), 1604 (s), 1524 (s), 1370 (s), 1256 (s), 1226 (s), 994 (s), 804 (s). Anal. Calcd for C_46_H_62_N_4_Th: C, 61.18; H, 6.92; N, 6.20. Found: C, 61.15; H, 6.91; N, 6.23.

### Method **B**. NMR Scale

A C_6_D_6_ (0.3 mL) solution of PhCN (2.1 mg, 0.02 mmol) was slowly added to a J. Young NMR tube charged with (η^5^-C_5_Me_5_)_2_Th=N­(dipp)­(dmap) (**4**; 16.0 mg, 0.02 mmol) and C_6_D_6_ (0.2 mL). Resonances of **25** were observed by ^1^H NMR spectroscopy (100% conversion) when this solution was kept at room temperature overnight.

### Preparation of (η^5^-C_5_Me_5_)_2_Th­[NHC­(CH_2_Ph)=N­(dipp)]­(N=C=CHPh) (**26**). Method **A**

This compound was prepared as yellow crystals from the reaction of (η^5^-C_5_Me_5_)_2_Th=N­(dipp)­(dmap) (**4**; 200 mg, 0.25 mmol) and PhCH_2_CN (59 mg, 0.50 mmol) in toluene (15 mL) at room temperature and recrystallization from a benzene solution by a similar procedure as that in the synthesis of **6**. The product was isolated by filtration, quickly washed with cooled *n*-hexane (2 mL), and dried at room temperature under vacuum overnight. Yield: 187 mg (82%). M.p.: 266–268 °C (dec.). ^1^H NMR (C_6_D_6_): δ 7.21 (d, *J* = 1.4 Hz, 1H, phenyl), 7.12 (m, 6H, phenyl), 7.06 (d, *J* = 7.3 Hz, 1H, phenyl), 7.01 (d, *J* = 7.1 Hz, 2H, phenyl), 6.85 (d, *J* = 7.4 Hz, 2H, phenyl), 6.78 (t, *J* = 7.3 Hz, 1H, phenyl), 5.69 (s, 1H, C=C*H*), 3.53 (s, 1H, N*H*), 3.35 (m, 2H, C*H*(CH_3_)_2_), 3.29 (s, 2H, C*H*_2_), 1.99 (s, 30H, CpC*H*_3_), 1.49 (d, *J* = 6.6 Hz, 6H, CH­(C*H*_3_)_2_), 1.27 (d, *J* = 6.7 Hz, 6H, CH­(C*H*_3_)_2_) ppm. ^13^C­{^1^H} NMR (C_6_D_6_): δ 176.9 (N*C*N), 163.6 (*C*=N), 143.0 (phenyl *C*), 142.8 (phenyl *C*), 141.8 (phenyl *C*), 134.7 (phenyl *C*), 130.5 (phenyl *C*), 129.1 (phenyl *C*), 128.7 (phenyl *C*), 128.3 (phenyl *C*), 125.8 (phenyl *C*), 125.12 (ring *C*), 125.05 (phenyl *C*), 122.5 (phenyl *C*), 122.3 (phenyl *C*), 118.7 (H*C*=C), 42.2 (*C*H_2_), 36.9 (*C*H­(CH_3_)_2_), 28.2 (*C*H­(CH_3_)_2_), 26.9 (CH­(*C*H_3_)_2_), 26.0 (CH­(*C*H_3_)_2_), 11.6 (Cp*C*H_3_) ppm. IR (KBr, cm^–1^): ν 3034 (s), 2956 (s), 2923 (s), 2862 (s), 2045 (s; N=C=C), 1608 (s), 1479 (s), 1257 (s), 1228 (s), 1001 (s), 804 (s). Anal. Calcd for C_48_H_61_N_3_Th: C, 63.21; H, 6.74; N, 4.61. Found: C, 63.23; H, 6.72; N, 4.63.

### Method **B**. NMR Scale

A C_6_D_6_ (0.3 mL) solution of PhCH_2_CN (4.7 mg, 0.04 mmol) was slowly added to a J. Young NMR tube charged with (η^5^-C_5_Me_5_)_2_Th=N­(dipp)­(dmap) (**4**; 16.0 mg, 0.02 mmol) and C_6_D_6_ (0.2 mL). Resonances of **26** and those of dmap were observed by ^1^H NMR spectroscopy (100% conversion) when this solution was kept at room temperature overnight.

### Preparation of (η^5^-C_5_Me_5_)_2_Th­[NHC­(C_6_H_11_)=N­(dipp)]­[N=C=C­(CH_2_)_5_] (**27**). Method **A**

This compound was prepared as yellow crystals from the reaction of (η^5^-C_5_Me_5_)_2_Th=N­(dipp)­(dmap) (**4**; 200 mg, 0.25 mmol) and C_6_H_11_CN (55 mg, 0.50 mmol) in toluene (15 mL) at 80 °C and recrystallization from a benzene solution by a similar procedure as that in the synthesis of **6**. The product was isolated by filtration, quickly washed with cooled *n*-hexane (2 mL), and dried at room temperature under vacuum overnight. Yield: 175 mg (78%). M.p.: 170–172 °C (dec.). ^1^H NMR (C_6_D_6_): δ 7.15 (d, *J* = 7.4 Hz, 2H, phenyl), 7.06 (d, *J* = 7.5 Hz, 1H, phenyl), 5.48 (s, 1H, N*H*), 3.17 (m, 2H, C*H*(CH_3_)_2_), 2.11 (s, 30H, CpC*H*_3_), 1.76 (m, 2H, Cy), 1.60 (m, 4H, Cy), 1.56 (m, 1H, Cy), 1.53 (m, 2H, Cy), 1.48 (d, *J* = 6.6 Hz, 6H, CH­(C*H*_3_)_2_), 1.37 (m, 6H, Cy), 1.22 (d, *J* = 6.6 Hz, 6H, CH­(C*H*_3_)_2_), 1.12 (m, 4H, Cy), 0.82 (m, 2H, Cy) ppm. ^13^C­{^1^H} NMR (C_6_D_6_): δ 182.3 (N=*C*), 176.0 (HN*C*), 143.1 (phenyl), 142.5 (phenyl), 128.3 (phenyl), 124.3 (phenyl), 123.9 (ring *C*), 121.7 (NC=*C*), 40.9 (Cy *C*), 35.5 (*C*H­(CH_3_)_2_), 31.9 (*C*H­(CH_3_)_2_), 28.8 (Cy *C*), 28.2 (Cy *C*), 27.6 (Cy *C*), 27.4 (Cy *C*), 26.6 (Cy *C*), 26.5 (Cy *C*), 26.0 (CH­(*C*H_3_)_2_), 25.7 (CH­(*C*H_3_)_2_), 11.8 (Cp*C*H_3_) ppm. IR (KBr, cm^–1^): ν 2926 (s), 2856 (s), 2025 (s; C=C=N), 1604 (s), 1487 (s), 1427 (s), 1255 (s), 1097 (s), 1026 (s), 804 (s). Anal. Calcd for C_46_H_69_N_3_Th: C, 61.66; H, 7.76; N, 4.69. Found: C, 61.64; H, 7.78; N, 4.71.

### Method **B**. NMR Scale

A C_6_D_6_ (0.3 mL) solution of C_6_H_11_CN (4.4 mg, 0.04 mmol) was slowly added to a J. Young NMR tube charged with (η^5^-C_5_Me_5_)_2_Th=N­(dipp)­(dmap) (**4**; 16.0 mg, 0.02 mmol) and C_6_D_6_ (0.2 mL). Resonances of **27** and those of dmap were observed by ^1^H NMR spectroscopy (100% conversion) when this solution was heated to 80 °C overnight.

### Preparation of (η^5^-C_5_Me_5_)_2_Th­{NH­[C=C­(CN)­(CH_2_)_3_]}]­{NH­[C=NC­(=Ndipp)­(CH_2_)_4_]} (**28**)

This compound was prepared as colorless crystals from the reaction of (η^5^-C_5_Me_5_)_2_Th=N­(dipp)­(dmap) (**4**; 200 mg, 0.25 mmol) and 1,4-(CH_2_)_4_(CN)_2_ (54 mg, 0.50 mmol) in toluene (15 mL) at room temperature and recrystallization from a benzene solution by a similar procedure as that in the synthesis of **6**. The product was isolated by filtration, quickly washed with cooled *n*-hexane (2 mL), and dried at room temperature under vacuum overnight. Yield: 152 mg (68%). M.p.: 190–192 °C (dec.). ^1^H NMR (C_6_D_6_): δ 8.30 (s, 1H, N*H*), 7.16 (d, *J* = 7.7 Hz, 2H, phenyl), 7.06 (m, 1H, phenyl), 5.75 (s, 1H, N*H*), 3.05 (m, 2H, C*H*(CH_3_)_2_), 2.62 (t, *J* = 7.0 Hz, 2H, C*H*_2_), 2.15 (s, 30H, CpC*H*_3_), 2.03 (m, 4H, C*H*_2_), 1.90 (m, 2H, C*H*_2_), 1.45 (m, 2H, C*H*_2_), 1.32 (m, 2H, C*H*_2_), 1.29 (d, *J* = 6.8 Hz, 6H, CH­(C*H*_3_)_2_), 1.23 (d, *J* = 7.0 Hz, 6H, CH­(C*H*_3_)_2_), 0.66 (m, 2H, C*H*_2_) ppm. ^13^C­{^1^H} NMR (C_6_D_6_): δ 176.8 (ThN*C*N), 173.8 (*C*=N), 164.4 (phenyl), 144.6 (phenyl), 138.1 (phenyl), 124.6 (ring *C*), 123.8 (phenyl), 123.6 (*C*=C), 123.1 (C=*C*), 73.0 (*C*≡N), 32.8 (*C*H_2_), 30.5 (*C*H_2_), 28.2 (*C*H­(CH_3_)_2_), 24.4 (*C*H­(CH_3_)_2_), 23.9 (CH­(*C*H_3_)_2_), 23.5 (CH­(*C*H_3_)_2_), 23.0 (*C*H_2_), 22.9 (*C*H_2_), 22.5 (*C*H_2_), 22.0 (*C*H_2_), 15.7 (*C*H_2_), 11.7 (Cp*C*H_3_) ppm. IR (KBr, cm^–1^): ν 2958 (s), 2912 (s), 2868 (s), 2157­(s; *C*≡N), 1611 (s), 1520 (s), 1432 (s), 1312 (s), 1228 (s), 988 (s), 806 (s). Anal. Calcd for C_44_H_63_N_5_Th: C, 59.11; H, 7.10; N, 7.83. Found: C, 59.14; H, 7.08; N, 7.81. After isolation of the colorless crystals of **28**, the solvent of the mother liquid was removed. ^1^H NMR spectroscopy showed the presence of the resonances of **28**, dmap, and other unidentified compounds in the residue.

### Preparation of (η^5^-C_5_Me_5_)_2_Th­[2-C­(=Ndipp)­N­(C_6_H_11_)-3-N­(C_6_H_11_)-5-(C_6_H_11_)-6,6-(CH_2_)_5_-(1,3-C_4_HN_2_)]·2.5C_6_H_6_ (29·2.5C_6_H_6_)

A benzene (5 mL) solution of C_6_H_11_NC (109 mg, 1.00 mmol) was added to a benzene (10 mL) solution of (η^5^-C_5_Me_5_)_2_Th=N­(dipp)­(dmap) (**4**; 200 mg, 0.25 mmol) without stirring at room temperature. After the solution was stored at room temperature overnight without stirring, yellow crystals were isolated from the solution by filtration. The product was washed with *n*-hexane and dried at room temperature under vacuum overnight, and the product was identified as **29**·2.5C_6_H_6_ by X-ray diffraction analysis. Yield: 269 mg (82%). M.p.: 230–232 °C (dec.). IR (KBr, cm^–1^): ν 2960 (s), 2927 (s), 2853 (s), 1644 (s), 1616 (s), 1448 (s), 1383 (s), 1360 (s), 1260 (s), 1097 (s), 1020 (s), 801 (s). Anal. Calcd for C_75_H_106_N_5_Th: C, 68.72; H, 8.16; N, 5.35. Found: C, 68.74; H, 8.17; N, 5.32. This compound was insoluble in common (deuterated) solvents such as pyridine, THF, toluene, and CD_2_Cl_2_, precluding its characterization by NMR spectroscopy.

### Preparation of (η^5^-C_5_Me_5_)_2_Th­[NH­(*p*-tolyl)]­[2-(dippN_3_)-4-(Me_2_N)­C_5_H_3_N] (**30**). Method **A**

This compound was prepared as yellow crystals from the reaction of (η^5^-C_5_Me_5_)_2_Th=N­(dipp)­(dmap) (**4**; 200 mg, 0.25 mmol) and (*p*-tolyl)­N_3_ (34 mg, 0.25 mmol) in toluene (15 mL) at room temperature and recrystallization from a benzene solution by a similar procedure as that in the synthesis of **6**. The product was isolated by filtration, quickly washed with cooled *n*-hexane (2 mL), and dried at room temperature under vacuum overnight. Yield: 196 mg (84%). M.p.: 186–188 °C (dec.). ^1^H NMR (C_6_D_6_): δ 8.08 (s, 1H, py), 7.68 (d, *J* = 6.6 Hz, 1H, phenyl), 7.29 (d, *J* = 7.6 Hz, 2H, phenyl), 6.96 (d, *J* = 7.9 Hz, 2H, phenyl), 6.91 (d, *J* = 8.2 Hz, 2H, phenyl), 6.62 (d, *J* = 2.5 Hz, 1H, py), 5.81 (m, 1H, py), 3.60 (m, 2H, C*H*(CH_3_)_2_), 2.26 (s, 3H, tolylC*H*_3_), 2.15 (s, 6H, N­(C*H*_3_)_2_), 2.10 (s, 30H, CpC*H*_3_), 1.40 (d, *J* = 6.9 Hz, 12H, CH­(C*H*_3_)_2_) ppm; N*H* proton was not observed. ^13^C­{^1^H} NMR (C_6_D_6_): δ 167.6 (aryl *C*), 156.4 (aryl *C*), 154.3 (aryl *C*), 148.4 (aryl *C*), 145.2 (aryl *C*), 143.3 (aryl *C*), 141.4 (aryl *C*), 129.2 (aryl *C*), 128.3 (aryl *C*), 124.6 (ring *C*), 123.5 (aryl *C*), 119.9 (aryl *C*), 100.4 (py *C*), 87.3 (py *C*), 38.4 (N*C*H_3_), 29.1 (*C*H­(CH_3_)_2_), 28.3 (*C*H­(CH_3_)_2_), 24.2 (CH­(*C*H_3_)_2_), 23.5 (CH­(*C*H_3_)_2_), 20.8 (tolyl*C*H_3_), 11.7 (Cp*C*H_3_) ppm. IR (KBr, cm^–1^): ν 2960 (s), 2915 (s), 2861 (s), 1602 (s), 1427 (s), 1381 (s), 1262 (s), 1148 (s), 1106 (s), 1011 (s), 812 (s). Anal. Calcd for C_46_H_64_N_6_Th: C, 59.21; H, 6.91; N, 9.01. Found: C, 59.24; H, 6.89; N, 9.02.

### Method **B**. NMR Scale

A C_6_D_6_ (0.3 mL) solution of (*p*-tolyl)­N_3_ (2.7 mg, 0.02 mmol) was slowly added to a J. Young NMR tube charged with (η^5^-C_5_Me_5_)_2_Th=N­(dipp)­(dmap) (**4**; 16.0 mg, 0.02 mmol) and C_6_D_6_ (0.2 mL). Resonances of **30** were observed by ^1^H NMR spectroscopy (100% conversion) when this solution was kept at room temperature overnight.

### Preparation of (η^5^-C_5_Me_5_)_2_Th­(N_3_)­[NH­(2,6-^*i*^Pr_2_-4-(Ph_3_C)­C_6_H_2_)] (**31**). Method **A**

This compound was prepared as colorless crystals from the reaction of (η^5^-C_5_Me_5_)_2_Th=N­(dipp)­(dmap) (**4**; 200 mg, 0.25 mmol) and Ph_3_CN_3_ (72 mg, 0.25 mmol) in toluene (15 mL) at room temperature and recrystallization from a benzene solution by a similar procedure as that in the synthesis of **6**. The product was isolated by filtration, quickly washed with cooled *n*-hexane (2 mL), and dried at room temperature under vacuum overnight. Yield: 207 mg (86%). M.p.: 184–186 °C (dec.). ^1^H NMR (C_6_D_6_): δ 7.50 (d, *J* = 7.7 Hz, 6H, phenyl), 7.16 (d, *J* = 4.6 Hz, 2H, phenyl), 7.12 (t, *J* = 7.7 Hz, 6H, phenyl), 7.03 (t, *J* = 7.3 Hz, 3H, phenyl), 4.67 (s, 1H, N*H*), 2.87 (br s, 2H, C*H*(CH_3_)_2_), 1.92 (s, 30H, CpC*H*_3_), 1.27 (m, 12H, CH­(C*H*_3_)_2_) ppm. ^13^C­{^1^H} NMR (C_6_D_6_): δ 149.1 (phenyl *C*), 148.2 (phenyl *C*), 137.0 (phenyl *C*), 134.5 (phenyl *C*), 131.8 (phenyl *C*), 128.3 (phenyl *C*), 127.6 (ring *C*), 126.04 (phenyl *C*), 126.00 (phenyl *C*), 65.4 (Ph_3_ *C*), 28.1 (*C*H­(CH_3_)_2_), 23.4 (CH­(*C*H_3_)_2_), 22.5 (CH­(*C*H_3_)_2_), 10.9 (Cp*C*H_3_) ppm. IR (KBr, cm^–1^): ν 2916 (s), 2862 (s), 2086 (s; N_3_), 1610 (s), 1443 (s), 1359 (s), 1265 (s), 1165 (s), 1030 (s), 1005 (s), 855 (s). Anal. Calcd for C_51_H_62_N_4_Th: C, 63.60; H, 6.49; N, 5.82. Found: C, 63.62; H, 6.51; N, 5.80.

### Method **B**. NMR Scale

A C_6_D_6_ (0.3 mL) solution of Ph_3_CN_3_ (5.7 mg, 0.02 mmol) was slowly added to a J. Young NMR tube charged with (η^5^-C_5_Me_5_)_2_Th=N­(dipp)­(dmap) (**4**; 16.0 mg, 0.02 mmol) and C_6_D_6_ (0.2 mL). Resonances of **31** and those of dmap were observed by ^1^H NMR spectroscopy (100% conversion) when this solution was kept at room temperature overnight.

### Preparation of (η^5^-C_5_Me_5_)_2_Th­(N_3_)­[NH­(2,6-^*i*^Pr_2_-4-(Me_3_Si)­C_6_H_2_)] (**32**). Method **A**

This compound was prepared as colorless crystals from the reaction of (η^5^-C_5_Me_5_)_2_Th=N­(dipp)­(dmap) (**4**; 200 mg, 0.25 mmol) and Me_3_SiN_3_ (29 mg, 0.25 mmol) in toluene (15 mL) at room temperature and recrystallization from a benzene solution by a similar procedure as that in the synthesis of **6**. The product was isolated by filtration, quickly washed with cooled *n*-hexane (2 mL), and dried at room temperature under vacuum overnight. Yield: 163 mg (82%). M.p.: 180–182 °C (dec.). ^1^H NMR (C_6_D_6_): δ 7.46 (s, 2H, phenyl), 4.50 (s, 1H, N*H*), 3.28 (m, 1H, C*H*(CH_3_)_2_), 2.65 (m, 1H, C*H*(CH_3_)_2_), 1.92 (s, 30H, CpC*H*_3_), 1.46 (s, 12H, CH­(C*H*_3_)_2_), 0.37 (s, 9H, Si­(C*H*_3_)_3_) ppm. ^13^C­{^1^H} NMR (C_6_D_6_): δ 153.0 (phenyl *C*), 134.4 (phenyl *C*), 131.5 (phenyl *C*), 128.5 (phenyl *C*), 126.2 (ring *C*), 28.2 (*C*H­(CH_3_)_2_), 23.6 (CH­(*C*H_3_)_2_), 22.6 (CH­(*C*H_3_)_2_), 11.0 (Cp*C*H_3_), −0.36 (Si­(*C*H_3_)_3_) ppm. ^29^Si­{^1^H} NMR (C_6_D_6_): δ −5.22 ppm. IR (KBr, cm^–1^): ν 2956 (s), 2902 (s), 2866 (s), 2092 (s; N_3_), 1614 (s), 1598 (s), 1585 (s), 1460 (s), 1439 (s), 1365 (s), 1261 (s), 1227 (s), 1158 (s), 910 (s), 863 (s), 836 (s). Anal. Calcd for C_35_H_56_N_4_SiTh: C, 53.01; H, 7.12; N, 7.07. Found: C, 52.99; H, 7.11; N, 7.09.

### Method **B**. NMR Scale

A C_6_D_6_ (0.3 mL) solution of Me_3_SiN_3_ (2.3 mg, 0.02 mmol) was slowly added to a J. Young NMR tube charged with (η^5^-C_5_Me_5_)_2_Th=N­(dipp)­(dmap) (**4**; 16.0 mg, 0.02 mmol) and C_6_D_6_ (0.2 mL). Resonances of **32** and those of dmap were observed by ^1^H NMR spectroscopy (100% conversion) when this solution was kept at room temperature overnight.

### X-ray Crystallography

Single-crystal X-ray diffraction measurements were carried out on a Rigaku Saturn CCD diffractometer using Mο Kα radiation (λ = 0.71073 Å) or Cu Kα radiation (λ = 1.54184 Å). An empirical absorption correction was applied using the SADABS program.^12^ All structures were solved by direct methods and refined by full-matrix least-squares on *F* ^2^ using the SHELXL program package.^13^ The crystal data and experimental data for **2**–**19** and **21**–**32** are summarized in the Supporting Information (Tables S1–S7). Selected bond lengths and angles are listed in Table 1.

## Supplementary Material





## Notes

Selected reactivity of the thorium imido complexes, molecular structures, crystal parameters, computational details, and NMR spectra (PDF)
Cartesian coordinates of all stationary points optimized at the B3PW91-SMD level (XYZ)
