# Supplementary material for: Reactivity Studies on the Terminal Thorium Imido Metallocene (η5‑C5Me5)2Th(=Ndipp)(dmap)
Source: Inorg Chem. 2026 Jun 28;65(27):15299–325. doi: 10.1021/acs.inorgchem.6c01355 (PMC13370881; doi:10.1021/acs.inorgchem.6c01355)
Supplement: Supplementary file 1 [file ic6c01355_si_001.pdf]

Supporting Information for

Reactivity Studies on the Terminal Thorium Imido Metallocene (η^5-C_5Me_5)$_2$Th(=Ndipp)(dmap)

Yi Heng,[†] Xiaoying Cao,[†] Wanjian Ding,[†] Guohua Hou,[†] Guofu Zi,*[,†] and Marc D. Walter*[,‡]

[†]Department of Chemistry, Beijing Normal University, Beijing 100875, China

[‡]Institut für Anorganische und Analytische Chemie, Technische Universität Braunschweig, Hagenring 30, 38106 Braunschweig, Germany

*Corresponding authors. E-mail: gzi@bnu.edu.cn (G.Z.), mwalter@tu-bs.de (M.D.W.)

Table of contents

1. **Figures** **S2**
2. **Crystallographic details** **S9**
3. **Computational Studies** **S16**
4. **NMR spectra** **S23**
5. **References** **S52**

1. Figures

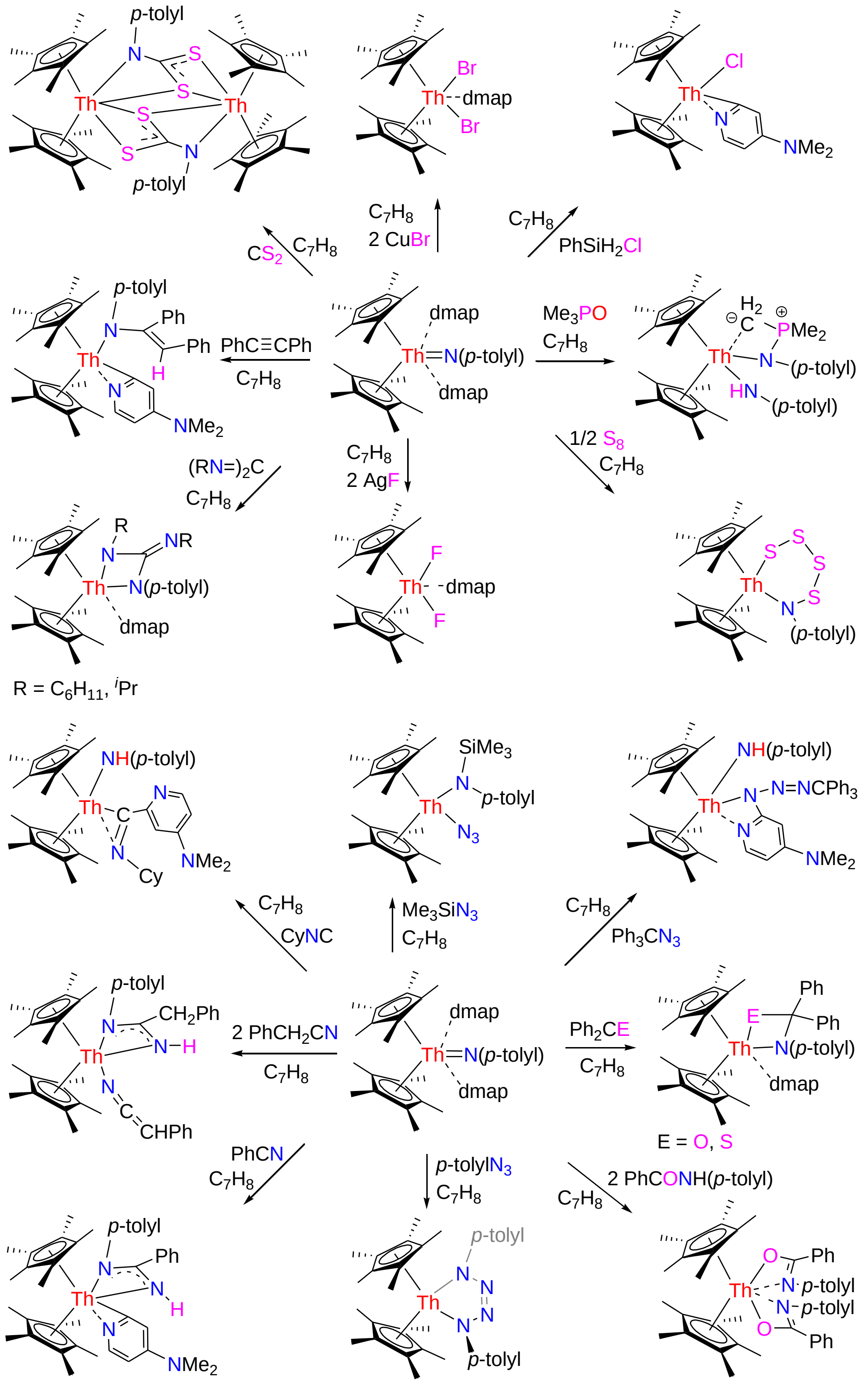

Figure S1. Selected examples concerning the reactivity of (η^5-C_5Me_5)$_2$Th=N(*p*-tolyl)(dmap)$_2$.

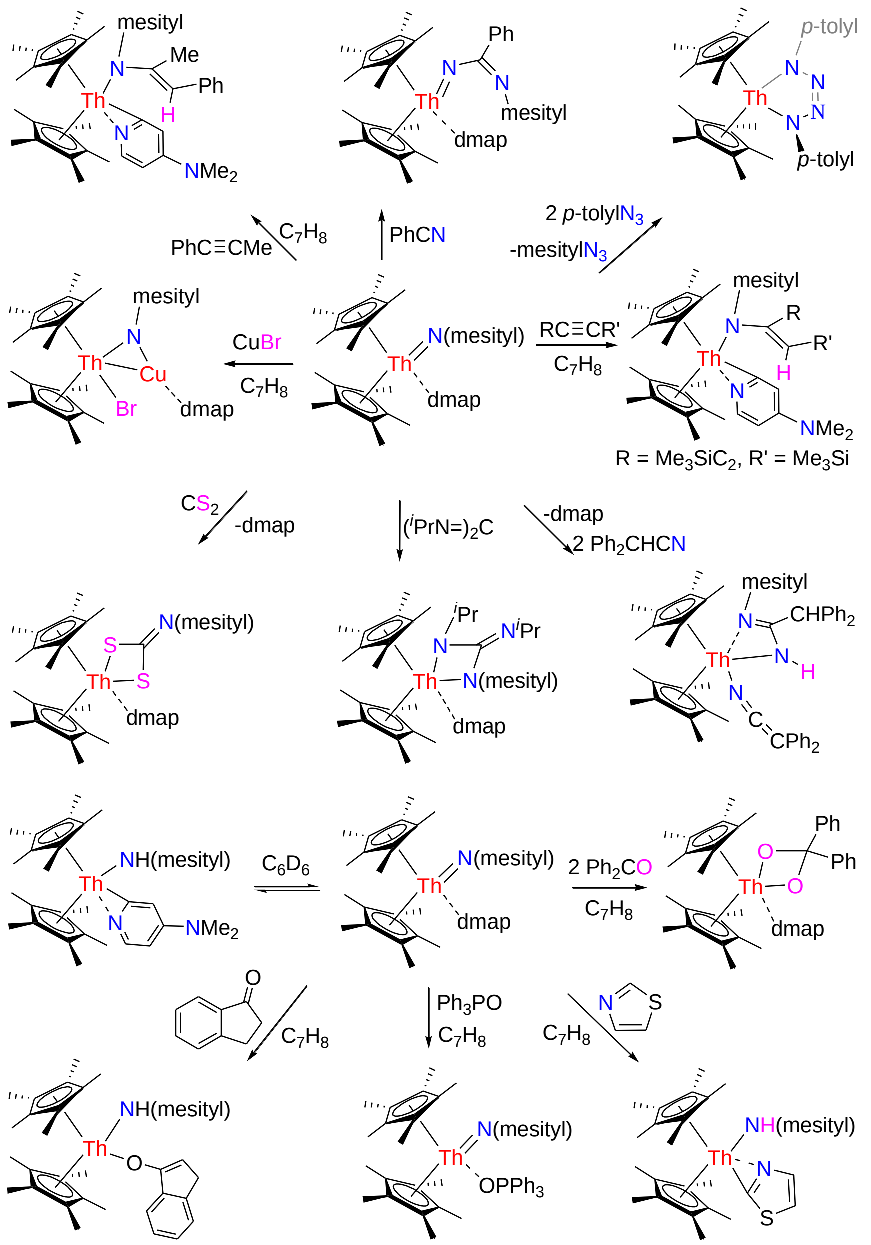

Figure S2. Selected examples concerning the reactivity of (η^5-C_5Me_5)$_2$Th=N(mesityl)(dmap).

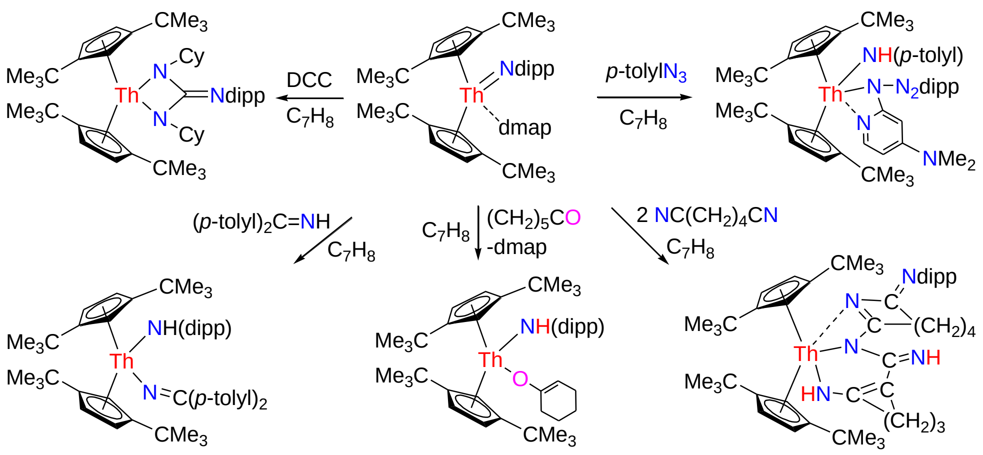

Figure S3a. Selected examples concerning the reactivity of [η^5-1,3-(Me_3C)$_2C_5H_3$]$_2$Th=N(dipp)(dmap).

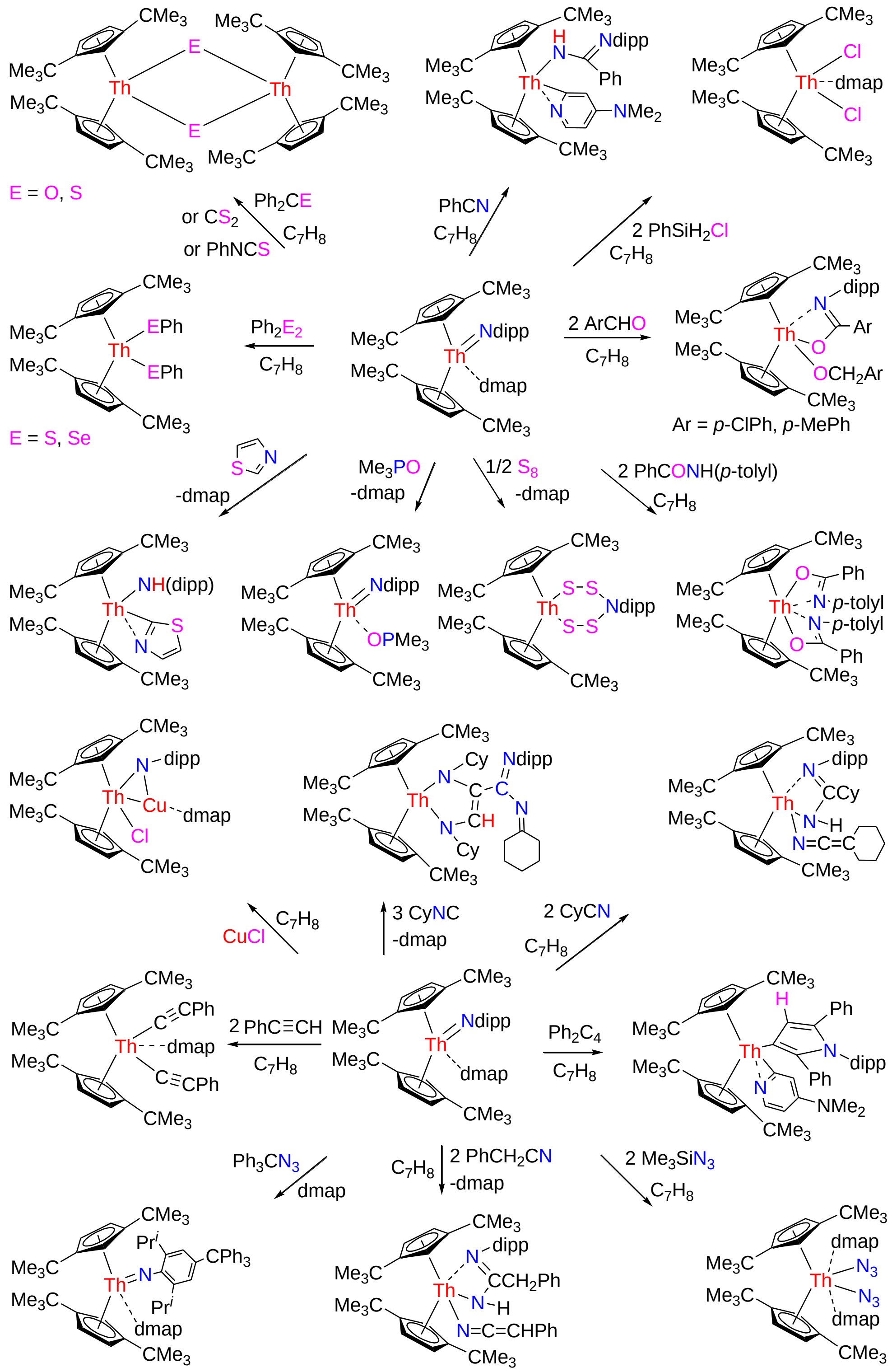

Figure S3b. Selected examples concerning the reactivity of [η^5-1,3-($Me_3C)_2C_5H_3]_2Th$=N(dipp)(dmap).

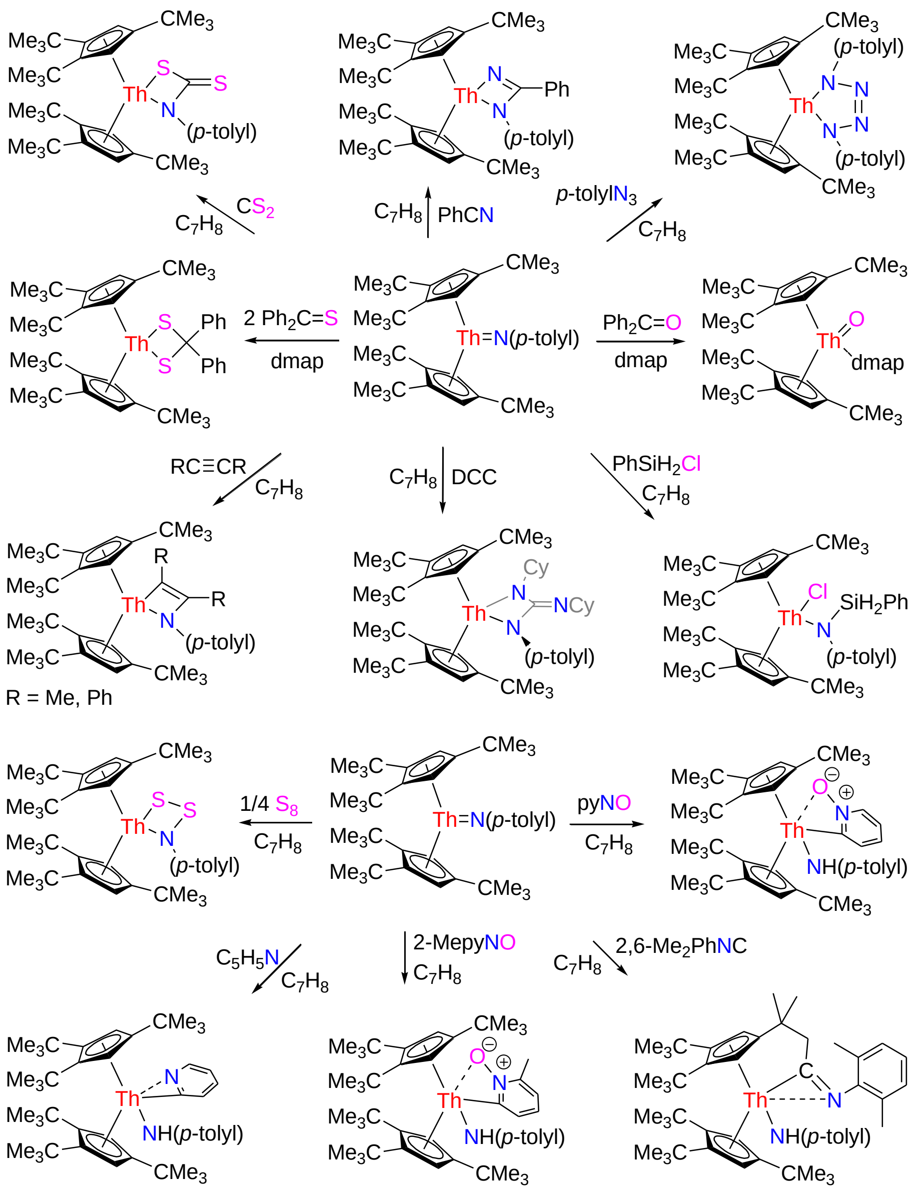

Figure S4. Selected examples concerning the reactivity of $[\eta^5\text{-}1,2,4\text{-}(Me_3C)_3C_5H_2]_2Th{=}N(p\text{-tolyl})$.

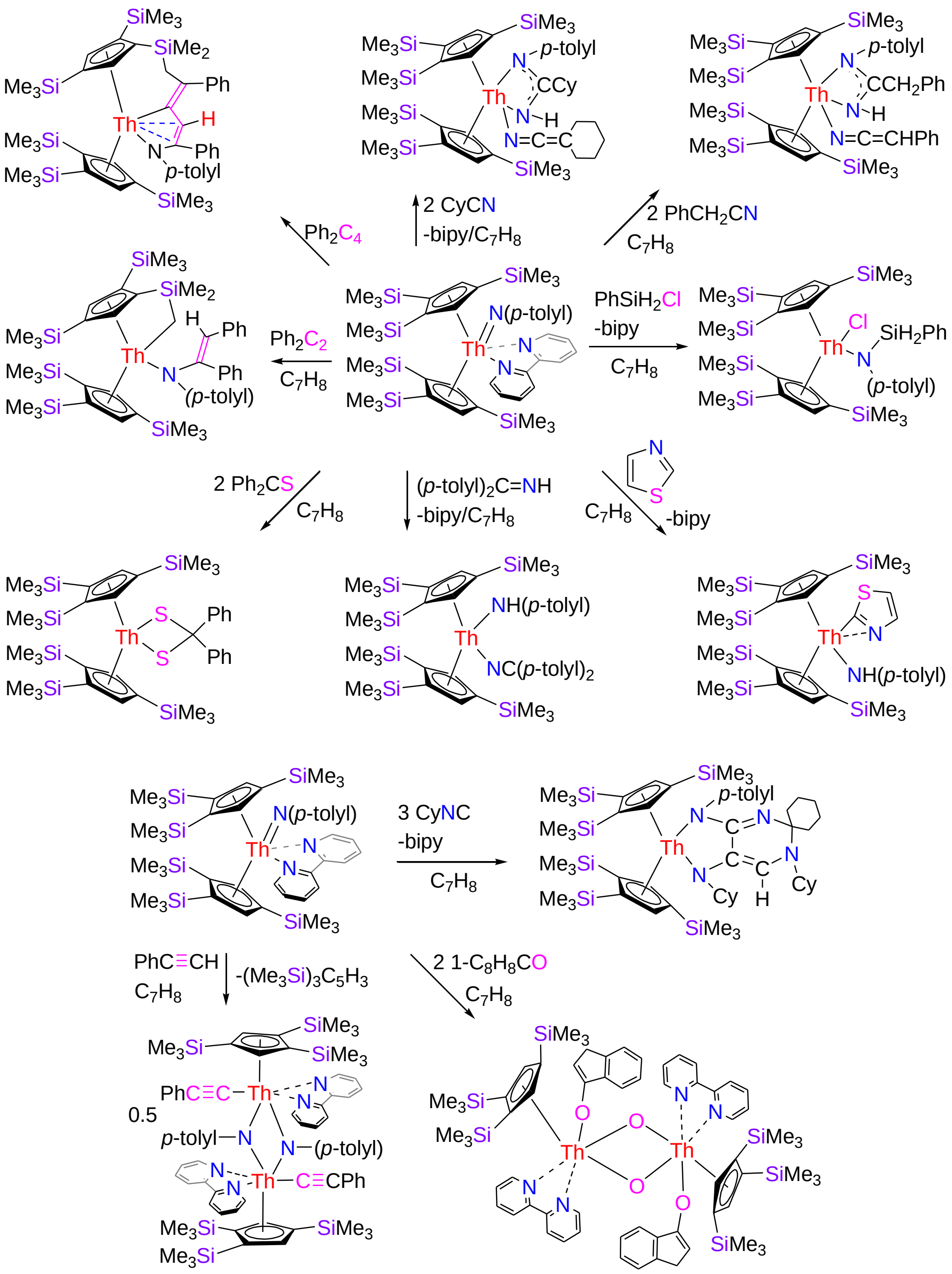

Figure S5. Selected examples concerning the reactivity of [η^5-1,2,4-$(Me_3Si)_3C_5H_2$]$_2$Th=N(*p*-tolyl)(bipy).

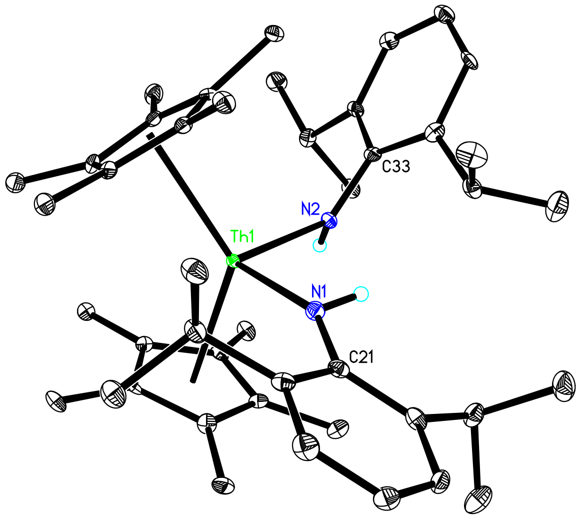

Figure S6. Molecular structure of **8** (thermal ellipsoids drawn at the 35% probability level). Selected bond lengths (Å) and angles (°): Th(1)-C(Cp) (av.) 2.840(11), Th(1)-C(Cp) (range) 2.786(11) to 2.877(13), Th(1)-Cp (cent) 2.563(11) and 2.582(11), Th(1)-N(1) 2.341(10), Tn(1)-N(2) 2.335(16), Cp(cent)-Th(1)-Cp(cent) 129.6(4), N(1)-Th(1)-N(2) 99.6(5).

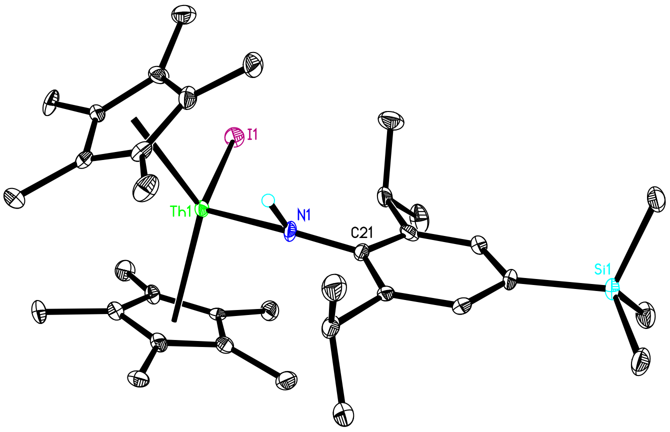

Figure S7. Molecular structure of **12** (thermal ellipsoids drawn at the 35% probability level).

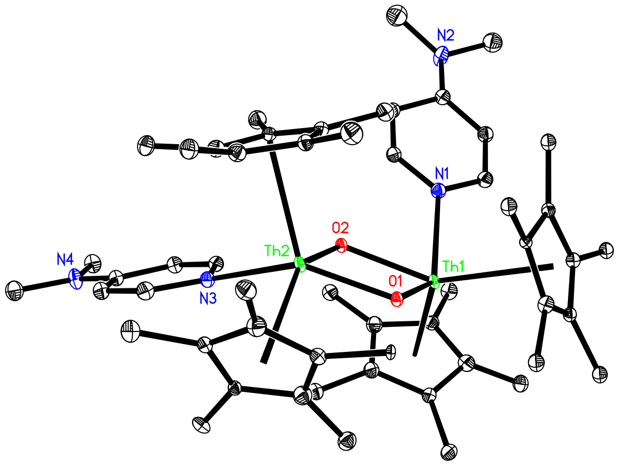

Figure S8. Molecular structure of **20** (thermal ellipsoids drawn at the 35% probability level).

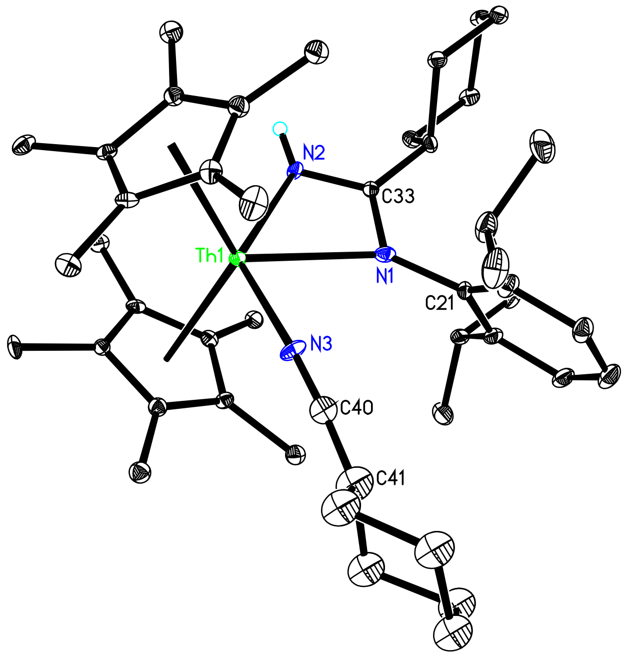

Figure S9. Molecular structure of **27** (thermal ellipsoids drawn at the 35% probability level).

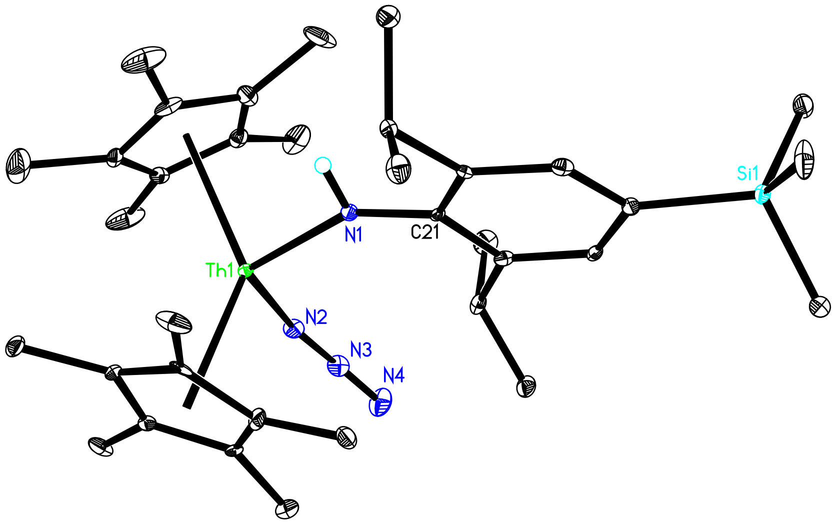

Figure S10. Molecular structure of **32** (thermal ellipsoids drawn at the 35% probability level).

2. Crystallographic details

Table S1. Crystal Data and Experimental Parameters for Compounds 2-6

Compound	**2**	**3**	**4**	**5** $\cdot 0.5C_6H_6$	**6**
Formula	$C_{44}H_{66}N_2Th$	$C_{37}H_{52}N_2Th$	$C_{39}H_{57}N_3Th$	$C_{38}H_{59}NOPTh$	$C_{35}H_{50}N_2STh$
Fw	855.02	756.84	799.91	808.87	762.87
crystal system	monoclinic	monoclinic	orthorhombic	monoclinic	monoclinic
space group	$C2/c$	$P2_1/n$	$P2_12_12_1$	$P2_1/c$	$P2_1/n$
a (Å)	21.738(1)	10.741(1)	13.689(1)	19.016(1)	10.667(1)
b (Å)	11.936(1)	39.304(1)	15.805(1)	11.263(1)	38.852(1)
c (Å)	30.312(1)	16.135(1)	16.506(1)	18.265(1)	16.129(1)
α (deg)	90	90	90	90	90
β (deg)	97.07(1)	95.58(1)	90	109.63(1)	94.28(1)
γ (deg)	90	90	90	90	90
V (Å^3)	7805.36(13)	6779.1(2)	3571.08(9)	3684.70(7)	6665.77(11)
temperature (K)	99.98(10)	101(1)	99.99(10)	100.00(10)	99.99(10)
Z	8	8	4	4	8
D_{calc} (g/cm^3)	1.455	1.483	1.488	1.458	1.520
μ(Mo/Kα)$_{calc}$ (cm^{-1})	12.540	14.362	13.672	13.658	15.181
size (mm)	0.10 × 0.10 × 0.10	0.50 × 0.20 × 0.20	0.10 × 0.10 × 0.10	0.20 × 0.05 × 0.05	0.40 × 0.20 × 0.20
F(000)	3472	3024	1608	1628	3040
2θ range (deg)	8.20 to 153.26	7.11 to 153.11	7.74 to 152.58	9.28 to 152.05	7.14 to 152.84
no. of reflns, collected	26300	54878	14918	26347	47271
no of obsd reflns	7707	13453	6448	7295	13431
no of variables	450	743	326	396	734
abscorr (T_{max}, T_{min})	1.00, 0.64	1.00, 0.17	1.00, 0.68	1.00, 0.35	1.00, 0.55
R	0.039	0.061	0.049	0.033	0.037
R_w	0.105	0.173	0.115	0.083	0.088
R_{all}	0.044	0.077	0.052	0.046	0.044
Gof	1.07	1.05	1.03	1.07	1.05
CCDC	2535660	2535683	2535656	2535662	2535676

Table S2. Crystal Data and Experimental Parameters for Compounds 7-11

Compound	**7**	**8**	**9**	**10**	**11**
Formula	$C_{38}H_{54}N_2OTh$	$C_{38}H_{54}N_2Th$	$C_{39}H_{50}N_4Th$	$C_{33}H_{46}N_2Cl_2Th$	$C_{35}H_{56}NClSiTh$
Fw	786.87	770.87	806.87	773.66	786.38
crystal system	monoclinic	orthorhombic	triclinic	triclinic	monoclinic
space group	*Cc*	$P2_12_12_1$	$P\bar{1}$	$P\bar{1}$	$P2_1/c$
a (Å)	9.166(1)	16.200(1)	10.844(1)	9.429(1)	17.154(1)
b (Å)	21.828(1)	15.360(1)	12.592(1)	11.082(1)	15.687(1)
c (Å)	17.569(1)	13.513(1)	15.972(1)	15.701(1)	13.856(1)
α (deg)	90	90	72.19(1)	94.49(1)	90
β (deg)	101.82(1)	90	86.89(1)	96.99(1)	109.24(1)
γ (deg)	90	90	69.59(1)	92.74(1)	90
V (Å^3)	3440.38(14)	3362.31(16)	1942.62(6)	1620.72(5)	3520.38(9)
temperature (K)	100.00(10)	100.00(10)	99.99(10)	100.00(10)	100.00(10)
Z	4	4	2	2	4
D_{calc} (g/cm^3)	1.519	1.523	1.379	1.585	1.484
μ(Mo/Kα)$_{calc}$ (cm^{-1})	14.196	14.490	12.584	16.514	14.835
size (mm)	0.10 × 0.10 × 0.05	0.10 × 0.10 × 0.10	0.20 × 0.15 × 0.10	0.20 × 0.10 × 0.10	0.20 × 0.10 × 0.10
F(000)	1576	1544	804	764	1576
2θ range (deg)	8.10 to 152.67	7.93 to 152.83	7.87 to 152.62	8.02 to 152.75	7.85 to 153.21
no. of reflns, collected	11605	12831	25304	21038	45519
no of obsd reflns	4552	6093	7809	6474	7259
no of variables	398	350	397	355	360
abscorr (T_{max}, T_{min})	1.00, 0.47	1.00, 0.47	1.00, 0.42	1.00, 0.71	1.00, 0.18
R	0.037	0.067	0.032	0.026	0.050
R_w	0.107	0.197	0.080	0.068	0.114
R_{all}	0.037	0.072	0.033	0.027	0.055
Gof	1.08	1.14	1.09	1.08	1.14
CCDC	2535654	2535657	2535668	2535658	2535672

Table S3. Crystal Data and Experimental Parameters for Compounds 12-15

Compound	**12**	**13**	**14** ·2.5C_6H_6	**15**
Formula	$C_{35}H_{56}NISiTh$	$C_{42}H_{62}NBrTh$	$C_{79}H_{109}N_2I_2Th_2$	$C_{32}H_{47}NS_4Th$
Fw	877.83	892.87	1804.56	805.98
crystal system	monoclinic	triclinic	triclinic	triclinic
space group	$P2_1/n$	$P\bar{1}$	$P\bar{1}$	$P\bar{1}$
a (Å)	13.953(1)	9.745(1)	10.297(1)	9.839(1)
b (Å)	15.677(1)	9.908(1)	17.921(1)	13.084(1)
c (Å)	16.714(1)	23.834(1)	23.619(1)	13.557(1)
α (deg)	90	80.38(1)	68.25(1)	103.19(1)
β (deg)	98.19(1)	86.75(1)	86.29(1)	93.74(1)
γ (deg)	90	62.36(1)	76.40(1)	100.35(1)
V (Å^3)	3618.57(15)	2009.33(11)	3933.69(12)	1661.08(7)
temperature (K)	100.00(10)	100.00(10)	100.00(11)	100.01(10)
Z	4	2	2	2
D_{calc} (g/cm^3)	1.611	1.476	1.524	1.611
μ(Mo/Kα)$_{calc}$ (cm^{-1})	20.453	13.317	18.554	16.972
size (mm)	0.20 × 0.10 × 0.05	0.10 × 0.10 × 0.05	0.25 × 0.15 × 0.10	0.20 × 0.10 × 0.10
F(000)	1720	892	1766	800
2θ range (deg)	7.73 to 152.46	7.53 to 153.24	7.88 to 152.72	6.74 to 153.12
no. of reflns, collected	24928	23109	53333	19546
no of obsd reflns	7288	7955	15814	6646
no of variables	373	428	802	357
abscorr (T_{max}, T_{min})	1.00, 0.19	1.00, 0.50	1.00, 0.07	1.00, 0.17
R	0.053	0.053	0.034	0.036
R_w	0.140	0.136	0.084	0.094
R_{all}	0.062	0.059	0.038	0.039
Gof	1.06	1.10	1.06	1.08
CCDC	2535663	2535680	2535684	2535678

Table S4. Crystal Data and Experimental Parameters for Compounds 16-19

Compound	**16**	**17**	**18**	**19**
Formula	$C_{48}H_{65}N_3Th$	$C_{55}H_{67}N_3Th$	$C_{40}H_{53}NTh$	$C_{92}H_{142}N_{10}Th_2$
Fw	916.07	1002.15	779.87	1852.23
crystal system	monoclinic	monoclinic	triclinic	monoclinic
space group	$P2_1/n$	$P2_1/n$	$P\bar{1}$	$P2_1/n$
a (Å)	11.143(1)	11.525(1)	10.510(1)	25.845(1)
b (Å)	15.609(1)	20.219(1)	15.793(1)	12.743(1)
c (Å)	23.587(1)	20.652(1)	22.385(1)	26.342(1)
α (deg)	90	90	78.04(1)	90
β (deg)	96.18(1)	101.78(1)	83.86(1)	97.59(1)
γ (deg)	90	90	78.41(1)	90
V (Å^3)	4078.85(8)	4711.29(8)	3552.41(15)	8599.3(3)
temperature (K)	100.00(10)	100.01(10)	99.98(10)	100.00(10)
Z	4	4	4	4
D_{calc} (g/cm^3)	1.492	1.413	1.458	1.431
μ(Mo/Kα)$_{calc}$ (cm^{-1})	12.050	10.486	13.715	11.447
size (mm)	0.15 × 0.10 × 0.10	0.10 × 0.05 × 0.02	0.10 × 0.08 × 0.05	0.15 × 0.10 × 0.08
F(000)	1856	2032	1560	3776
2θ range (deg)	6.80 to 152.56	8.16 to 152.96	6.42 to 152.74	6.77 to 153.51
no. of reflns, collected	29167	33165	43066	17295
no of obsd reflns	8208	9466	14235	17295
no of variables	486	548	791	894
abscorr (T_{max}, T_{min})	1.00, 0.18	1.00, 0.51	1.00, 0.66	1.00, 0.65
R	0.044	0.044	0.041	0.107
R_w	0.112	0.117	0.105	0.304
R_{all}	0.049	0.049	0.053	0.134
Gof	1.02	1.01	1.09	1.10
CCDC	2535682	2535669	2535673	2535659

Table S5. Crystal Data and Experimental Parameters for Compounds 21-24

Compound	**21**	**22**	**23**	**24**
Formula	$C_{38}H_{57}NOTh$	$C_{40}H_{57}N_3S_2Th$	$C_{46}H_{50}N_2O_2Th$	$C_{46}H_{50}N_2Th$
Fw	775.88	876.04	894.92	862.92
crystal system	orthorhombic	triclinic	monoclinic	monoclinic
space group	$P2_12_12_1$	$P\bar{1}$	$P2_1/c$	$P2_1/c$
a (Å)	14.685(1)	13.474(1)	15.948(1)	17.537(1)
b (Å)	15.205(1)	14.424(1)	15.177(1)	10.300(1)
c (Å)	15.638(1)	26.389(1)	16.176(1)	21.813(1)
α (deg)	90	91.20(1)	90	90
β (deg)	90	104.58(1)	98.63(1)	104.57(1)
γ (deg)	90	91.79(1)	90	90
V (Å^3)	3491.60(8)	4958.93(18)	3870.83(11)	3813.45(6)
temperature (K)	100.01(10)	100.00(10)	100.01(10)	100.00(10)
Z	4	4	4	4
D_{calc} (g/cm^3)	1.476	1.173	1.536	1.503
μ(Mo/Kα)$_{calc}$ (cm^{-1})	13.968	10.655	12.723	12.851
size (mm)	0.20 × 0.10 × 0.10	0.10 × 0.05 × 0.05	0.10 × 0.10 × 0.10	0.20 × 0.20 × 0.20
F(000)	1560	1760	1784	1720
2θ range (deg)	8.11 to 153.03	6.78 to 178.20	8.03 to 157.89	8.38 to 152.97
no. of reflns, collected	13474	19860	28094	27367
no of obsd reflns	6234	19860	7656	7682
no of variables	388	778	362	452
abscorr (T_{max}, T_{min})	1.00, 0.45	1.00, 0.29	1.00, 0.62	1.00, 0.75
R	0.029	0.107	0.089	0.028
R_w	0.074	0.284	0.200	0.074
R_{all}	0.029	0.147	0.096	0.030
Gof	1.08	1.08	1.44	1.06
CCDC	2535679	2535677	2535666	2535667

Table S6. Crystal Data and Experimental Parameters for Compounds 25-28

Compound	**25**	**26**	**27**	**28**
Formula	$C_{46}H_{62}N_4Th$	$C_{48}H_{61}N_3Th$	$C_{46}H_{69}N_3Th$	$C_{44}H_{63}N_5Th$
Fw	903.03	912.03	896.08	894.03
crystal system	monoclinic	monoclinic	monoclinic	monoclinic
space group	$P2_1/n$	$P2_1/n$	Pn	$P2_1/c$
a (Å)	11.343(1)	9.991(1)	11.117(1)	14.715(1)
b (Å)	17.983(1)	19.777(1)	10.689(1)	17.130(1)
c (Å)	20.800(1)	20.648(1)	21.023(1)	17.558(1)
α (deg)	90	90	90	90
β (deg)	94.90(1)	91.15(1)	99.29(1)	110.17(1)
γ (deg)	90	90	90	90
V (Å^3)	4227.25(7)	4078.94(17)	2465.28(11)	4154.04(9)
temperature (K)	99.99(13)	100.01(10)	99.99(10)	100.01(10)
Z	4	4	2	4
D_{calc} (g/cm^3)	1.419	1.485	1.207	1.430
μ(Mo/Kα)$_{calc}$ (cm^{-1})	11.626	12.050	9.954	11.831
size (mm)	0.20 × 0.10 × 0.10	0.20 × 0.10 × 0.10	0.20 × 0.10 × 0.10	0.25 × 0.15 × 0.10
F(000)	1824	1840	912	1808
2θ range (deg)	6.51 to 152.34	8.57 to 153.62	8.27 to 152.66	7.44 to 152.58
no. of reflns, collected	30769	27834	15649	30145
no of obsd reflns	8394	8162	6549	8353
no of variables	475	487	410	449
abscorr (T_{max}, T_{min})	1.00, 0.67	1.00, 0.63	1.00, 0.53	1.00, 0.28
R	0.038	0.040	0.049	0.038
R_w	0.096	0.099	0.124	0.106
R_{all}	0.044	0.049	0.053	0.040
Gof	1.06	1.09	1.06	1.07
CCDC	2535674	2535670	2535664	2535665

Table S7. Crystal Data and Experimental Parameters for Compounds 29-32

Compound	**29**	**30**	**31**	**32**
Formula	$C_{75}H_{106}N_5Th$	$C_{46}H_{64}N_6Th$	$C_{51}H_{62}N_4Th$	$C_{35}H_{56}N_4SiTh$
Fw	1309.68	933.07	963.08	792.96
crystal system	triclinic	triclinic	triclinic	triclinic
space group	$P\bar{1}$	$P\bar{1}$	$P\bar{1}$	$P\bar{1}$
a (Å)	12.230(1)	11.779(1)	9.552(1)	11.279(1)
b (Å)	15.191(1)	12.486(1)	9.591(1)	11.777(1)
c (Å)	18.478(1)	16.719(1)	25.960(1)	13.836(1)
α (deg)	98.17(1)	95.18(1)	85.41(1)	87.39(1)
β (deg)	91.19(1)	105.16(1)	85.75(1)	84.39(1)
γ (deg)	95.98(1)	113.82(1)	70.85(1)	84.80(1)
V (Å^3)	3377.3(2)	2117.07(9)	2236.60(11)	1820.24(13)
temperature (K)	100.00(10)	100.00(10)	99.98(10)	100.01(10)
Z	2	2	2	2
D_{calc} (g/cm^3)	1.288	1.464	1.430	1.447
μ(Mo/Kα)$_{calc}$ (cm^{-1})	2.252	11.640	11.027	4.156
size (mm)	0.20 × 0.10 × 0.10	0.20 × 0.10 × 0.10	0.30 × 0.10 × 0.10	0.20 × 0.10 × 0.05
F(000)	1362	944	972	796
2θ range (deg)	6.70 to 57.74	7.94 to 152.19	6.84 to 153.66	6.65 to 56.90
no. of reflns, collected	32123	26507	27214	14449
no of obsd reflns	14438	8459	8913	7556
no of variables	732	475	522	381
abscorr (T_{max}, T_{min})	1.00, 0.71	1.00, 0.58	1.00, 0.36	1.00, 0.65
R	0.038	0.042	0.040	0.056
R_w	0.074	0.110	0.101	0.088
R_{all}	0.051	0.044	0.044	0.077
Gof	1.04	1.06	1.11	1.03
CCDC	2535671	2535661	2535675	2535655

3. Computational Studies

Calculations were performed with the Gaussian 09 program (G09),[1] employing the B3PW91 functional, plus a SMD[2] model (denoted as B3PW91-SMD), with a standard 6-31G(d) basis set for the elements C, H and N and a quasi-relativistic 5f-in-valence effective-core potential (ECP78MWB) treatment with 78 electrons in the core region for Th and the corresponding optimized segmented ((14s13p10d8f6g)/[10s9p5d4f3g]) basis set for the valence shells of Th,[3] to fully optimize the structures of reactants, transition state(s), intermediates, and products, and to also account for the experimental reaction conditions using toluene as a solvent (dielectric constant ε = 2.379). All stationary points were subsequently characterized by vibrational analyses, from which their respective zero-point (vibrational) energy (ZPE) were extracted and used in the relative energy determinations. In addition, frequency calculations were also performed to ensure that the determined structures for reactants, intermediates, products and transition states resided at minima and 1st order saddle points, respectively, on their potential energy hypersurfaces.

Table S8. The optimized Cartesian Coordinates (in Å) of stationary points for **4** ⇌ **4′**, obtained with B3PW91-SMD/6-31G(d,p)$\cup$ECP78MWB method.

Species	Cartesian coordinates	
**4**	C 7.171119 7.240962 12.247717<br>C 7.789145 6.057915 11.765474<br>C 8.809252 6.442954 10.835186<br>C 8.832539 7.866827 10.769873<br>C 7.806407 8.361966 11.627410<br>C 9.827284 5.531783 10.207650<br>H 10.720310 5.433317 10.843085<br>H 10.171360 5.904183 9.236271<br>H 9.440661 4.519620 10.044870<br>C 7.569129 4.678575 12.322631<br>H 8.073442 3.910673 11.726236<br>H 6.509278 4.400157 12.394080<br>H 7.978209 4.594932 13.340502<br>C 6.233578 7.322816 13.417179<br>H 5.694452 6.384950 13.581400<br>H 5.487553 8.118989 13.311848<br>H 6.788218 7.537566 14.343557<br>C 7.588466 9.798063 12.011204<br>H 6.538690 10.016525 12.241731<br>H 7.898865 10.485929 11.219226<br>H 8.167722 10.061878 12.909222<br>C 9.867724 8.694956 10.067717<br>H 10.730219 8.887569 10.723939<br>H 9.477515 9.666080 9.749694<br>H 10.249840 8.199890 9.169822<br>C 3.697804 6.683626 8.218463<br>C 3.625509 8.022192 8.691992<br>C 3.580634 7.980050 10.121039<br>C 3.575144 6.605702 10.519902<br>C 3.657986 5.809981 9.349298<br>C 3.518209 4.315830 9.302193<br>H 2.460126 4.017824 9.241326<br>H 3.925129 3.830383 10.197993<br>H 4.019314 3.876213 8.432976	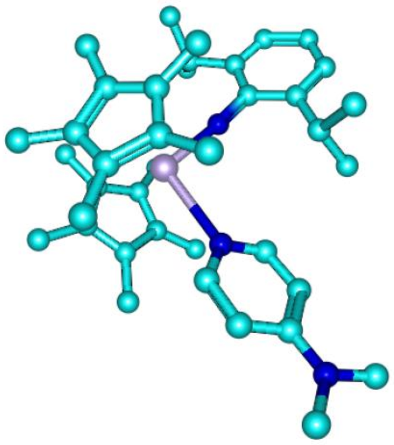

	C	3.583156	6.264962	6.779105	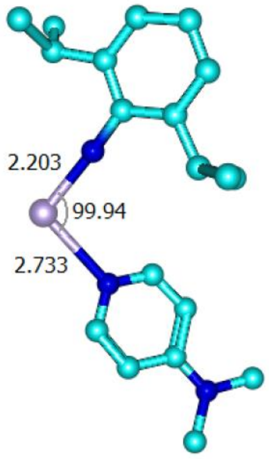
	H	4.036654	5.284388	6.593307	
	H	4.058096	6.982446	6.099934	
	H	2.529393	6.186836	6.469537	
	C	3.405979	9.226642	7.826290	
	H	4.057905	9.232055	6.946621	
	H	3.583406	10.158615	8.370024	
	H	2.367199	9.262205	7.463448	
	C	3.312957	9.157240	11.018517	
	H	3.763871	10.081164	10.638813	
	H	3.694753	9.004034	12.034505	
	H	2.233752	9.350305	11.113937	
	C	3.294277	6.080345	11.898601	
	H	2.249600	5.744320	11.983014	
	H	3.442679	6.841927	12.669406	
	H	3.917705	5.217727	12.166888	
	C	7.708562	9.493992	6.902133	
	C	8.663897	9.076747	5.903987	
	C	9.225886	9.997544	5.021165	
	H	9.938716	9.642316	4.276886	
	C	8.912305	11.353082	5.062023	
	H	9.363829	12.055501	4.365173	
	C	8.005023	11.780684	6.026609	
	H	7.752820	12.839537	6.080075	
	C	7.404399	10.904037	6.930586	
	C	9.076956	7.621186	5.780135	
	H	8.594467	7.096096	6.612736	
	C	8.579204	7.003712	4.465497	
	H	7.492799	7.110384	4.357613	
	H	8.830972	5.935783	4.403814	
	H	9.038712	7.497496	3.599459	
	C	10.592092	7.419558	5.917307	
	H	11.140521	7.883867	5.087624	
	H	10.847816	6.350802	5.918842	
	H	10.971697	7.860719	6.845941	
	C	6.438945	11.486295	7.948388	
	H	6.024696	10.636617	8.504347	
	C	5.276245	12.241907	7.288979	
	H	5.630880	13.136554	6.761543	
	H	4.547261	12.578182	8.038893	
	H	4.750492	11.617816	6.558740	
	C	7.152754	12.408692	8.947821	
	H	8.003236	11.906961	9.422431	
	H	6.468917	12.746005	9.739031	
	H	7.543424	13.304440	8.447636	
	C	8.000175	1.095236	4.835116	
	H	7.092110	1.372856	4.285011	
	H	8.259474	0.071881	4.561097	
	H	8.818566	1.752170	4.511828	
	C	8.013682	-0.013761	7.089940	
	H	8.838006	0.134794	7.800273	
	H	8.272110	-0.854477	6.445036	
	H	7.112183	-0.286111	7.653731	
	N	7.141952	8.600701	7.763513	
	N	6.823889	4.832921	8.088159	
	C	6.969704	3.695923	8.798321	
	H	6.829150	3.777457	9.871598	
	C	7.279206	2.467153	8.253697	
	H	7.380757	1.615648	8.915559	
	C	7.461706	2.344199	6.853589	
	C	7.273385	3.533150	6.105660	
	H	7.371276	3.544442	5.027072	
	C	6.970826	4.714803	6.752628	
	H	6.841335	5.625354	6.177359	
	N	7.793589	1.165274	6.271073	
	Th	6.380447	7.234120	9.315169	
**TS4**	C	7.131011	7.914258	11.543854	
	C	7.920907	6.753164	11.309969	
	C	8.867626	7.057260	10.283861	

C	8.664413	8.409914	9.881144
C	7.577628	8.935656	10.646181
C	9.987448	6.169852	9.825635
H	10.881408	6.306795	10.452469
H	10.281988	6.387140	8.794791
H	9.724763	5.107365	9.870526
C	7.889126	5.501971	12.141242
H	8.338278	4.649471	11.619938
H	6.871396	5.215719	12.434620
H	8.456921	5.632823	13.074520
C	6.203726	8.107411	12.708542
H	5.593459	7.220404	12.915679
H	5.522582	8.950986	12.562167
H	6.773780	8.316497	13.626395
C	7.151749	10.377571	10.694537
H	6.087311	10.493979	10.930908
H	7.335375	10.899321	9.748787
H	7.706599	10.927711	11.468716
C	9.554086	9.173792	8.943768
H	10.514599	9.412551	9.423651
H	9.109535	10.124671	8.633799
H	9.775609	8.614340	8.029409
C	3.606222	6.673956	7.715516
C	3.461692	7.856751	8.501573
C	3.478895	7.473326	9.881922
C	3.654024	6.063718	9.944242
C	3.736433	5.569638	8.607446
C	3.702183	4.127757	8.194712
H	2.668743	3.810279	7.989323
H	4.090204	3.462847	8.972708
H	4.283388	3.943018	7.285608
C	3.495398	6.566110	6.221956
H	4.201608	5.837553	5.808406
H	3.694796	7.520494	5.726248
H	2.485287	6.247521	5.923700
C	3.074587	9.220575	8.002151
H	3.314423	9.353307	6.943505
H	3.560645	10.033649	8.555671
H	1.990762	9.379819	8.105863
C	3.079178	8.374325	11.015560
H	3.503936	9.382203	10.932888
H	3.368358	7.973168	11.991201
H	1.986198	8.502225	11.036976
C	3.566127	5.218495	11.183873
H	2.530764	4.899902	11.376106
H	3.899614	5.755889	12.079012
H	4.163298	4.301987	11.108994
C	7.888305	8.170212	5.784440
C	9.139146	7.728863	5.228719
C	9.840724	8.539348	4.333879
H	10.785540	8.177216	3.931360
C	9.375473	9.788662	3.938657
H	9.943398	10.404339	3.244987
C	8.154277	10.218442	4.444583
H	7.760474	11.184096	4.129367
C	7.406065	9.445813	5.335201
C	9.731087	6.366532	5.557657
H	9.153592	5.948789	6.388577
C	9.586422	5.408873	4.365777
H	8.542492	5.331030	4.044649
H	9.943045	4.402207	4.621477
H	10.171850	5.763720	3.507106
C	11.201978	6.430721	5.994433
H	11.862668	6.730252	5.171142
H	11.542915	5.445256	6.338117
H	11.354789	7.145023	6.811546
C	6.050581	9.982368	5.761408
H	5.624357	9.242895	6.449945
C	5.094608	10.093763	4.564584

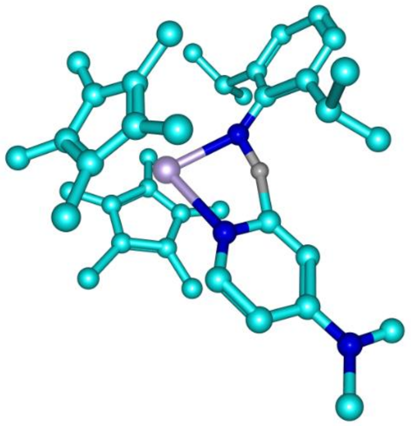

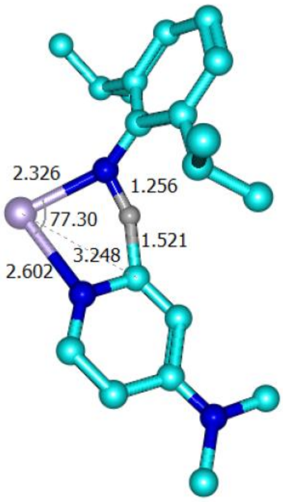

	H 5.458530 10.826200 3.832955<br>H 4.093686 10.417835 4.880446<br>H 4.996563 9.133354 4.046160<br>C 6.144213 11.326326 6.495142<br>H 6.800833 11.263577 7.371554<br>H 5.155366 11.663854 6.833192<br>H 6.550505 12.111101 5.845143<br>C 7.142301 1.030325 4.569323<br>H 6.193425 1.447389 4.204486<br>H 7.216052 -0.000249 4.217918<br>H 7.963719 1.601210 4.117438<br>C 7.268213 -0.208942 6.743308<br>H 8.126551 -0.243481 7.427057<br>H 7.377331 -1.030675 6.033393<br>H 6.354031 -0.385666 7.328647<br>N 7.182645 7.409931 6.703569<br>N 6.948616 4.662659 8.087913<br>C 7.030665 3.488688 8.743436<br>H 7.005898 3.528463 9.831730<br>C 7.135228 2.268845 8.111072<br>H 7.202869 1.368567 8.710587<br>C 7.134904 2.228524 6.692976<br>C 7.045507 3.477869 6.031450<br>H 7.051440 3.505012 4.946005<br>C 6.971348 4.693788 6.711830<br>H 7.094540 6.185250 6.440858<br>N 7.217584 1.045029 6.017755<br>Th 6.272183 7.067333 8.816259	
**4′**	C 3.927571 10.330048 6.162747<br>C 4.518618 9.877161 4.946995<br>C 3.588248 10.109849 3.891625<br>C 2.438099 10.749873 4.451995<br>C 2.643959 10.878910 5.857403<br>C 4.611759 10.388725 7.499420<br>H 5.343379 9.582438 7.622359<br>H 3.901264 10.310947 8.329098<br>H 5.157345 11.335748 7.626103<br>C 5.936092 9.416967 4.775503<br>H 6.599519 10.274883 4.588579<br>H 6.053822 8.726902 3.935613<br>H 6.322069 8.905859 5.662729<br>C 3.872168 9.956948 2.424434<br>H 4.346487 10.862065 2.015950<br>H 2.961749 9.788886 1.836909<br>H 4.554314 9.125161 2.220877<br>C 1.372506 11.465316 3.673740<br>H 1.686666 12.499759 3.467450<br>H 0.423628 11.529694 4.217422<br>H 1.170426 10.999953 2.704522<br>C 1.774985 11.655723 6.802832<br>H 1.906117 11.325355 7.837750<br>H 0.708959 11.565736 6.563452<br>H 2.018684 12.728625 6.770681<br>C -0.025815 6.230868 5.760425<br>C 0.702285 5.660307 4.677576<br>C 0.662761 6.579824 3.586752<br>C -0.097521 7.718682 3.995667<br>C -0.516050 7.505894 5.343827<br>C -0.345440 5.566002 7.067935<br>H -1.292541 5.009638 7.006922<br>H -0.449371 6.294212 7.879020<br>H 0.427204 4.851856 7.371822<br>C 1.260680 4.268395 4.624966<br>H 1.319646 3.820955 5.622180<br>H 2.269409 4.236808 4.200112<br>H 0.623367 3.612358 4.014351<br>C 1.097851 6.284854 2.179737<br>H 1.871606 5.511757 2.145849<br>H 1.485413 7.166926 1.656465	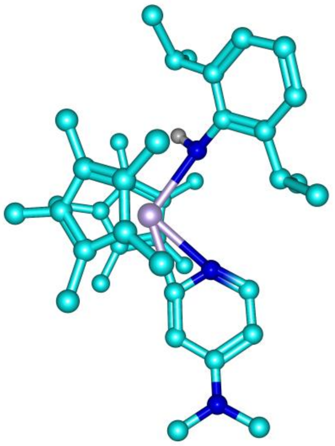<br>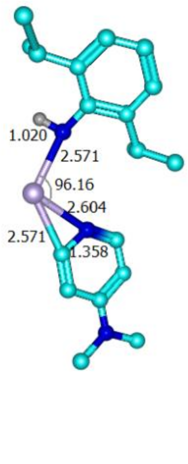

	H 0.254299 5.910812 1.579900<br>C -0.652311 8.754883 3.061535<br>H 0.027853 8.979392 2.233201<br>H -0.884707 9.697545 3.565161<br>H -1.589623 8.396051 2.610054<br>C -1.474580 8.365833 6.116264<br>H -2.510187 8.015515 5.991329<br>H -1.452312 9.409919 5.784213<br>H -1.258645 8.358389 7.189775<br>C 5.391366 5.934468 4.402030<br>C 6.243307 5.680835 5.523083<br>C 7.591782 5.384141 5.323798<br>H 8.224753 5.199915 6.188513<br>C 8.150194 5.291844 4.052256<br>H 9.203875 5.057759 3.920837<br>C 7.320257 5.484819 2.953766<br>H 7.740572 5.392171 1.954781<br>C 5.967478 5.802066 3.092310<br>C 5.659860 5.606196 6.924488<br>H 4.840450 6.331006 6.990713<br>C 6.653933 5.938076 8.042463<br>H 7.423849 5.164705 8.155688<br>H 6.134881 6.008992 9.006291<br>H 7.163958 6.893032 7.867714<br>C 5.052659 4.211161 7.150118<br>H 4.281857 3.996241 6.402807<br>H 4.596067 4.121176 8.145197<br>H 5.828457 3.438914 7.069765<br>C 5.113733 5.943810 1.839185<br>H 4.291244 6.637425 2.066318<br>C 5.847814 6.546079 0.634903<br>H 6.362046 7.478134 0.895986<br>H 5.134155 6.765487 -0.169294<br>H 6.593593 5.858132 0.218847<br>C 4.494543 4.591330 1.447751<br>H 5.283565 3.879308 1.174844<br>H 3.819219 4.690607 0.587532<br>H 3.931473 4.145572 2.276008<br>C 1.741247 8.280869 8.011972<br>C 1.176152 8.734909 9.201873<br>H 0.437130 9.529217 9.165687<br>C 1.558089 8.184183 10.450372<br>C 2.524084 7.143528 10.415084<br>H 2.866450 6.658105 11.321496<br>C 3.042629 6.738821 9.203237<br>H 3.781829 5.943387 9.161095<br>N 4.059980 6.277829 4.590426<br>N 2.673799 7.293549 8.035868<br>Th 2.364747 7.961834 5.538447<br>H 3.571771 6.120366 3.709191<br>N 1.034629 8.622293 11.632395<br>C 1.496315 8.063527 12.888539<br>H 1.298117 6.985047 12.950179<br>H 2.573223 8.225095 13.037368<br>H 0.967682 8.546695 13.711728<br>C 0.077355 9.710782 11.640575<br>H -0.230542 9.910909 12.668343<br>H 0.505409 10.635032 11.227502<br>H -0.821817 9.461119 11.061948	

Table S9. Frequencies of the stationary points optimized for **4** ⇌ **4′**.

Species	Frequencies (cm^{-1})
**4**	11 23 29 30 36 41 48 53 68 72 74 79 84 87 91 96 98 106 112 114<br>118 122 123 133 134 137 139 141 144 149 152 153 159 162 168 174 183 184 191 197<br>201 207 217 227 237 242 253 261 263 267 268 271 274 277 284 284 289 291 294 299<br>301 302 306 322 324 348 362 379 386 404 433 441 443 449 490 548 548 549 551 555<br>557 558 559 562 567 592 607 609 613 618 626 634 634 651 677 719 752 757 783 813<br>820 820 821 824 826 829 837 896 913 915 926 932 935 957 968 969 970 971 979 981<br>982 983 1003 1029 1047 1050 1051 1054 1060 1060 1061 1063 1064 1067 1067 1076 1091 1091 1093 1093<br>1094 1103 1129 1130 1132 1135 1140 1150 1151 1155 1174 1187 1191<br>1193 1194 1196 1198 1207 1230 1251 1269 1290 1294 1310 1355 1370 1373 1374 1375 1392<br>1414 1414 1425 1426 1428 1428 1432 1432 1434 1435 1437 1438 1439 1440 1443 1448 1448 1450 1451 1453<br>1467 1474 1475 1488 1489 1492 1496 1497 1499 1500 1501 1502 1504 1505 1505 1507 1509 1509 1509 1510<br>1511 1511 1514 1514 1514 1515 1519 1520 1522 1524 1528 1529 1530 1531 1531 1533 1534 1540<br>1545 1546 1549 1552 1591 1597 1608 1654 1686 3026 3027 3030 3030 3030 3030 3031 3032 3032 3034 3034<br>3038 3041 3041 3044 3048 3067 3072 3080 3082 3087 3091 3093 3095 3097 3099 3102 3102 3104 3106 3109<br>3111 3112 3117 3120 3126 3127 3128 3128 3130 3130 3131 3139 3143 3148 3149 3154 3155<br>3161 3168 3177 3188 3201 3233 3245 3256 3262
**TS4**	-1361 19 24 30 35 39 43 49 58 63 69 70 73 77 85 92 96 99 107<br>111 115 119 120 124 129 131 135 140 144 147 148 153 157 162 166 169 176 177 179<br>181 188 195 198 210 226 233 244 248 257 263 267 270 272 278 280 282 287 290 292<br>296 296 301 312 315 324 346 354 379 405 411 414 430 440 446 458 497 547 550 551<br>552 555 556 557 560 566 570 588 606 608 611 622 632 640 652 656 693 716 756 761<br>796 814 815 815 820 821 824 825 869 884 906 913 931 933 939 956 966 968 968 971<br>972 973 975 985 1019 1047 1049 1051 1053 1059 1060 1061 1061 1065 1067 1067 1074 1077 1090 1091<br>1092 1092 1093 1127 1129 1133 1138 1141 1148 1151 1170 1175 1187 1191<br>1192 1194 1195 1198 1205 1212 1244 1266 1287 1293 1298 1305 1337 1351 1360 1364 1387<br>1414 1415 1415 1422 1423 1426 1427 1430 1433 1434 1435 1437 1438 1439 1441 1445 1448 1450 1454 1456<br>1464 1469 1473 1475 1488 1490 1490 1491 1494 1495 1497 1499 1502 1503 1503 1504 1505 1505 1507 1507<br>1509 1510 1510 1511 1511 1513 1514 1515 1521 1524 1524 1527 1528 1529 1530 1531 1531 1533 1542 1546<br>1548 1553 1555 1585 1616 1648 1655 1717 3029 3032 3033 3033 3034 3034 3035 3036 3036 3037 3040 3040<br>3040 3040 3045 3048 3061 3089 3089 3091 3091 3091 3094 3095 3096 3098 3108 3108 3109 3109 3109 3114<br>3115 3119 3121 3127 3128 3129 3130 3133 3138 3142 3145 3146 3148 3148 3150 3151 3168 3169<br>3177 3178 3179 3205 3217 3246
**4′**	7 22 26 28 37 38 49 56 64 69 71 78 83 86 91 101 104 107 108 114<br>117 121 127 131 133 135 139 143 145 149 152 156 161 163 169 175 177 179 187 201<br>208 213 227 230 240 250 257 270 274 276 277 281 284 285 286 289 291 293 295 298<br>298 301 306 315 350 369 378 379 390 407 416 444 446 450 491 522 544 547 548 551<br>552 556 557 560 562 567 572 598 609 610 621 626 634 643 645 690 694 752 768 787<br>805 818 819 822 824 826 826 854 868 903 907 929 933 935 949 970 972 972 976 977<br>979 980 985 1010 1049 1050 1053 1054 1058 1059 1061 1061 1062 1063 1072 1080 1083 1092 1093 1093<br>1094 1094 1128 1129 1131 1142 1149 1149 1153 1173 1177 1186 1191<br>1192 1195 1197 1199 1213 1238 1256 1289 1297 1298 1307 1336 1345 1354 1368 1375 1412<br>1414 1417 1420 1423 1425 1425 1425 1432 1433 1435 1436 1437 1438 1438 1440 1448 1450 1451 1453 1456<br>1468 1471 1473 1475 1488 1489 1490 1491 1492 1494 1497 1503 1503 1504 1505 1506 1507 1507 1508 1509<br>1511 1512 1513 1514 1515 1518 1519 1524 1525 1527 1527 1529 1530 1531 1532 1532 1533 1533 1543<br>1544 1546 1551 1556 1582 1630 1648 1658 3026 3031 3032 3033 3033 3034 3034 3035 3036 3037 3037 3039<br>3041 3042 3043 3050 3052 3073 3090 3092 3096 3098 3098 3103 3104 3104 3107 3108 3109 3111 3115<br>3118 3118 3120 3126 3127 3133 3135 3138 3139 3141 3142 3143 3144 3145 3148 3152 3158 3170<br>3179 3185 3197 3212 3217 3221 3251 3497

Table S10. The energies (with ZPE correction), enthalpies and free energies (in au at 298K) and corresponding relative values (in kcal/mol) for **4** ⇌ **4′**, obtained with B3PW91-SMD/6-31G(d,p)∪ECP78MWB method.

Species	E	H	G
**4**	-1719.26933 (0.0)	-1719.21599 (0.0)	-1719.34600 (0.0)
**TS4**	-1719.23199 (23.4)	-1719.17879 (23.3)	-1719.30792 (23.9)
**4′**	-1719.26805 (0.8)	-1719.21469 (0.8)	-1719.34519 (0.5)

4. NMR spectra

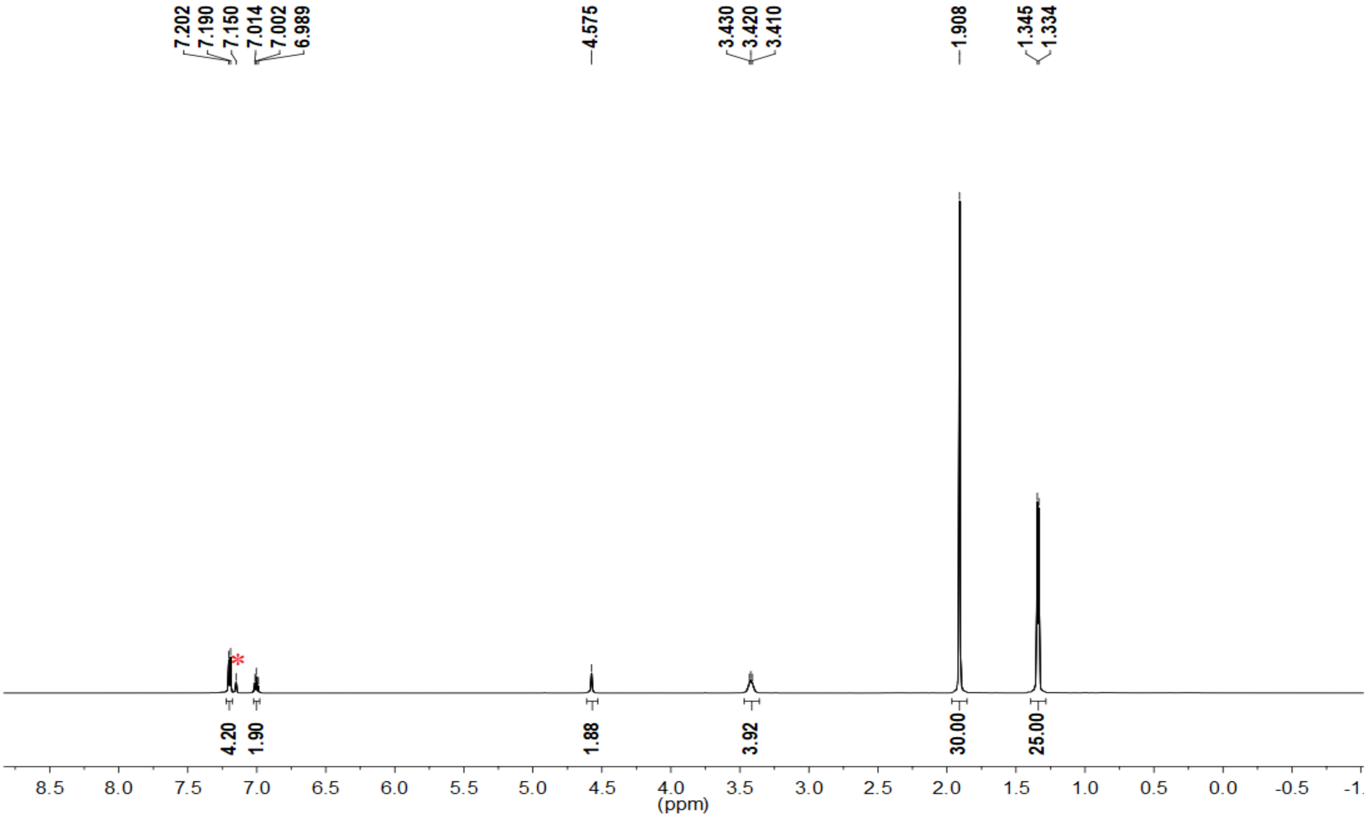

Figure S11. ^{1}H NMR (C_6D_6; 20 ℃) spectrum for compound **2** (* solvent).

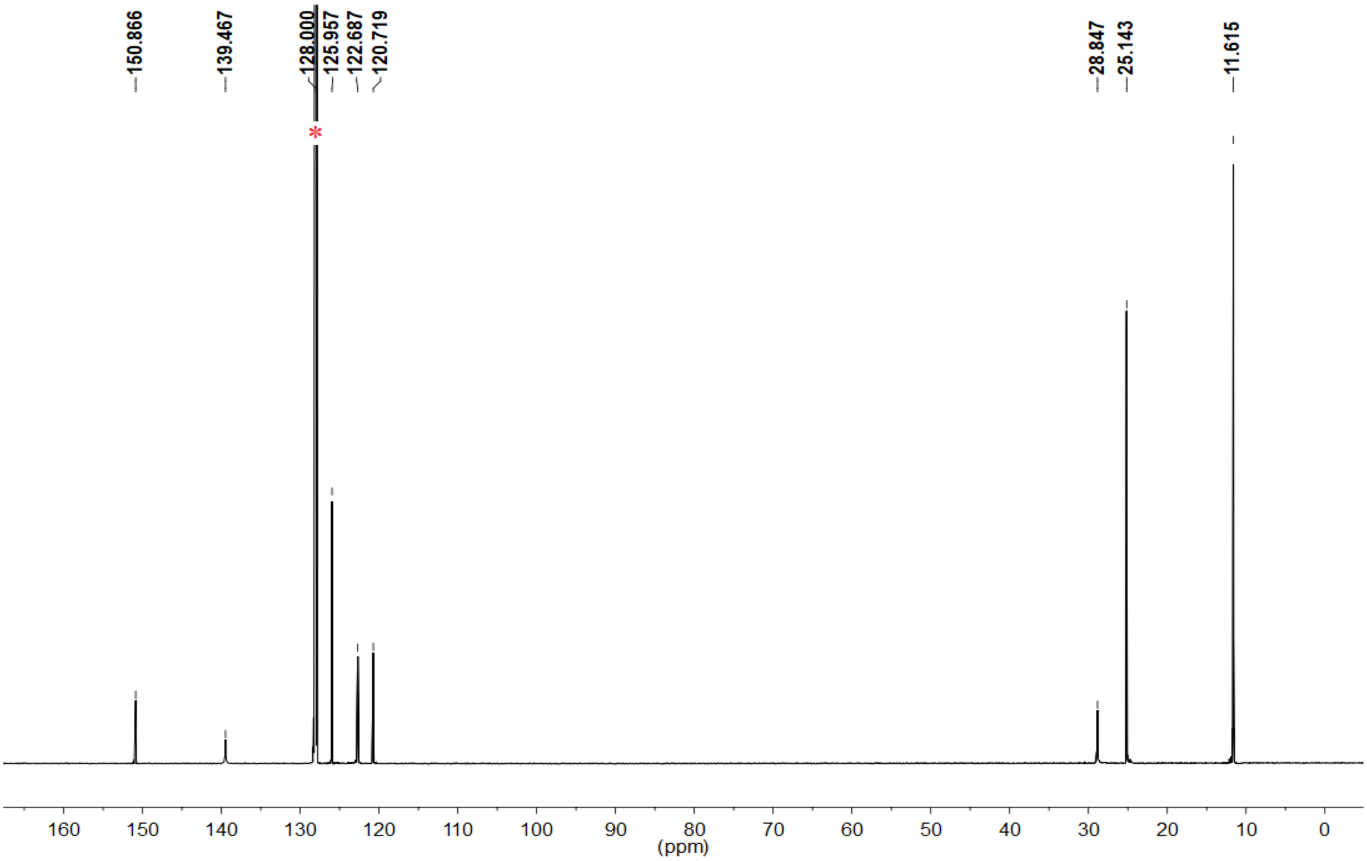

Figure S12. $^{13}C\{^{1}H\}$ NMR (C_6D_6; 20 ℃) spectrum for compound **2** (* solvent).

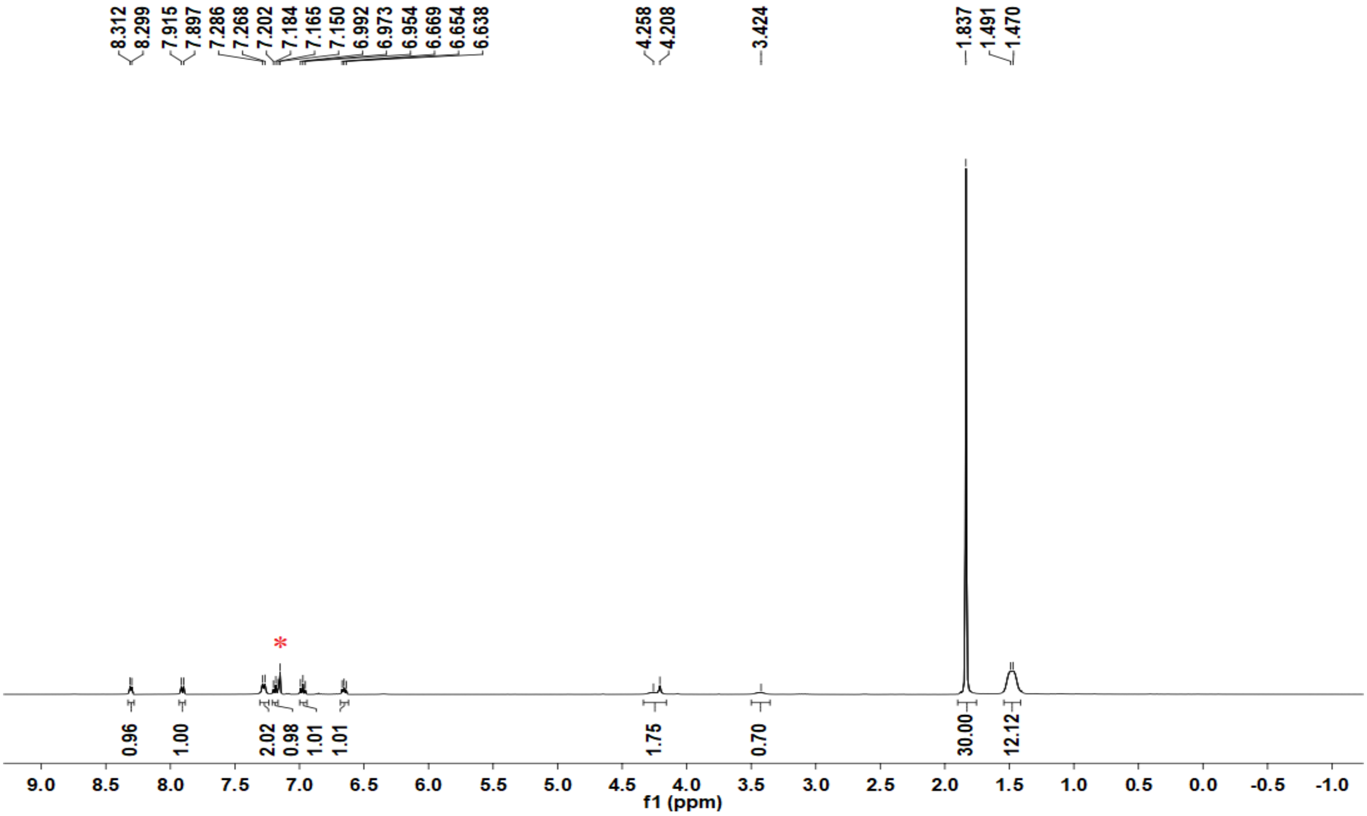

Figure S13. ^{1}H NMR (C_6D_6; 20 ℃) spectrum for compound **3** (* solvent).

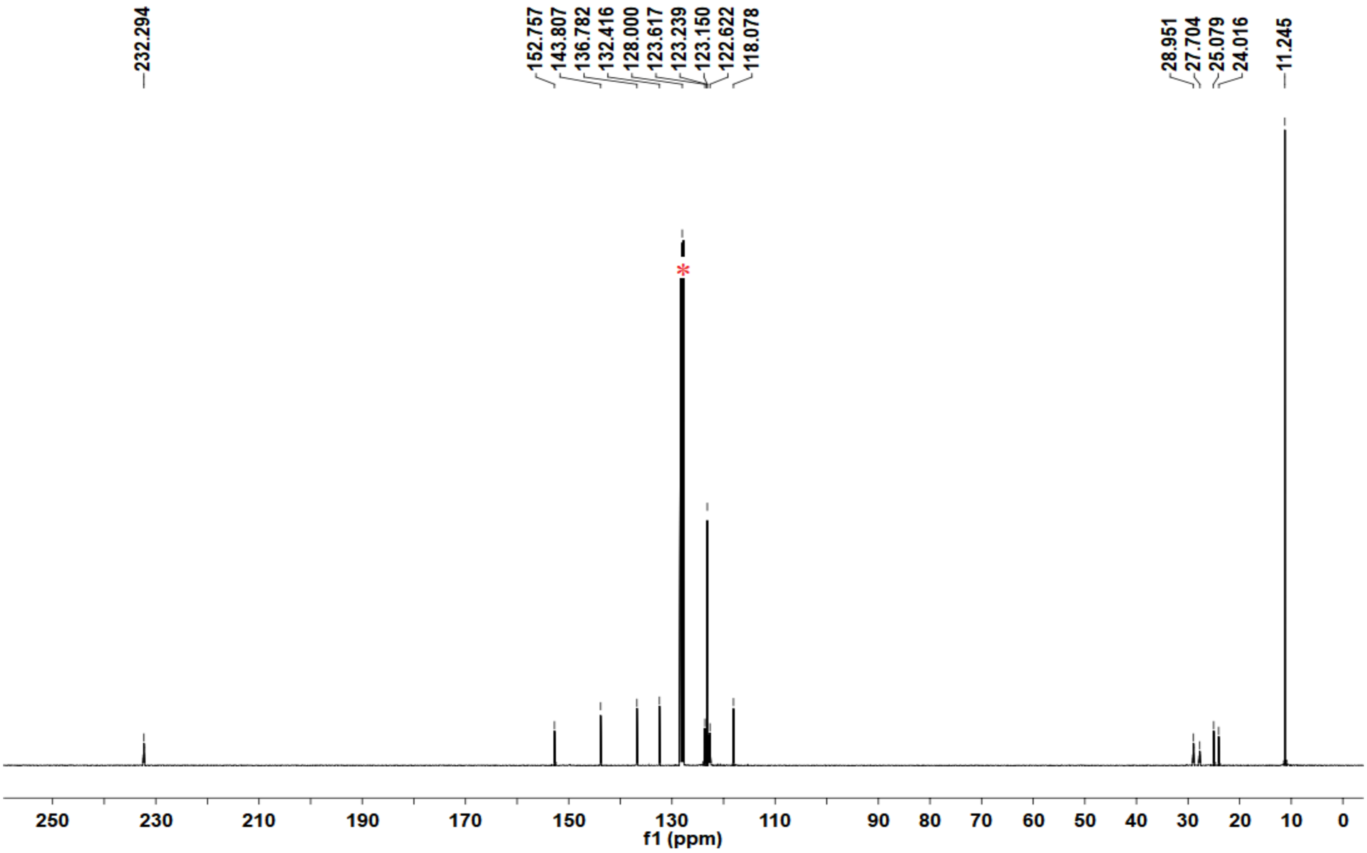

Figure S14. $^{13}C\{^{1}H\}$ NMR (C_6D_6; 20 ℃) spectrum for compound **3** (* solvent).

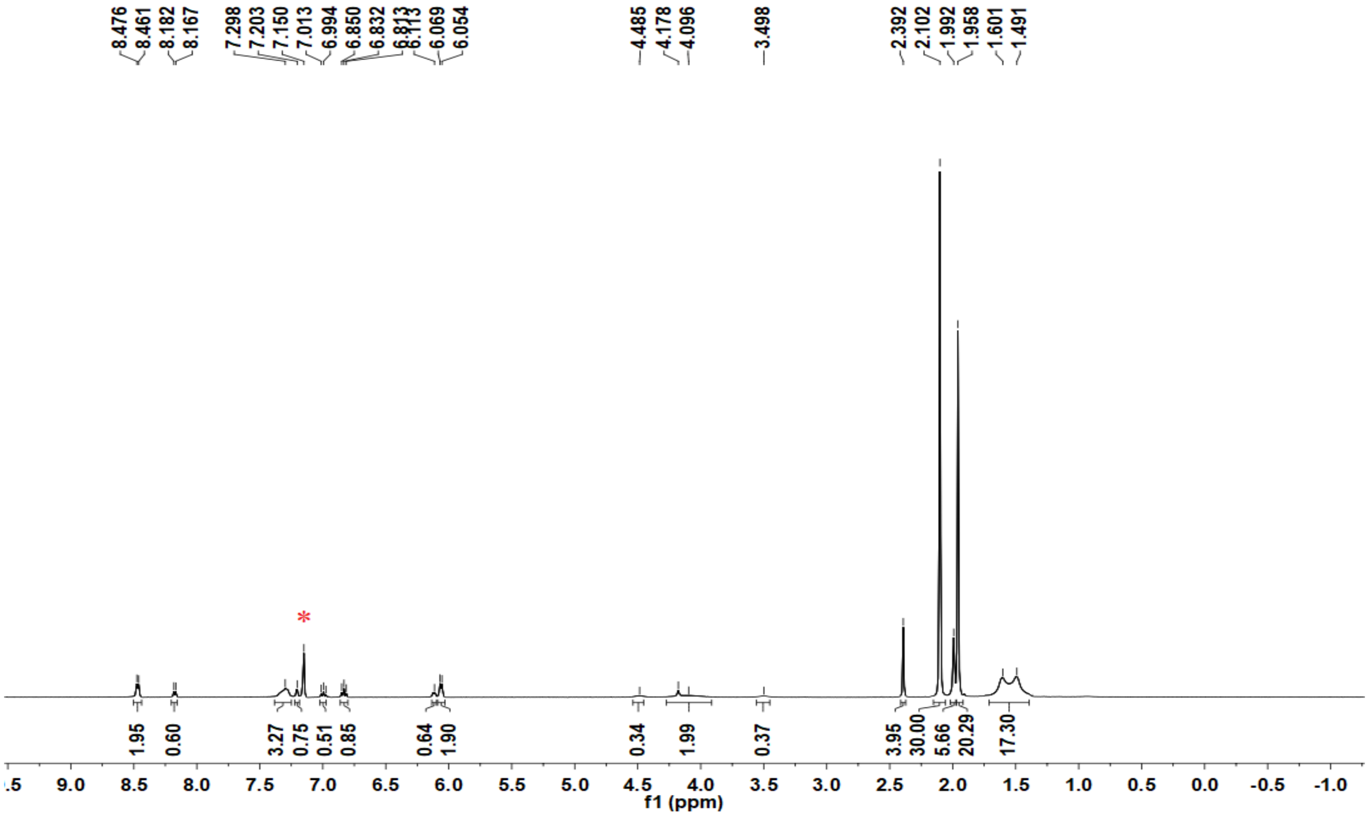

Figure S15. ^{1}H NMR (C_6D_6; 20 ℃) spectrum for compounds **4** and **4′** (ratio: *ca.* 1.5:1) (* solvent).

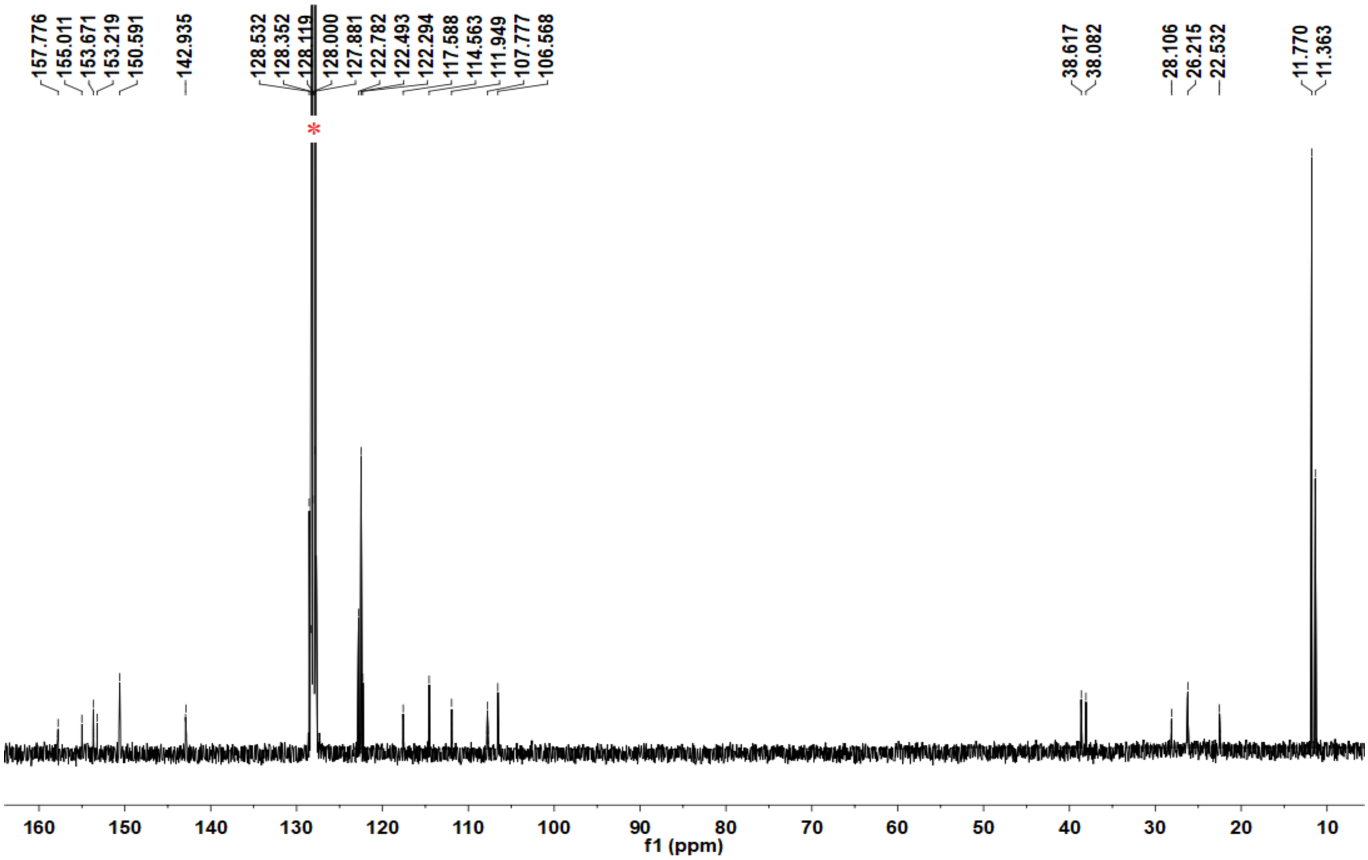

Figure S16. $^{13}C\{^{1}H\}$ NMR (C_6D_6; 20 ℃) spectrum for compounds **4** and **4′** (* solvent).

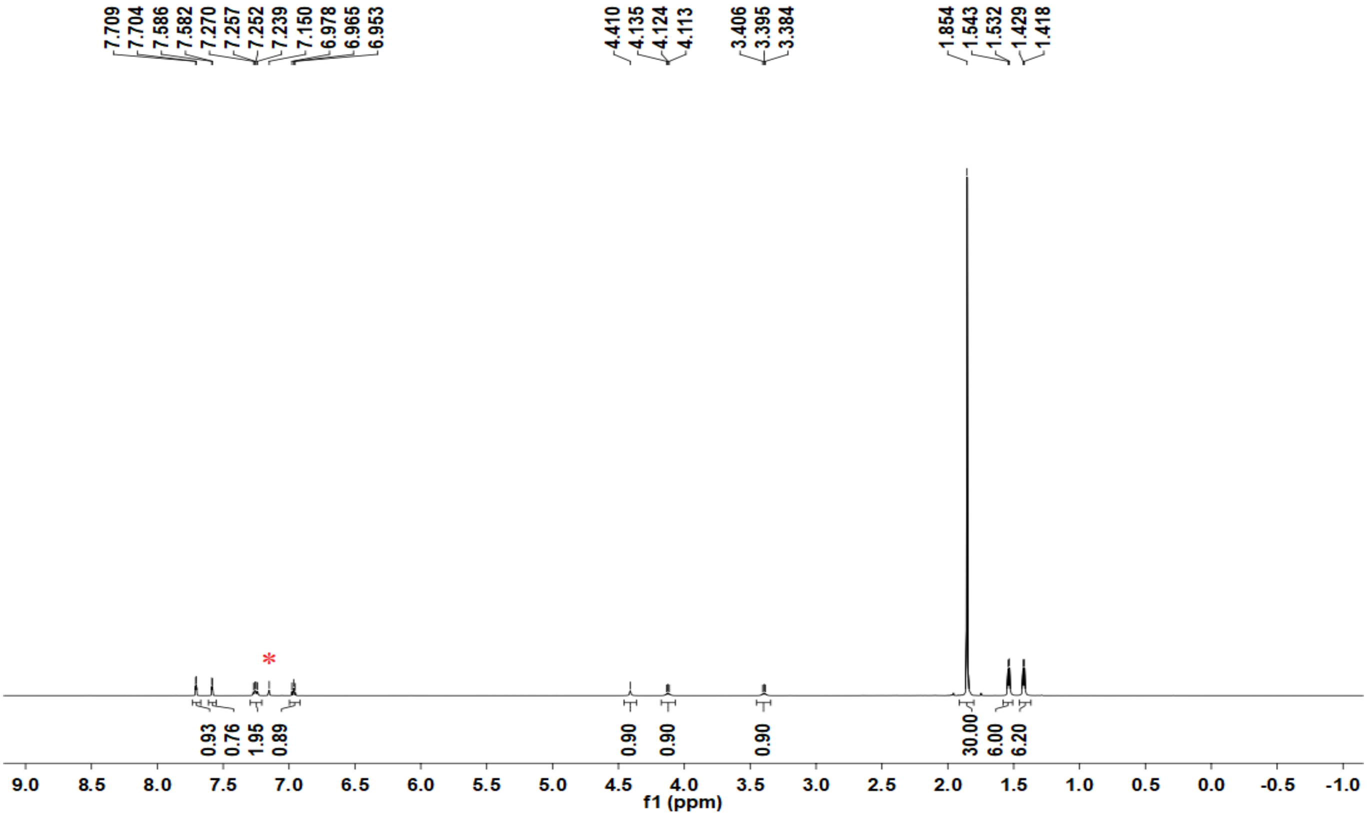

Figure S17. ^{1}H NMR (C_6D_6; 20 ℃) spectrum for compound **6** (* solvent).

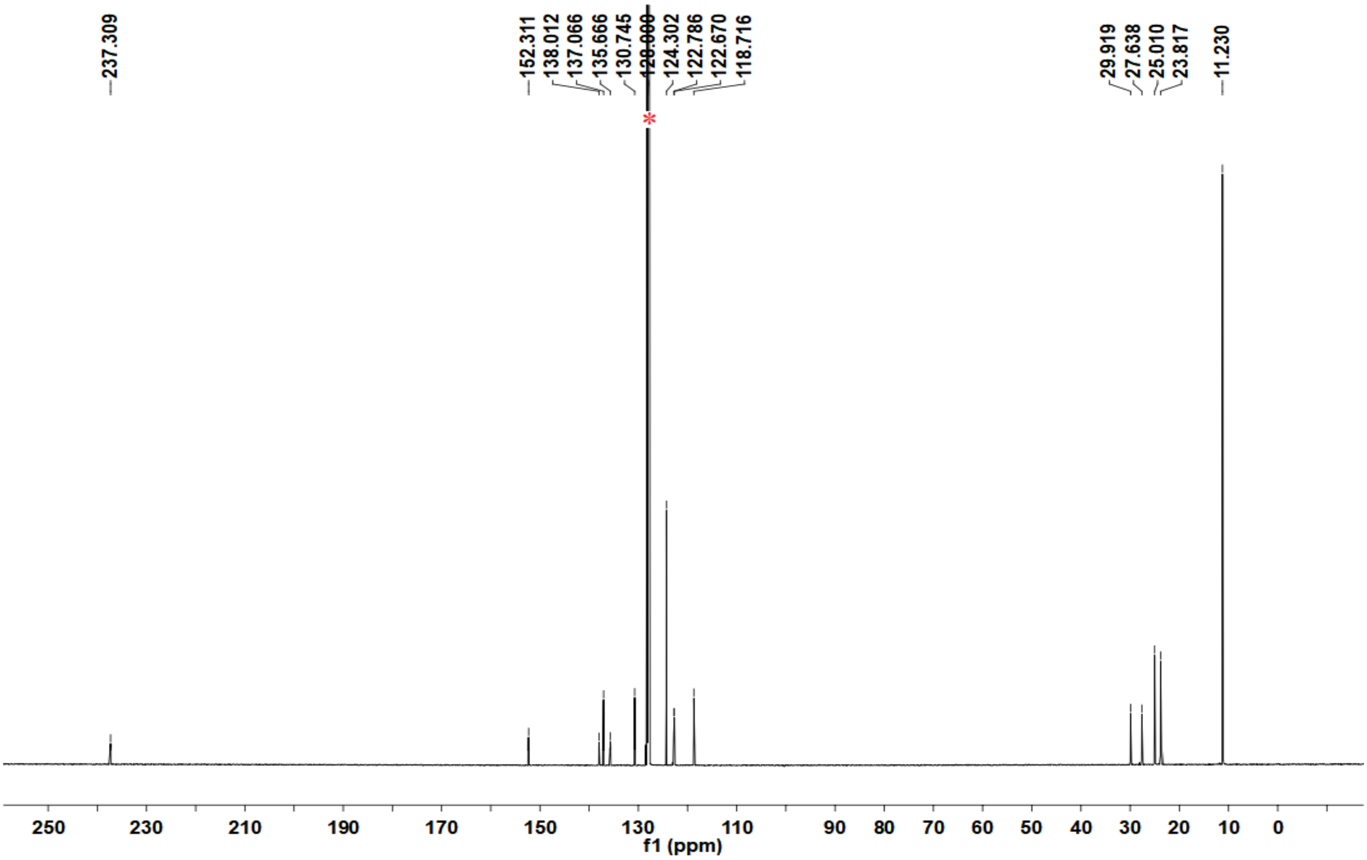

Figure S18. $^{13}C\{^{1}H\}$ NMR (C_6D_6; 20 ℃) spectrum for compound **6** (* solvent).

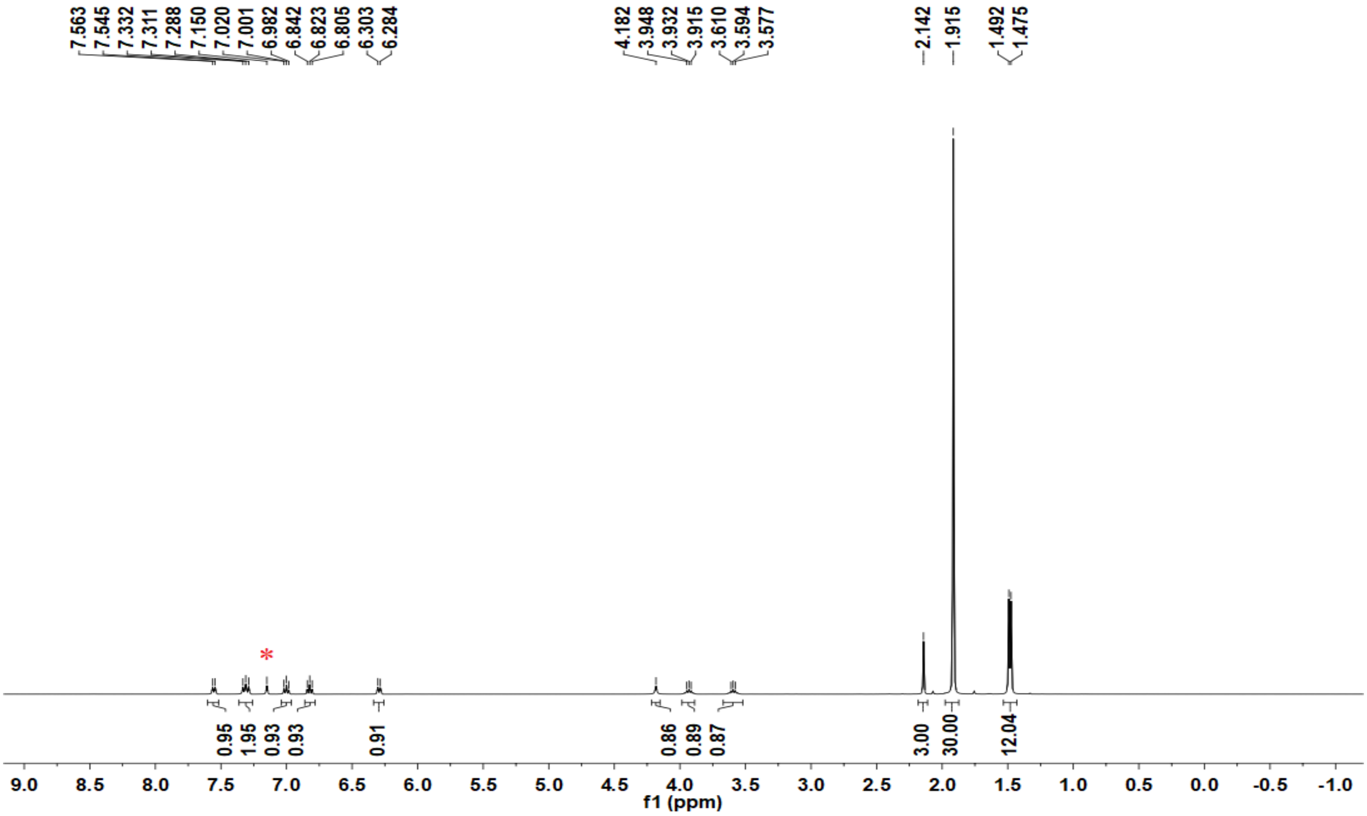

Figure S19. ^{1}H NMR (C_6D_6; 20 ℃) spectrum for compound **7** (* solvent).

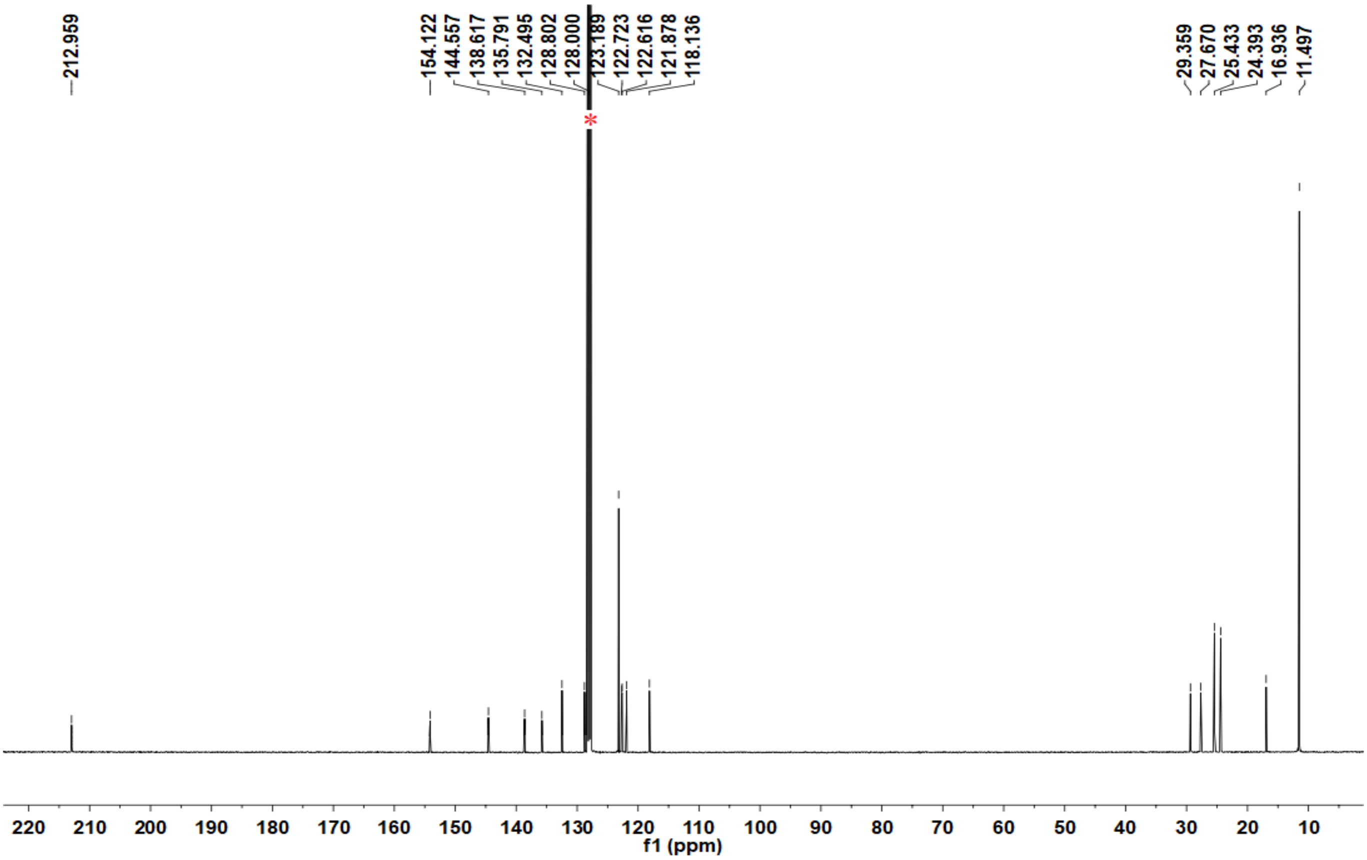

Figure S20. $^{13}C\{^{1}H\}$ NMR (C_6D_6; 20 ℃) spectrum for compound **7** (* solvent).

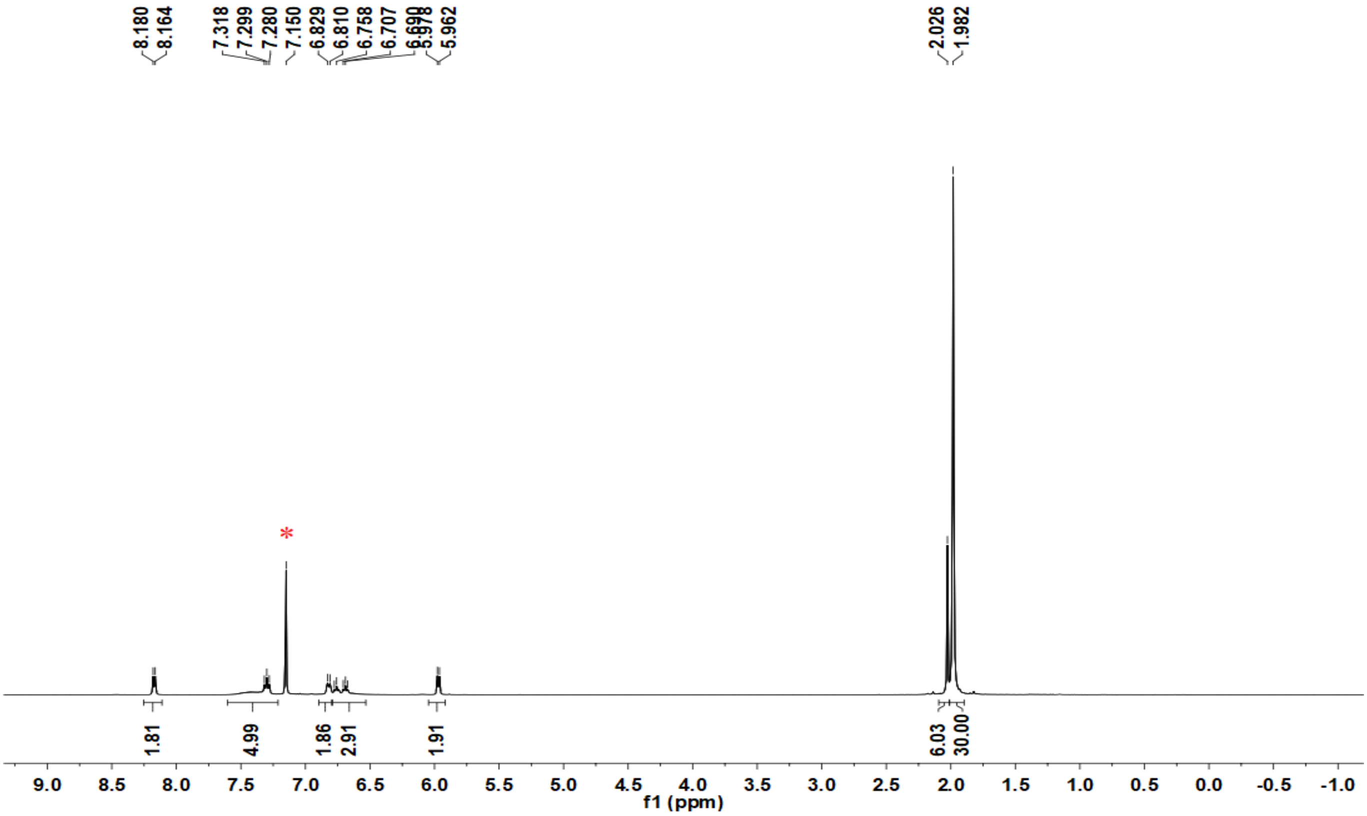

Figure S21. ^{1}H NMR (C_6D_6; 20 ℃) spectrum for compound **9** (* solvent).

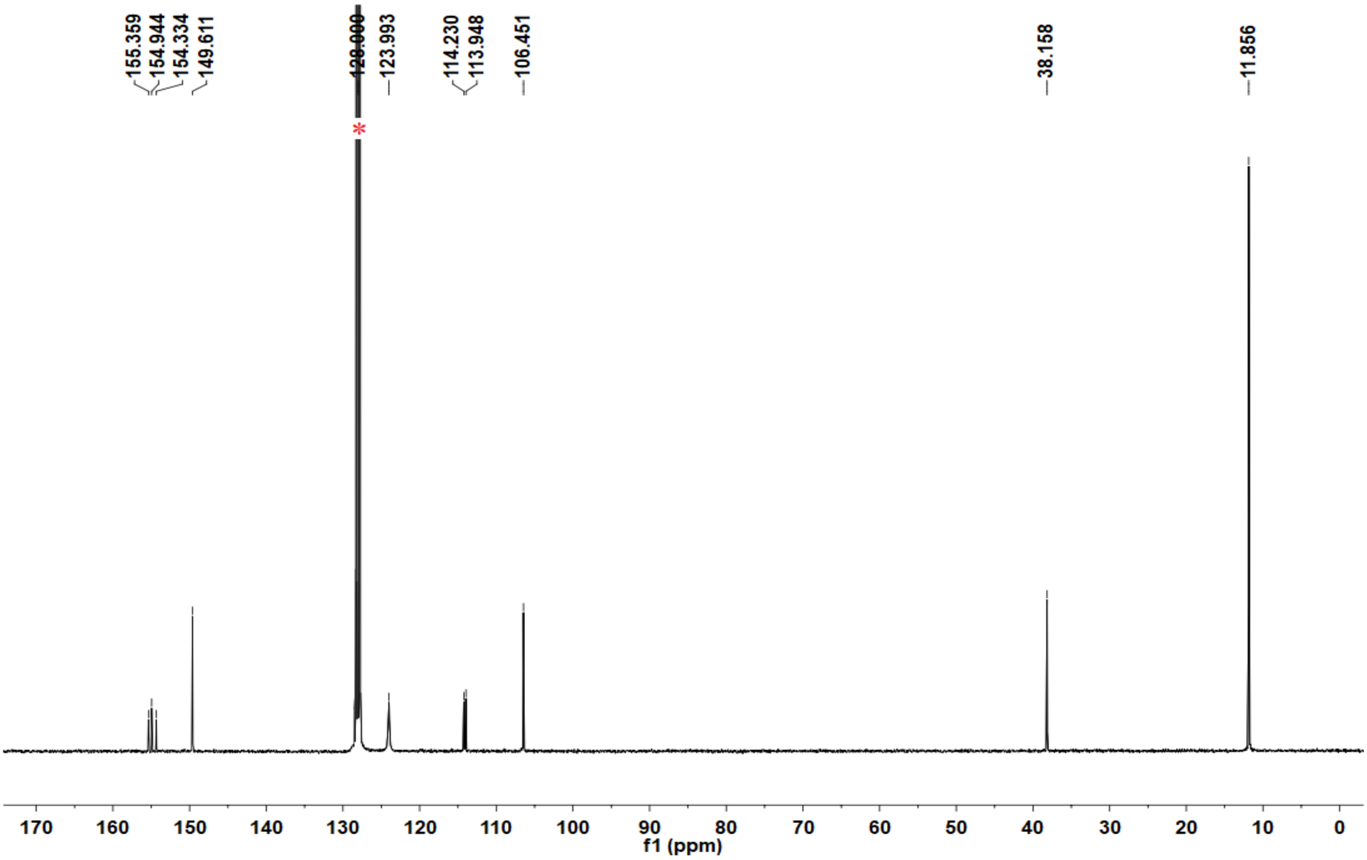

Figure S22. $^{13}C\{^{1}H\}$ NMR (C_6D_6; 20 ℃) spectrum for compound **9** (* solvent).

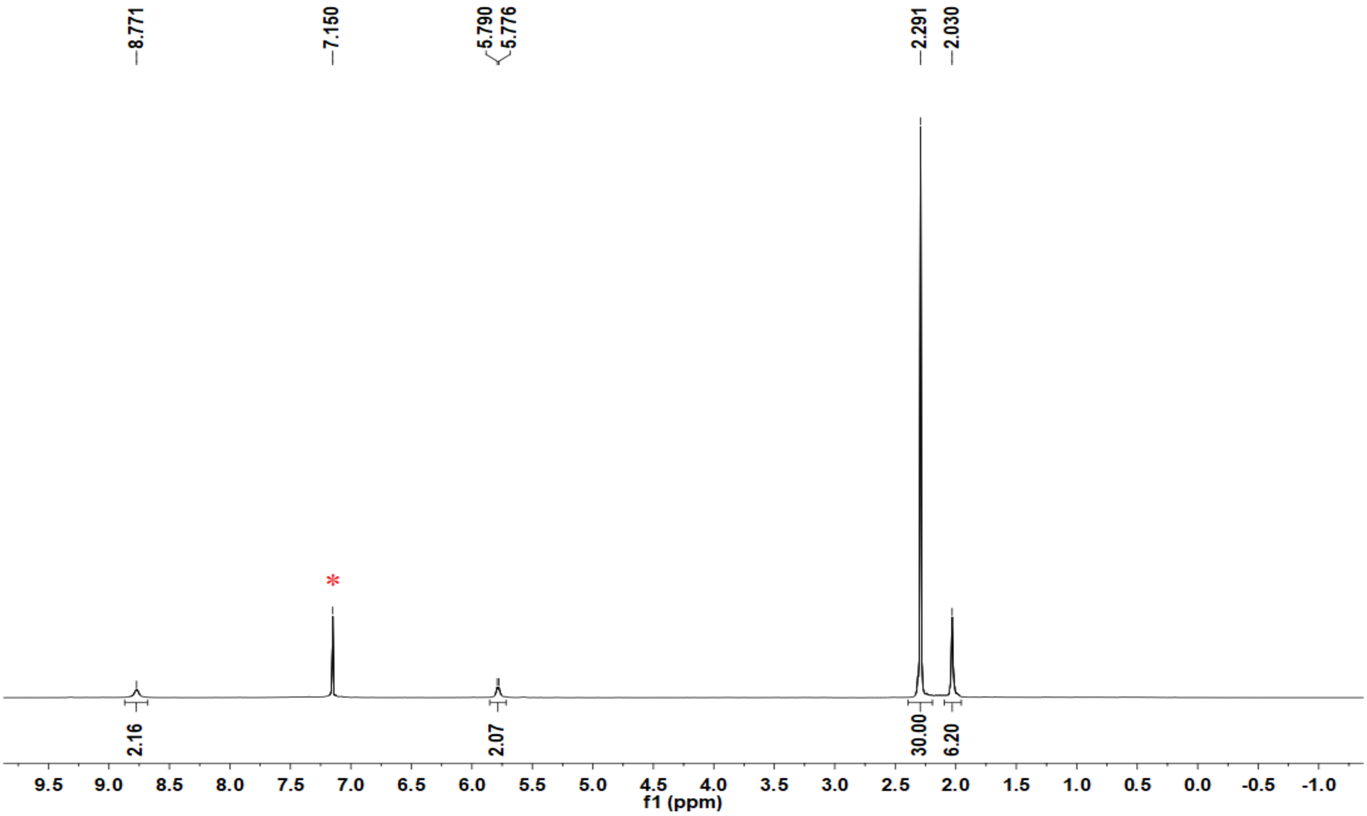

Figure S23. ^{1}H NMR (C_6D_6; 20 ℃) spectrum for compound **10** (* solvent).

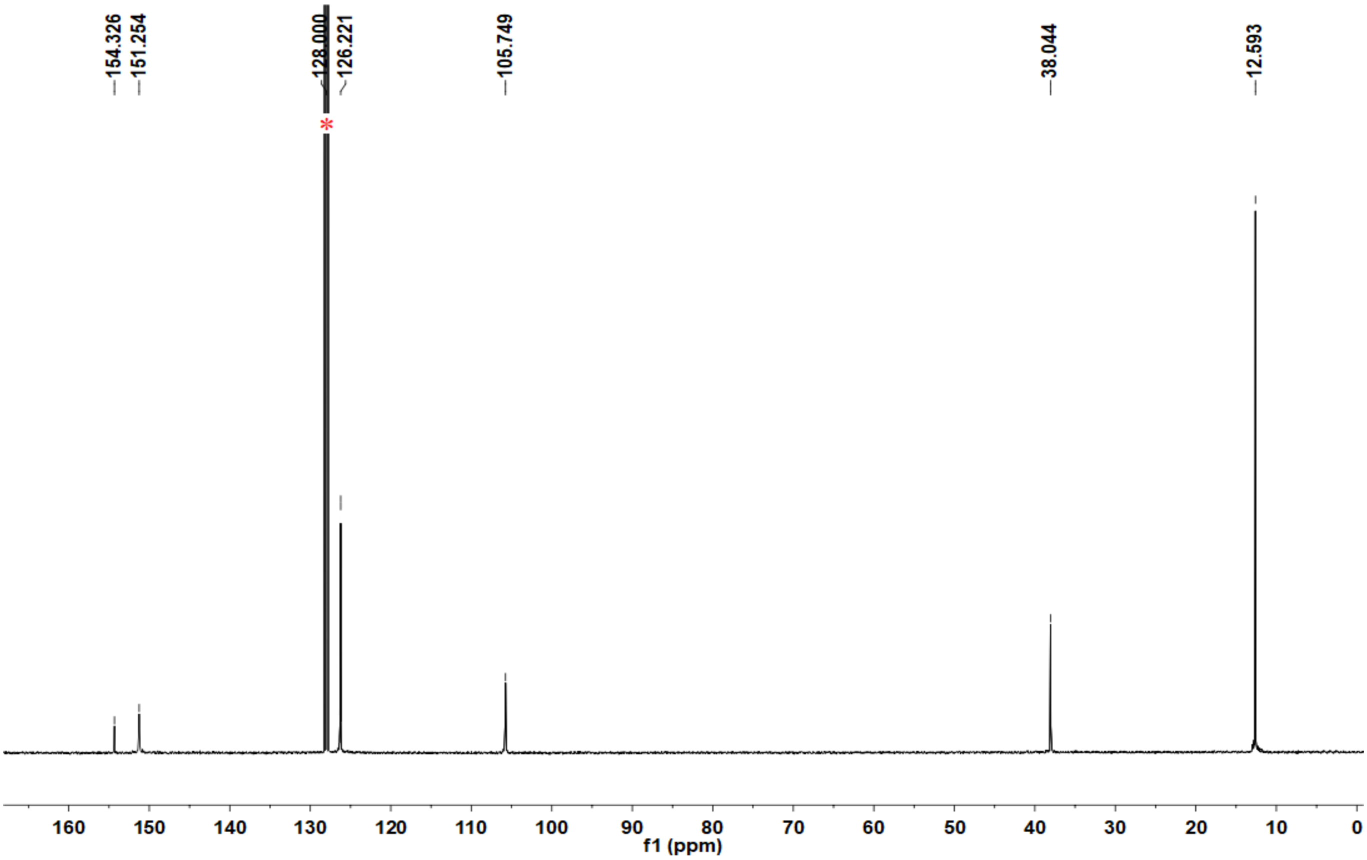

Figure S24. $^{13}C\{^{1}H\}$ NMR (C_6D_6; 20 ℃) spectrum for compound **10** (* solvent).

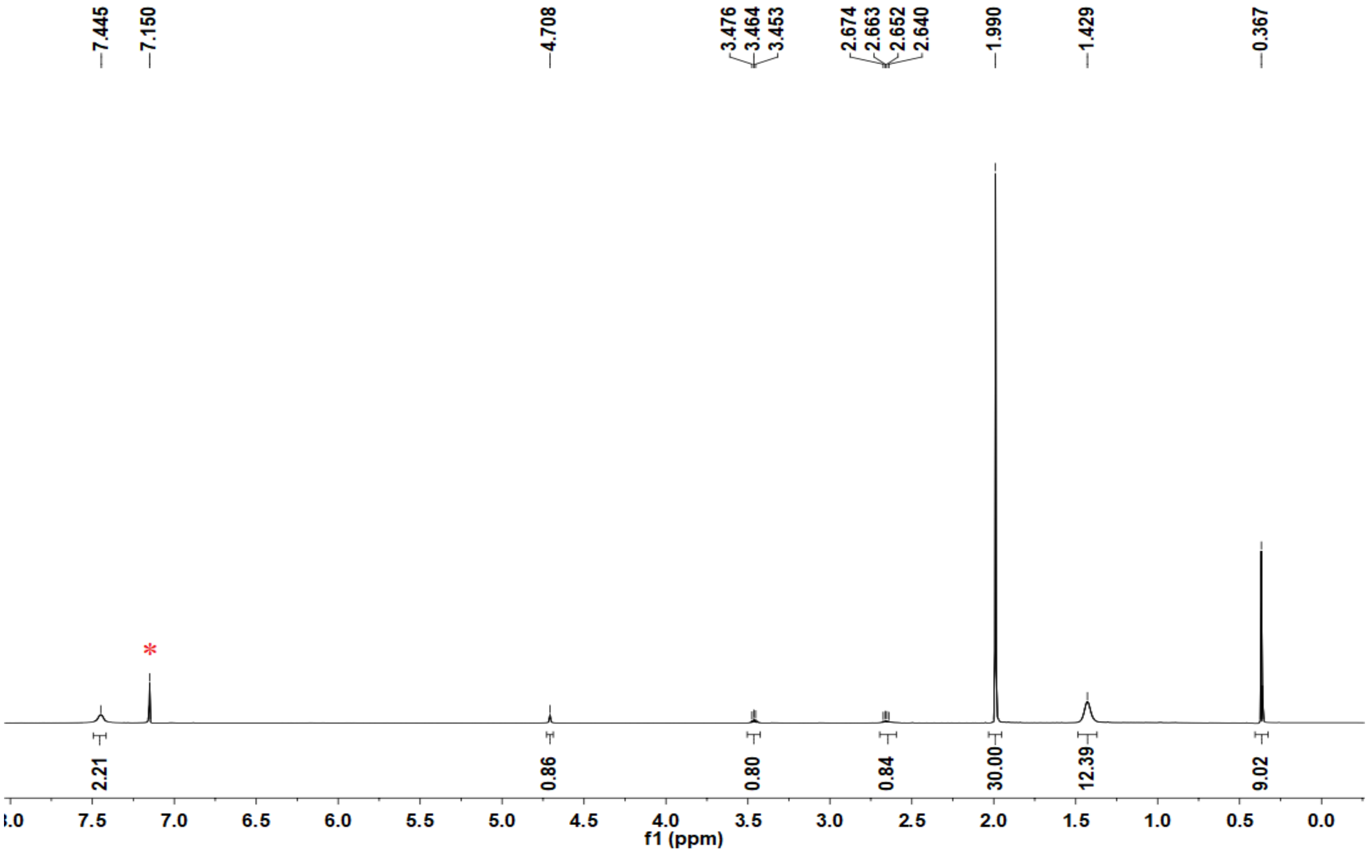

Figure S25. ^{1}H NMR (C_6D_6; 20 ℃) spectrum for compound **11** (* solvent).

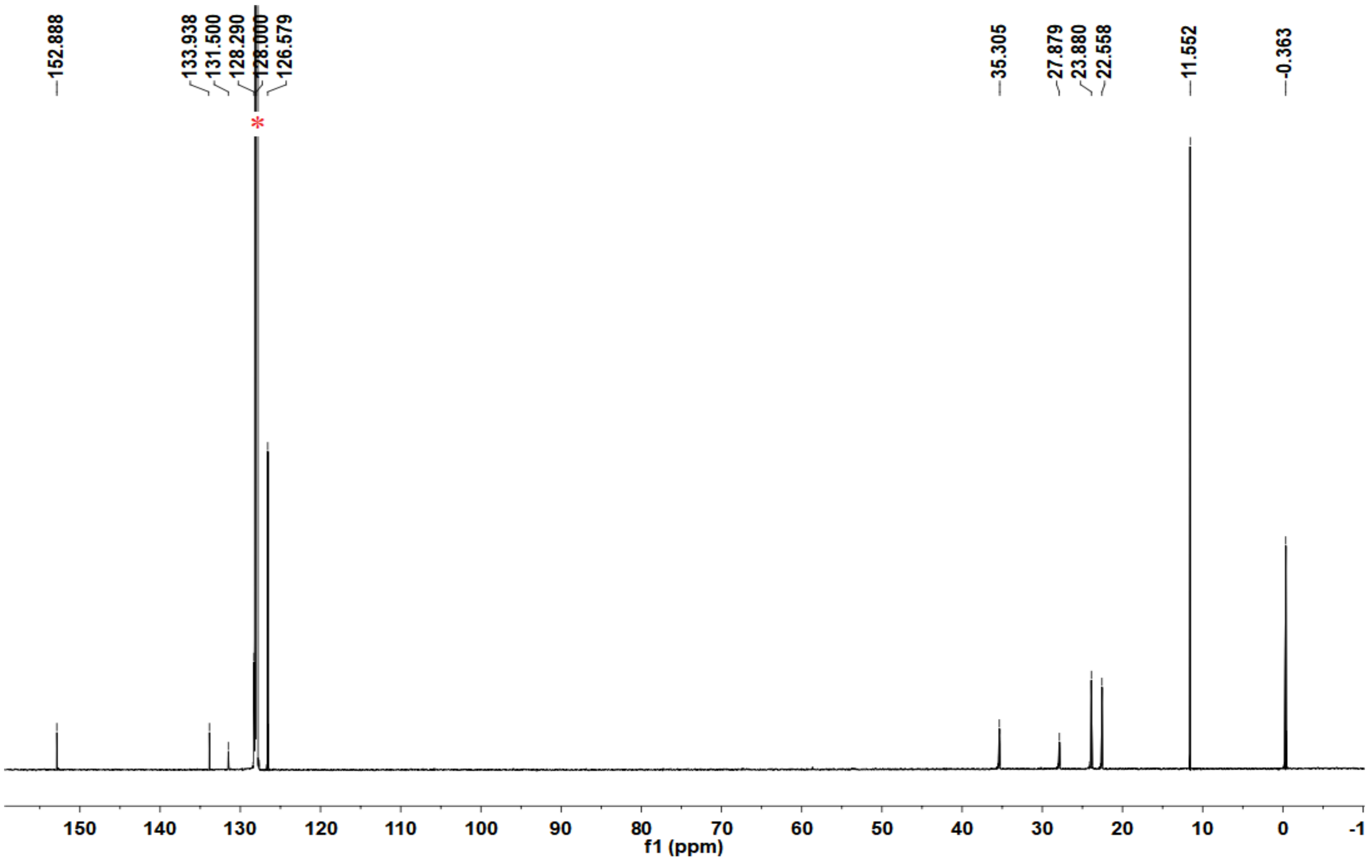

Figure S26. $^{13}C\{^{1}H\}$ NMR (C_6D_6; 20 ℃) spectrum for compound **11** (* solvent).

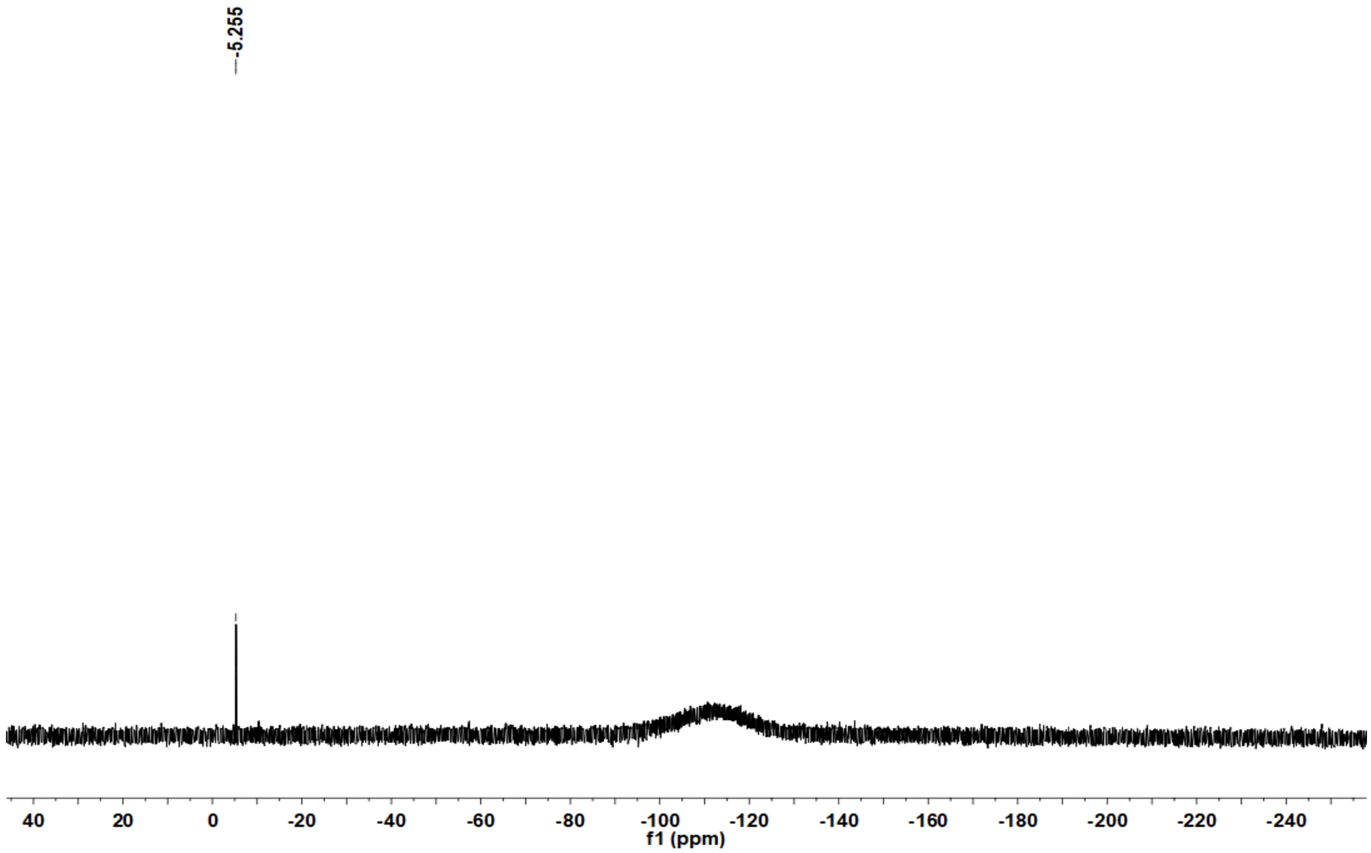

Figure S27. $^{29}Si\{^{1}H\}$ NMR (C_6D_6; 20 ℃) spectrum for compound **11**.

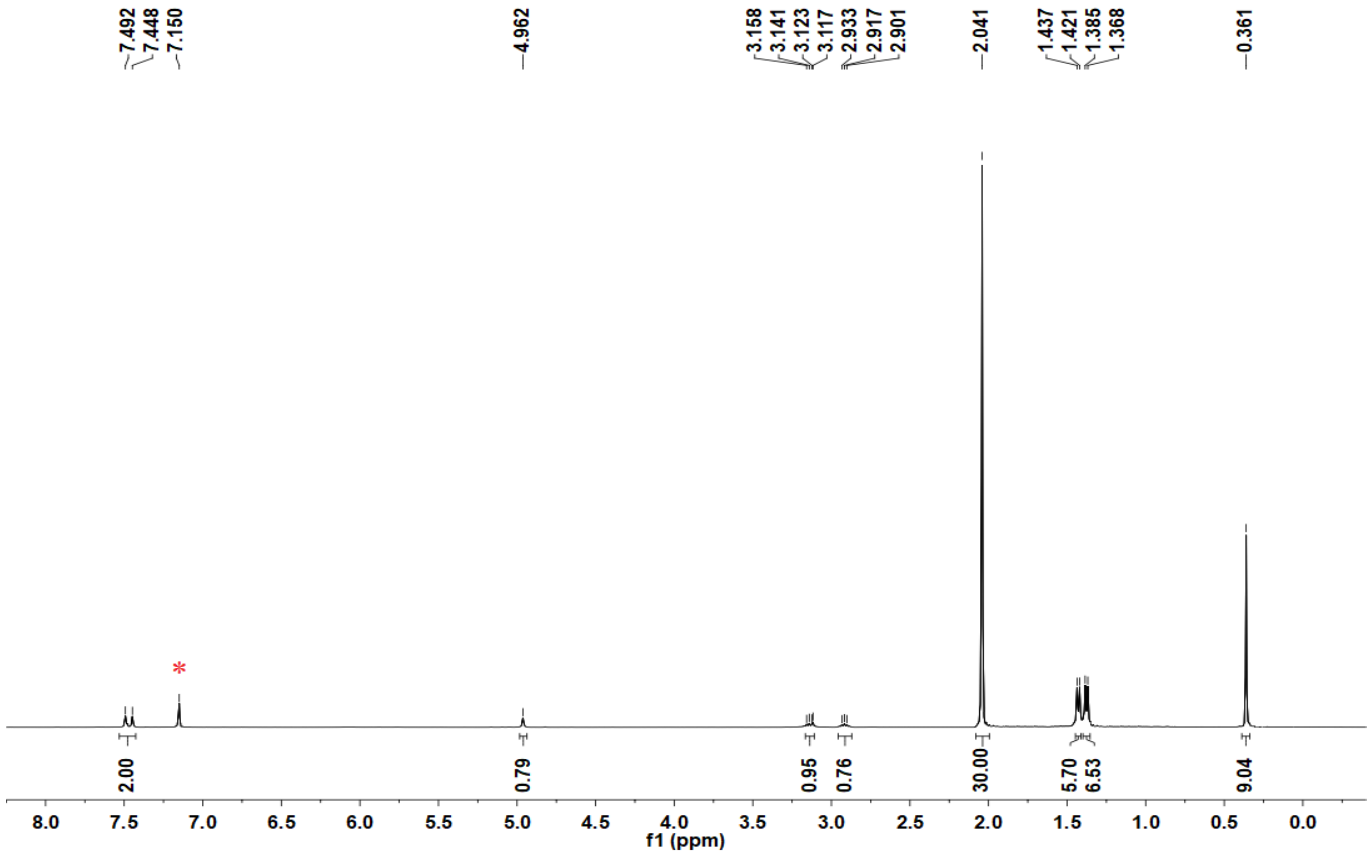

Figure S28. ^{1}H NMR (C_6D_6; 20 ℃) spectrum for compound **12** (* solvent).

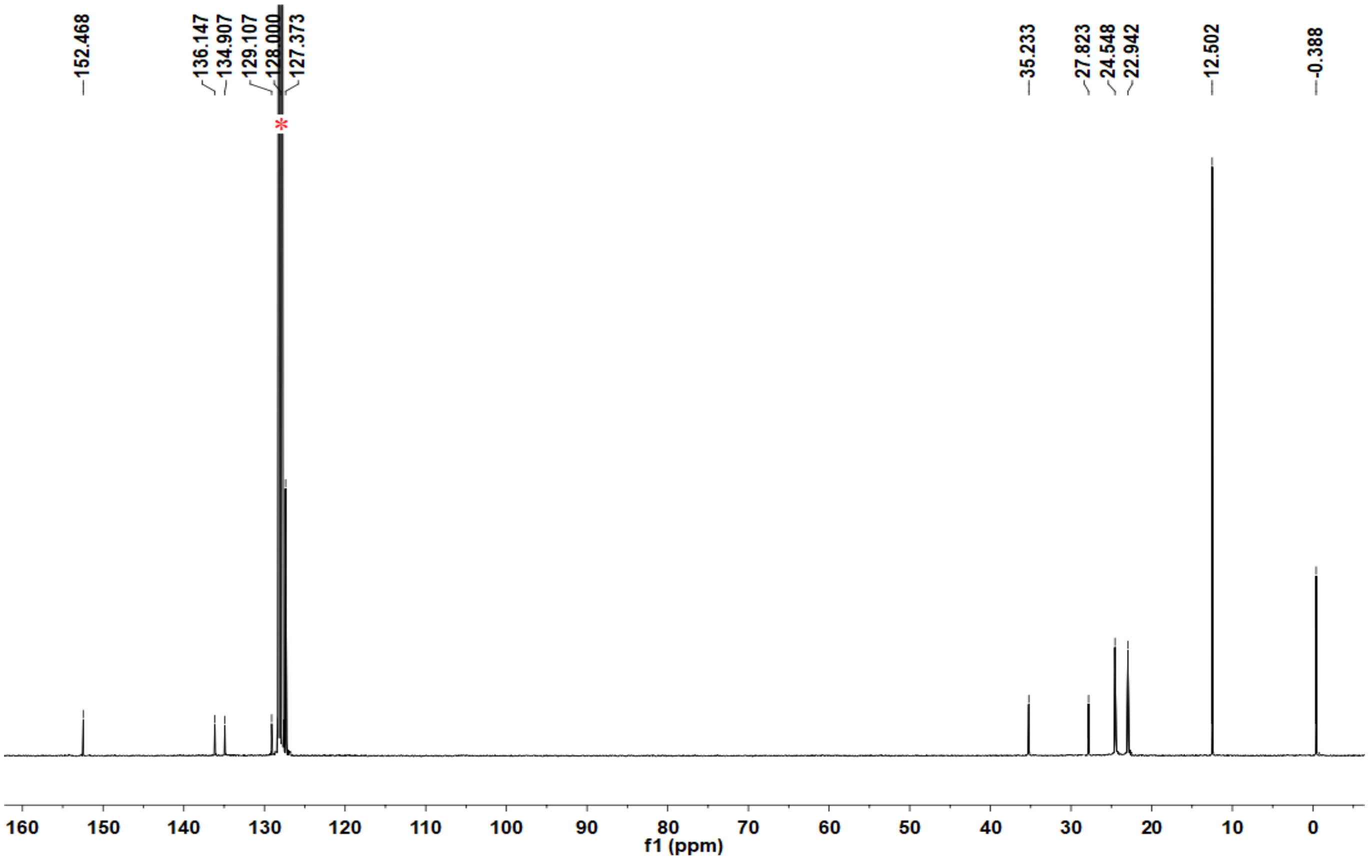

Figure S29. $^{13}C\{^{1}H\}$ NMR (C_6D_6; 20 ℃) spectrum for compound **12** (* solvent).

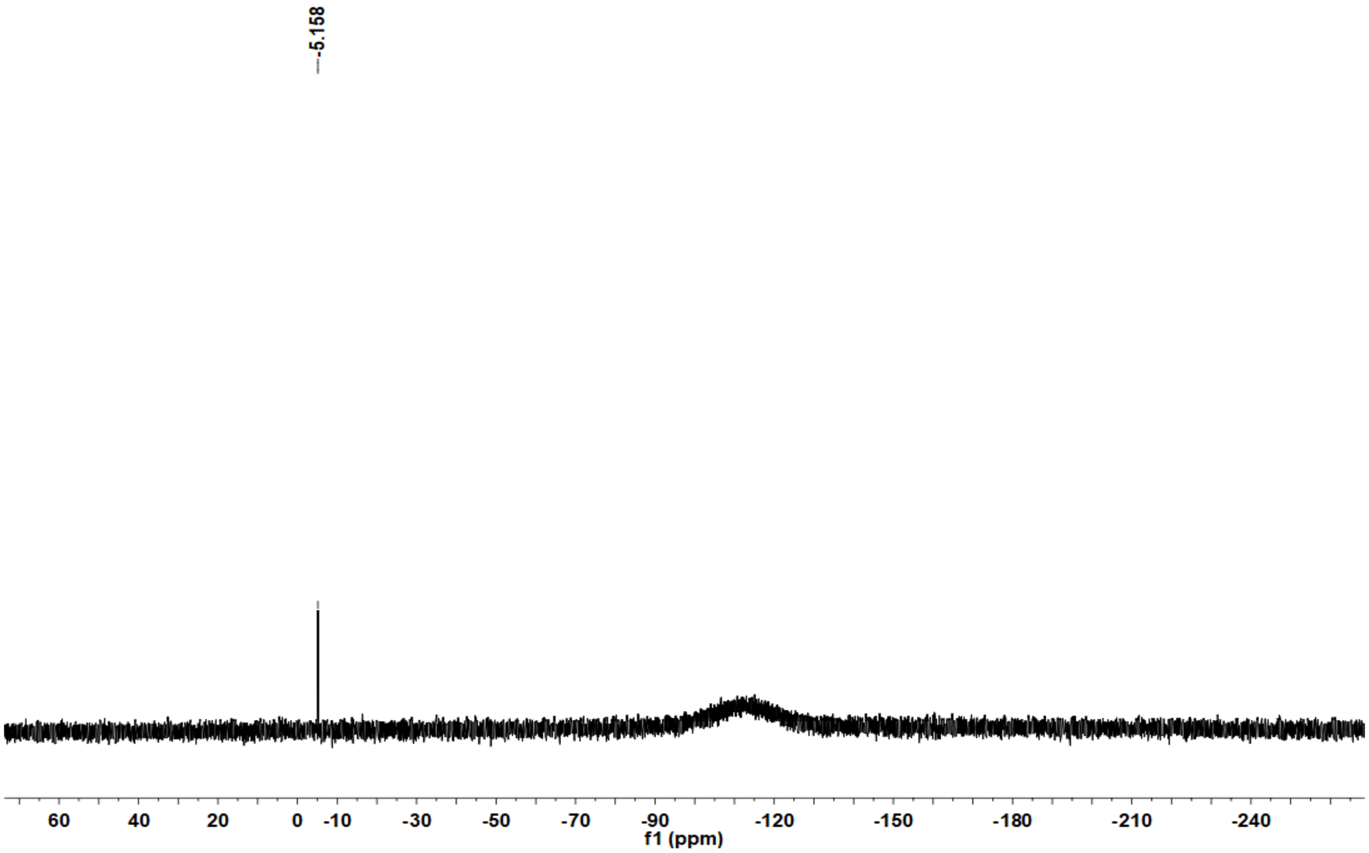

Figure S30. $^{29}Si\{^{1}H\}$ NMR (C_6D_6; 20 ℃) spectrum for compound **12**.

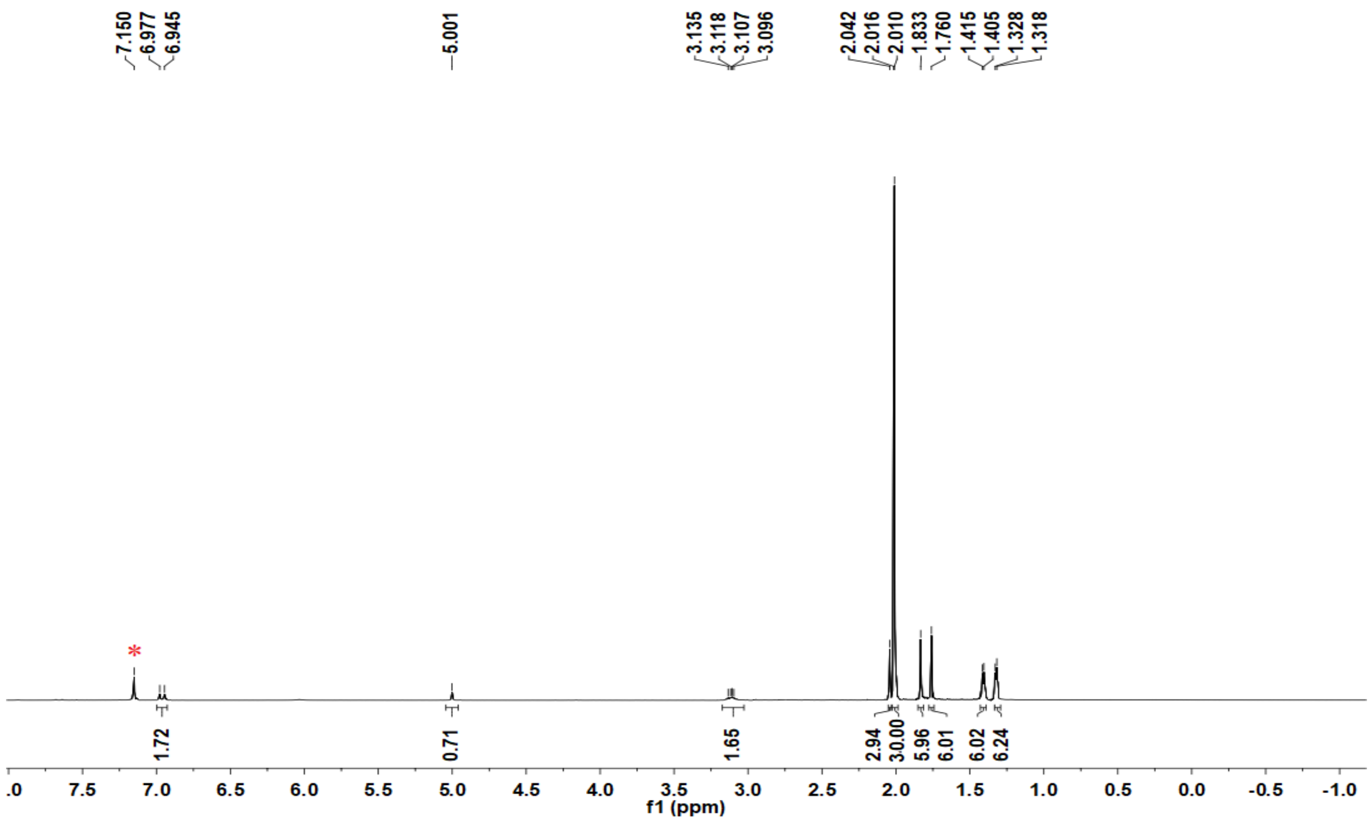

Figure S31. ^{1}H NMR (C_6D_6; 20 ℃) spectrum for compound **13** (* solvent).

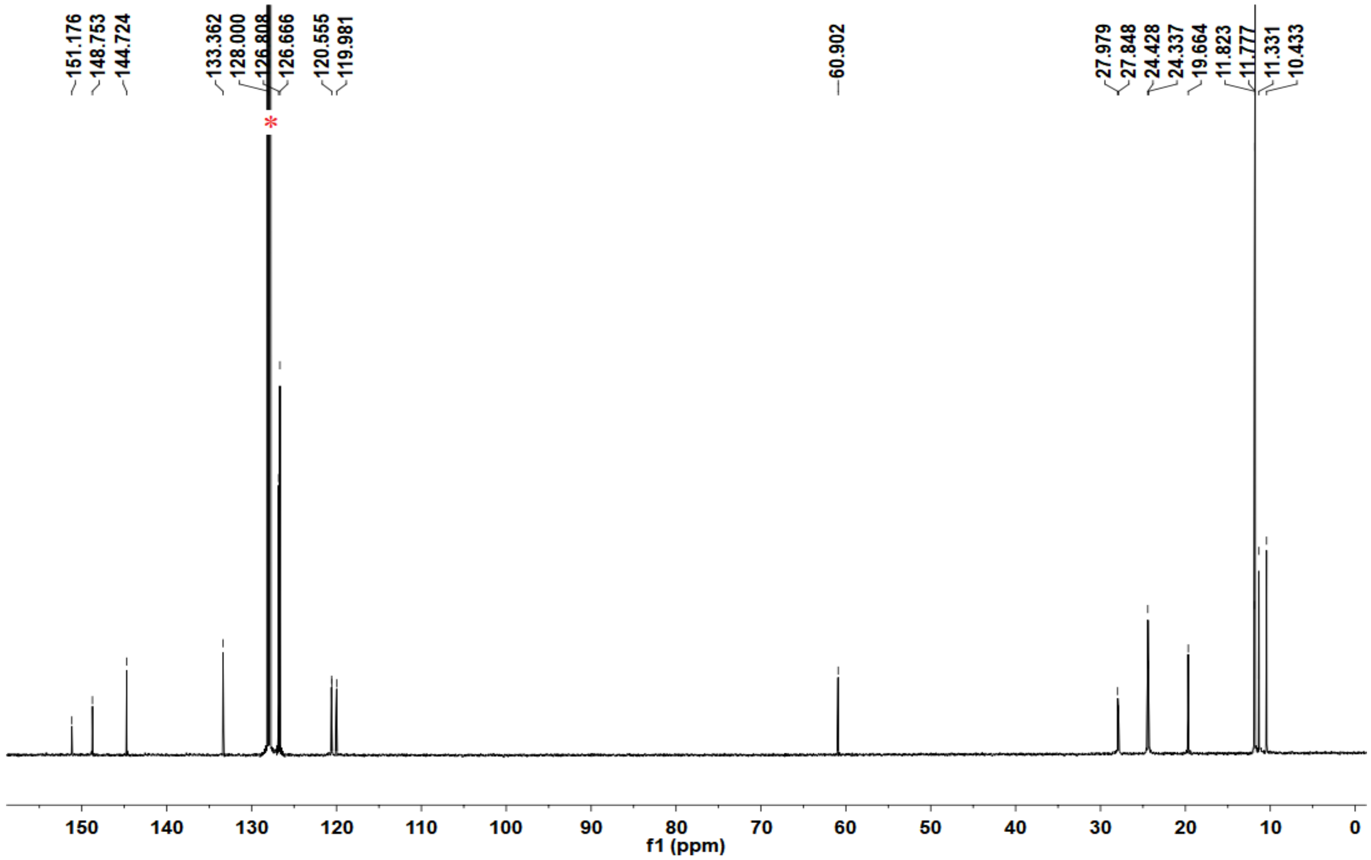

Figure S32. $^{13}C\{^{1}H\}$ NMR (C_6D_6; 20 ℃) spectrum for compound **13** (* solvent).

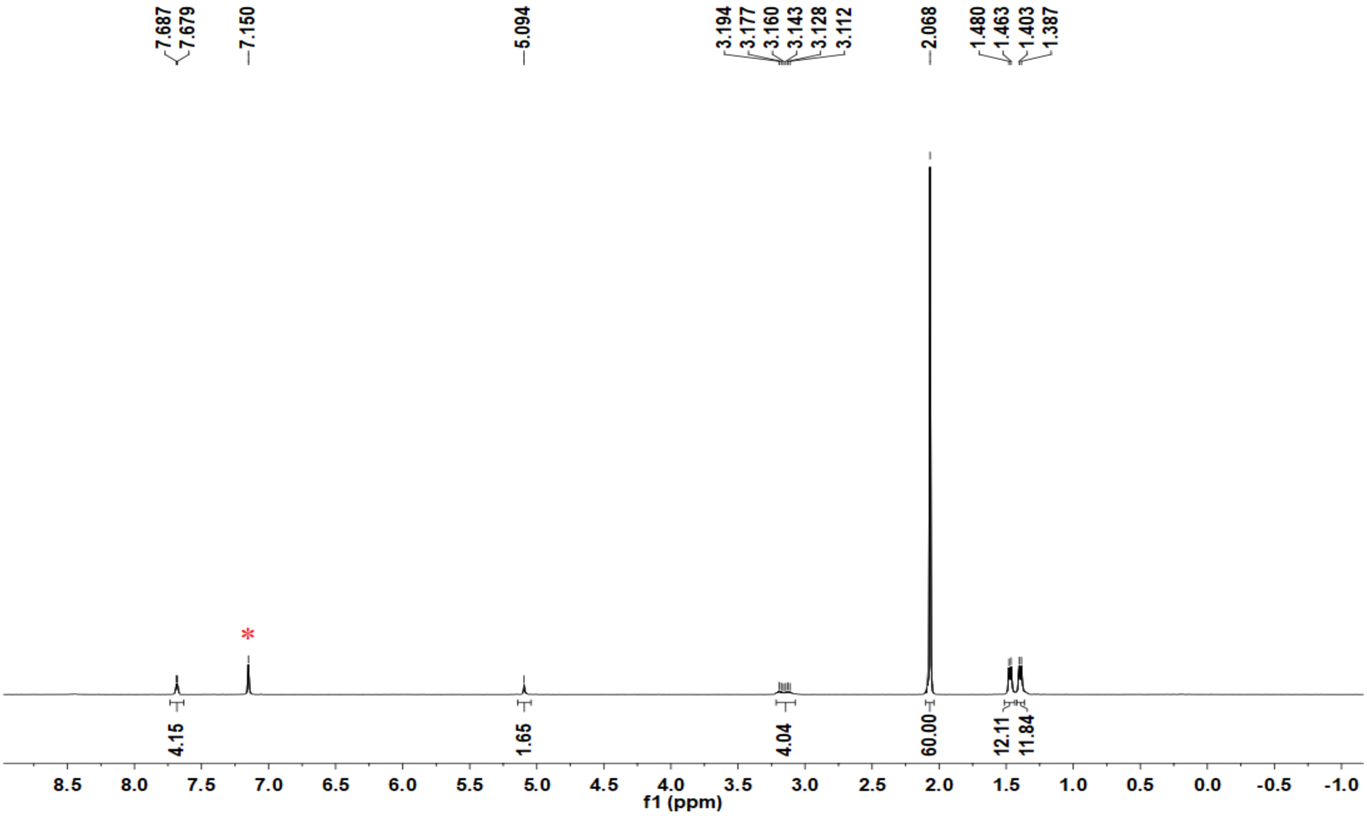

Figure S33. ^{1}H NMR (C_6D_6; 20 ℃) spectrum for compound **14** (* solvent).

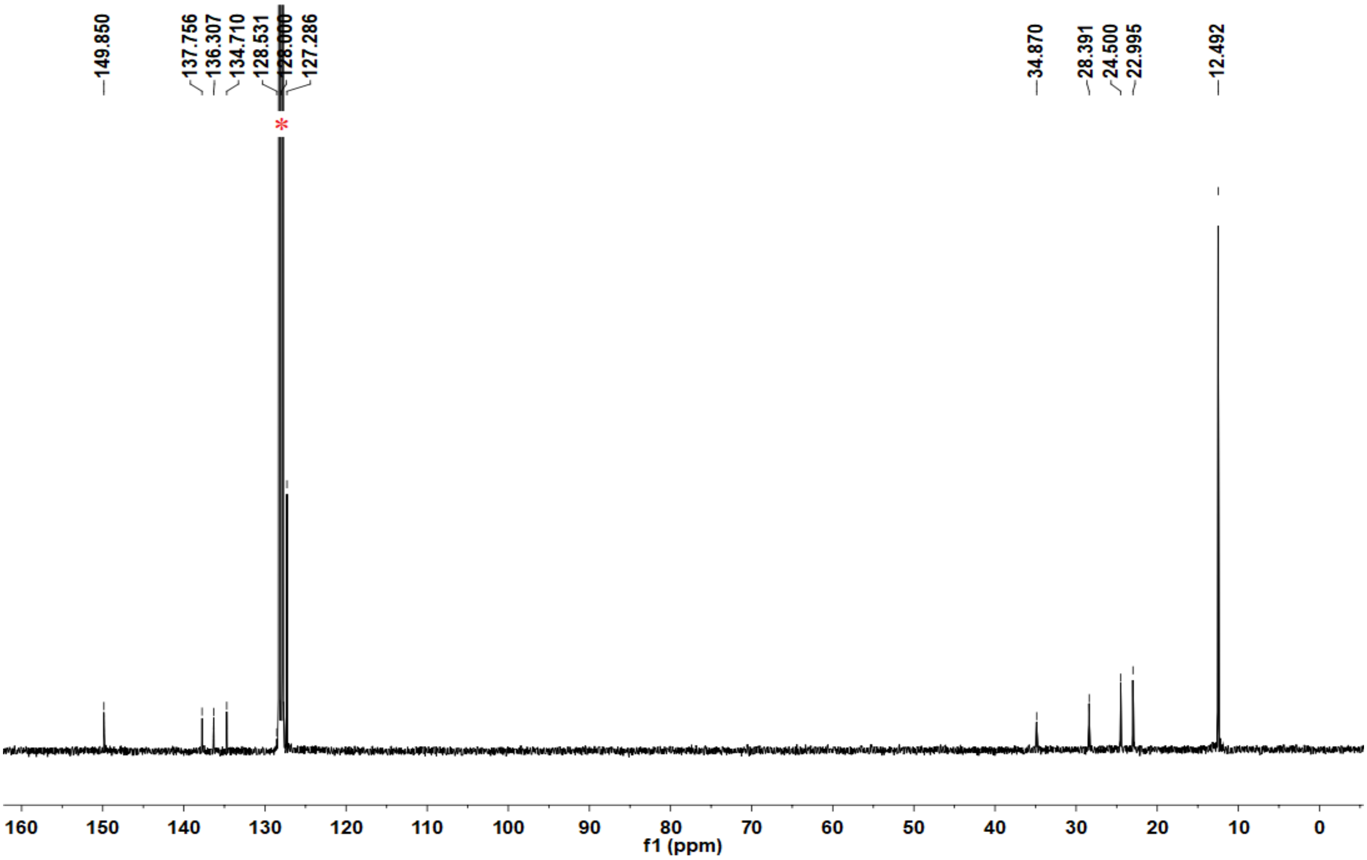

Figure S34. $^{13}C\{^{1}H\}$ NMR (C_6D_6; 20 ℃) spectrum for compound **14** (* solvent).

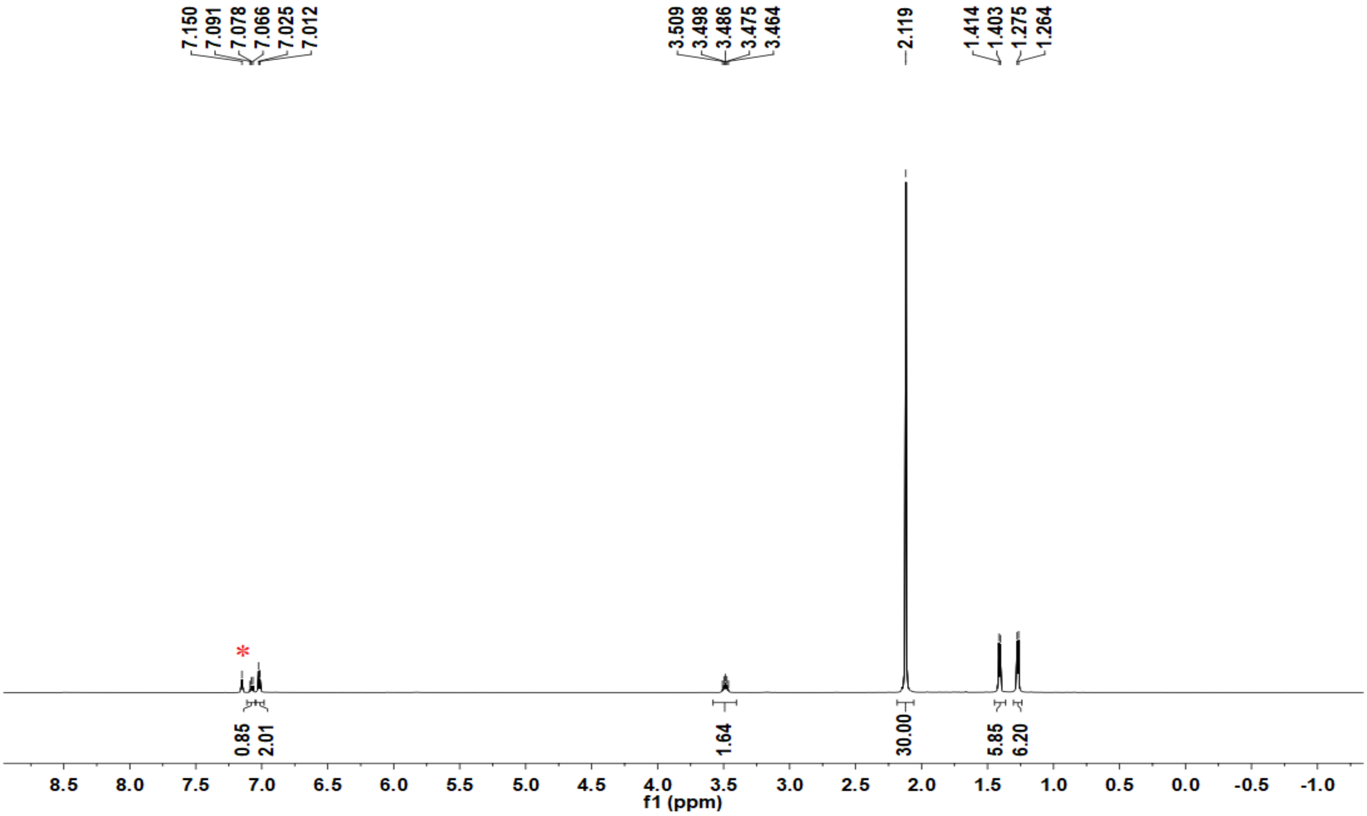

Figure S35. ^{1}H NMR (C_6D_6; 20 ℃) spectrum for compound **15** (* solvent).

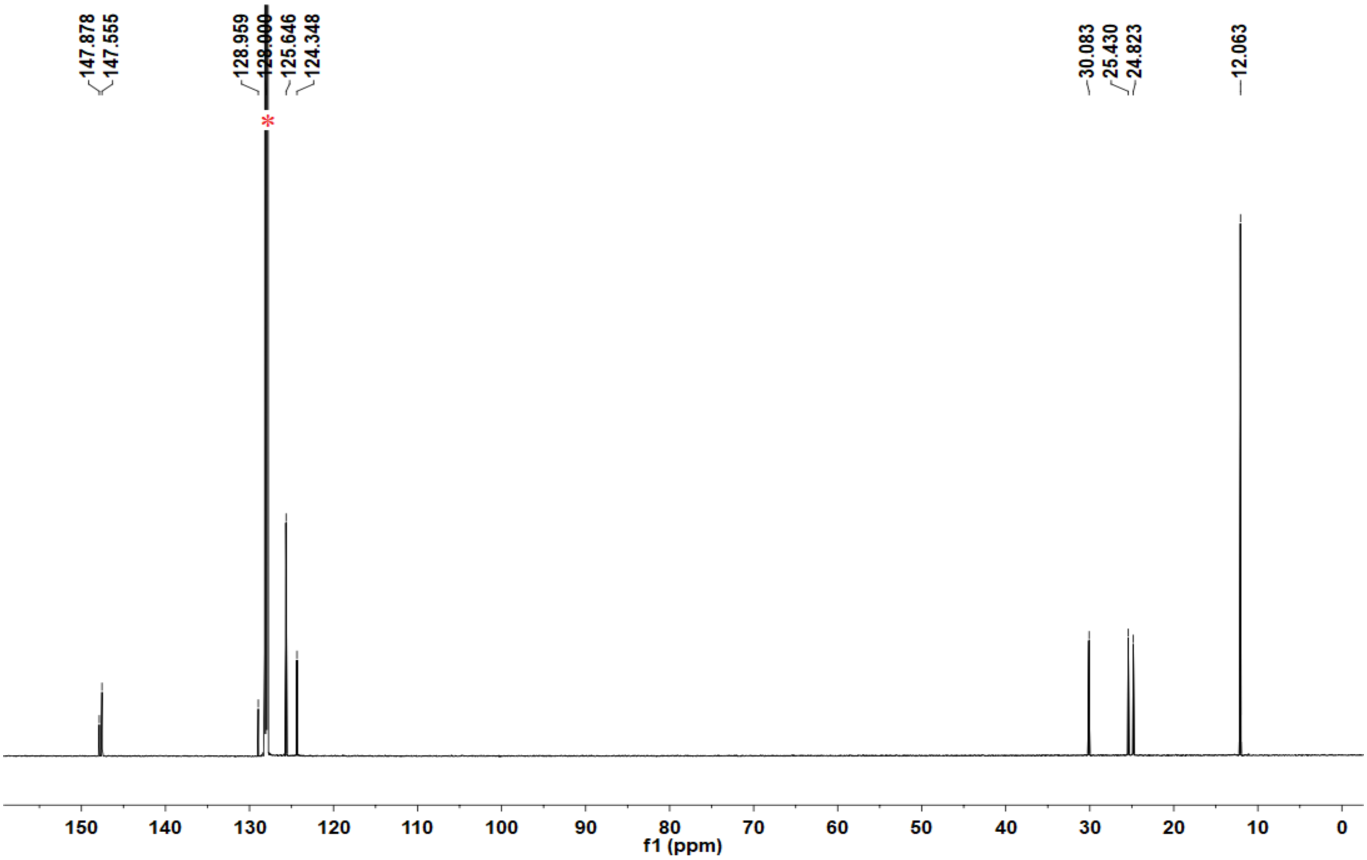

Figure S36. $^{13}C\{^{1}H\}$ NMR (C_6D_6; 20 ℃) spectrum for compound **15** (* solvent).

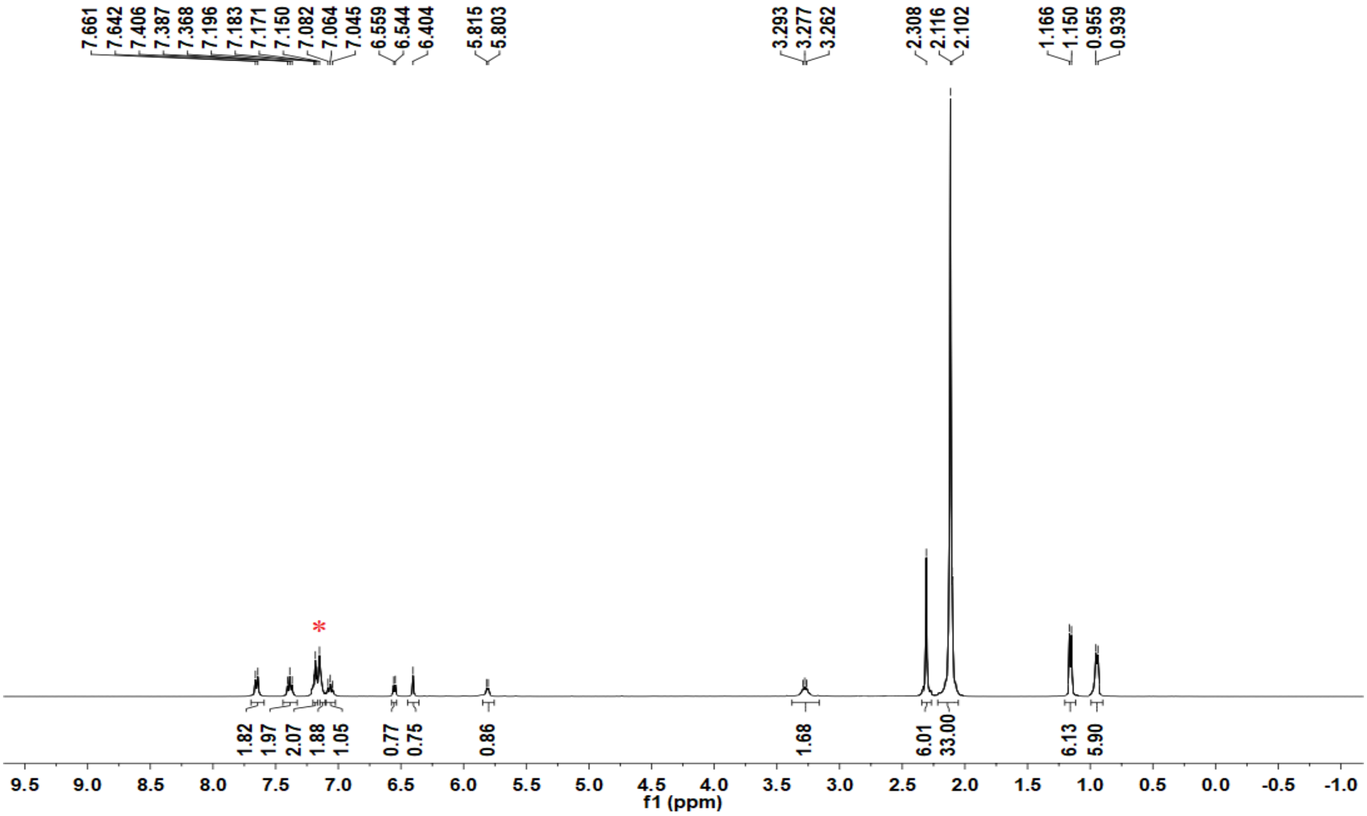

Figure S37. ^{1}H NMR (C_6D_6; 20 ℃) spectrum for compound **16** (* solvent).

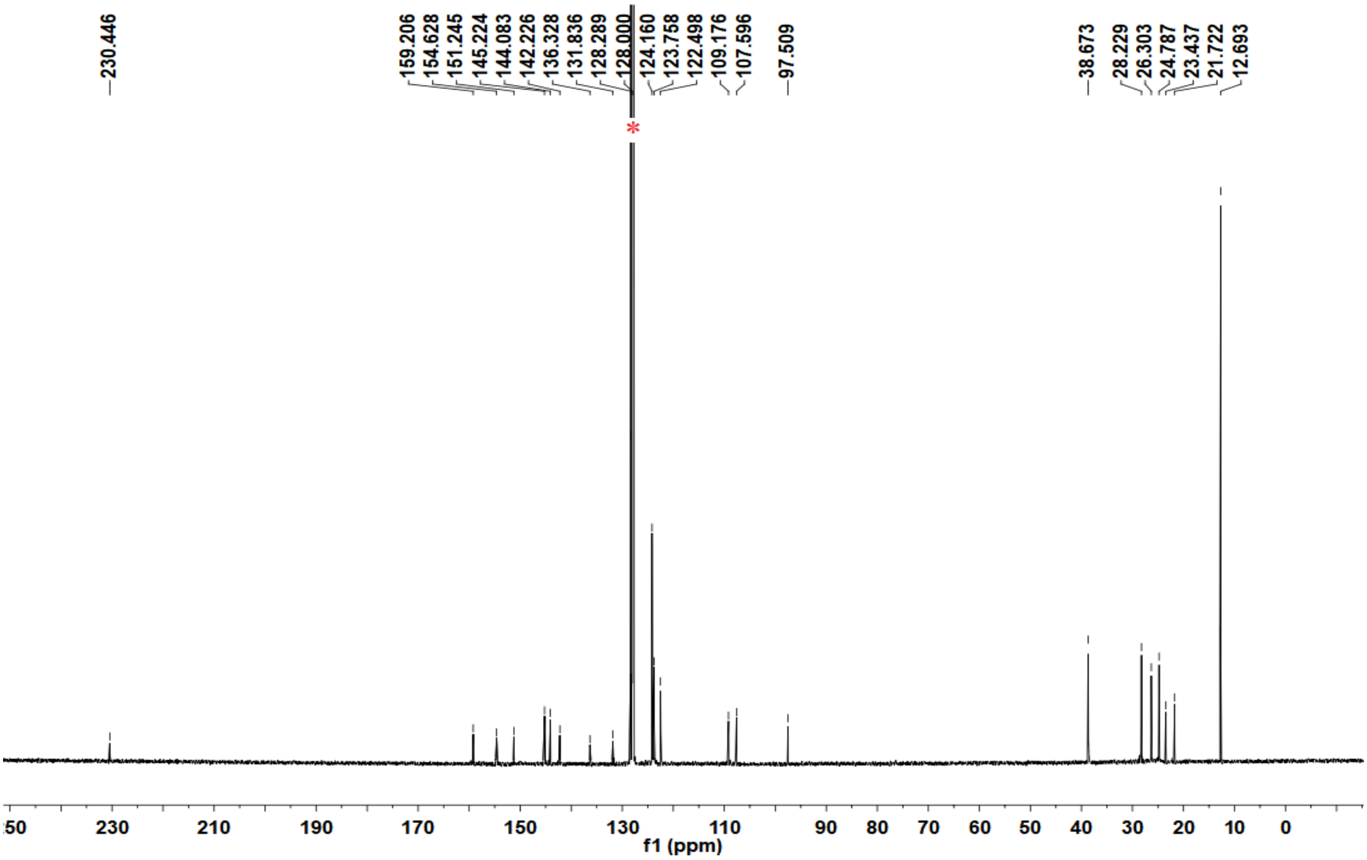

Figure S38. $^{13}C\{^{1}H\}$ NMR (C_6D_6; 20 ℃) spectrum for compound **16** (* solvent).

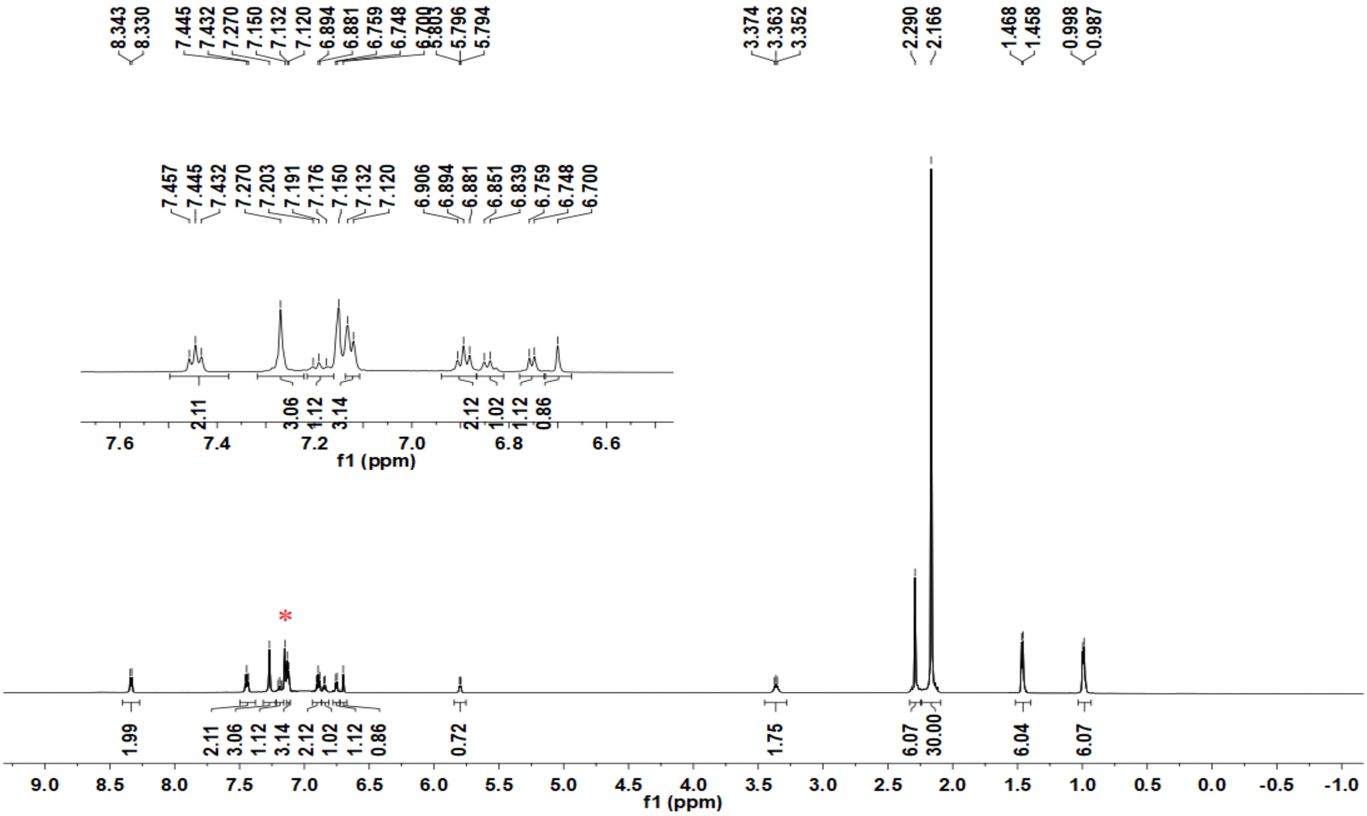

Figure S39. ^{1}H NMR (C_6D_6; 20 ℃) spectrum for compound **17** (* solvent).

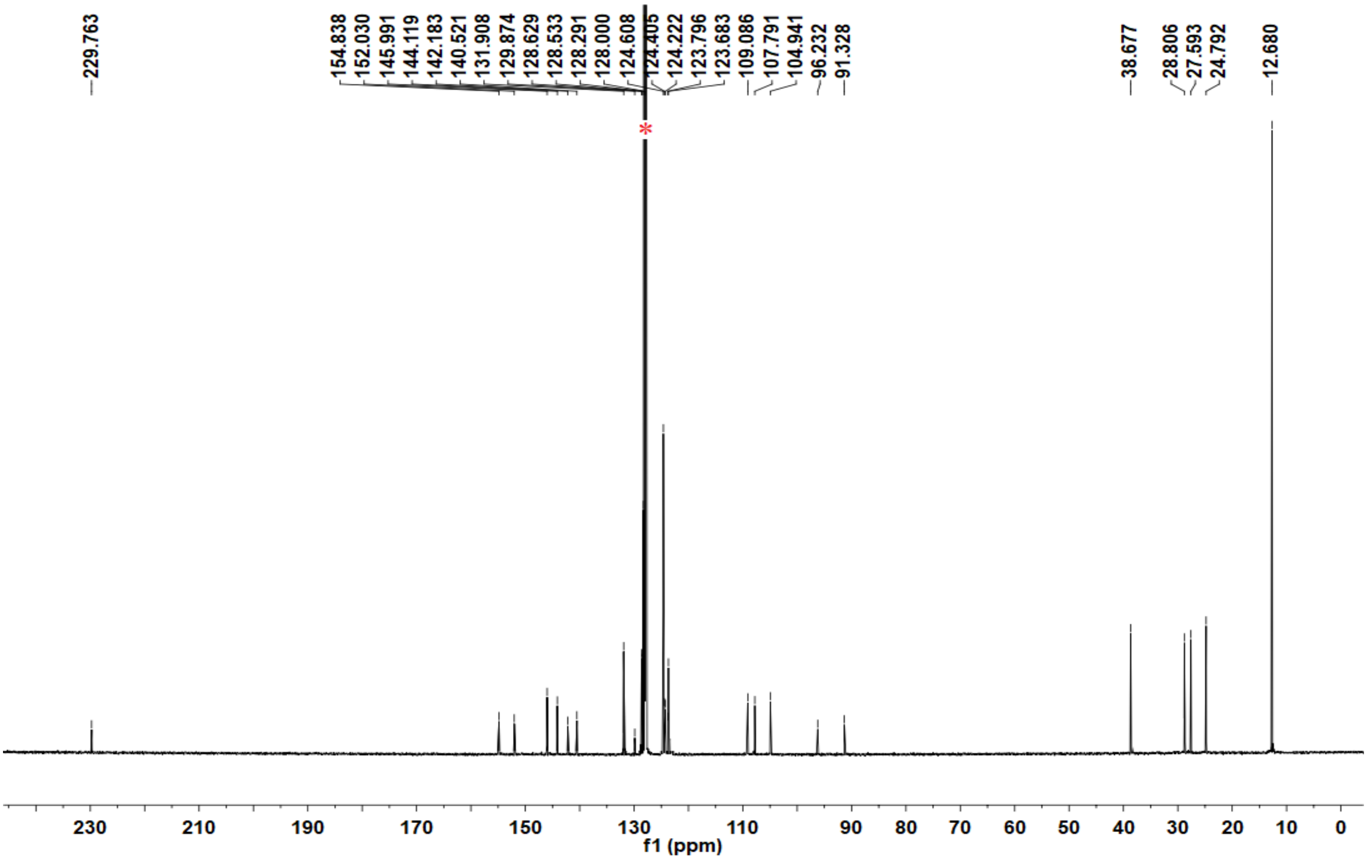

Figure S40. ^{13}C{^{1}H} NMR (C_6D_6; 20 ℃) spectrum for compound **17** (* solvent).

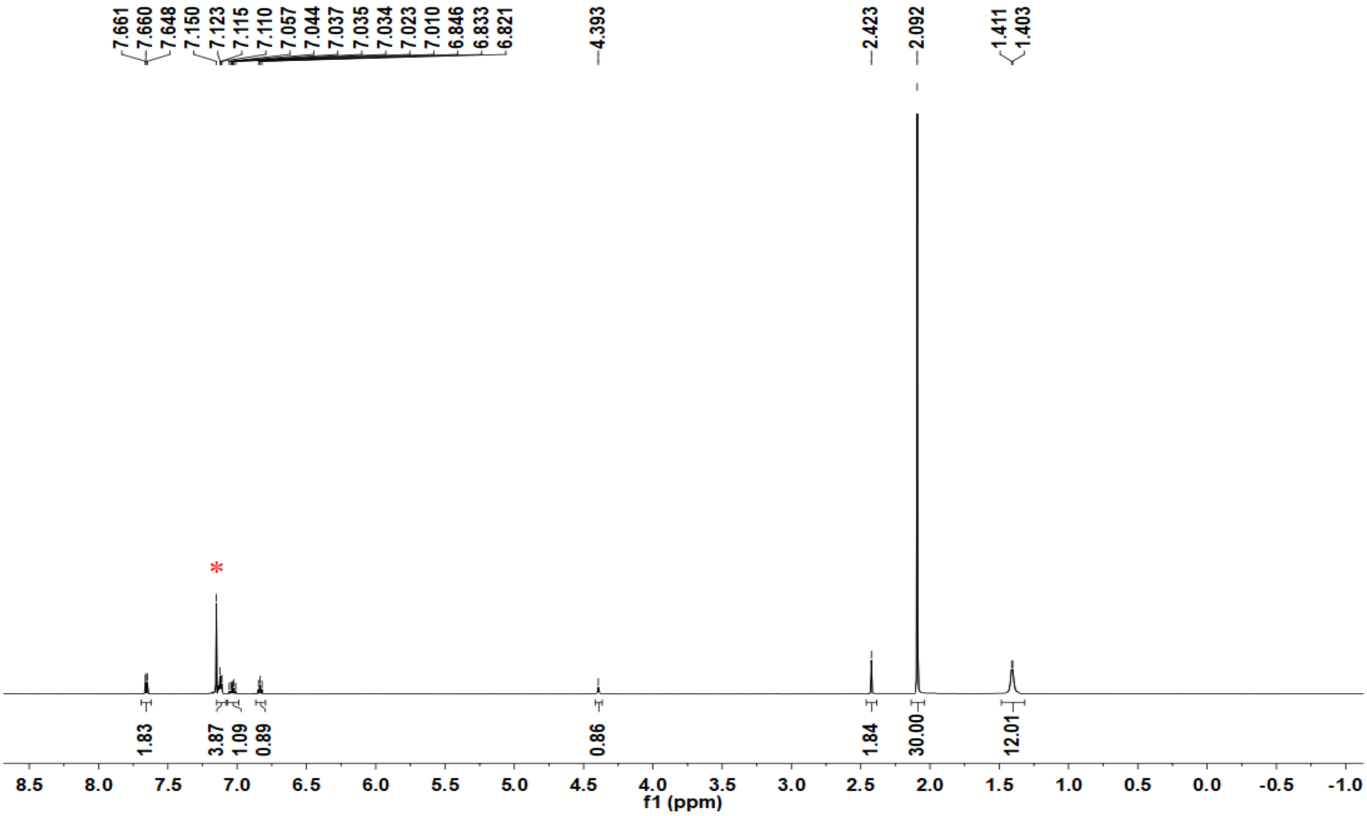

Figure S41. ^{1}H NMR (C_6D_6; 20 ℃) spectrum for compound **18** (* solvent).

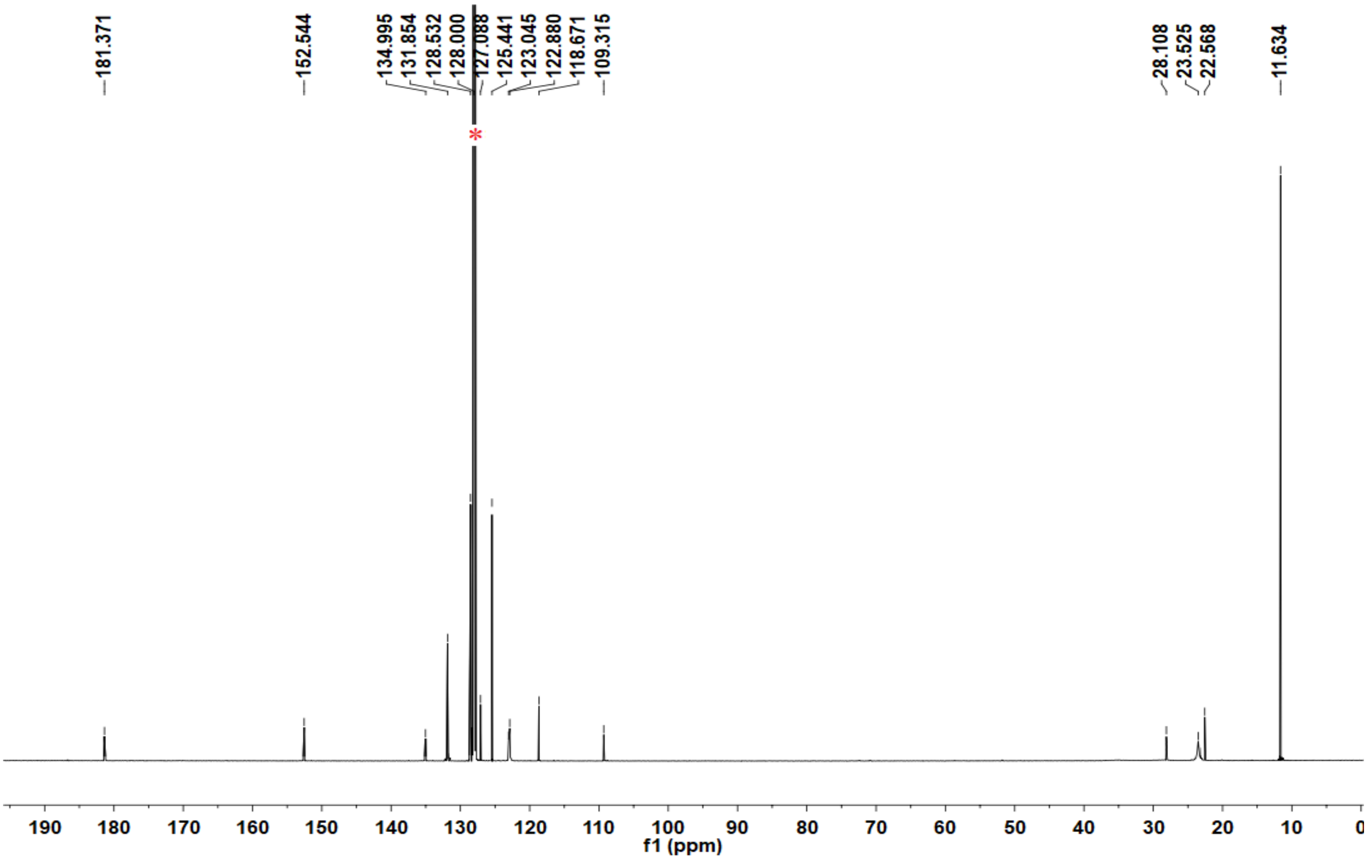

Figure S42. $^{13}C\{^{1}H\}$ NMR (C_6D_6; 20 ℃) spectrum for compound **18** (* solvent).

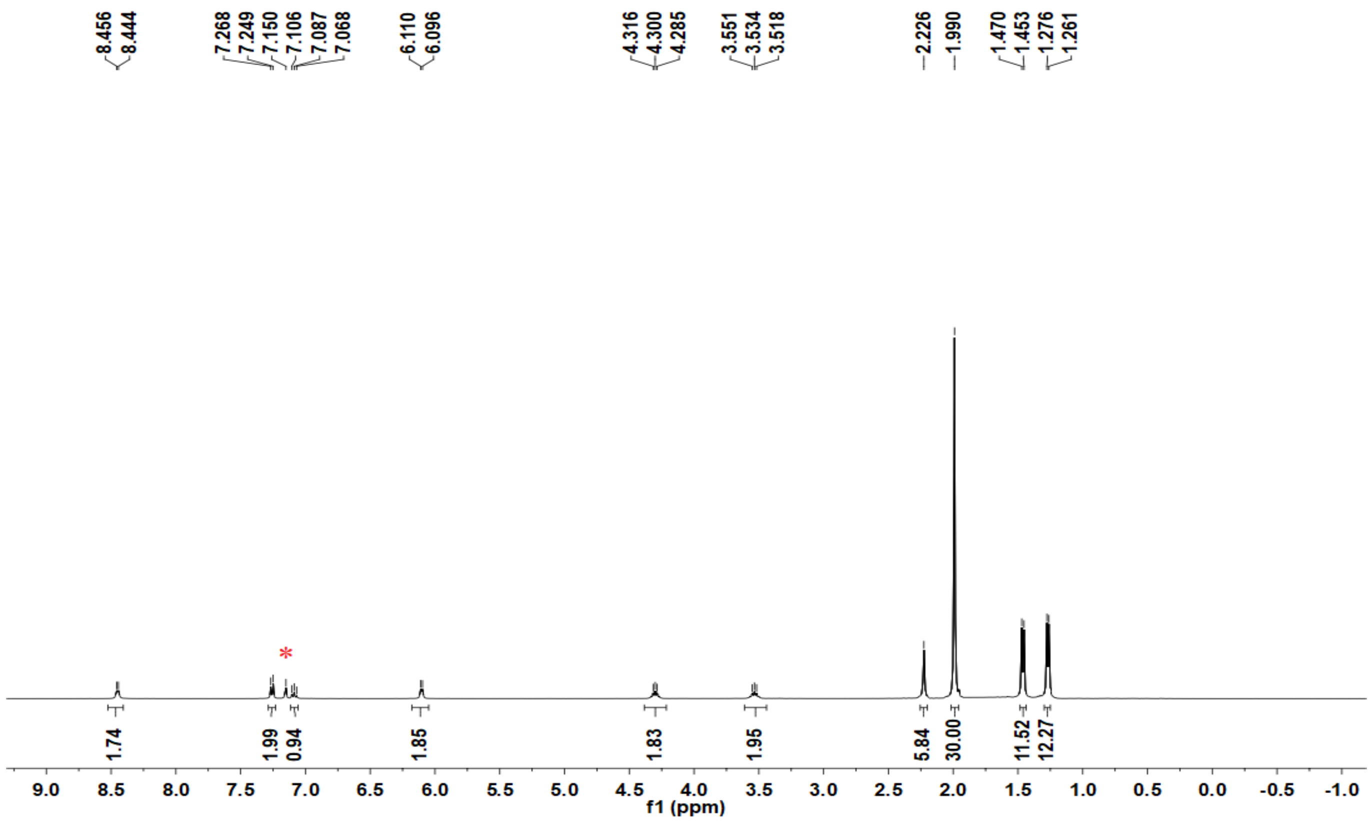

Figure S43. ^{1}H NMR (C_6D_6; 20 ℃) spectrum for compound **19** (* solvent).

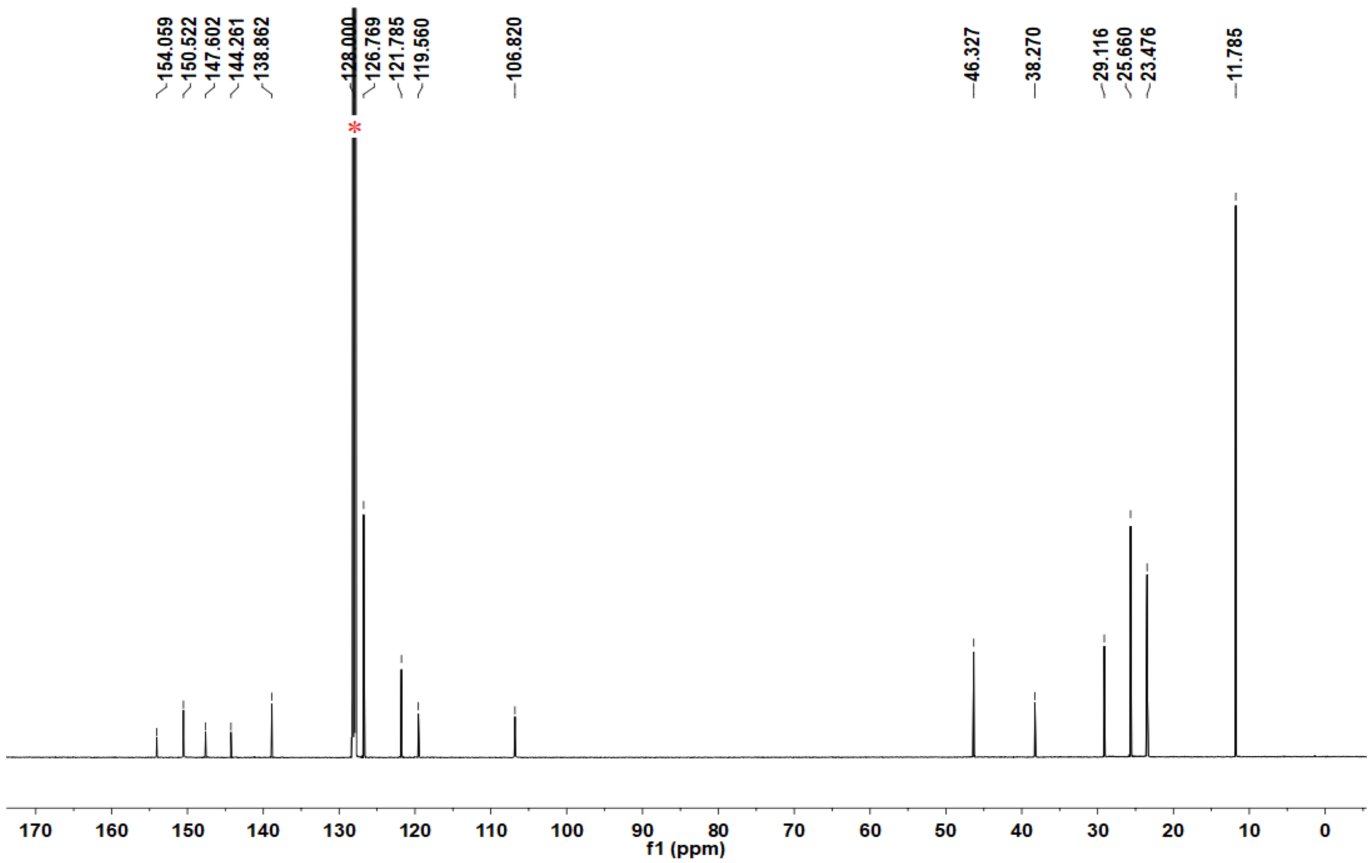

Figure S44. ^{13}C{^{1}H} NMR (C_6D_6; 20 ℃) spectrum for compound **19** (* solvent).

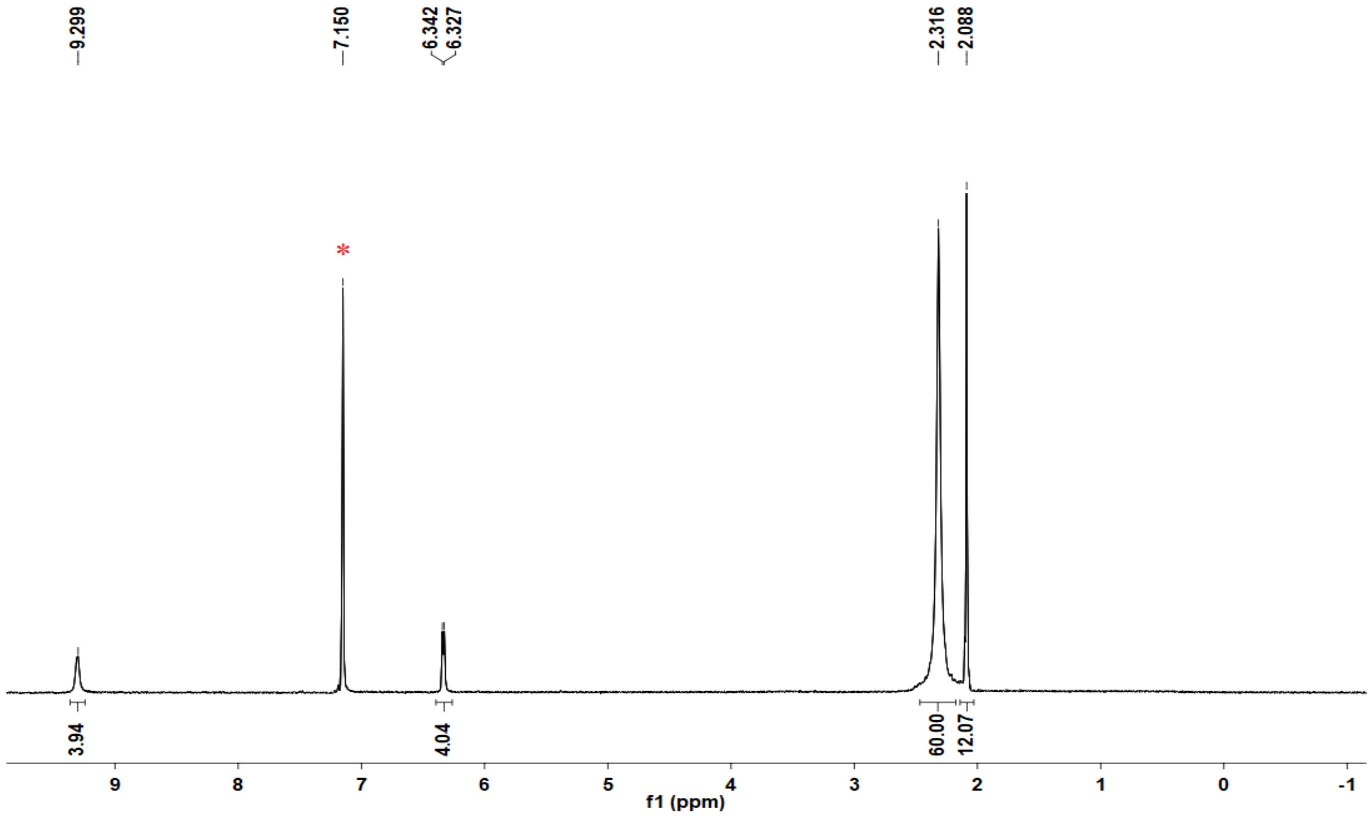

Figure S45. ^{1}H NMR (C_6D_6; 20 ℃) spectrum for compound **20** (* solvent).

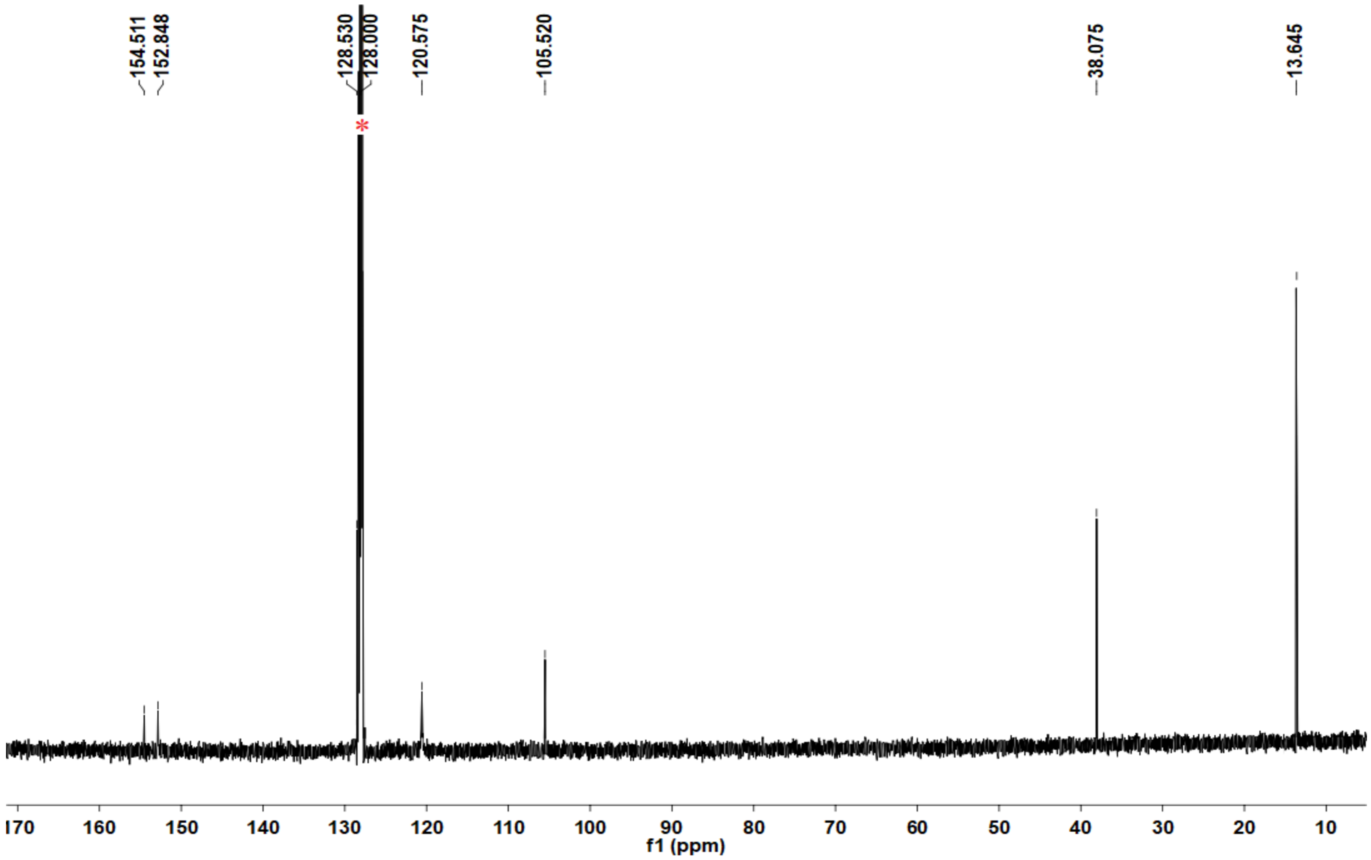

Figure S46. $^{13}C\{^{1}H\}$ NMR (C_6D_6; 20 ℃) spectrum for compound **20** (* solvent).

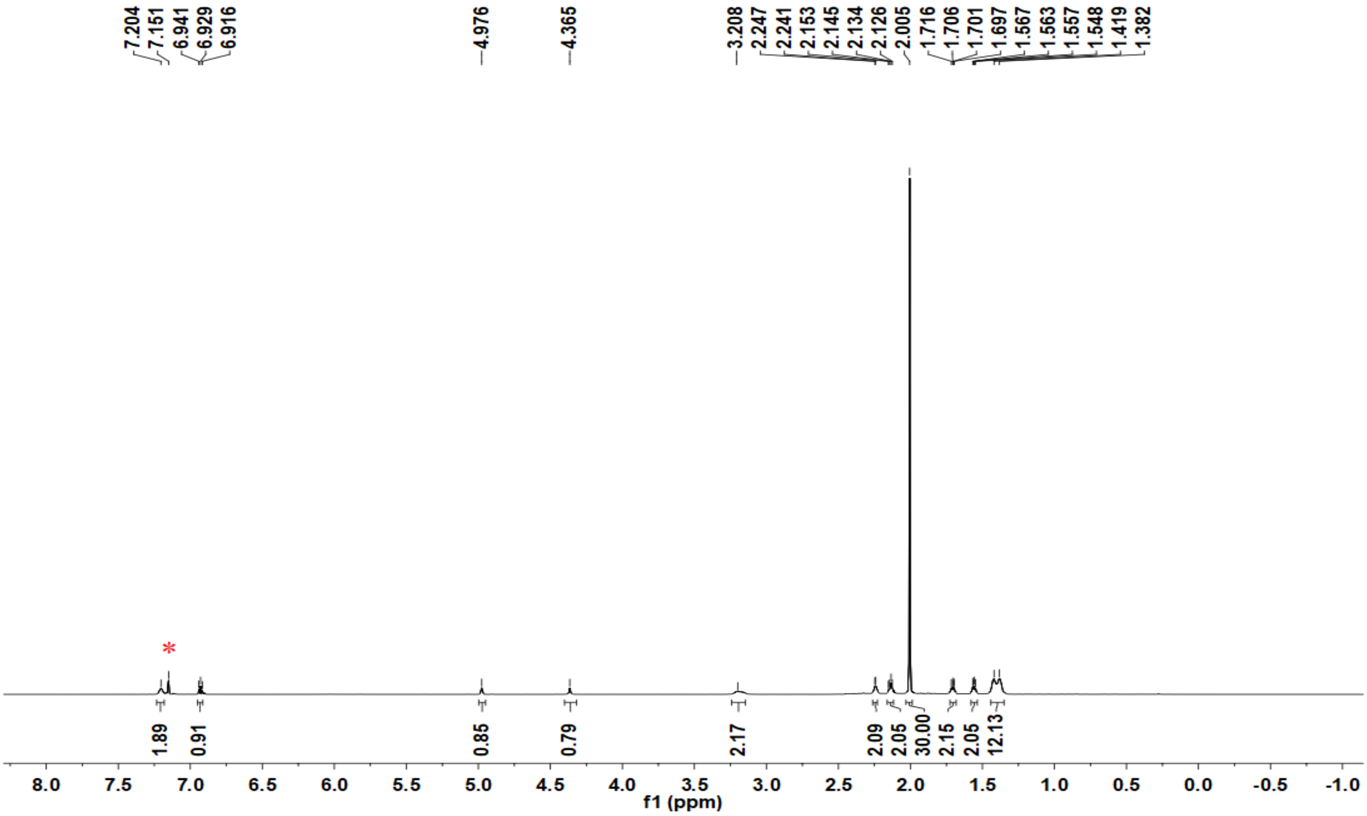

Figure S47. ^{1}H NMR (C_6D_6; 20 ℃) spectrum for compound **21** (* solvent).

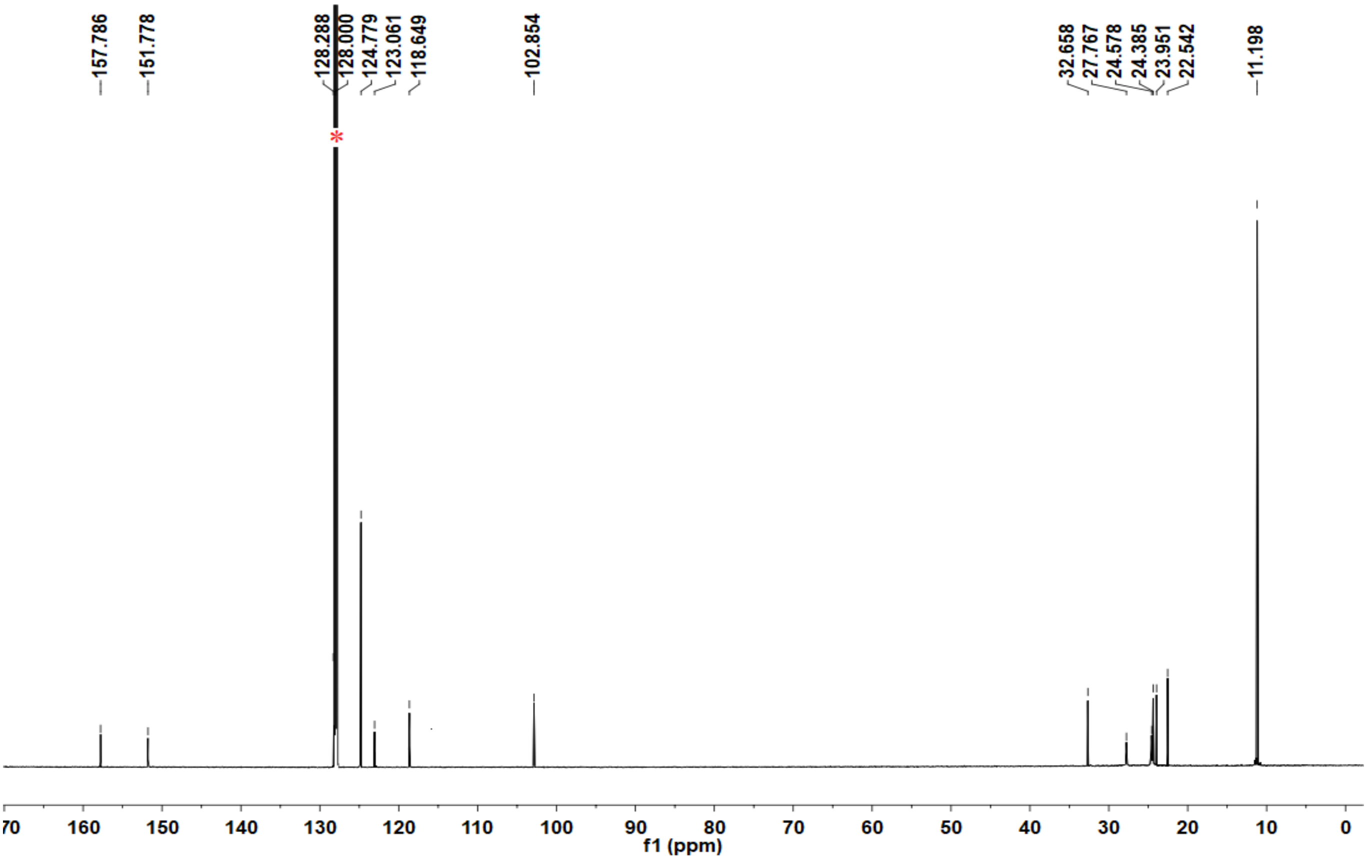

Figure S48. $^{13}C\{^{1}H\}$ NMR (C_6D_6; 20 ℃) spectrum for compound **21** (* solvent).

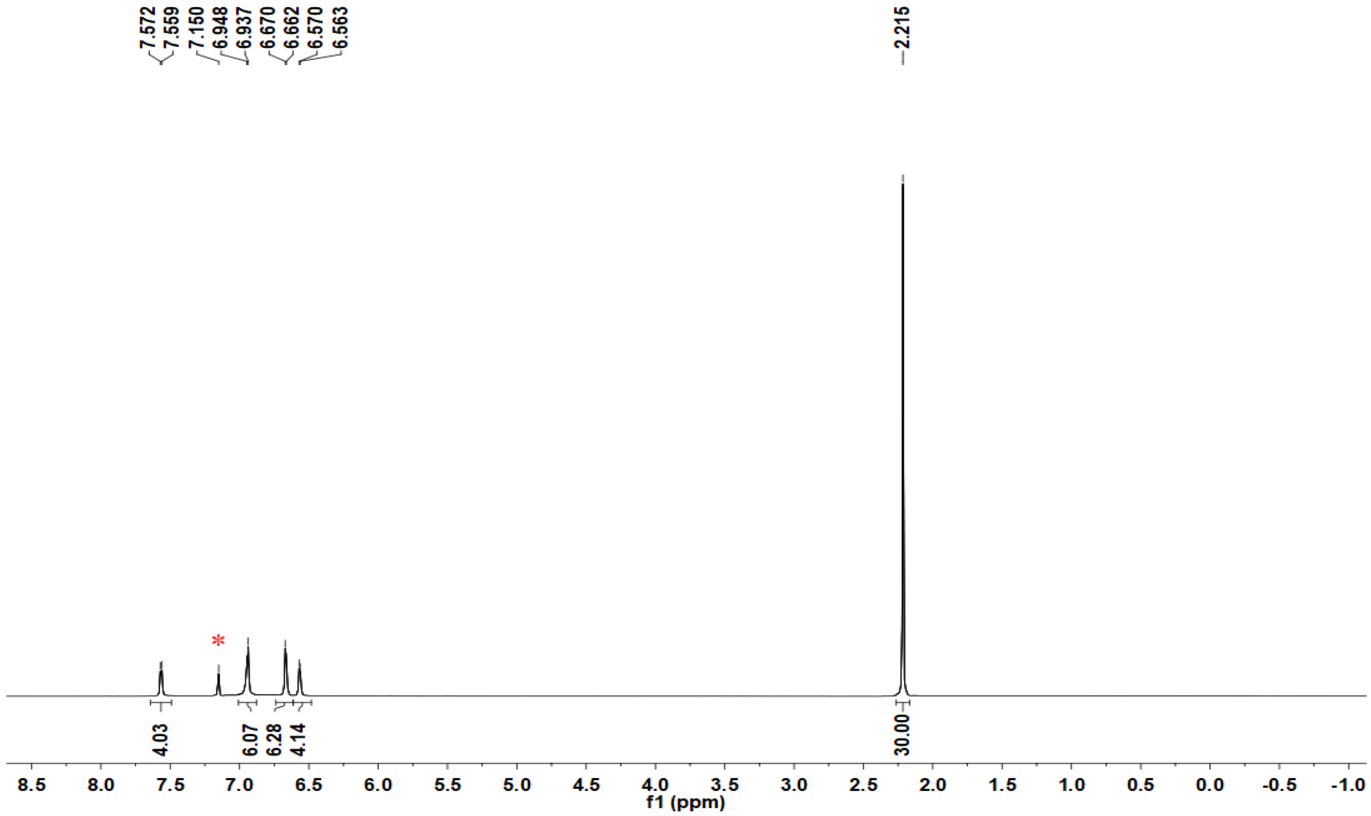

Figure S49. ^{1}H NMR (C_6D_6; 20 ℃) spectrum for compound **23** (* solvent).

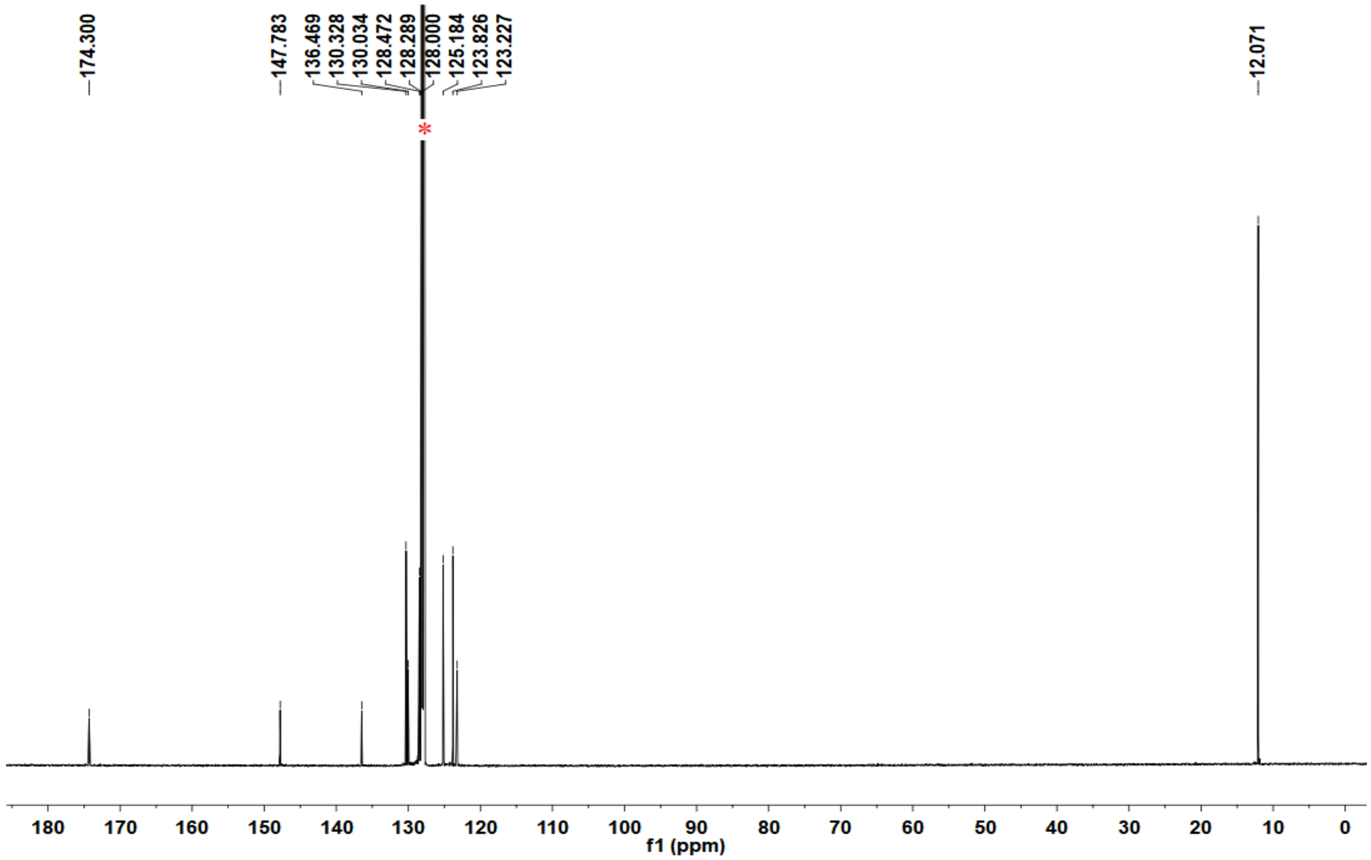

Figure S50. $^{13}C\{^{1}H\}$ NMR (C_6D_6; 20 ℃) spectrum for compound **23** (* solvent).

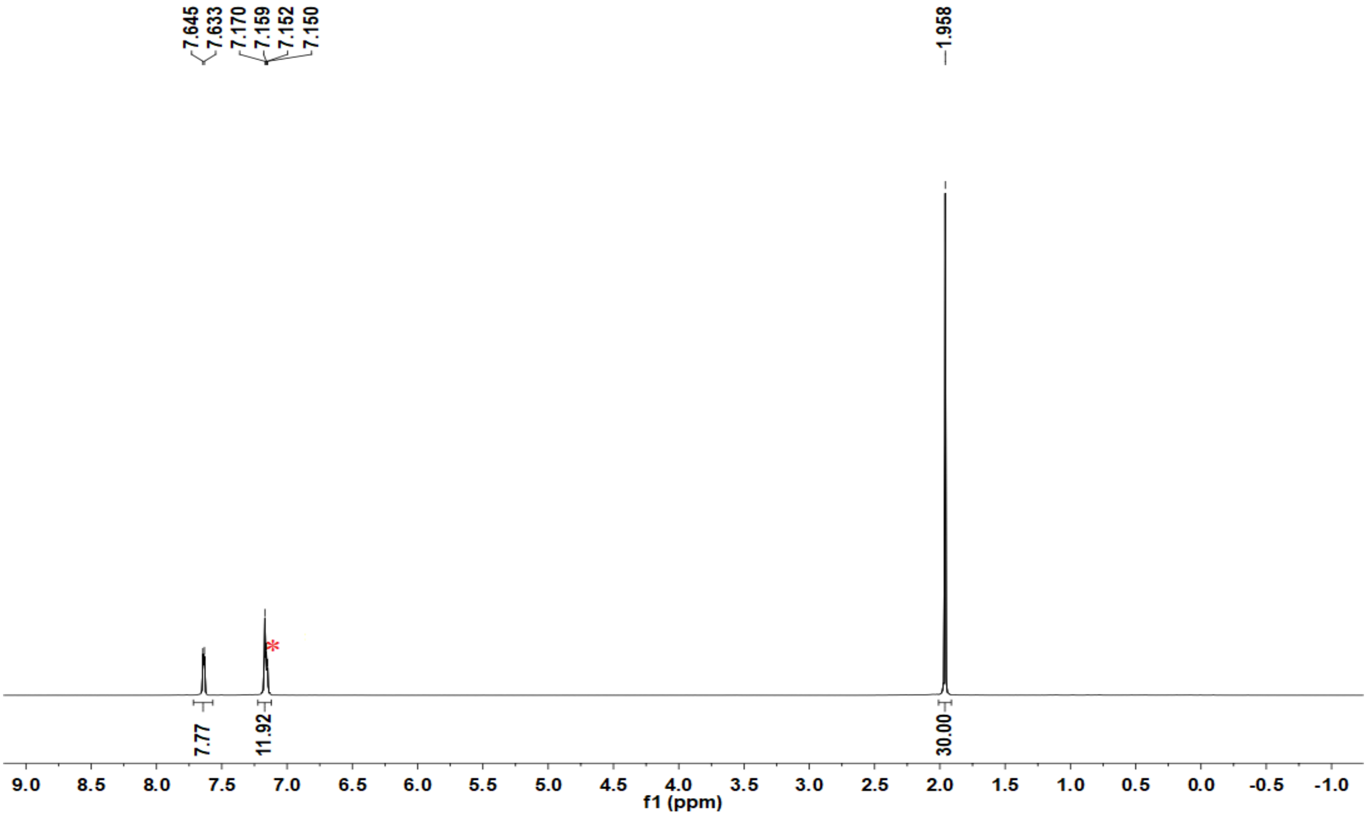

Figure S51. ^{1}H NMR (C_6D_6; 20 ℃) spectrum for compound **24** (* solvent).

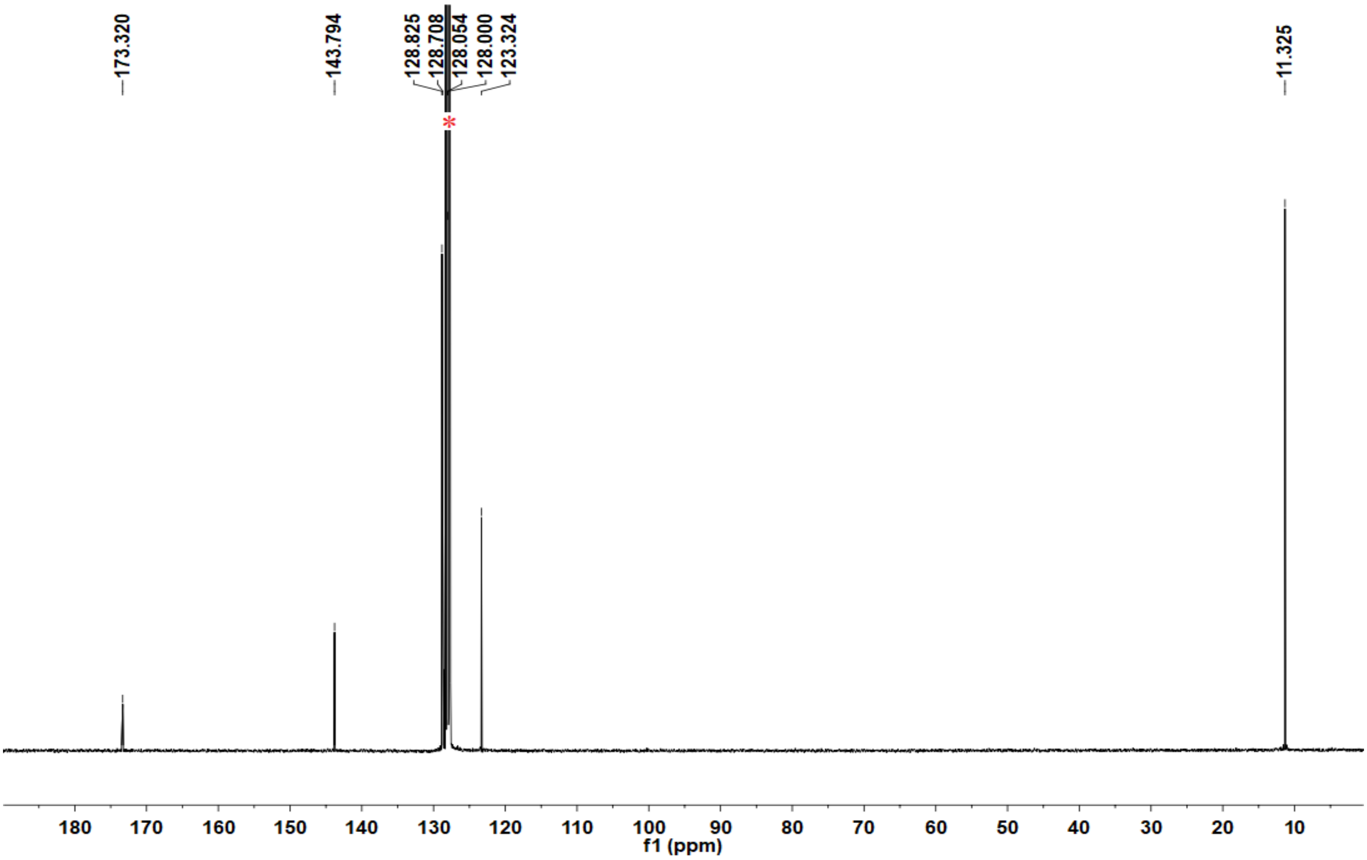

Figure S52. $^{13}C\{^{1}H\}$ NMR (C_6D_6; 20 ℃) spectrum for compound **24** (* solvent).

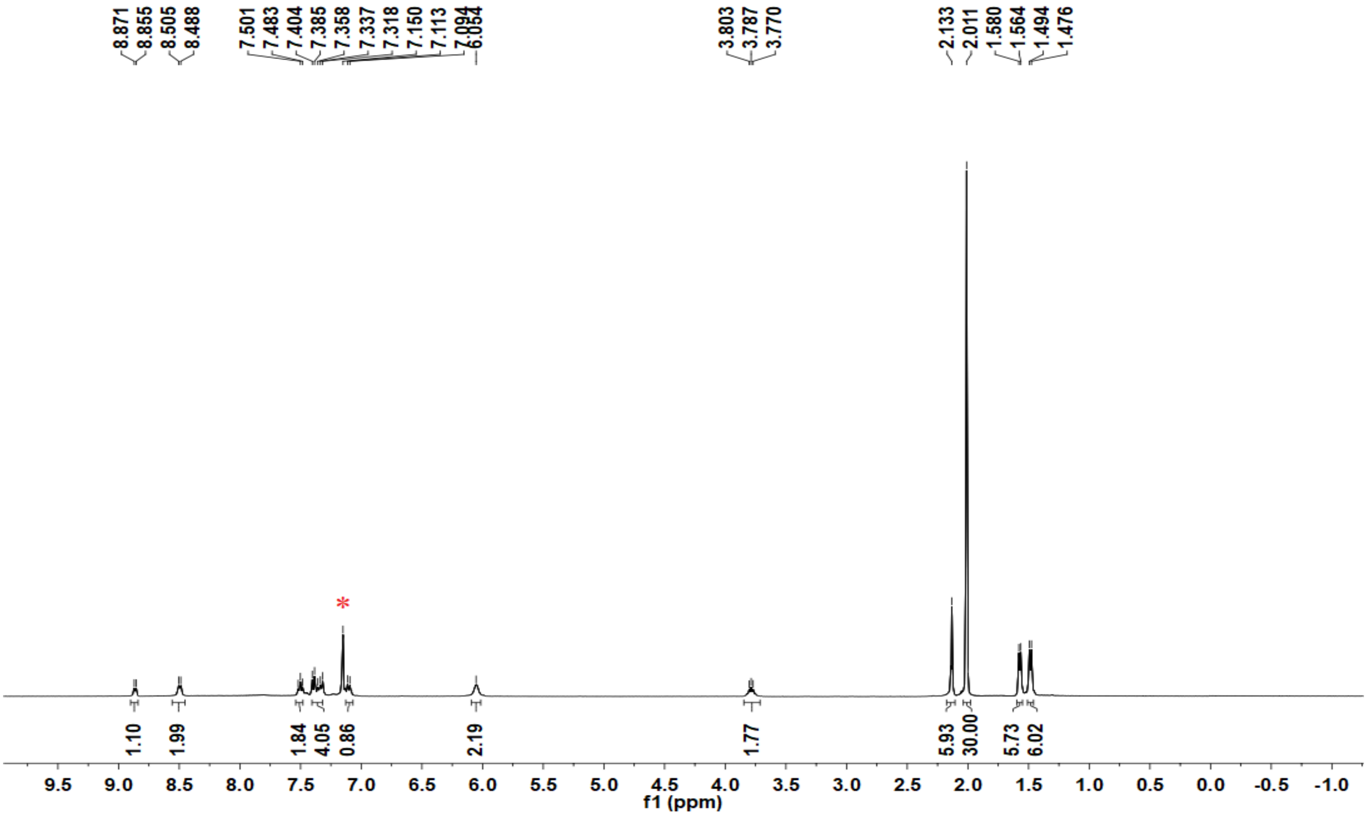

Figure S53. ^{1}H NMR (C_6D_6; 20 ℃) spectrum for compound **25** (* solvent).

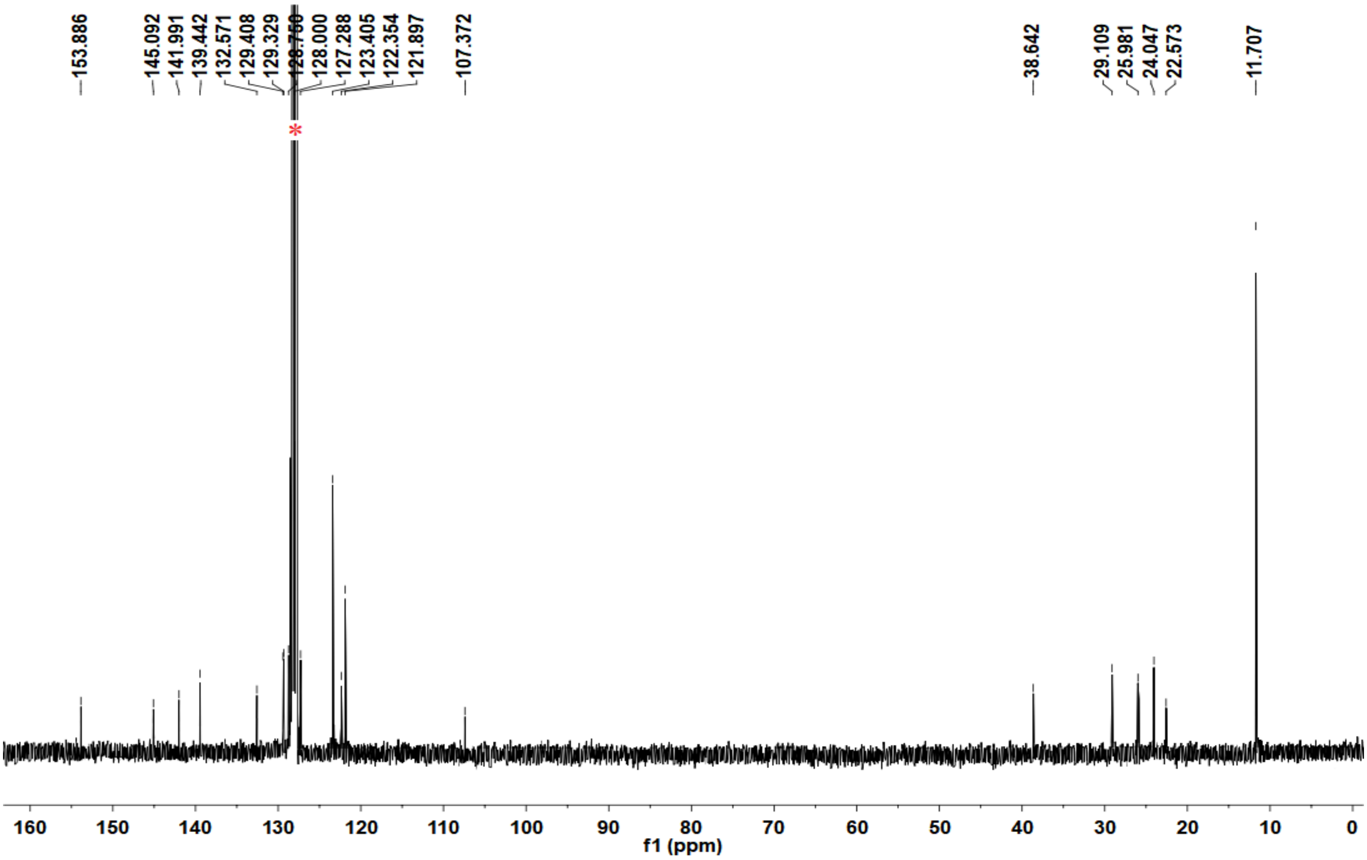

Figure S54. $^{13}C\{^{1}H\}$ NMR (C_6D_6; 20 ℃) spectrum for compound **25** (* solvent).

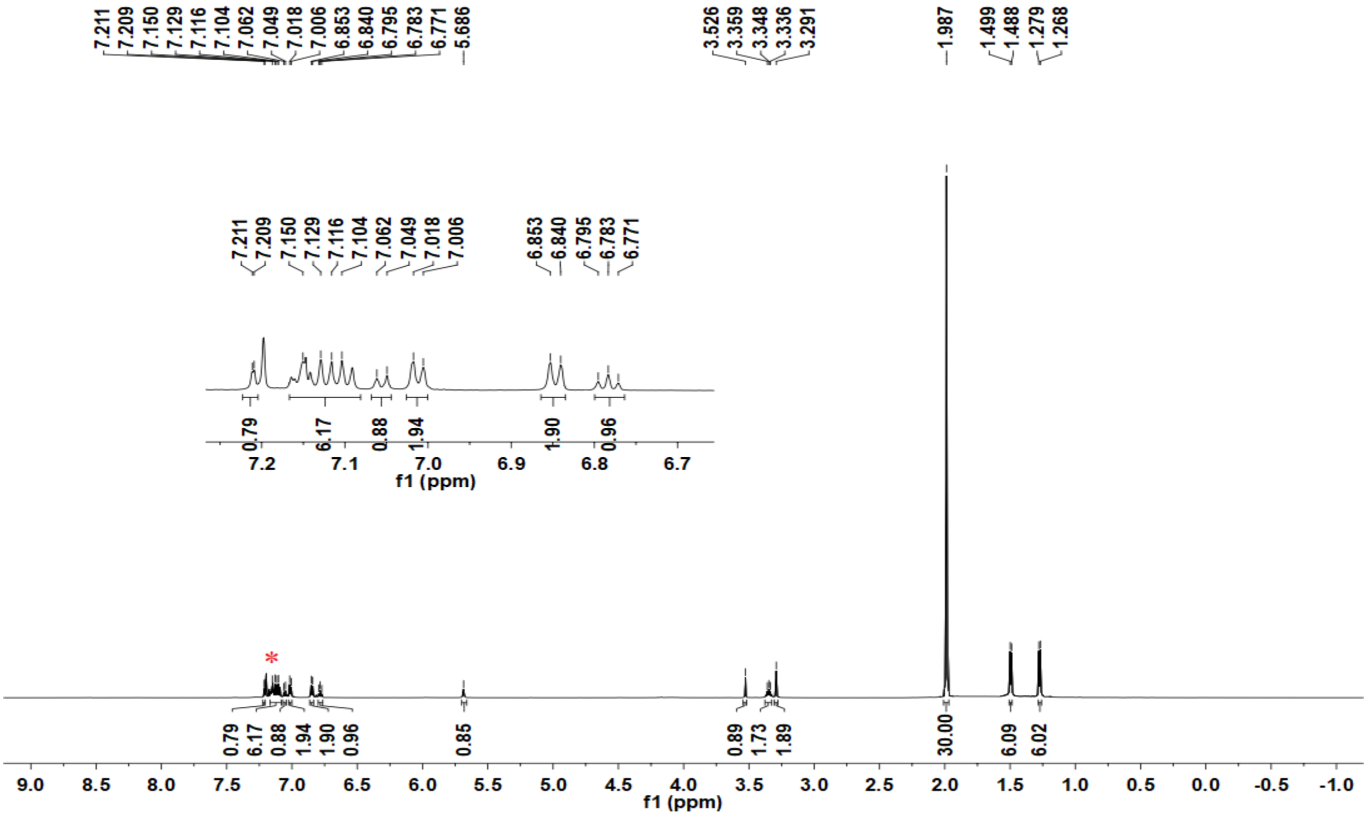

Figure S55. ^{1}H NMR (C_6D_6; 20 ℃) spectrum for compound **26** (* solvent).

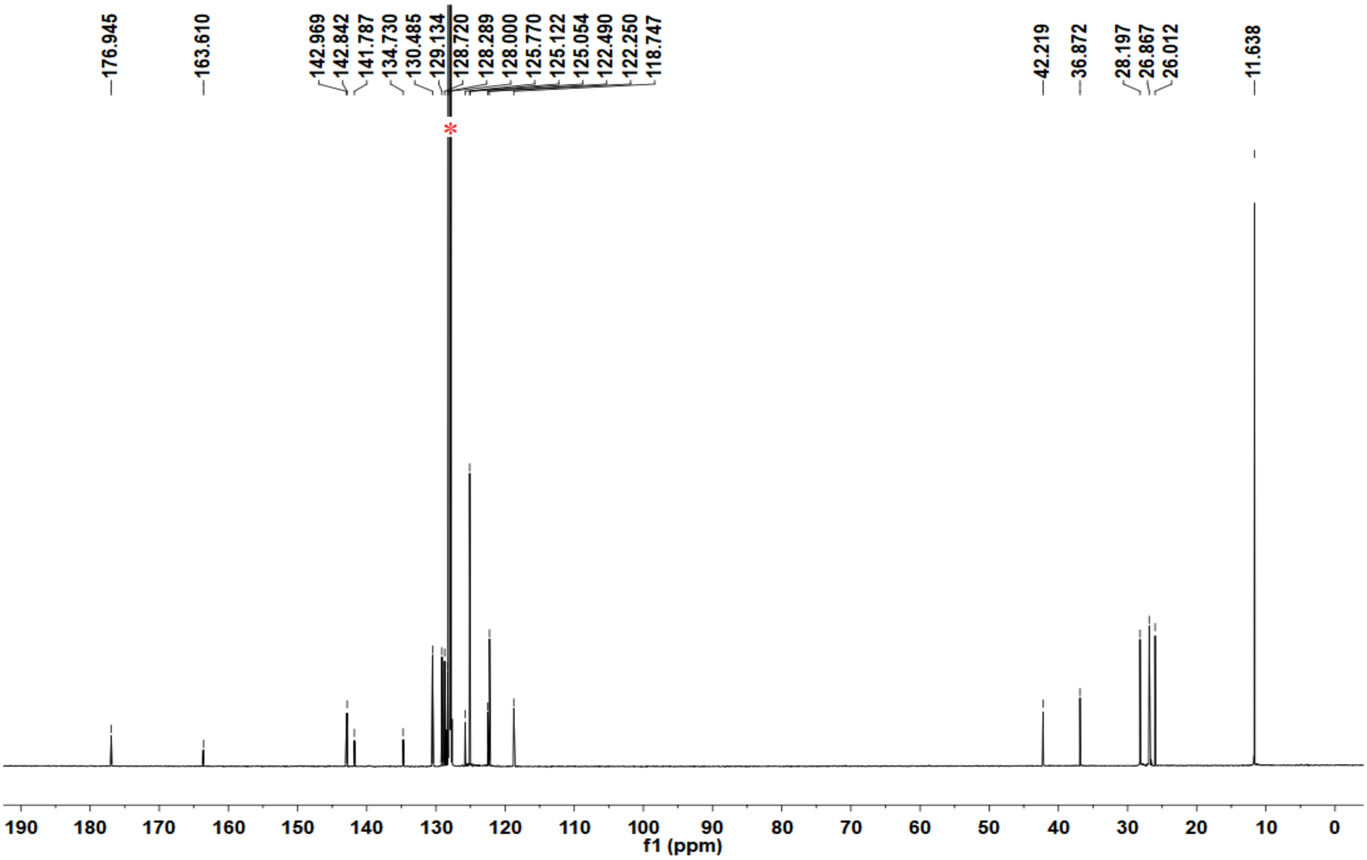

Figure S56. $^{13}C\{^{1}H\}$ NMR (C_6D_6; 20 ℃) spectrum for compound **26** (* solvent).

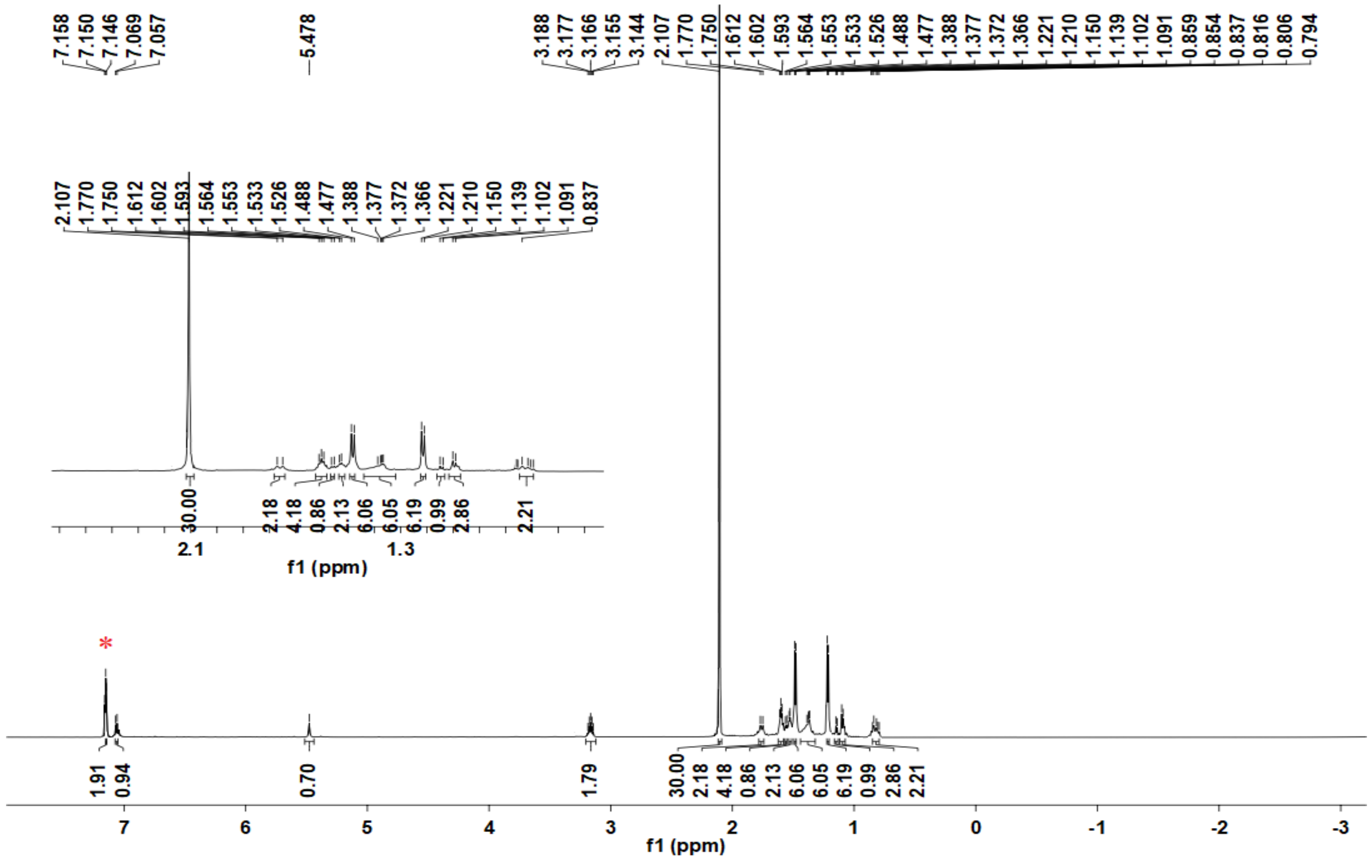

Figure S57. ^{1}H NMR (C_6D_6; 20 ℃) spectrum for compound **27** (* solvent).

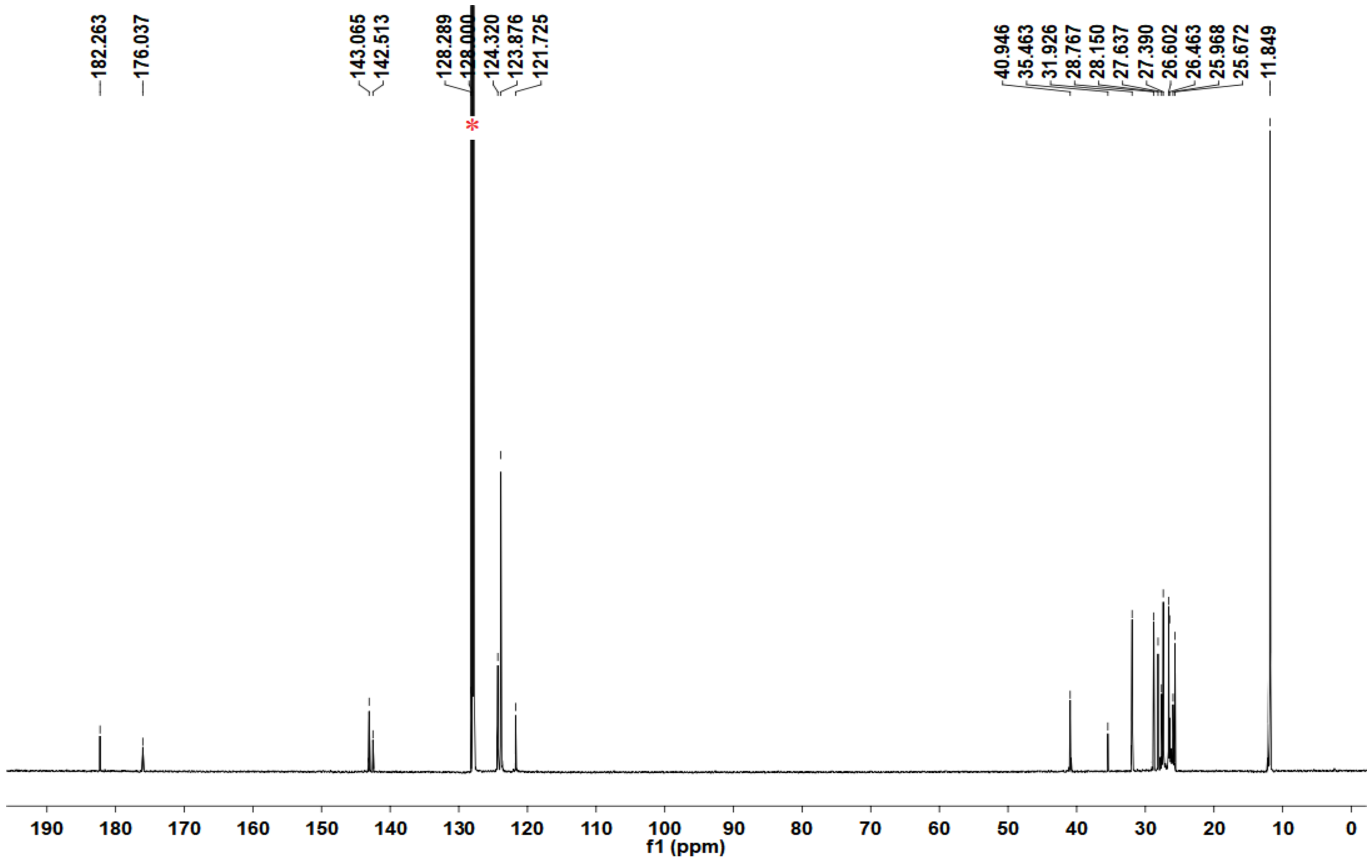

Figure S58. $^{13}C\{^{1}H\}$ NMR (C_6D_6; 20 ℃) spectrum for compound **27** (* solvent).

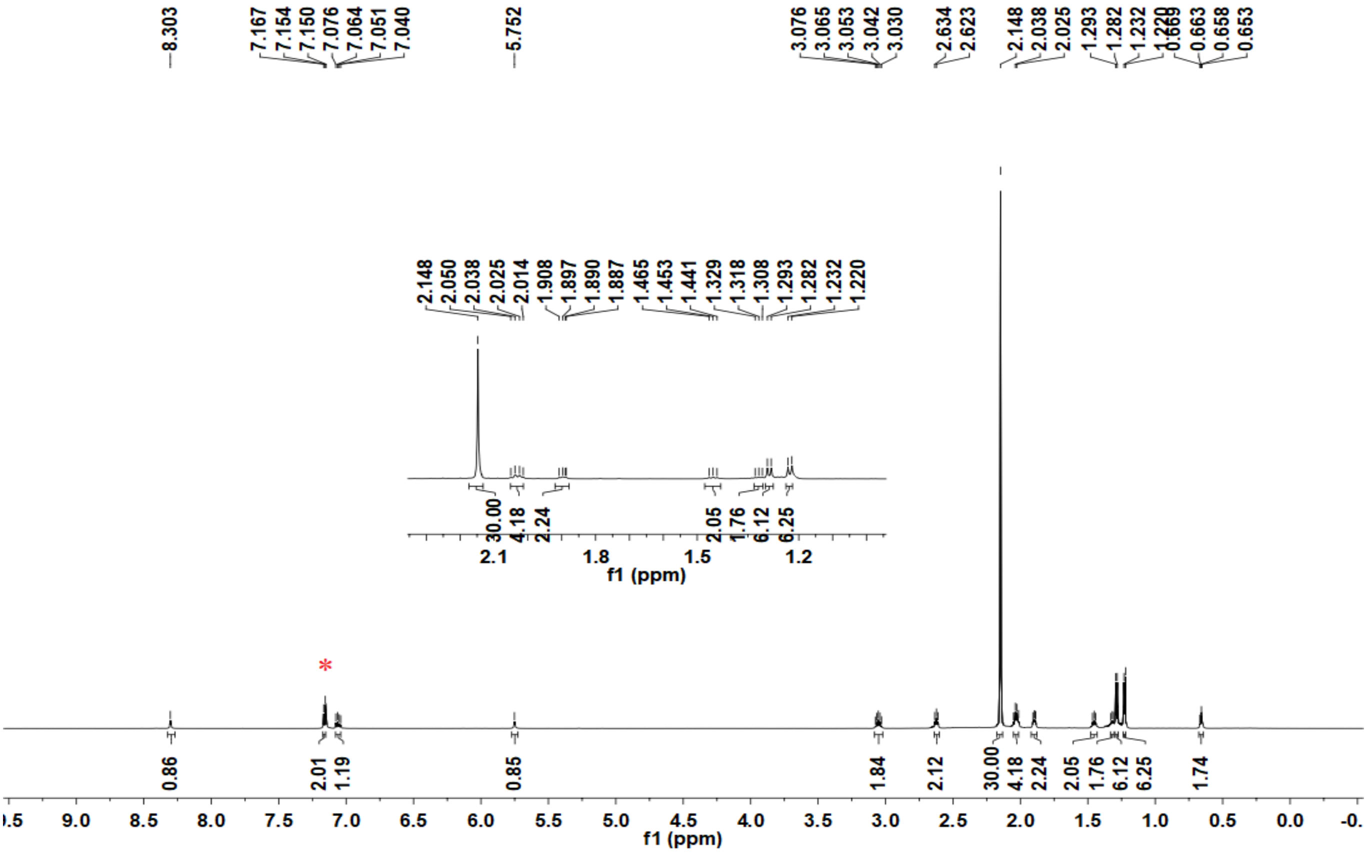

Figure S59. ^{1}H NMR (C_6D_6; 20 ℃) spectrum for compound **28** (* solvent).

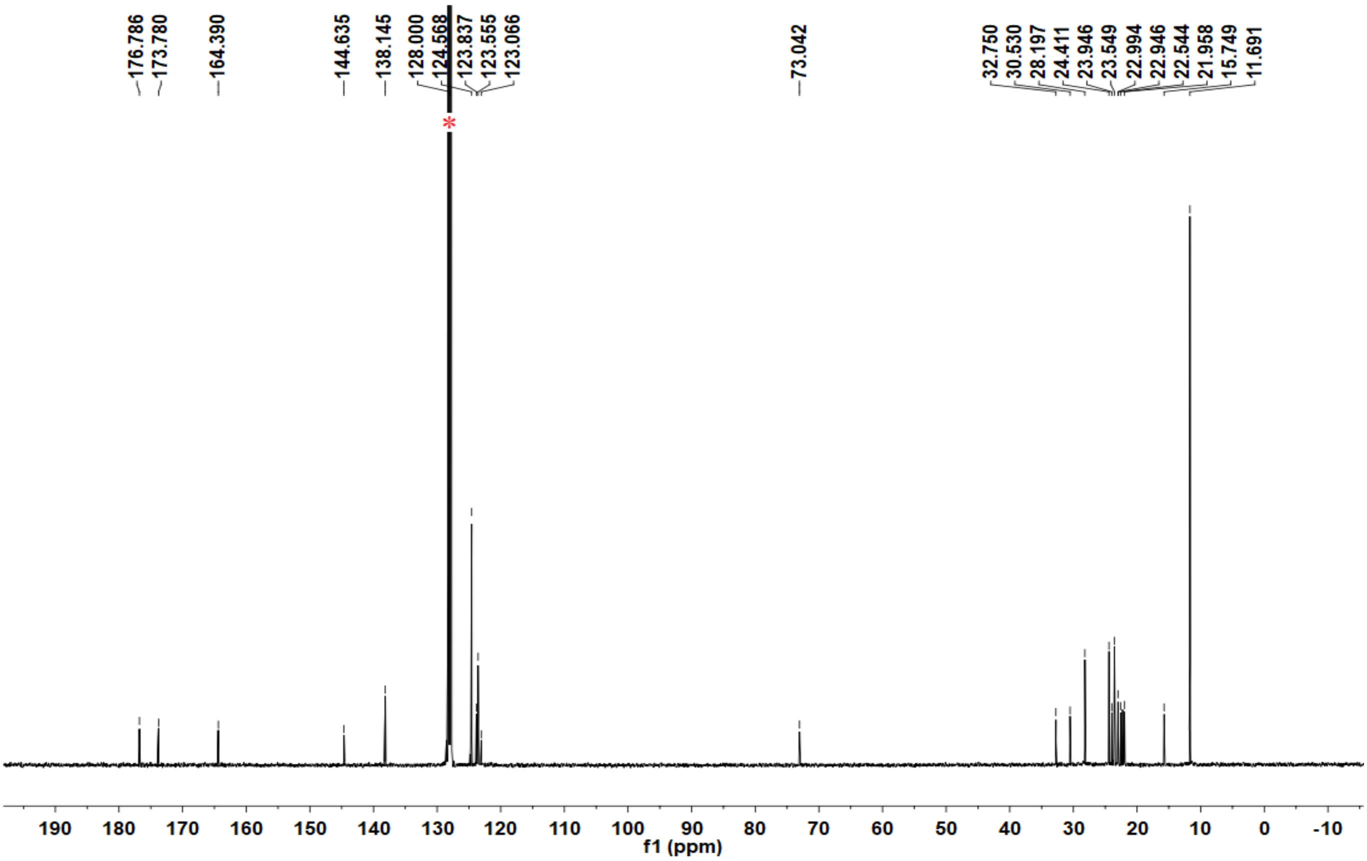

Figure S60. $^{13}C\{^{1}H\}$ NMR (C_6D_6; 20 ℃) spectrum for compound **28** (* solvent).

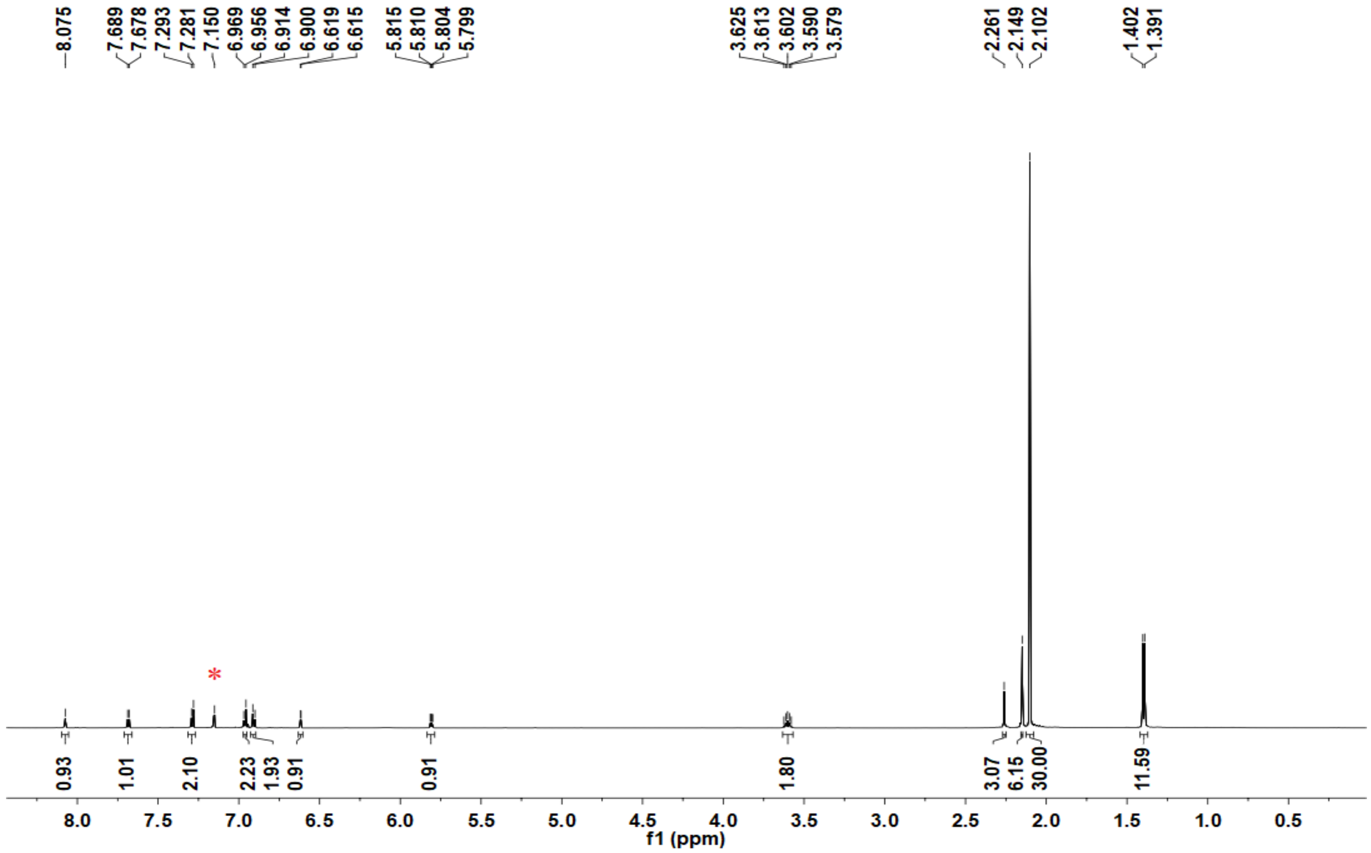

Figure S61. ^{1}H NMR (C_6D_6; 20 ℃) spectrum for compound **30** (* solvent).

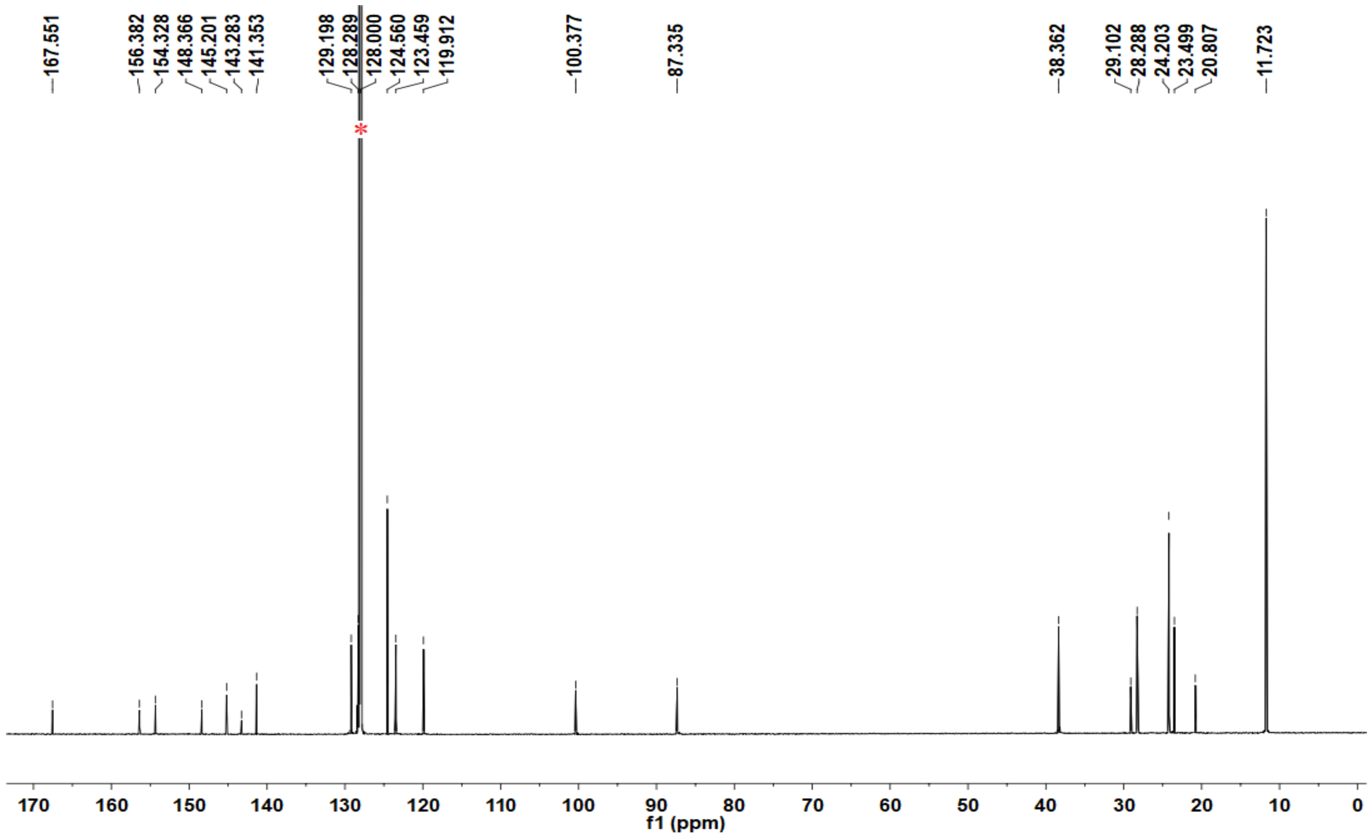

Figure S62. $^{13}C\{^{1}H\}$ NMR (C_6D_6; 20 ℃) spectrum for compound **30** (* solvent).

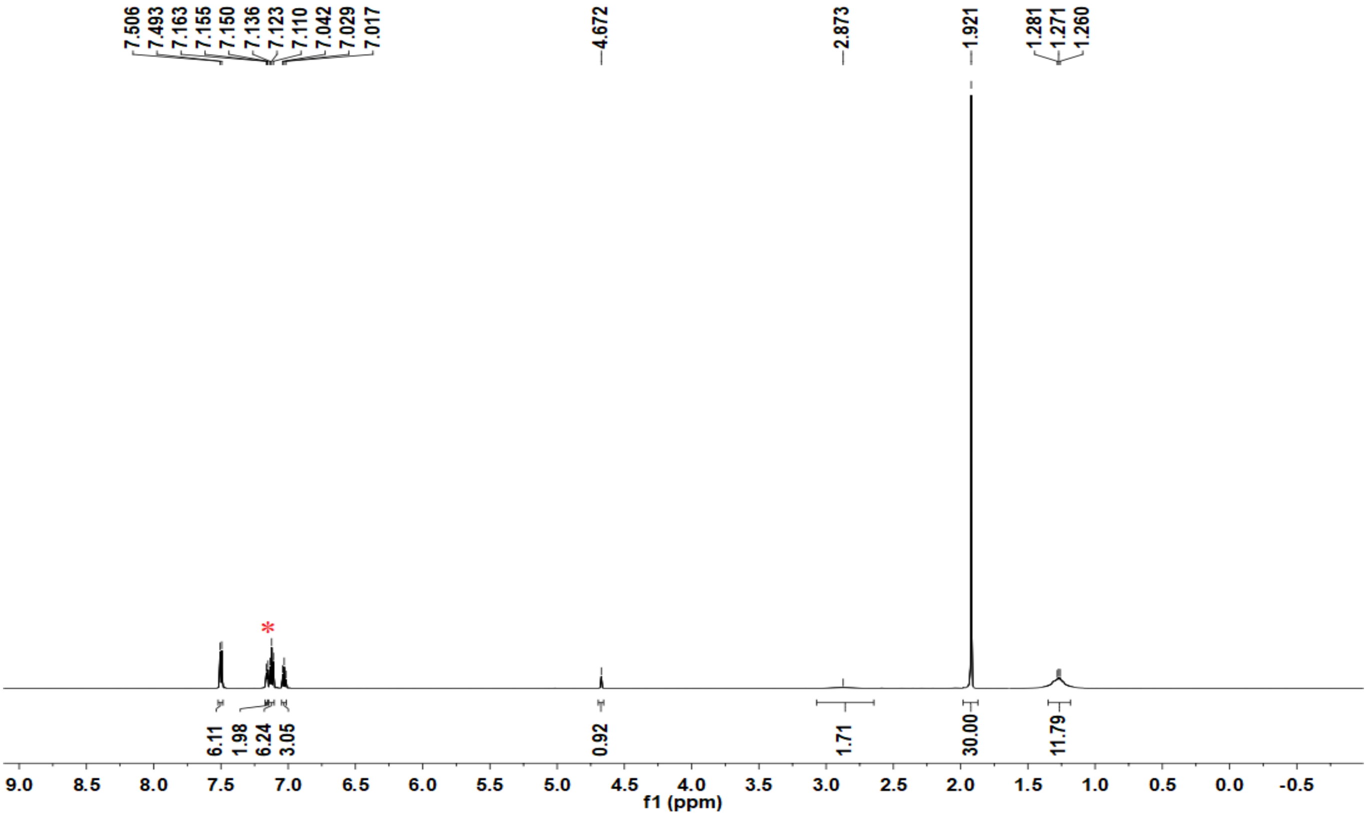

Figure S63. ^{1}H NMR (C_6D_6; 20 ℃) spectrum for compound **31** (* solvent).

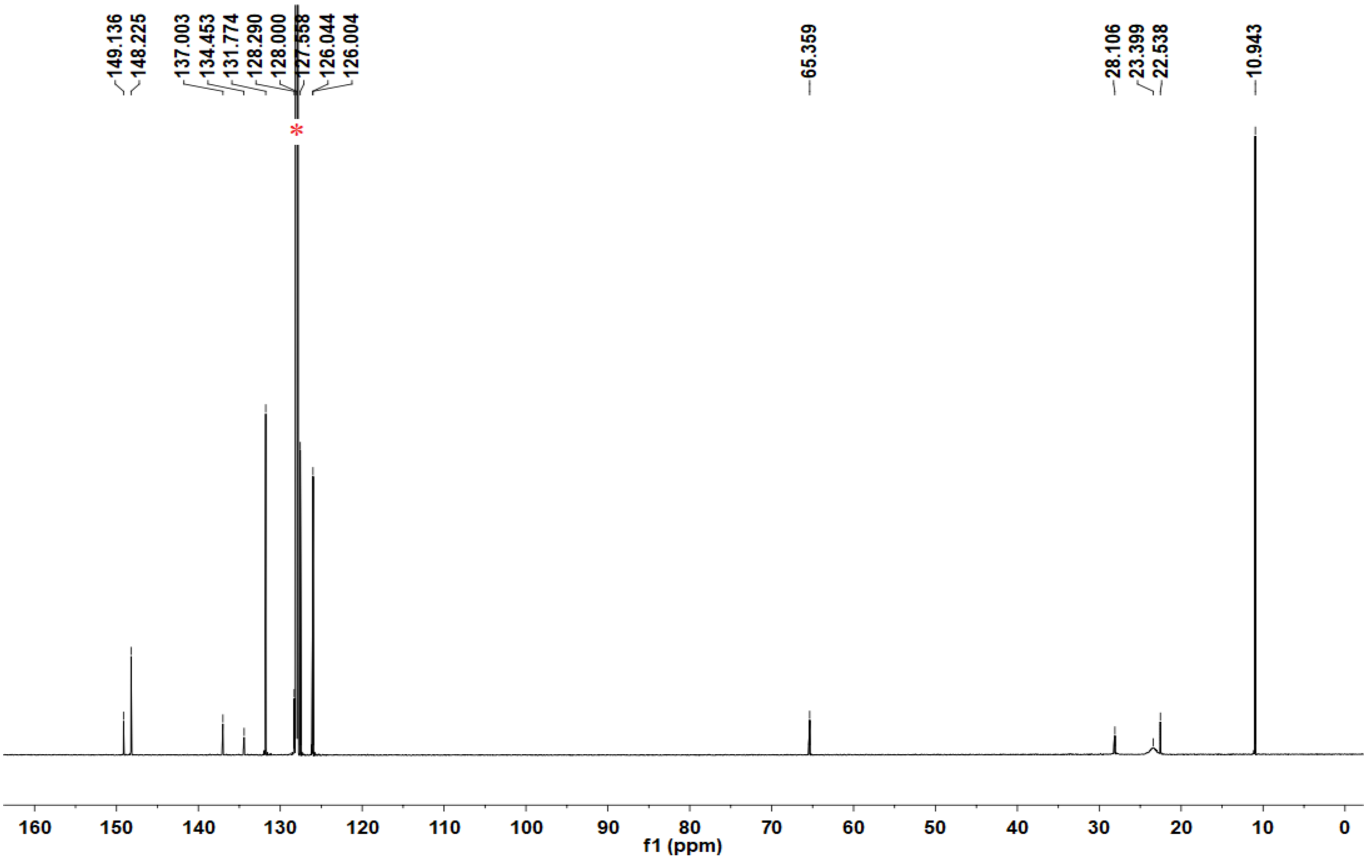

Figure S64. $^{13}C\{^{1}H\}$ NMR (C_6D_6; 20 ℃) spectrum for compound **31** (* solvent).

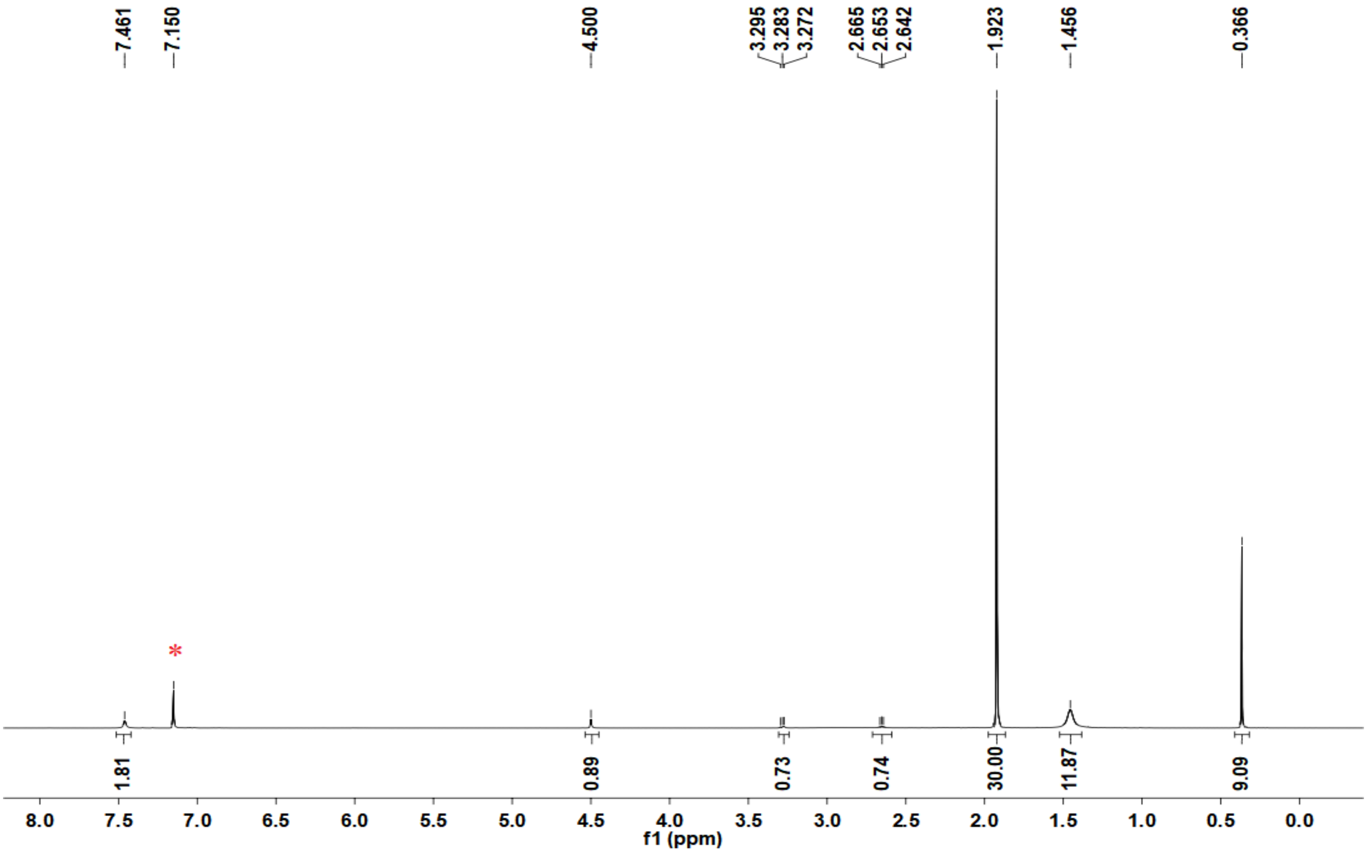

Figure S65. ^{1}H NMR (C_6D_6; 20 ℃) spectrum for compound **32** (* solvent).

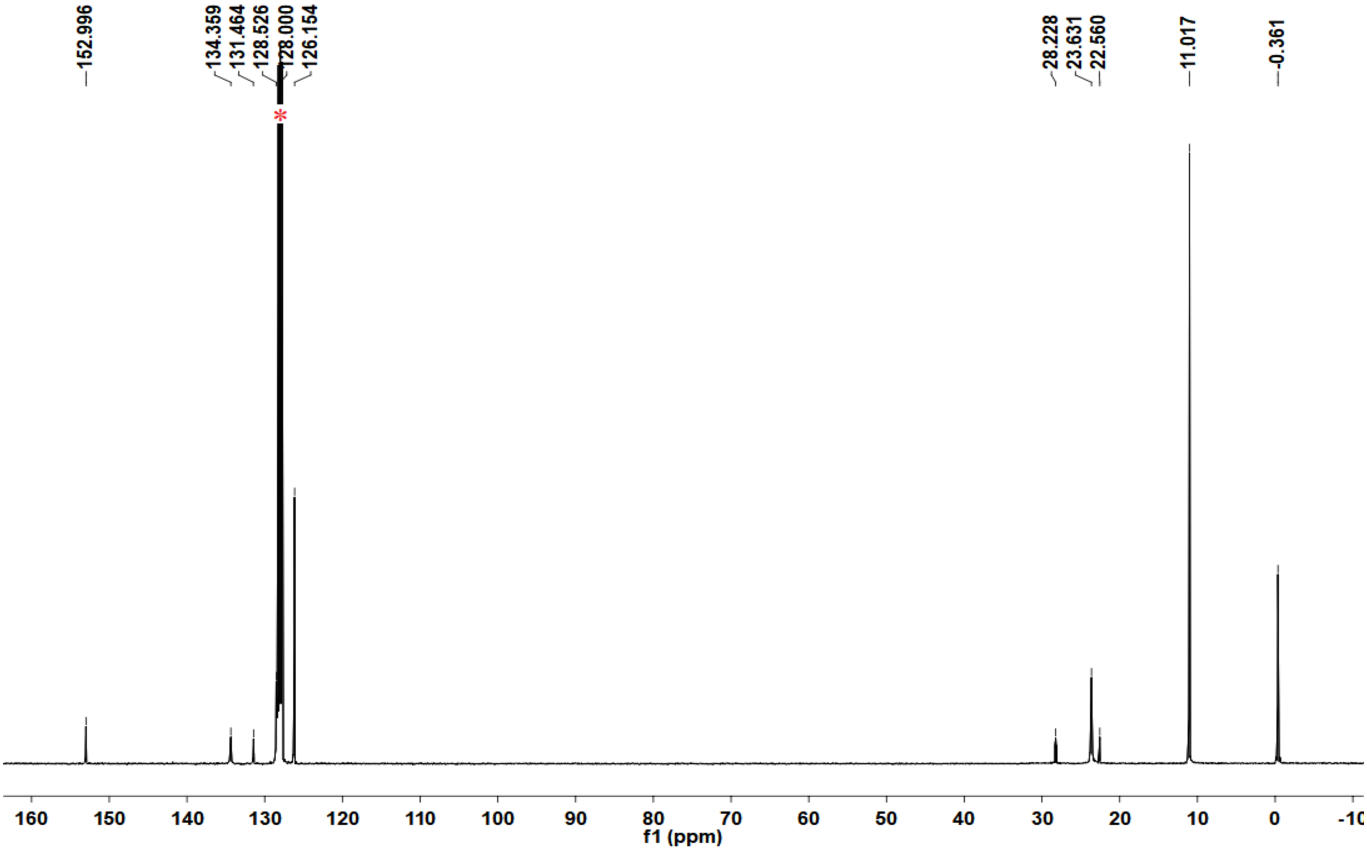

Figure S66. $^{13}C\{^{1}H\}$ NMR (C_6D_6; 20 ℃) spectrum for compound **32** (* solvent).

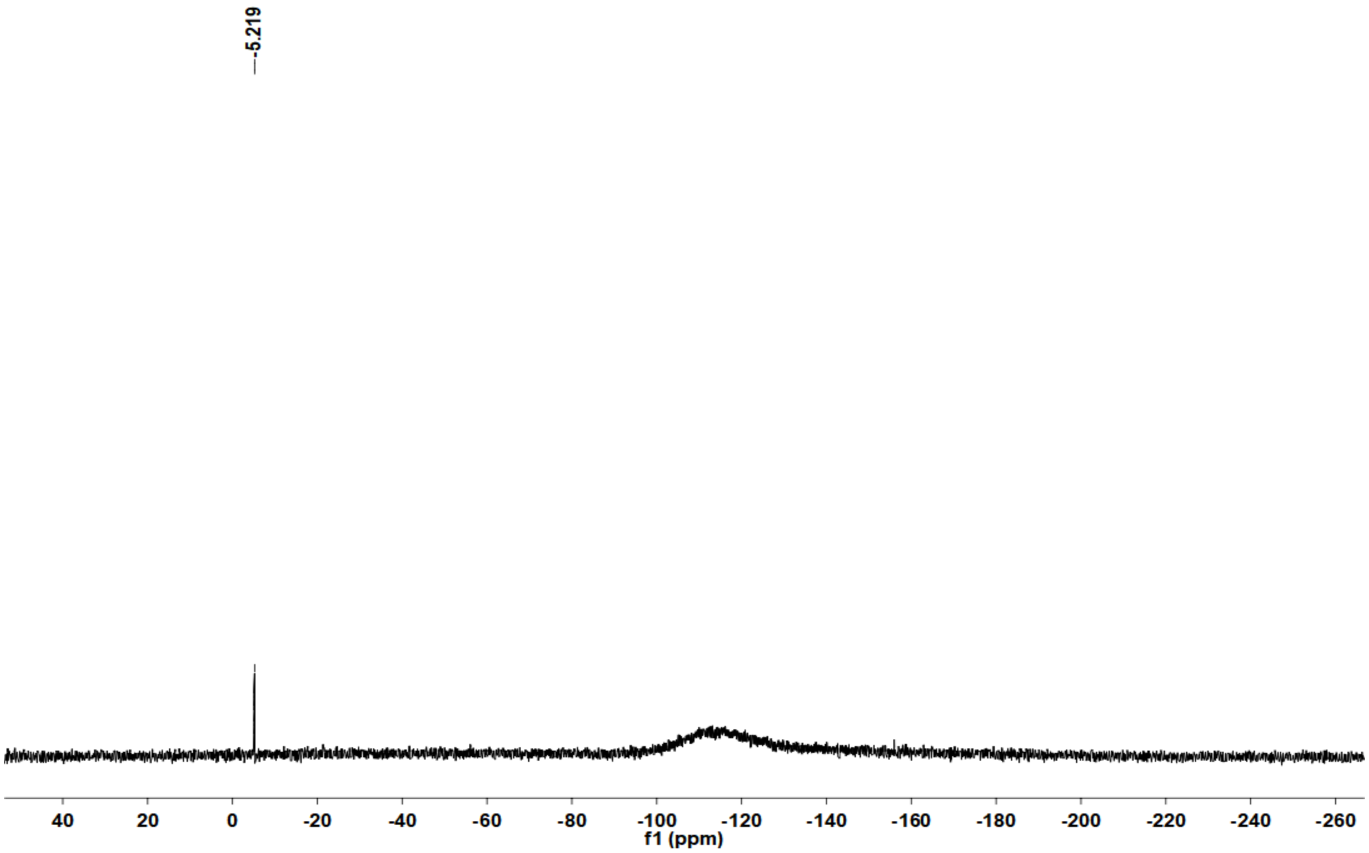

Figure S67. $^{29}Si\{^{1}H\}$ NMR (C_6D_6; 20 ℃) spectrum for compound **32**.

5. References

(1) Frisch, M. J.; Trucks, G. W.; Schlegel, H. B.; Scuseria, G. E.; Robb, M. A.; Cheeseman, J. R.; Scalmani, G.; Barone, V.; Mennucci, B.; Petersson, G. A.; Nakatsuji, H.; Caricato, M.; Li, X.; Hratchian, H. P.; Izmaylov, A. F.; Bloino, J.; Zheng, G.; Sonnenberg, J. L.; Hada, M.; Ehara, M.; Toyota, K.; Fukuda, R.; Hasegawa, J.; Ishida, M.; Nakajima, T.; Honda, Y.; Kitao, O.; Nakai, H.; Vreven, T.; Montgomery, J. A. Jr.; Peralta, J. E.; Ogliaro, F.; Bearpark, M.; Heyd, J. J.; Brothers, E.; Kudin, K. N.; Staroverov, V. N.; Kobayashi, R.; Normand, J.; Raghavachari, K.; Rendell, A.; Burant, J. C.; Iyengar, S. S.; Tomasi, J.; Cossi, M.; Rega, N.; Millam, J. M.; Klene, M.; Knox, J. E.; Cross, J. B.; Bakken, V.; Adamo, C.; Jaramillo, J.; Gomperts, R.; Stratmann, R. E.; Yazyev, O.; Austin, A. J.; Cammi, R.; Pomelli, C.; Ochterski, J. W.; Martin, R. L.; Morokuma, K.; Zakrzewski, V. G.; Voth, G. A.; Salvador, P.; Dannenberg, J. J.; Dapprich, S.; Daniels, A. D.; Farkas, O.; Foresman, J. B.; Ortiz, J. V.; Cioslowski, J.; Fox, D. J. *Gaussian 09*, Revision A.02, Gaussian, Inc.: Wallingford CT, 2009.

(2) Marenich, A. V.; Cramer, C. J.; Truhlar, D. G. Universal solvation model based on solute electron density and a continuum model of the solvent defined by the bulk dielectric constant and atomic surface tensions. *J. Phys. Chem. B*, **2009**, *113*, 6378-6396.

(3) (a) Küchle, W.; Dolg, M.; Stoll, H.; Preuss, H. Energy-adjusted pseudopotentials for the actinides. Parameter sets and test calculations for thorium and thorium monoxide. *J. Chem. Phys.* **1994**, *100*, 7535-7542. (b) Cao, X.; Dolg, M.; Stoll, H. Valence basis sets for relativistic energy-consistent small-core actinide pseudopotentials. *J. Chem. Phys.* **2003**, *118*, 487-496. (c) Cao, X.; Dolg, M. Segmented contraction scheme for small-core actinide pseudopotential basis sets. *J. Mol. Struct. (THEOCHEM)* **2004**, *673*, 203-209.
